# Supplementary material for: Photoelectrochemical Fe/Ni cocatalyzed C−C functionalization of alcohols
Source: Nat Commun. 2024 Jun 19;15:5245. doi: 10.1038/s41467-024-49557-7 (PMC11187109; doi:10.1038/s41467-024-49557-7)
Supplement: Supplementary file 1 — Supplementary information [file 41467_2024_49557_MOESM1_ESM.pdf]

Photoelectrochemical Fe/Ni Cocatalyzed C−C Functionalization of Alcohols

Long Zou[1,2], Rui Sun[1,2], Yongsheng Tao[1], Xiaofan Wang[1], Xinyue Zheng[1], and Qingquan Lu[1]*

[1]The Institute for Advanced Studies (IAS), Wuhan University, Wuhan 430072, P. R. China.

[2]L. Zou. and R. Sun. contributed equally.

gci2011@whu.edu.cn

Supplementary Information

Table of Contents

1 Supplementary Methods 3

1.1 General information 3

1.2 Description of the LED light source. 3

2 Supplementary Discussion..5

2.1 Synthesis of Starting Materials 5

2.2 Experimental Procedures..6

2.3 Graphical Guide for C−C Functionalization of Alcohols 7

2.4 Optimization of the Reaction Conditions...10

2.5 Scale-up Experiment. 11

2.6 Unsuccessful Substrates 12

2.7 Mechanistic Studies..12

2.8 Characterization of Products 20

3 NMR Spectra 45

4 Supplementary References...104

1 Supplementary Methods

1.1 General information

All reactions were conducted under an argon atmosphere unless otherwise noted. All reagents, unless otherwise stated, were directly used as received from commercial suppliers (Alfa, Tci, Innochem, Aladdin, Energy Chemical, etc.) without further purification. Thin layer chromatography (TLC) employed 0.25 mm glass silica gel plates. Visualization of spots on TLC plate was accomplished with short-wave UV light, and phosphomolybdic acid, $KMnO_4$ or staining over I_2 chamber. Flash chromatography column was packed with 200-300 mesh silica gel in petroleum (bp. 60-90 °C). Proton nuclear magnetic resonance (^1H NMR) spectra and carbon nuclear magnetic resonance (^{13}C NMR) spectra were recorded on JNM ECZ 400 (400 MHz) and AVANCE NEO 600 (600 MHz) spectrometers. All chemical shifts (δ) were reported in ppm and coupling constants (J) in Hz. All Chemical shifts are reported in parts per million downfield from tetramethylsilane and are referenced to residual undeuterated solvent ($CHCl_3$ at 7.26 ppm ^1H NMR, 77.16 ppm ^{13}C NMR,). Data are represented as follows: chemical shift, multiplicity (br = broad, s = singlet, d = doublet, t = triplet, q = quartet, m = multiplet). All compounds were characterized by high resolution mass spectra (HRMS) (Bruker UltiMate3000 & Compact). Gas chromatography-mass spectrometry (GC-MS) was recorded on an Agilent 8890-5977B MSD Series spectrometer. Gas chromatography (GC) was recorded on an Agilent 8890 Series spectrometer. Cyclic voltammetry studies were carried out on a CHI660E potentiostat. A glassy carbon disk, Pt wire, and Ag/AgCl (in saturated potassium chloride) were used as the working, counter, and reference electrodes, respectively.

1.2 Description of the LED light source.

Each light intensity was measured with an energy spectrometer (Photocatalytic energy spectrometer, Model WATTCAS PCS230850). The 20 W 398.9 nm LED light (emitting area: 23 × 23 mm) was assembled using twenty 398.9 nm chips in a compact fashion. The emitting wavelength of 20W LED (398.9 nm) laser was recorded in Supplementary

Figure 2.

Note: The photocatalytic energy spectrometer was placed vertically directly above the light source, and the light source continued to radiate for two minutes. Meanwhile, fan was enforced for effective thermal management to maintain luminous efficiency and life expectancy of LED light.

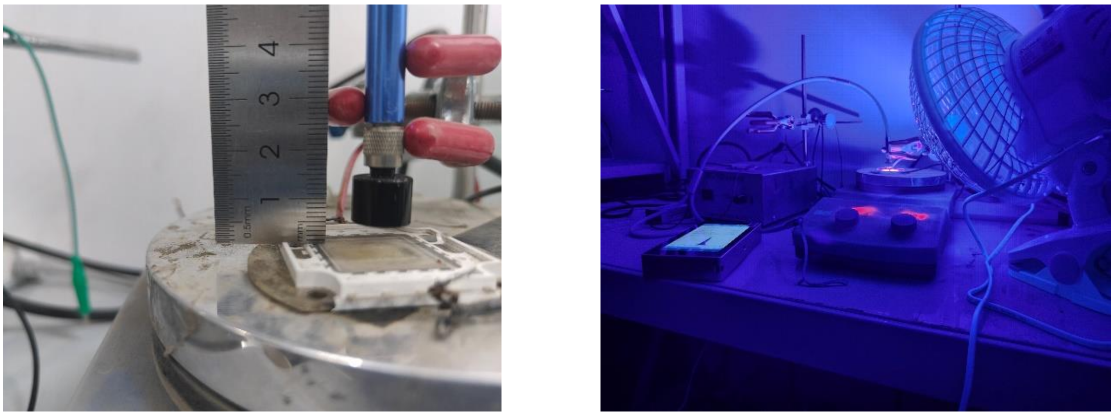

Supplementary Figure 1. Each light intensity was measured with an energy spectrometer

Distance (cm)	Illuminance (lx)	Ee (mW/cm^2)	Peak wavelength (nm)
0.5	647.0	834.313	398.9

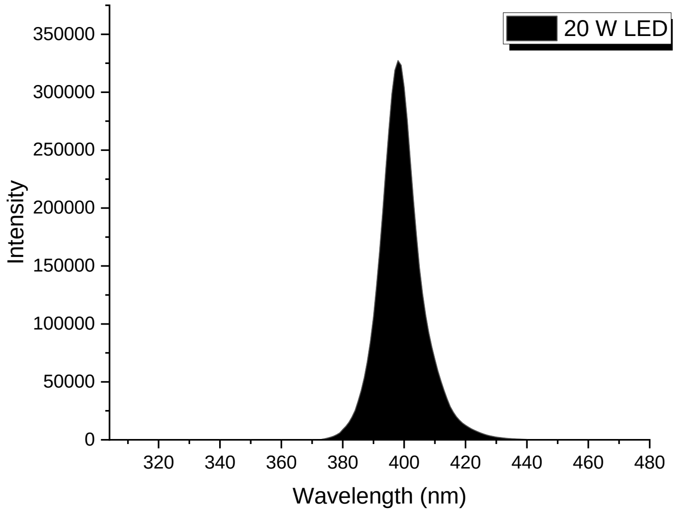

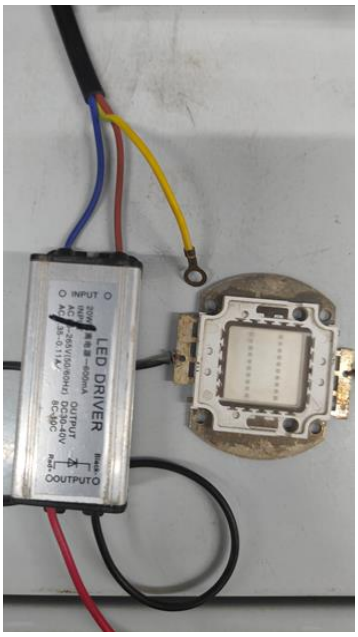

Supplementary Figure 2. The emission spectrum of the 20 W LED (398.9 nm) light.

2 Supplementary Discussion

2.1 Synthesis of Starting Materials

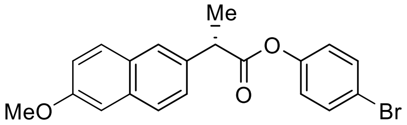

S1

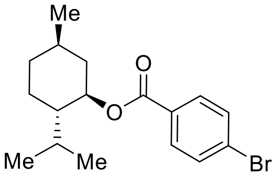

S2

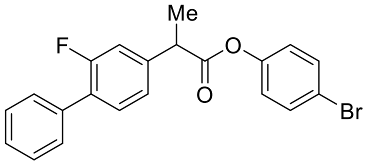

S3

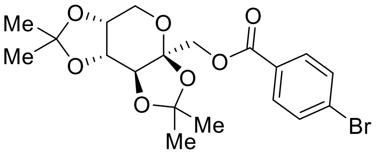

S4

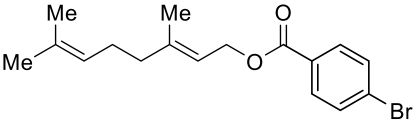

S5

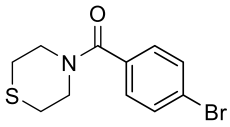

S6

Supplementary Figure 3. Substrates **S1**[1], **S2**[1], **S4**[2], **S5**[3], **S6**[4] were prepared according to reports in the literature.

General procedure A for synthesis of S3[1].

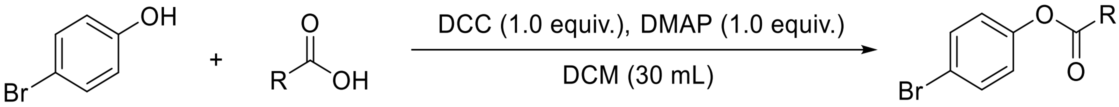

Supplementary Figure 4. A flask was charged with 4-bromophenol (1.838 g, 10.63 mmol, 1.25 equiv.), the corresponding acid (10.63 mmol, 1.25 equiv.) and 4-dimethylaminopyridine (1.038 g, 8.50 mmol, 1.0 equiv.) and then vacuum filled under argon. The solids were then suspended in CH_2Cl_2 (15 mL), and the reaction flask was placed in an ice/water bath. Dicyclohexylcarbodiimide (1.754 g, 8.50 mmol, 1.0 equiv.) in CH_2Cl_2 (15 mL) was then added dropwise to the reaction flask. After stirring for 12 h, the reaction was filtered through a pad of celite, which was washed with additional CH_2Cl_2 (20 mL). The organic solution was then washed with brine, dried over Na_2SO_4, and the solvent was removed via rotary evaporation. The crude solid was purified via silica gel chromatography to yield the desired product.

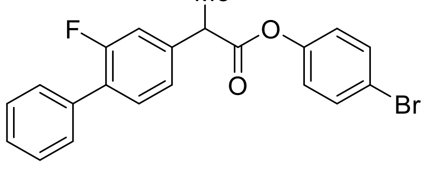

4-Bromophenyl 2-(2-fluoro-[1,1'-biphenyl]-4-yl)propanoate (S3):

This compound was prepared according to general procedure **A** from 4-bromophenol (1.838 g, 10.63 mmol, 1.0 equiv.), purification by silica gel column chromatography (petroleum ether/ethyl acetate) afforded **S3** (3.61 g, 85%) as colorless oil. **^{1}H NMR (600 MHz, Chloroform-*d*)** δ 7.37 (d, *J* = 8.4 Hz, 2H), 7.25 – 7.22 (m, 5H), 7.17 – 7.14 (m, 1H), 7.03 (d, *J* = 9.6 Hz, 2H), 6.72 (d, *J* = 8.8 Hz, 2H), 3.76 (q, *J* = 7.0 Hz, 1H), 1.43 (d, *J* = 7.0 Hz, 3H). **^{13}C NMR (151 MHz, Chloroform-*d*)** δ 170.2, 159.7 (d, *J* = 248.8 Hz), 149.7, 141.0 (d, *J* = 7.6 Hz), 135.2, 132.3, 131.0 (d, *J* = 4.0 Hz), 128.9 (d, *J* = 2.8 Hz), 128.5, 128.1 (d, *J* = 13.2 Hz), 127.8, 123.5 (d, *J* = 3.0 Hz), 123.1, 118.9, 115.3 (d, *J* = 23.8 Hz), 45.0, 18.3. **^{19}F NMR (565 MHz, Chloroform-*d*)** δ -116.74 (m). **HRMS (ESI)** m/z calcd. for $C_{21}H_{17}BrFO_2$ ($[M+H]^+$): 399.0390, found: 399.0382.

2.2 Experimental Procedures

General Procedure B:

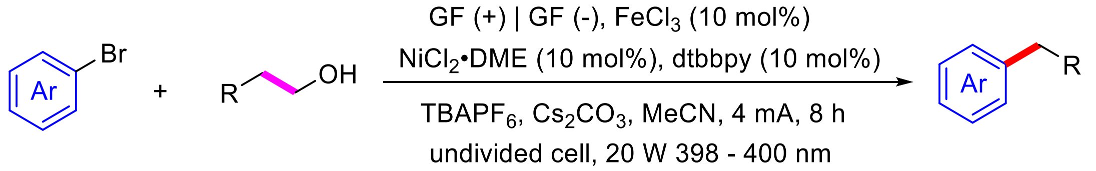

Supplementary Figure 5. In an oven-dried two-necked cell (20 mL) equipped with a Teflon-coated magnetic stir bar and two graphite felt electrodes (15 mm × 15 mm × 2.5 mm), Cesium carbonate (196 mg, 0.6 mmol, 2.0 equiv.), $TBAPF_6$ (116.2 mg, 0.3 mmol, 1.0 equiv.) and $FeCl_3$ (4.8 mg, 10 mol%) were added in a glovebox. The reaction cell was sealed and moved out from the glovebox. Afterwards, pre-catalyst solution (it was prepared by a mix of $NiCl_2$•DME (6.6 mg, 10 mol%), 4,4'-di-tert-butyl-2,2'-bipyridine (8.1 mg, 10 mol%) in anhydrous MeCN (6.0 mL) under argon atmosphere, and was stirred for 5 minutes.), aryl bromides (0.3 mmol, 1.0 equiv.) and alcohols (0.9 mmol, 3.0 equiv.) were added to the reaction cell via syringe. The reaction mixture was pre-stirred for 5 minutes and was electrolyzed at a constant current of 4 mA under irradiation by a 20 W purple LED lamp (0.3 cm away, with cooling fan to keep the reaction temperature at 25 ºC) for 8 h. After the reaction, the reaction mixture was concentrated (the residual product on electrodes were rinsed with EtOAc), and purified

by column chromatography (eluted with ethyl acetate/petroleum ether) to afford the pure product.

General Procedure C:

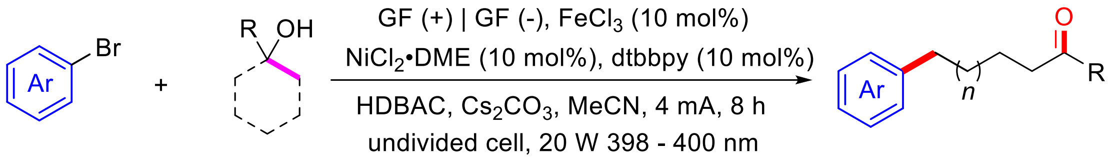

Supplementary Figure 6. In an oven-dried two-necked cell (20 mL) equipped with a Teflon-coated magnetic stir bar and two graphite felt electrodes (15 mm × 15 mm × 2.5 mm), Cesium carbonate (196 mg, 0.6 mmol, 2.0 equiv.), HDBAC (Benzyldimethylhexadecylammonium chloride) (118.8 mg, 0.3 mmol, 1.0 equiv.) and $FeCl_3$ (4.8 mg, 10 mol%) were added in a glovebox. The reaction cell was sealed and moved out from the glovebox. Afterwards, pre-catalyst solution (it was prepared by a mix of $NiCl_2$•DME (6.6 mg, 10 mol%), 4,4'-di-tert-butyl-2,2'-bipyridine (8.1 mg, 10 mol%) in anhydrous MeCN (6.0 mL) under argon atmosphere, and was stirred for 5 minutes.), aryl bromides (0.3 mmol, 1.0 equiv.) and alcohols (0.9 mmol, 3.0 equiv.) were added to the reaction cell via syringe. The reaction mixture was pre-stirred for 5 minutes and was electrolyzed at a constant current of 4 mA under irradiation by a 20 W purple LED lamp (0.3 cm away, with cooling fan to keep the reaction temperature at 25 ºC) for 8 h. After the reaction, the reaction mixture was concentrated (the residual product on electrodes were rinsed with EtOAc), and purified by column chromatography (eluted with ethyl acetate/petroleum ether) to afford the pure product.

2.3 Graphical Guide for C−C Functionalization of Alcohols

Materials used for set-up:

Graphite felt was purchased from Inner Mongolia Wanxing Carbon Co., Ltd. (http://wxcarbon.chemcp.com, Product Model: SMZ5MM). Electrode holder was purchased from Wuhan Gaossunion Technology Co., Ltd (www.gaossunion.com, Product Model: pt-3). All undivided cells were custom made by the Wuhan Ruiboer

Technology Co., Ltd.

Supplementary Figure 7. Materials used for two-necked cell.

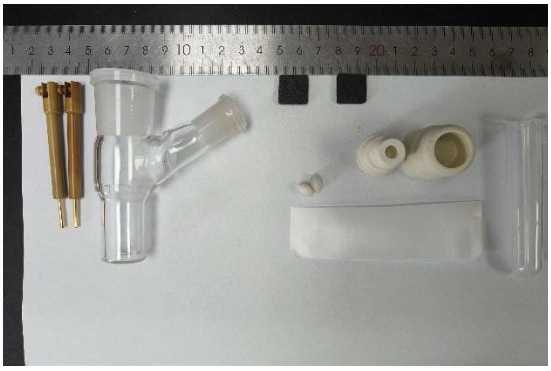

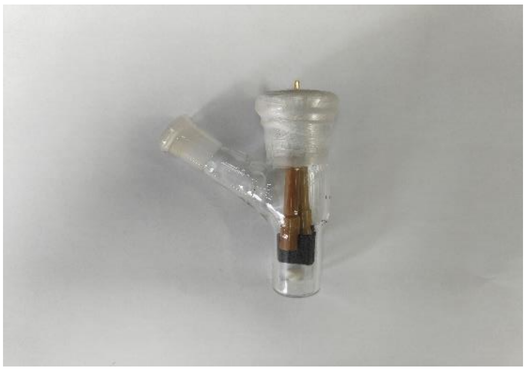

Supplementary Figure 8. Reagents used for reaction: Cesium carbonate, $TBAPF_6$, $FeCl_3$, $NiCl_2$•DME, 4,4'-di-tert-butyl-2,2'-bipyridine, anhydrous MeCN and the substrates (such as 4'-bromoacetophenone, 2-methyl-1-phenyl-2-propanol).

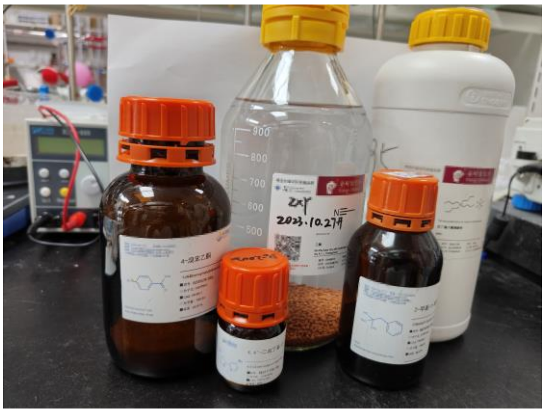

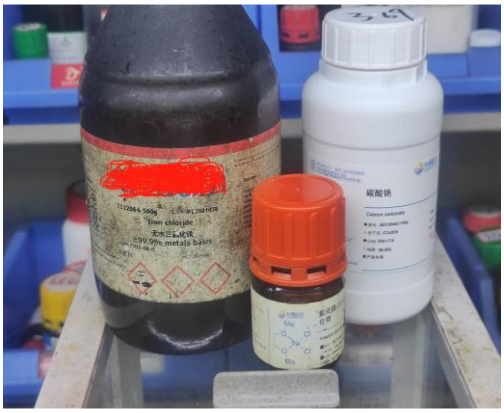

Supplementary Figure 9. $FeCl_3$ (4.8 mg, 10 mol%), Cesium carbonate (196 mg, 0.6 mmol, 2.0 equiv.) and $TBAPF_6$ (116.2 mg, 0.3 mmol, 1.0 equiv.) were added to the oven-dried two-necked cell equipped with a Teflon-coated magnetic stir bar in the glovebox. $NiCl_2$•DME (6.6 mg, 10 mol%) and 4,4'-di-tert-butyl-2,2'-bipyridine (8.1 mg, 10 mol%) were added to a PE tube for the preparation of pre-catalyst solution.

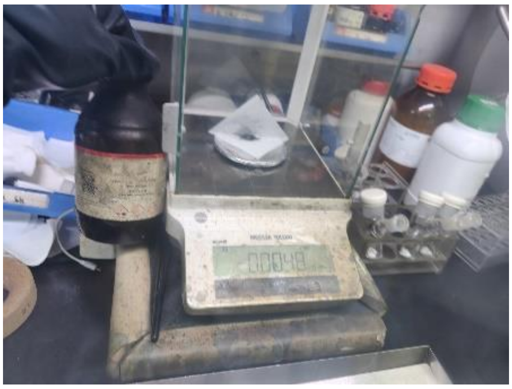

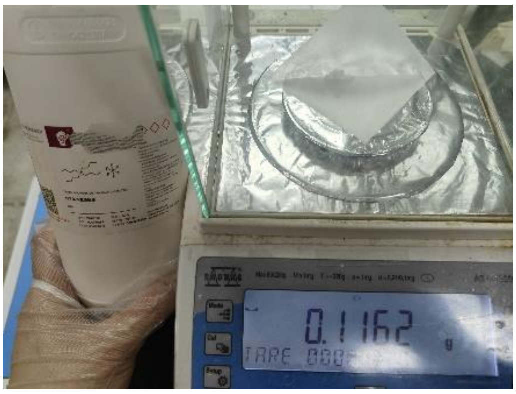

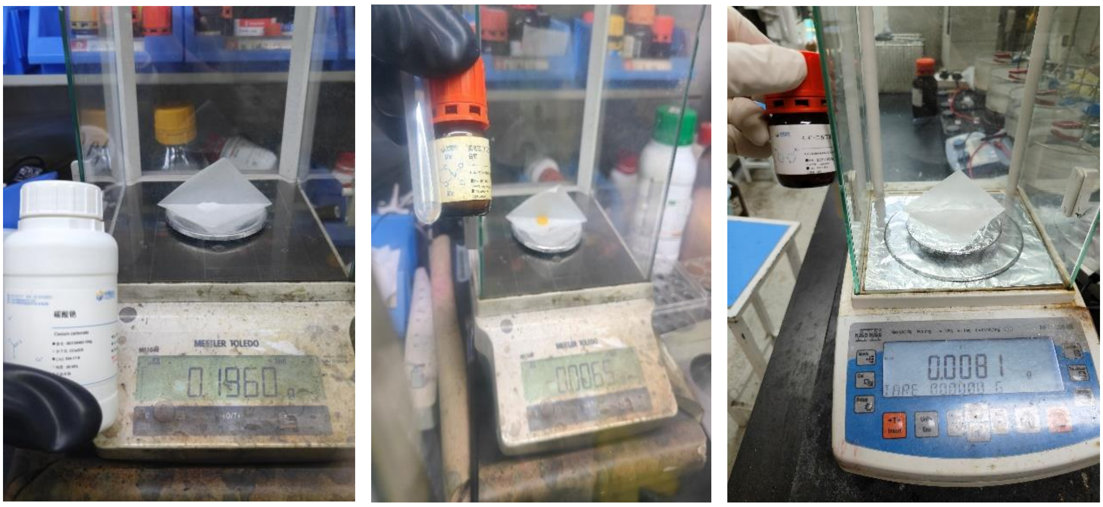

Supplementary Figure 10. The PE tube was sealed. Then, anhydrous acetonitrile (6.0 mL) was added to the PE tube and the pre-catalyst solution was stirred for 5 minutes. Afterwards, pre-catalyst solution, aryl bromides (0.3 mmol, 1.0 equiv.) and alcohols (0.9 mmol, 3.0 equiv.) were added to the reaction cell via syringe. The reaction mixture was pre-stirred for 5 minutes.

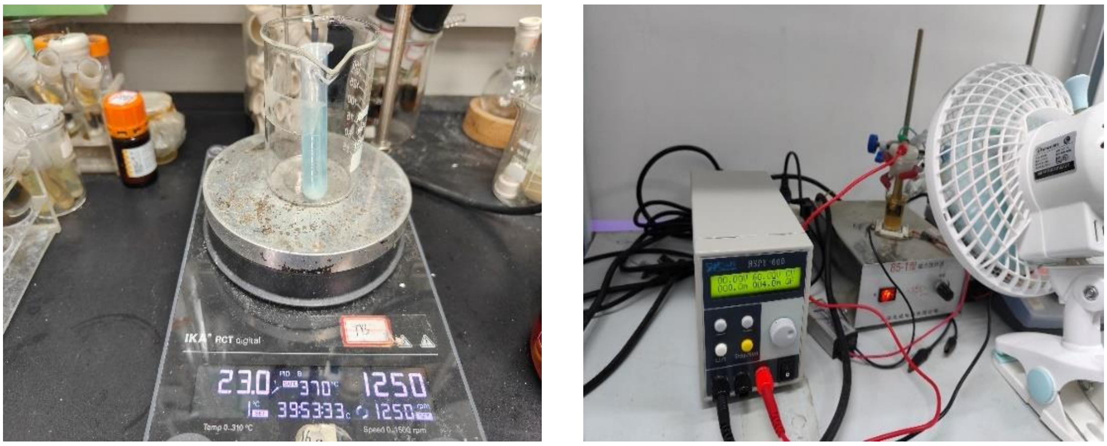

Supplementary Figure 11. The reaction mixture was electrolyzed at a constant current of 4 mA and irradiated with a 20 W purple LED lamp (0.3 cm away, with cooling fan to keep the reaction temperature at 25 ºC) for 8 h.

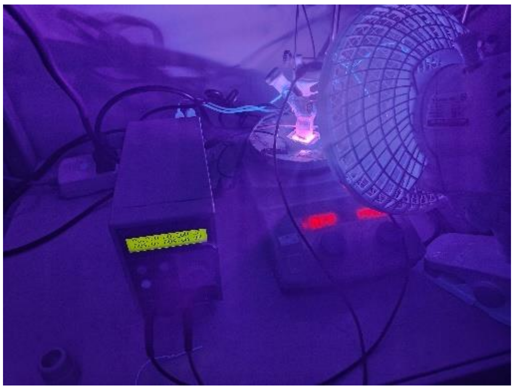

6. After the reaction, the reaction mixture was concentrated (the residual product on electrodes were rinsed with EtOAc), and purified by column chromatography (eluted with ethyl acetate/petroleum ether) to afford the pure product.

2.4 Optimization of the Reaction Conditions.

Supplementary Table 1: Optimization of the reaction conditions.

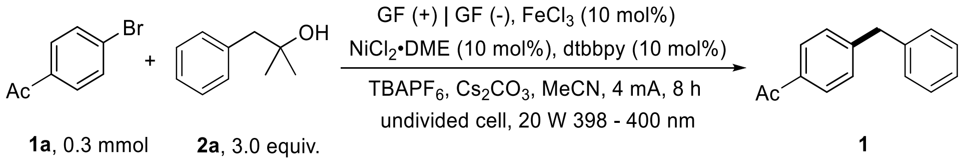

Entry	Variation from standard conditions	Yield (%)[b]
1	none	89 (85)
2	$CuCl_2$ as anodic catalyst	n.d.
3	$CeCl_3$ as anodic catalyst	n.d.
4	$CoCl_2$ as cathodic catalyst	n.d.
5	tpy[c] instead of dtbbpy	6
6	bpy[d] instead of dtbbpy	36
7	phenanthroline instead of dtbbpy	15
8	$LiClO_4$ instead of $TBAPF_6$	4
9	DMF instead of MeCN	8
10	THF instead of MeCN	9
11	10 W 398 – 400 nm	36
12	30 W 398 – 400 nm	63
13	I = 6 mA	70
14	w/o Cs_2CO_3	n.d.
15	w/o $FeCl_3$ or $NiCl_2$•DME	n.d.
16	w/o electricity or light	n.d.

[a]Reaction conditions: **1a** (0.3 mmol, 1.0 equiv.), **2a** (3.0 equiv.), $FeCl_3$ (10 mol%), $NiCl_2$•DME (10 mol%), 4,4'-di-tert-butyl-2,2'-bipyridine (10 mol%), $TBAPF_6$ (1.0 equiv.), Cs_2CO_3 (2.0 equiv.), anhydrous MeCN (6.0 mL), 4 mA, 8 h, 20 W 398 - 400 nm, fan, argon, graphite felt as anode and cathode (1.5 cm × 1.5 cm × 0.25 cm), undivided cell, [b]GC yields using biphenyl as an internal standard, (isolated yields in parentheses). [c]tpy = 2,6-bis(2-pyridyl)pyridine. [d]bpy = 2,2'-bipyridine. n.d. = not detected. w/o = without.

2.5 Scale-up Experiment.

Supplementary Figure 12. Scale-up experiment for the synthesis of product **1**.

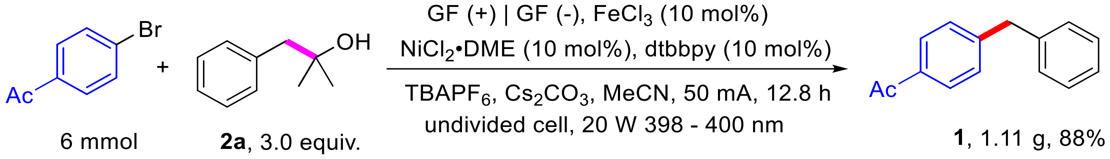

Reaction conditions: **1a** (6 mmol, 1.0 equiv.), **2a** (3.0 equiv.), $FeCl_3$ (10 mol%), $NiCl_2$•DME (10 mol%), 4,4'-di-tert-butyl-2,2'-bipyridine (10 mol%), $TBAPF_6$ (1.0 equiv.), Cs_2CO_3 (2.0 equiv.), anhydrous MeCN (100 mL), 50 mA, 12.8 h, 20 W 398 - 400 nm, fan, argon, graphite felt as anode and cathode (3.5 cm × 4.0 cm × 0.25 cm), undivided cell.

Supplementary Figure 13. Materials used for scale-up experiment.

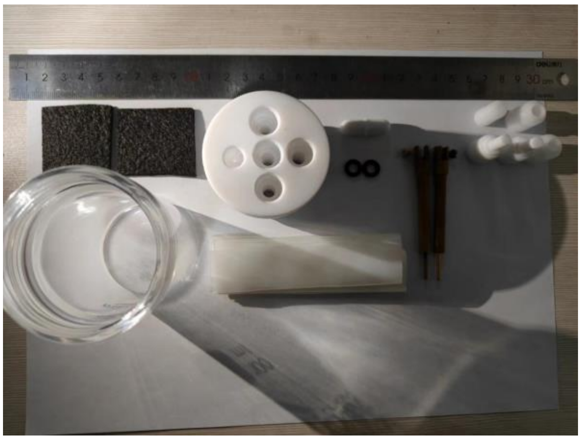

Supplementary Figure 14. The reaction mixture was electrolyzed at a constant current of 50 mA and irradiated with three 20 W purple LED lamps (1.0 – 1.5 cm away, with cooling fan to keep the reaction temperature at 25 ºC) for 12.8 h.

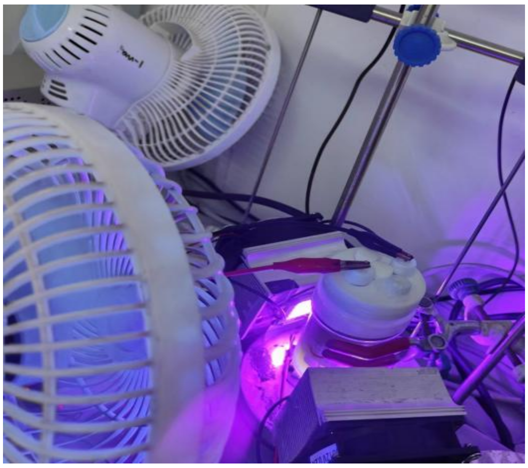

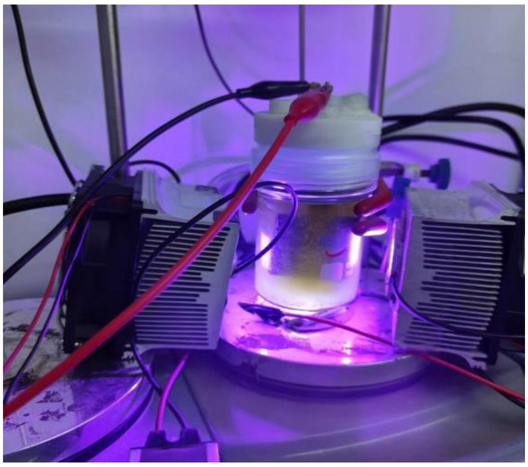

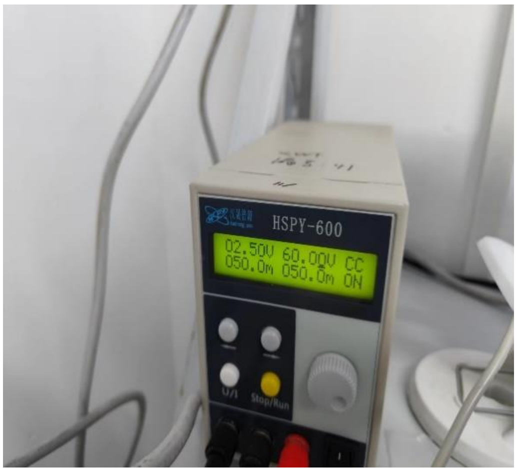

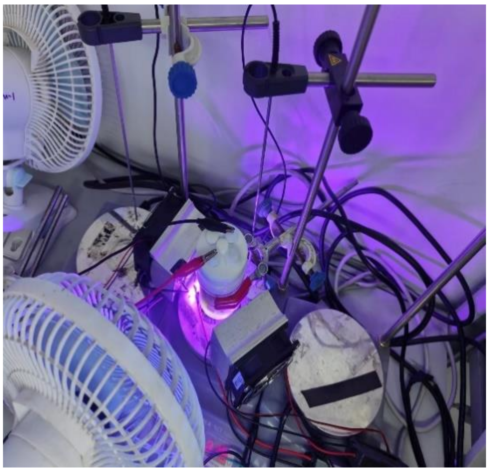

4. After the reaction, the reaction mixture was concentrated (the residual product on electrodes were rinsed with EtOAc), and purified by column chromatography (eluted with ethyl acetate/petroleum ether) to afford the pure desired product.

2.6 Unsuccessful Substrates

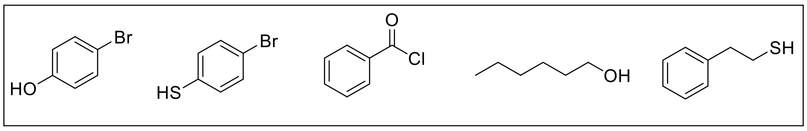

2.7 Mechanistic Studies

2.7.1 Cyclic Voltammetry Experiments

General information: Cyclic voltammetry (CV) experiments were conducted in a 20 mL two-necked cell set-up fitted with a glassy carbon working electrode (3 mm in diameter), an Ag/AgCl reference electrode, and a platinum wire counter electrode.

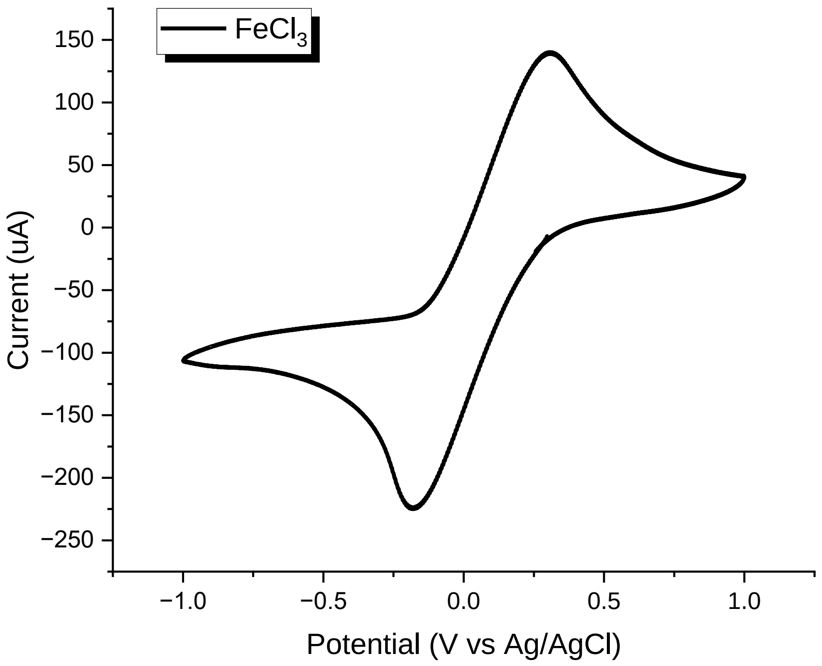

Supplementary Figure 15. Cyclic voltammograms recorded on a glassy carbon electrode at 300 mVs^{-1}. Black line: $FeCl_3$ (0.05 mmol), $TBAPF_6$ (0.10 mmol) in 6.0 mL MeCN.

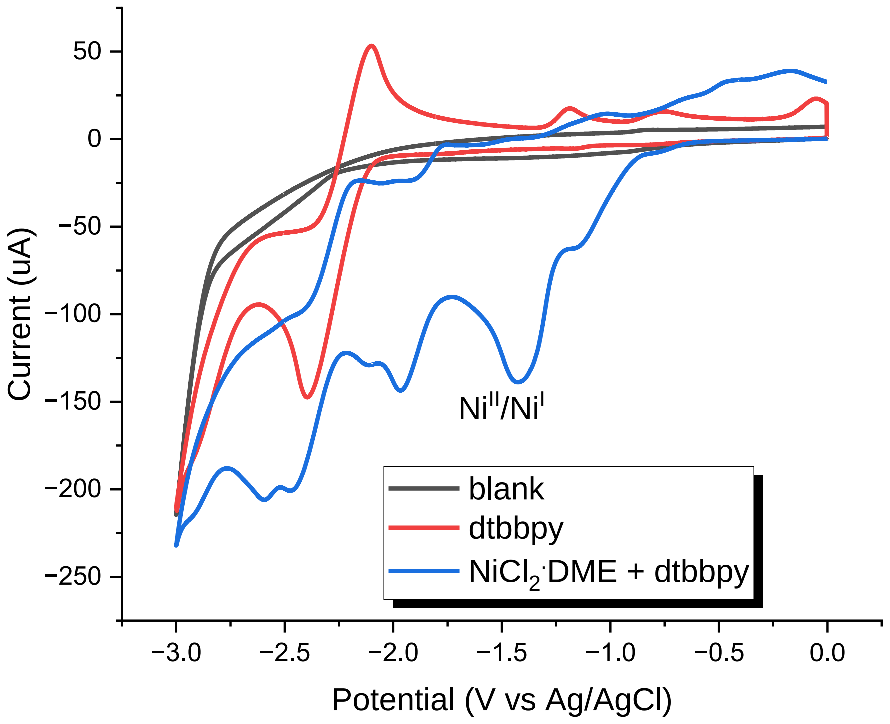

Supplementary Figure 16. Cyclic voltammograms recorded on a glassy carbon electrode at 100 mVs^{-1}. Black line: $^{n}Bu_4NPF_6$ (0.10 mmol) in 6.0 mL MeCN. Red line: dtbbpy (0.05 mmol), $^{n}Bu_4NPF_6$ (0.10 mmol) in 6.0 mL MeCN. Blue line: $NiCl_2$•DME (0.05 mmol), dtbbpy (0.05 mmol), $^{n}Bu_4NPF_6$ (0.10 mmol) in 6.0 mL MeCN.

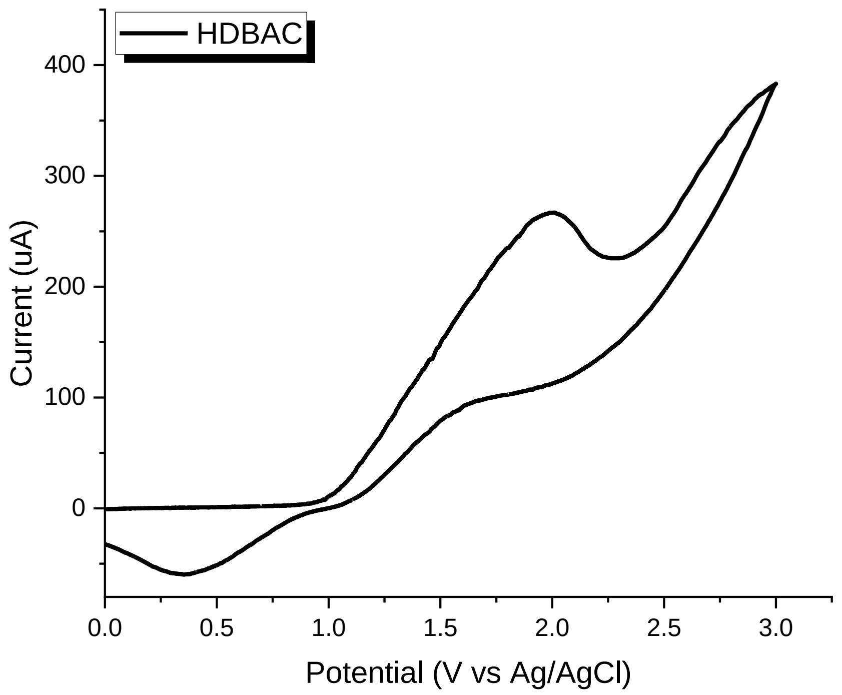

Supplementary Figure 17. Cyclic voltammograms recorded on a glassy carbon electrode at 100 mVs^{-1}. Black line: HDBAC (0.05 mmol), $TBAPF_6$ (0.10 mmol) in 6.0 mL MeCN.

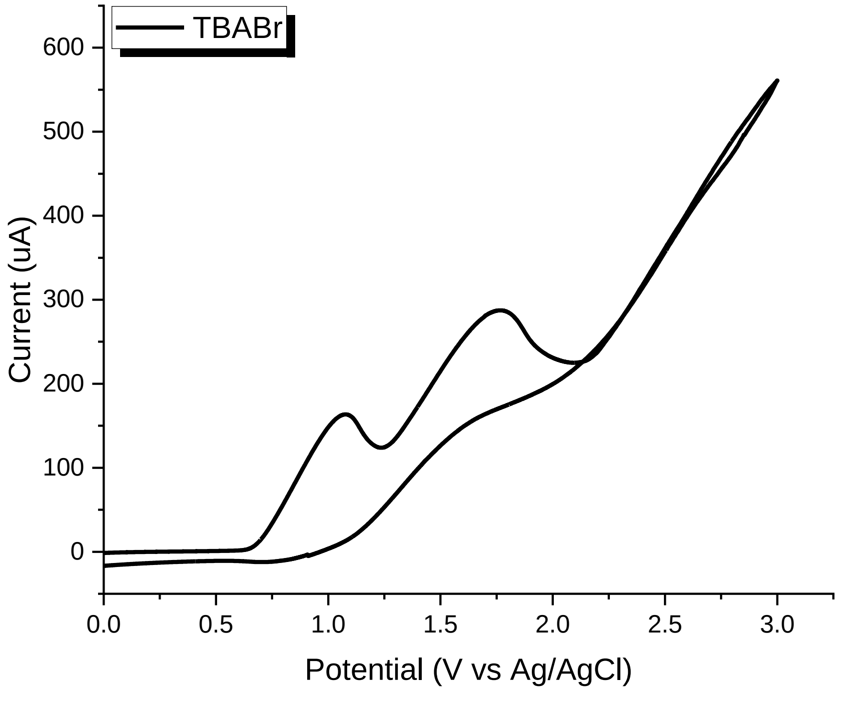

Supplementary Figure 18. Cyclic voltammograms recorded on a glassy carbon electrode at 100 mVs^{-1}. Black line: TBABr (0.05 mmol), $TBAPF_6$ (0.10 mmol) in 6.0 mL MeCN.

2.7.2 Radical trapping experiment.

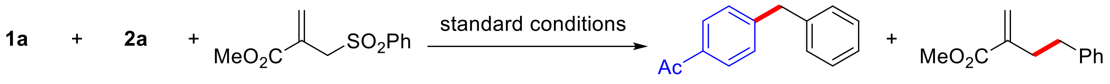

2.0 equiv. **1**, n.d. **59**, 7%

Supplementary Figure 19. Radical trapping experiment.

According to the general procedure **B**, the model reaction was carried out under standard conditions in the presence of methyl 2-((phenylsulfonyl)methyl)acrylate (2.0 equiv.). After the reaction, the reaction mixture was concentrated (the residual product on electrodes were rinsed with EtOAc), and purified by column chromatography (eluted with ethyl acetate/petroleum ether) to afford the pure product **59**.

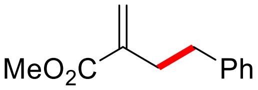

Methyl 2-methylene-4-phenylbutanoate (59)[5]**:** Yellow oil was obtained with 7% isolated yield (based on alkene, 0.6 mmol scale, 8.0 mg).

^{1}H NMR (600 MHz, Chloroform-*d*) δ 7.30 – 7.28 (m, 2H), 7.20 – 7.18 (m, 3H), 6.16 (s, 1H), 5.51 (s, 1H), 3.77 (s, 3H), 2.80 (t, J = 8.0 Hz, 2H), 2.63 (t, J = 8.0 Hz, 2H).

^{13}C NMR (151 MHz, Chloroform-*d*) δ 167.7, 141.5, 140.0, 128.6, 128.5, 126.1, 125.5, 52.0, 35.0, 34.0.

2.7.3 Kinetic studies

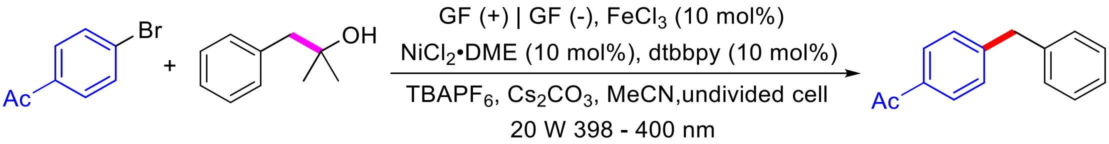

1a, 0.3 mmol **2a**, 3.0 equiv. **1**

Supplementary Figure 20. According to the general procedure **B**, the model reactions were conducted at 2 mA, 4 mA, 6 mA and 8 mA, respectively, in which biphenyl (0.1 mmol each) was employed as internal standard. In each reaction, samples of 0.1 mL were taken from the reaction cell at 60 min, 90 min, 120 min, 150 min, 180 min, respectively. The organic phase was filtered through a 1-2 cm filter pipe which was filled with silica gel. The crude mixture was analyzed by GC spectroscopy. The initial rates were calculated and plotted against the reactant concentration to determine the

reaction orders.

Supplementary Table 2. The concentrations of **1** (10^{-3} M) at different time in various **current**.

**C1** (10^{-3} M) / Time / **I** (mA)	60 min	90 min	120 min	150 min	180 min
2.0	0.81	1.04	1.54	2.06	2.68
4.0	1.0	1.64	2.44	3.32	4.39
6.0	1.58	2.44	3.48	4.87	6.21
8.0	1.28	2.24	3.81	5.63	7.20

Note: **C1** = the concentrations of **1**

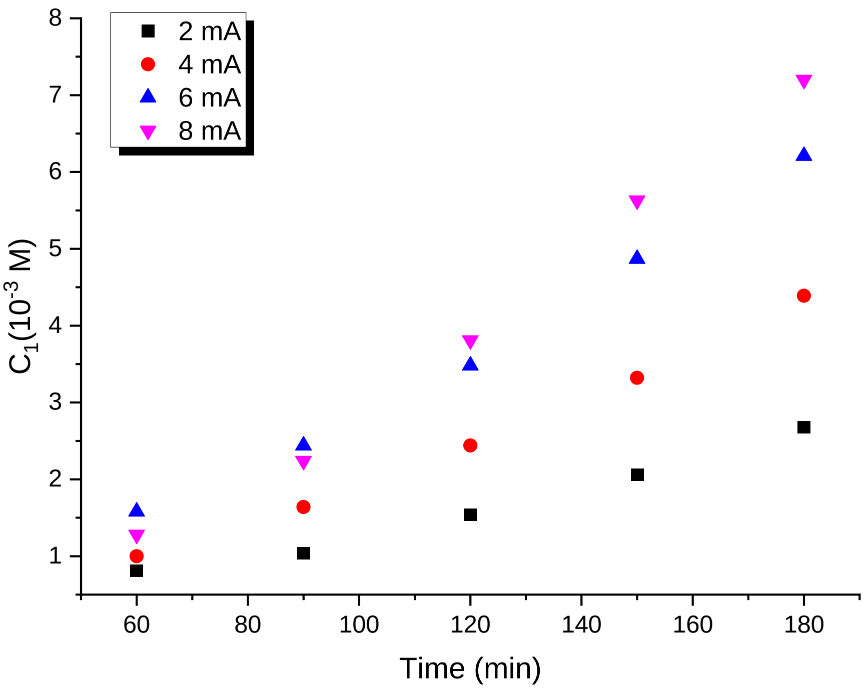

Supplementary Figure 21. The concentrations of product **1** at different time in various current.

Supplementary Table 3. The initial rates with various **current**.

**I** (mA)	2.0	4.0	6.0	8.0
Initial Rate (10^{-4} M/min)	0.01587	0.0282	0.03897	0.05077

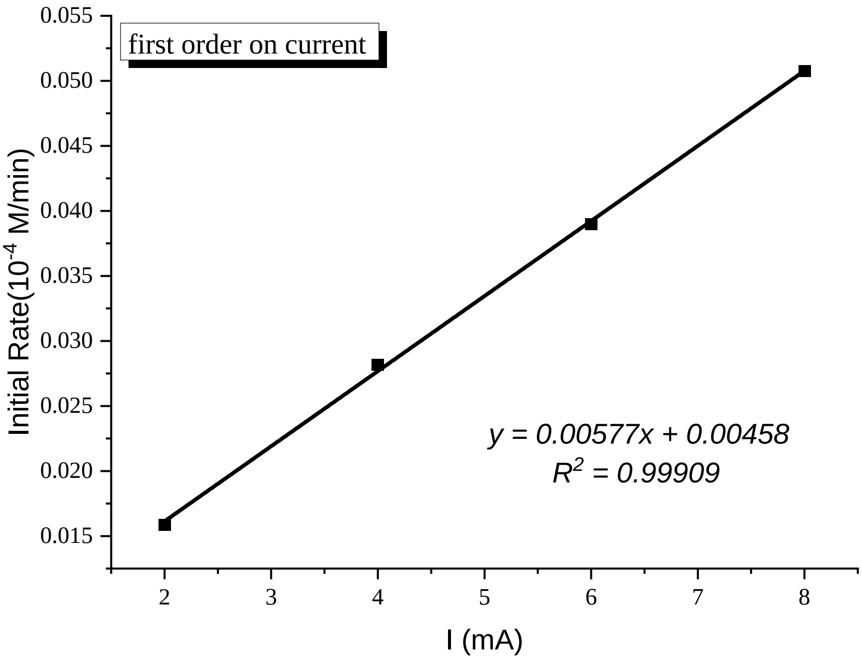

Supplementary Figure 22. Linear relation between the initial rates and current.

2.7.4 Electrode Voltage Over the Course of Electrolysis.

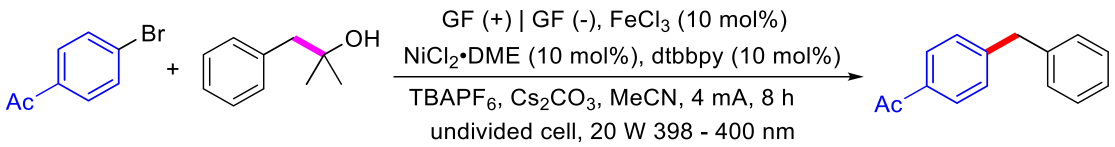

1a, 0.3 mmol **2a**, 3.0 equiv. **1**

Supplementary Figure 23. According to the general procedure **B**, the electrode potential was monitored under standard conditions using Ag/AgCl as a reference electrode. The electrodes (graphite felts) and reference electrode were inserted into the reaction mixture together during the electrolysis. A multimeter was used to detect electrode potential between anode/cathode and reference electrode respectively (keep anode or cathode with reference electrode as close as possible when testing).

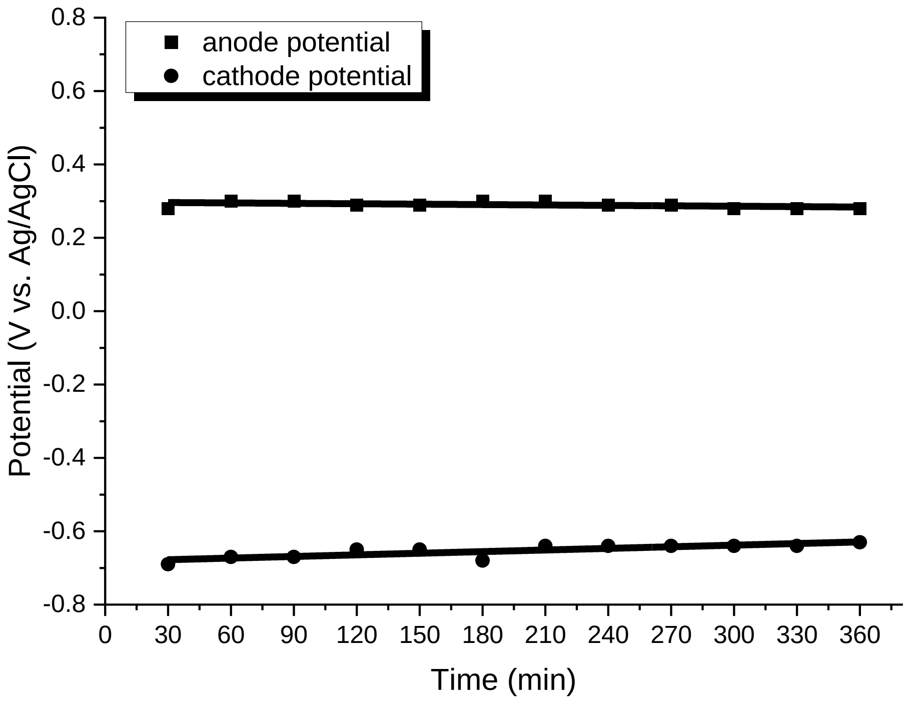

Supplementary Figure 24. Electrode voltage over the course of electrolysis.

2.7.5 Constant Voltage Electrolysis

2.7.5.1 Constant Voltage Electrolysis of Chloride anion

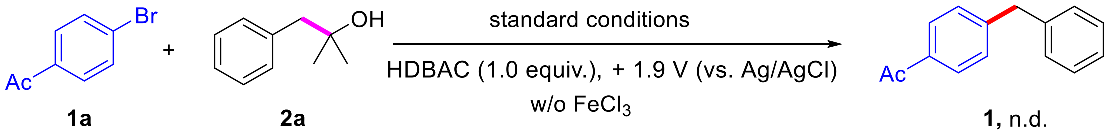

Supplementary Figure 25. According to the general procedure **B**, the reaction mixture was electrolyzed at a constant potential of 1.9 V (vs. Ag/AgCl) by employing 4'-bromoacetophenone (0.3 mmol), **2a** (0.9 mmol, 3.0 equiv.) and HDBAC (0.3 mmol, 1.0 equiv.) as substrates in the absence of $FeCl_3$. The reaction mixture was then analyzed by gas chromatography to obtain the yield of **1** using biphenyl as an internal standard.

2.7.5.2 Constant Voltage Electrolysis of Bromine anion

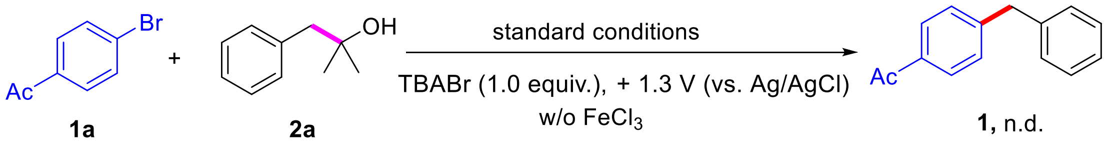

Supplementary Figure 26. According to the general procedure **B**, the reaction mixture was electrolyzed at a constant potential of 1.3 V (vs. Ag/AgCl) by employing 4'-

bromoacetophenone (0.3 mmol), **2a** (0.9 mmol, 3.0 equiv.) and TBABr (0.3 mmol, 1.0 equiv.) as substrates in the absence of $FeCl_3$. The reaction mixture was then analyzed by gas chromatography to obtain the yield of **1** using biphenyl as an internal standard.

2.7.6 The reaction of 4-bromoacetophenone with 2-methyl-1-phenyl-2-propanol.

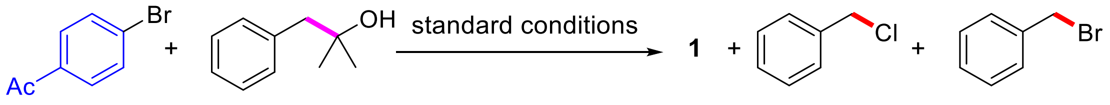

1a 2a 89% n.d. n.d.

Supplementary Figure 27. According to the general procedure **B**, the reaction was carried out under standard conditions. The reaction mixture was then analyzed by gas chromatography to obtain the yield of product using biphenyl as an internal standard.

Supplementary Figure 28. The reaction mixture was analyzed by GC-MS.

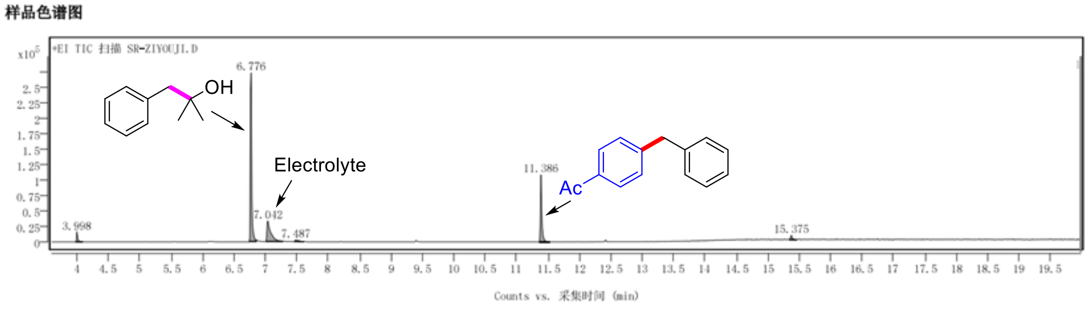

Supplementary Figure 29. The compound benzyl chloride was analyzed by GC-MS.

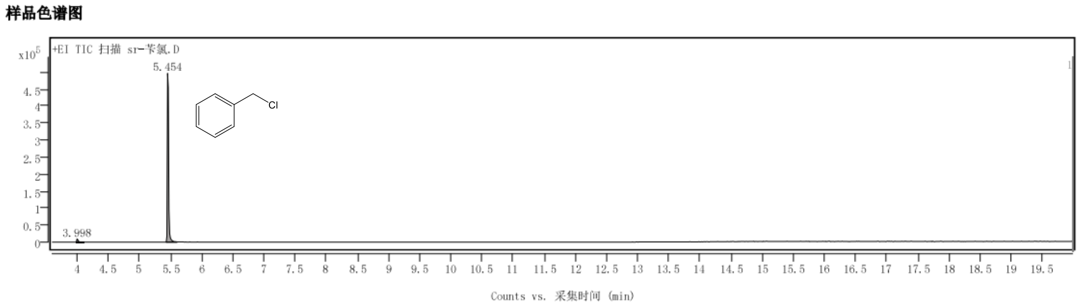

Supplementary Figure 30. The compound benzyl bromide was analyzed by GC-MS.

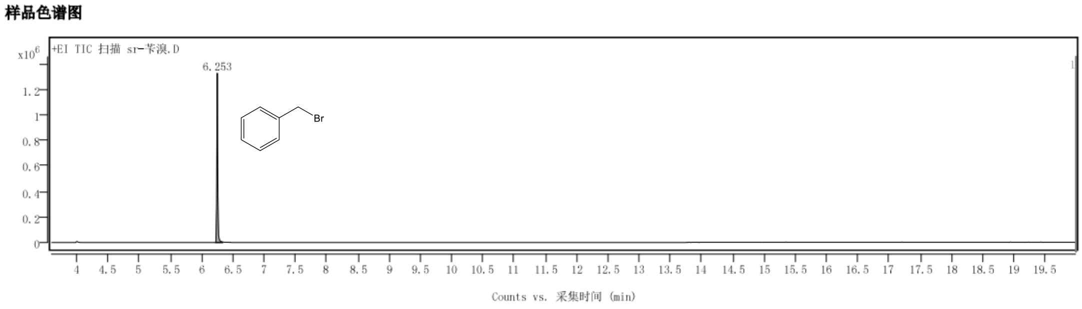

2.8 Characterization of Products

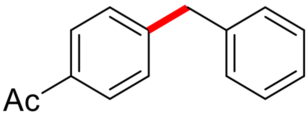

1-(4-Benzylphenyl)ethan-1-one (1)[6]**:** Yellow oil was obtained with 85% isolated yield following the general procedure B (0.3 mmol scale, 53.6 mg, average yield of two times).

^{1}H NMR (400 MHz, Chloroform-*d*) δ 7.89 – 7.85 (m, 2H), 7.30 – 7.25 (m, 4H), 7.22 – 7.19 (m, 1H), 7.18 – 7.15 (m, 2H), 4.01 (s, 2H), 2.54 (s, 3H).

^{13}C NMR (101 MHz, Chloroform-*d*) δ 197.8, 146.7, 140.1, 135.3, 129.2, 129.0, 128.6(9), 128.6(9), 126.5, 41.9, 26.6.

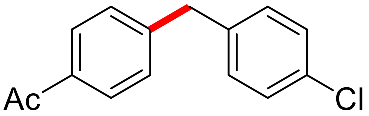

1-(4-(4-Chlorobenzyl)phenyl)ethan-1-one (2)[7]**:** Yellow oil was obtained with 71% isolated yield following the general procedure B (0.3 mmol scale, 52.1 mg, average yield of two times).

^{1}H NMR (400 MHz, Chloroform-*d*) δ 7.89 – 7.86 (m, 2H), 7.26 – 7.23 (m, 4H), 7.09 (d, *J* = 8.4 Hz, 2H), 3.98 (s, 2H), 2.56 (s, 3H).

^{13}C NMR (101 MHz, Chloroform-*d*) δ 197.8, 146.2, 138.6, 135.4, 132.3, 130.3, 129.1, 128.7(8), 128.7(7), 41.2, 26.6.

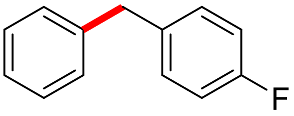

1-Benzyl-4-fluorobenzene (3)[8]**:** Yellow oil was obtained with 77% isolated yield following the general procedure B (0.3 mmol scale, 43.0 mg, average yield of two times).

^{1}H NMR (400 MHz, Chloroform-*d*) δ 7.30 – 7.26 (m, 2H), 7.20 (t, *J* = 7.6 Hz, 1H), 7.16 – 7.10 (m, 4H), 6.99 – 6.93 (m, 2H), 3.94 (s, 2H).

^{13}C NMR (101 MHz, Chloroform-*d*) δ 161.5 (d, J = 245.0 Hz), 141.1, 136.9 (d, J = 3.2, Hz), 130.4 (d, J = 7.8 Hz), 129.0, 128.7, 126.3, 115.3 (d, J = 21.2 Hz), 41.2.

^{19}F NMR (376 MHz, Chloroform-*d*): δ -117.21(m).

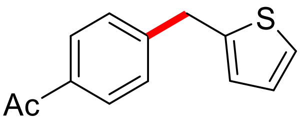

1-(4-(Thiophen-2-ylmethyl)phenyl)ethan-1-one (4) : White solid was obtained with 72% isolated yield following the general procedure B (0.3 mmol scale, 46.7 mg, average yield of two times).

^{1}H NMR (400 MHz, Chloroform-*d*) δ 7.91 – 7.87 (m, 2H), 7.32 – 7.30 (m, 2H), 7.15 – 7.13 (m, 1H), 6.93 – 6.91 (m, 1H), 6.80 – 6.79 (m, 1H), 4.18 (s, 2H), 2.55 (s, 3H).

^{13}C NMR (101 MHz, Chloroform-*d*) δ 197.7, 145.9, 142.6, 135.5, 128.8, 128.7, 127.0, 125.6, 124.3, 35.9, 26.6.

HRMS (ESI) m/z calcd. for $C_{13}H_{13}SO$ ($[M+H]^+$): 217.0682, found: 217.0681.

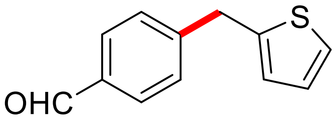

4-(Thiophen-2-ylmethyl)benzaldehyde (5): White solid was obtained with 62% isolated yield following the general procedure B (0.3 mmol scale, 37.6 mg, average yield of three times).

^{1}H NMR (600 MHz, Chloroform-d) δ 9.98 (s, 1H), 7.83 – 7.81 (m, 2H), 7.41 (d, J = 8.0 Hz, 2H), 7.18 (dd, J = 5.2, 1.2 Hz, 1H), 6.95 (dd, J = 5.2, 3.4 Hz, 1H), 6.84 – 6.83 (m, 1H), 4.23 (s, 2H).

^{13}C NMR (151 MHz, Chloroform-d) δ 192.0, 147.5, 142.3, 135.0, 130.2, 129.3, 127.1, 125.8, 124.5, 36.2.

HRMS (ESI) m/z calcd. for $C_{12}H_{11}SO$ ($[M+H]^+$): 203.0525, found: 203.0524.

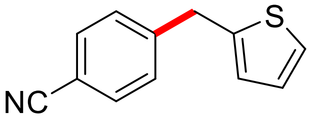

4-(Thiophen-2-ylmethyl)benzonitrile (6)[9]: White solid was obtained with 65% isolated yield following the general procedure B (0.3 mmol scale, 38.9 mg, average yield of three times).

^{1}H NMR (600 MHz, Chloroform-d) δ 7.58 (d, *J* = 8.2 Hz, 2H), 7.34 (d, *J* = 8.0 Hz, 2H), 7.19 (dd, *J* = 5.2, 1.2 Hz, 1H), 6.96 (dd, *J* = 5.2, 3.4 Hz, 1H), 6.83 (d, *J* = 3.4 Hz, 1H), 4.21 (s, 2H).

^{13}C NMR (151 MHz, Chloroform-d) δ 145.9, 141.7, 132.4, 129.3, 127.1, 125.9, 124.7, 118.9, 110.4, 36.0.

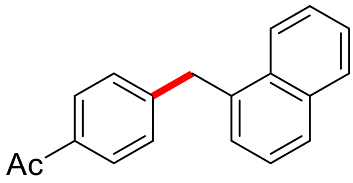

1-(4-(Naphthalen-1-ylmethyl)phenyl)ethan-1-one (7) : White solid was obtained with 70% isolated yield following the general procedure B (0.3 mmol scale, 54.7 mg, average yield of two times).

^{1}H NMR (600 MHz, Chloroform-d) δ 7.82 (d, *J* = 8.2 Hz, 1H), 7.79 – 7.76 (m, 3H), 7.70 (d, *J* = 6.4 Hz, 1H), 7.39 – 7.34 (m, 3H), 7.22 – 7.16 (m, 3H), 4.41 (s, 2H), 2.46 (s, 3H).

^{13}C NMR (151 MHz, Chloroform-d) δ 197.9, 146.6, 135.7, 135.4, 134.1, 132.1, 129.0, 128.9, 128.8, 127.6(7), 127.6(6), 126.3, 125.8, 125.7, 124.2, 39.3, 26.7.

HRMS (ESI) m/z calcd. for $C_{19}H_{17}O$ ([M+H]$^+$): 261.1274, found: 261.1275.

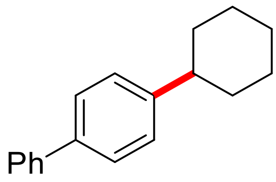

4-Cyclohexyl-1,1'-biphenyl (8)[12]: White solid was obtained with 51% isolated yield following the general procedure B (0.3 mmol scale, 36.2 mg, average yield of three times).

^{1}H NMR (600 MHz, Chloroform-d) δ 7.61 – 7.59 (m, 2H), 7.54 (d, *J* = 8.2 Hz, 2H), 7.45 – 7.42 (m, 2H), 7.35 – 7.32 (m, 1H), 7.30 (d, *J* = 8.2 Hz, 2H), 2.59 – 2.54 (m, 1H), 1.95 – 1.87 (m, 4H), 1.80 – 1.77 (m, 1H), 1.52 – 1.40 (m, 4H), 1.33 – 1.28 (m, 1H).

^{13}C NMR (151 MHz, Chloroform-d) δ 147.4, 141.3, 138.9, 128.8, 127.4, 127.1(7), 127.1(5), 127.0, 44.4, 34.6, 27.1, 26.3.

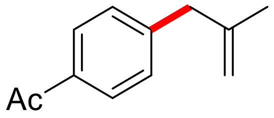

1-(4-(2-Methylallyl)phenyl)ethan-1-one (9)[10] : Yellow oil was obtained with 69% isolated yield following the general procedure B (0.3 mmol scale, 36.1 mg, average yield of three times).

^{1}H NMR (600 MHz, Chloroform-d) δ 7.90 – 7.88 (m, 2H), 7.29 – 7.27 (m, 2H), 4.85 – 4.84 (m, 1H), 4.74 – 4.73 (m, 1H), 3.37 (s, 2H), 2.59 (s, 3H), 1.67 (s, 3H).

^{13}C NMR (151 MHz, Chloroform-d) δ 198.0, 145.7, 144.2, 135.4, 129.2, 128.6, 112.8, 45.0, 26.7, 22.2.

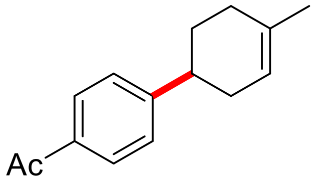

1-(4'-Methyl-1',2',3',6'-tetrahydro-[1,1'-biphenyl]-4-yl)ethan-1-one (10): Colorless oil was obtained with 56% isolated yield following the general procedure B (0.3 mmol scale, 36.0 mg, average yield of three times).

^{1}H NMR (400 MHz, Chloroform-d) δ 7.92 – 7.88 (m, 2H), 7.33 – 7.30 (m, 2H), 5.49 – 5.46 (m, 1H), 2.85 – 2.77 (m, 1H), 2.58 (s, 3H), 2.29 – 2.22 (m, 1H), 2.19 – 2.08 (m, 2H), 2.04 – 1.99 (m, 1H), 1.96 – 1.91 (m, 1H), 1.84 – 1.73 (m, 1H), 1.70 (s, 3H).

^{13}C NMR (101 MHz, Chloroform-d) δ 198.0, 153.2, 135.3, 134.2, 128.7, 127.3, 120.5, 40.2, 33.3, 30.5, 29.9, 26.7, 23.6.

HRMS (ESI) m/z calcd. for $C_{15}H_{19}O$ ($[M+H]^{+}$): 215.1430, found: 215.1431.

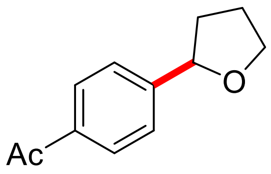

1-(4-((4-Methylcyclohex-3-en-1-yl)methyl)phenyl)ethan-1-one (11)[11]: Yellow oil was obtained with 52% isolated yield following the general procedure B (0.3 mmol scale, 29.7 mg, average yield of three times).

^{1}H NMR (600 MHz, Chloroform-d) δ 7.91 – 7.89 (m, 2H), 7.43 – 7.41 (d, *J* = 8.0 Hz, 2H), 4.94 (t, *J* = 7.2 Hz, 1H), 4.10 – 4.07 (m, 1H), 3.96 – 3.92 (m, 1H), 2.57 (s, 3H), 2.37 – 2.32 (m, 1H), 2.01 – 1.96 (m, 2H), 1.78 – 1.72 (m, 1H).

^{13}C NMR (151 MHz, Chloroform-d) δ 197.9, 149.3, 136.1, 128.5, 125.7, 80.2, 68.9, 34.8, 26.7, 26.0.

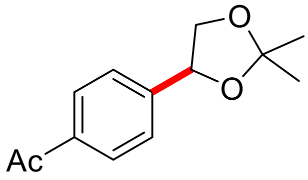

1-(4-(2,2-Dimethyl-1,3-dioxolan-4-yl)phenyl)ethan-1-one (12): Yellow oil was obtained with 52% isolated yield following the general procedure B (0.3 mmol scale, 34.4 mg, average yield of three times).

^{1}H NMR (600 MHz, Chloroform-d) δ 7.97 – 7.95 (m, 2H), 7.46 (d, *J* = 8.2 Hz, 2H), 5.13 (t, *J* = 6.4 Hz, 1H), 4.35 (dd, *J* = 8.2, 6.4 Hz, 1H), 3.69 (t, *J* = 8.0 Hz, 1H), 2.60 (s, 3H), 1.56 (s, 3H), 1.50 (s, 3H).

^{13}C NMR (151 MHz, Chloroform-d) δ 198.6, 144.9, 137.3, 128.8, 126.3, 110.3, 77.5, 71.6, 26.8, 26.6, 26.0.

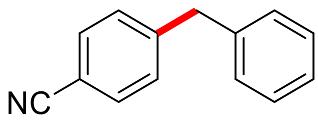

4-Benzylbenzonitrile (13)[6]: Yellow oil was obtained with 88% isolated yield following the general procedure B (0.3 mmol scale, 51.0 mg, average yield of two times).

^{1}H NMR (400 MHz, Chloroform-d) δ 7.55 – 7.52 (m, 2H), 7.32 – 7.21 (m, 5H), 7.15 (d, *J* = 7.0 Hz, 2H), 4.01 (s, 2H).

^{13}C NMR (151 MHz, Chloroform-d) δ 146.9, 139.5, 132.4, 129.8, 129.1, 128.9, 126.8, 119.1, 110.2, 42.1.

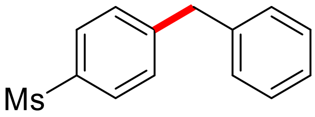

1-Benzyl-4-(methylsulfonyl)benzene (14)[8]: White solid was obtained with 79% isolated yield following the general procedure B (0.3 mmol scale, 58.4 mg, average yield of two times).

^{1}H NMR (400 MHz, Chloroform-d) δ 7.85 – 7.82 (m, 2H), 7.36 (d, *J* = 8.4 Hz, 2H), 7.32 – 7.28 (m, 2H), 7.24 – 7.16 (m, 3H), 4.04 (s, 2H), 3.00 (s, 3H).

^{13}C NMR (101 MHz, Chloroform-d) δ 147.7, 139.5, 138.3, 129.8, 129.0, 128.8, 127.6, 126.6, 44.5, 41.8.

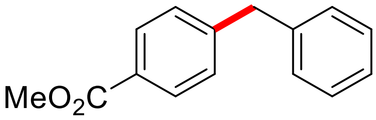

Methyl 4-benzylbenzoate (15)[6]: Yellow oil was obtained with 71% isolated yield following the general procedure B (0.3 mmol scale, 48.2 mg, average yield of two times).

^{1}H NMR (600 MHz, Chloroform-d) δ 7.95 (d, *J* = 8.2 Hz, 2H), 7.29 – 7.27 (m, 2H), 7.23 (d, *J* = 8.0 Hz, 2H), 7.21 – 7.18 (m, 1H), 7.15 (d, *J* = 7.0 Hz, 2H), 4.00 (s, 2H), 3.87 (s, 3H).

^{13}C NMR (151 MHz, Chloroform-d) δ 167.1, 146.6, 140.2, 129.9, 129.0, 128.7, 128.2, 126.5, 52.1, 42.0 *One peak is missing due to overlap*.

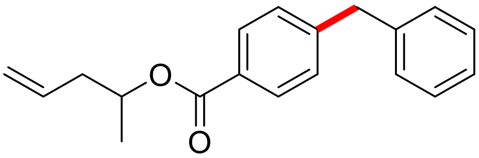

Pent-4-en-2-yl 4-benzylbenzoate (16): Colorless oil was obtained with 68% isolated

yield following the general procedure B (0.3 mmol scale, 57.2 mg, average yield of two times).

^{1}H NMR (600 MHz, Chloroform-d) δ 7.87 (d, *J* = 8.4 Hz, 2H), 7.21 – 7.18 (m, 2H), 7.16 (d, *J* = 8.2 Hz, 2H), 7.13 – 7.10 (m, 1H), 7.08 (d, *J* = 6.6 Hz, 2H), 5.77 – 5.70 (m, 1H), 5.13 – 5.08 (m, 1H), 5.05 – 4.98 (m, 2H), 3.92 (s, 2H), 2.40 – 2.35 (m, 1H), 2.33 – 2.28 (m, 1H), 1.24 (d, *J* = 6.2 Hz, 3H).

^{13}C NMR (151 MHz, Chloroform-d) δ 166.1, 146.5, 140.3, 133.8, 129.9, 129.0, 128.9, 128.8, 128.7, 126.5, 117.9, 70.7, 42.0, 40.5, 19.7.

HRMS (ESI) m/z calcd. for $C_{19}H_{21}O_2$ ($[M+H]^+$): 281.1536, found: 281.1546.

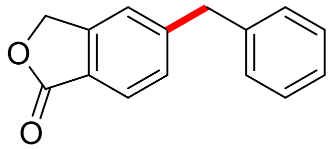

5-Benzylisobenzofuran-1(3H)-one (17)[9]**:** White solid was obtained with 82% isolated yield following the general procedure B (0.3 mmol scale, 55.2 mg, average yield of two times).

^{1}H NMR (600 MHz, Chloroform-d) δ 7.78 (s, 1H), 7.35 – 7.17 (m, 7H), 5.20 (s, 2H), 4.08 (s, 2H).

^{13}C NMR (151 MHz, Chloroform-d) δ 171.0, 148.4, 147.2, 139.6, 130.0, 128.9, 128.7, 126.6, 125.6, 123.7, 122.3, 69.5, 42.1.

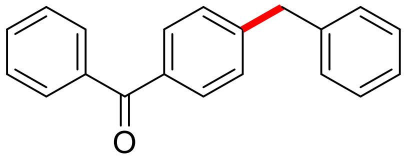

5-Benzylisobenzofuran-1(3H)-one (18)[13]**:** White solid was obtained with 80% isolated yield following the general procedure B (0.3 mmol scale, 65.4 mg, average yield of two times).

^{1}H NMR (600 MHz, Chloroform-d) δ 7.77 (d, *J* = 8.2 Hz, 2H), 7.73 (d, *J* = 6.4 Hz, 2H), 7.56 – 7.53 (m, 1H), 7.45 – 7.43 (m, 2H), 7.31 – 7.28 (m, 4H), 7.23 – 7.19 (m, 3H), 4.04 (s, 2H).

^{13}C NMR (151 MHz, Chloroform-d) δ 196.5, 146.3, 140.2, 137.9, 135.6, 132.3, 130.6, 130.1, 129.1, 128.9, 128.7, 128.3, 126.5, 42.0.

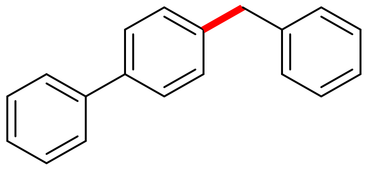

4-Benzyl-1,1'-biphenyl (19)[14]: White solid was obtained with 51% isolated yield following the general procedure B (0.3 mmol scale, 37.4 mg, average yield of two times).

^{1}H NMR (600 MHz, Chloroform-d) δ 7.54 (d, *J* = 7.0 Hz, 2H), 7.48 (d, *J* = 8.2 Hz, 2H), 7.38 – 7.36 (m, 2H), 7.29 – 7.25 (m, 3H), 7.22 – 7.16 (m, 5H), 3.98 (s, 2H).

^{13}C NMR (151 MHz, Chloroform-d) δ 141.1(1), 141.0(8), 140.4, 139.1, 129.4, 129.1, 128.8, 128.6, 127.3, 127.2, 127.1, 126.3, 41.7.

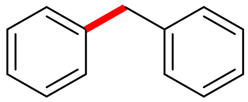

Diphenylmethane (20)[8]: Colorless oil was obtained with 68% isolated yield following the general procedure B (0.3 mmol scale, 34.3 mg, average yield of three times).

^{1}H NMR (600 MHz, Chloroform-d) δ 7.27 – 7.23 (m, 4H), 7.18 – 7.15 (m, 6H), 3.95 (s, 2H).

^{13}C NMR (151 MHz, Chloroform-d) δ 141.2, 129.0, 128.6, 126.2, 42.1.

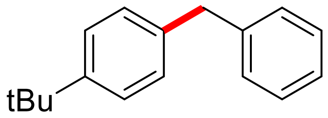

1-Benzyl-4-(tert-butyl)benzene (21)[15]: Colorless oil was obtained with 58% isolated yield following the general procedure B (0.3 mmol scale, 39.0 mg, average yield of three times).

^{1}H NMR (600 MHz, Chloroform-d) δ 7.31 – 7.25 (m, 4H), 7.20 – 7.16 (m, 3H), 7.12 – 7.10 (m, 2H), 3.94 (s, 2H), 1.29 (s, 9H).

^{13}C NMR (151 MHz, Chloroform-d) δ 149.0, 141.4, 138.2, 129.1, 128.6(4), 128.5(6), 126.1, 125.5, 41.6, 34.5, 31.5.

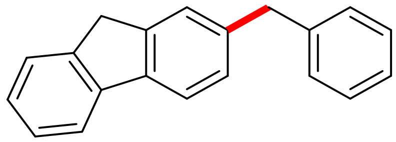

2-Benzyl-9H-fluorene (22)[6]: White solid was obtained with 84% isolated yield following the general procedure B (0.3 mmol scale, 64.6 mg, average yield of two times).

^{1}H NMR (600 MHz, Chloroform-d) δ 7.83 (d, *J* = 7.6 Hz, 1H), 7.78 (d, *J* = 7.8 Hz, 1H), 7.58 (d, *J* = 7.4 Hz, 1H), 7.46 – 7.43 (m, 2H), 7.41 – 7.39 (m, 2H), 7.38 – 7.35 (m, 1H), 7.34 – 7.30 (m, 4H), 4.14 (s, 2H), 3.91 (s, 2H).

^{13}C NMR (151 MHz, Chloroform-d) δ 143.8, 143.3, 141.7, 141.5, 139.9(2), 139.9(1), 129.1, 128.6, 127.7, 126.9, 126.5, 126.2, 125.7, 125.1, 119.9, 119.8, 42.2, 36.9.

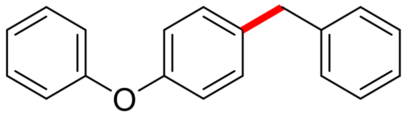

1-Benzyl-4-phenoxybenzene (23)[14]: Colorless oil was obtained with 45% isolated yield following the general procedure B (0.3 mmol scale, 35.1 mg, average yield of three times).

^{1}H NMR (400 MHz, Chloroform-d) δ 7.31 – 7.26 (m, 4H), 7.21 -7.17 (m, 3H), 7.14 – 7.11 (m, 2H), 7.07 – 7.03 (m, 1H), 6.99 – 6.96 (m, 2H), 6.94 – 6.90 (m, 2H), 3.94 (s, 2H).

^{13}C NMR (101 MHz, Chloroform-d) δ 157.6, 155.5, 140.5, 136.2, 130.3, 129.8, 129.0, 128.6, 126.3, 123.1, 119.2, 118.7, 41.3.

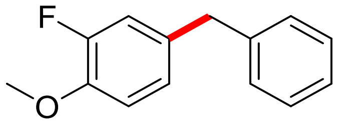

4-Benzyl-2-fluoro-1-methoxybenzene (24)[24]**:** Colorless oil was obtained with 51% isolated yield following the general procedure B (0.3 mmol scale, 33.1mg, average yield of three times).

^{1}H NMR (600 MHz, Chloroform-d) δ 7.29 – 7.26 (m, 2H), 7.20 – 7.17 (m, 1H), 7.15 (d, J = 6.8 Hz, 2H), 6.89 – 6.83 (m, 3H), 3.88 (s, 2H), 3.82 (s, 3H).

^{13}C NMR (101 MHz, Chloroform-d) δ 152.4 (d, J = 246.8 Hz), 146.0 (d, J = 10.8 Hz), 140.9, 134.4 (d, J = 6.0 Hz), 128.9, 128.6, 126.3, 124.4 (d, J = 3.4 Hz), 116.7 (d, J = 18.2 Hz), 113.5 (d, J = 2.2 Hz), 56.4, 41.0.

^{19}F NMR (565 MHz, Chloroform-*d*): δ -135.29 (m).

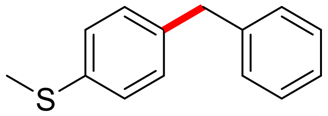

(4-Benzylphenyl)(methyl)sulfane (25)[16]**:** White solid was obtained with 52% isolated yield following the general procedure B (0.3 mmol scale, 33.4 mg, average yield of three times).

^{1}H NMR (400 MHz, Chloroform-d) δ 7.29 – 7.25 (m, 2H), 7.22 – 7.16 (m, 5H), 7.11 – 7.09 (m, 2H), 3.93 (s, 2H), 2.44 (s, 3H).

^{13}C NMR (101 MHz, Chloroform-d) δ 141.1, 138.3, 135.9, 129.6, 129.0, 128.6, 127.2, 126.2, 41.5, 16.3.

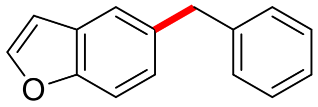

6-Benzylbenzofuran (26)[17]**:** Yellow oil was obtained with 65% isolated yield following the general procedure B (from bromide, 0.3 mmol scale, 40.6 mg, average yield of two times).

^{1}H NMR (600 MHz, Chloroform-d) δ 7.54 (d, *J* = 2.2 Hz, 1H), 7.40 – 7.36 (m, 2H), 7.27 – 7.25 (m, 2H), 7.19 – 7.15 (m, 3H), 7.10 (dd, *J* = 8.4 Hz, *J* = 1.8 Hz, 1H), 6.65 (d, *J* = 2.2 Hz, 1H), 4.04 (s, 2H).

^{13}C NMR (151 MHz, Chloroform-d) δ 153.8, 145.3, 141.8, 135.8, 129.0, 128.6, 127.7, 126.2, 125.6, 121.2, 111.3, 106.6, 41.9.

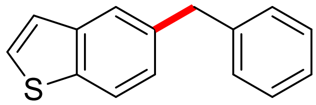

5-Benzylbenzo[b]thiophene (27)[18]: Yellow oil was obtained with 62% isolated yield following the general procedure B (0.3 mmol scale, 41.7 mg, average yield of two times).

^{1}H NMR (400 MHz, Chloroform-d) δ 7.74 (d, *J* = 8.2 Hz, 1H), 7.59 (s, 1H), 7.34 (d, *J* = 5.4 Hz, 1H), 7.28 – 7.24 (m, 2H), 7.21 – 7.13 (m, 5H), 4.06 (s, 2H).

^{13}C NMR (101 MHz, Chloroform-d) δ 141.4, 140.1, 137.7, 137.4, 129.1, 128.6, 126.7, 126.2, 125.9, 123.8, 123.7, 122.5, 41.9.

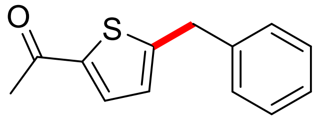

1-(5-Benzylthiophen-2-yl)ethan-1-one (28)[17]: White solid was obtained with 78% isolated yield following the general procedure B (0.3 mmol scale, 50.6 mg, average yield of two times).

^{1}H NMR (600 MHz, Chloroform-d) δ 7.52 (d, *J* = 3.8 Hz, 1H), 7.33 – 7.30 (m, 2H), 7.26 – 7.23 (m, 3H), 6.81 (d, *J* = 3.8 Hz, 1H), 4.14 (s, 2H), 2.48 (s, 3H).

^{13}C NMR (151 MHz, Chloroform-d) δ 190.6, 154.1, 143.0, 139.1, 132.4, 128.9, 128.8, 127.0, 126.5, 36.8, 26.6.

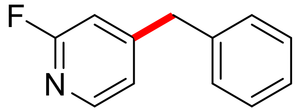

4-Benzyl-2-fluoropyridine (29)[19]: Colorless oil was obtained with 91% isolated yield

following the general procedure B (0.3 mmol scale, 51.1 mg, average yield of two times).

^{1}H NMR (600 MHz, Chloroform-d) δ 8.08 (d, *J* = 5.2 Hz, 1H), 7.33 – 7.30 (m, 2H), 7.26 – 7.24 (m, 1H), 7.16 (d, *J* = 7.0 Hz, 2H), 6.98 (d, *J* = 5.2 Hz, 1H), 6.70 (s, 1H), 3.97 (s, 2H).

^{13}C NMR (151 MHz, Chloroform-d) δ 164.4 (d, *J* = 238.6 Hz), 156.1 (d, *J* = 7.8 Hz), 147.5 (d, *J* = 15.2 Hz), 138.1, 129.1, 128.9, 127.0, 121.9 (d, *J* = 3.8 Hz), 109.5 (d, *J* = 37.2 Hz), 41.1 (d, *J* = 2.8 Hz).

^{19}F NMR (565 MHz, Chloroform-d): δ -68.61.

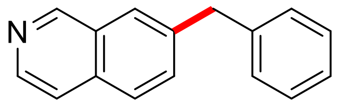

7-Benzylisoquinoline (30)[15]: Colorless oil was obtained with 84% isolated yield following the general procedure B (0.3 mmol scale, 55.3 mg, average yield of two times).

^{1}H NMR (600 MHz, Chloroform-d) δ 9.18 (s, 1H), 8.47 (d, *J* = 5.8 Hz, 1H), 7.84 (d, *J* = 8.4 Hz, 1H), 7.57 (s, 1H), 7.54 (d, *J* = 5.8 Hz, 1H), 7.42 (dd, *J* = 8.4, 1.6 Hz, 1H), 7.31 – 7.29 (m, 2H), 7.24 – 7.20 (m, 3H), 4.14 (s, 2H).

^{13}C NMR (151 MHz, Chloroform-d) δ 152.2, 143.8, 143.3, 140.0, 136.1, 129.1(2), 129.0(9), 128.7, 127.8, 127.5, 126.6, 125.6, 120.3, 41.9.

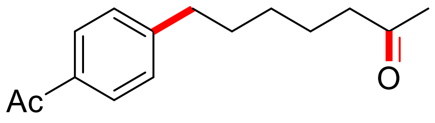

7-(4-Acetylphenyl)heptan-2-one (31): Yellow oil was obtained with 90% isolated yield following the general procedure C (0.3 mmol scale, 62.7 mg, average yield of two times).

^{1}H NMR (400 MHz, Chloroform-d) δ 7.87 (d, *J* = 8.4 Hz, 2H), 7.25 (d, *J* = 8.2 Hz, 2H), 2.65 (t, *J* = 7.6 Hz, 2H), 2.56 (s, 3H), 2.42 (t, *J* = 7.4 Hz, 2H), 2.12 (s, 3H), 1.67 – 1.56 (m, 4H), 1.36 – 1.28 (m, 2H).

^{13}C NMR (101 MHz, Chloroform-d) δ 208.8, 197.6, 148.2, 134.7, 128.4, 128.3, 43.3, 35.5, 30.7, 29.7, 28.5, 26.4, 23.3.

HRMS (ESI) m/z calcd. for $C_{15}H_{21}O_2$ ([M+H]$^+$): 233.1536, found: 233.1533.

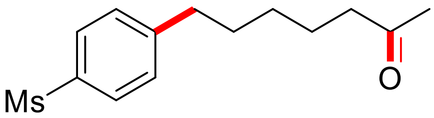

7-(4-(Methylsulfonyl)phenyl)heptan-2-one (32): White solid was obtained with 81% isolated yield following the general procedure C (0.3 mmol scale, 65.2 mg, average yield of two times).

^{1}H NMR (600 MHz, Chloroform-d) δ 7.78 (d, *J* = 8.2 Hz, 2H), 7.30 (d, *J* = 8.4 Hz, 2H), 2.99 (s, 3H), 2.64 (t, *J* = 7.6 Hz, 2H), 2.37 (t, *J* = 7.4 Hz, 2H), 2.07 (s, 3H), 1.61 – 1.52 (m, 4H), 1.30 – 1.24 (m, 2H).

^{13}C NMR (151 MHz, Chloroform-d) δ 209.0, 149.2, 137.9, 129.3, 127.4, 44.5, 43.4, 35.6, 30.8, 29.9, 28.6, 23.4.

HRMS (ESI) m/z calcd. for $C_{14}H_{21}O_3S$ ([M+H]$^+$): 269.1206, found: 269.1207.

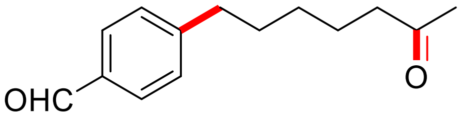

4-(6-Oxoheptyl)benzaldehyde (33): White solid was obtained with 49% isolated yield following the general procedure C (0.3 mmol scale, 32.1 mg, average yield of three times).

^{1}H NMR (400 MHz, Chloroform-d) δ 9.96 (s, 1H), 7.79 (d, *J* = 8.2 Hz, 2H), 7.32 (d, *J* = 8.0 Hz, 2H), 2.68 (t, *J* = 7.6 Hz, 2H), 2.42 (t, *J* = 7.4 Hz, 2H), 2.12 (s, 3H), 1.67 – 1.58 (m, 4H), 1.36 – 1.31 (m, 2H).

^{13}C NMR (101 MHz, Chloroform-d) δ 209.2, 192.2, 150.7, 135.2, 130.1, 129.2, 43.7, 36.1, 31.0, 30.1, 28.8, 23.6.

HRMS (ESI) m/z calcd. for $C_{14}H_{19}O_2$ ([M+H]$^+$): 219.1380, found: 219.1381.

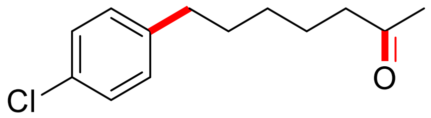

7-(4-Chlorophenyl)heptan-2-one (34): Colorless oil was obtained with 58% isolated yield following the general procedure C (0.3 mmol scale, 39.1 mg, average yield of three times).

^{1}H NMR (600 MHz, Chloroform-d) δ 7.23 – 7.21 (m, 2H), 7.09 – 7.07 (m, 2H), 2.56 (t, J = 7.6 Hz, 2H), 2.40 (t, J = 7.4 Hz, 2H), 2.11 (s, 3H), 1.61 – 1.56 (m, 4H), 1.32 – 1.28 (m, 2H).

^{13}C NMR (101 MHz, Chloroform-d) δ 209.1, 142.5, 128.3, 128.2, 125.6, 43.6, 35.7, 31.2, 29.8, 28.7, 23.6.

HRMS (ESI) m/z calcd. for $C_{13}H_{18}ClO$ ($[M+H]^+$): 225.1041, found: 225.1047.

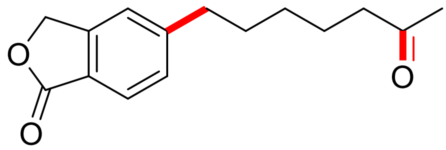

5-(6-Oxoheptyl)isobenzofuran-1(3H)-one (35): White solid was obtained with 63% isolated yield following the general procedure C (0.3 mmol scale, 46.6 mg, average yield of two times).

^{1}H NMR (600 MHz, Chloroform-d) δ 7.80 (d, J = 7.8 Hz, 1H), 7.34 (d, J = 7.8 Hz, 1H), 7.32 (s, 1H), 5.29 (s, 2H), 2.75 (t, J = 7.6 Hz, 2H), 2.45 (t, J = 7.2 Hz, 2H), 2.14 (s, 3H), 1.70 – 1.65 (m, 2H), 1.64 – 1.59 (m, 2H), 1.38 – 1.32 (m, 2H).

^{13}C NMR (151 MHz, Chloroform-d) δ 208.9, 171.1, 149.8, 147.1, 129.5, 125.4, 123.3, 121.8, 69.5, 43.4, 36.0, 31.0, 29.9, 28.6, 23.4.

HRMS (ESI) m/z calcd. for $C_{15}H_{19}O_3$ ($[M+H]^+$): 247.1329, found: 247.1326.

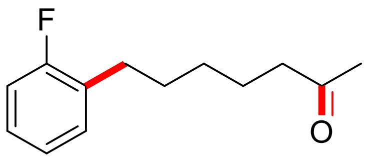

7-(2-Fluorophenyl)heptan-2-one (36): Colorless oil was obtained with 70% isolated yield following the general procedure C (0.3 mmol scale, 43.7 mg, average yield of two

times).

^{1}H NMR (600 MHz, Chloroform-d) δ 7.14 – 7.10 (m, 2H), 7.02 – 7.00 (m, 1H), 6.97 – 6.94 (m, 1H), 2.60 (t, *J* = 7.8 Hz, 2H), 2.38 (t, *J* = 7.4 Hz, 2H), 2.08 (s, 3H), 1.61 – 1.55 (m, 4H), 1.34 – 1.29 (m, 2H).

^{13}C NMR (101 MHz, Chloroform-d) δ 209.1, 161.1 (d, *J* = 245.0 Hz), 130.6 (d, *J* = 5.2 Hz), 129.2 (d, *J* = 16.0 Hz), 127.4 (d, *J* = 8.0 Hz), 123.9, 115.1 (d, *J* = 22.4 Hz), 43.6, 29.9 (d, *J* = 10.4 Hz), 28.8, 23.5 *Two peaks are missing due to overlap.*

^{19}F NMR (376 MHz, Chloroform-d): δ -119.02 (m).

HRMS (ESI) m/z calcd. for $C_{13}H_{18}FO$ ([M+H]$^+$): 209.1336, found: 209.1338.

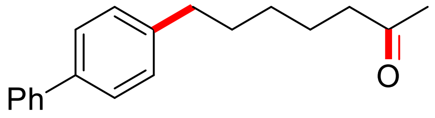

7-([1,1'-Biphenyl]-4-yl)heptan-2-one (37): White solid was obtained with 62% isolated yield following the general procedure C (0.3 mmol scale, 49.5 mg, average yield of two times).

^{1}H NMR (600 MHz, Chloroform-d) δ 7.57 (d, *J* = 6.6 Hz, 2H), 7.50 (d, *J* = 8.0 Hz, 2H), 7.42 – 7.40 (m, 2H), 7.32 – 7.29 (m, 1H), 7.23 (d, *J* = 7.8 Hz, 2H), 2.63 (t, *J* = 7.6 Hz, 2H), 2.41 (t, *J* = 7.4 Hz, 2H), 2.11 (s, 3H), 1.68 – 1.58 (m, 4H), 1.37 – 1.32 (m, 2H).

^{13}C NMR (151 MHz, Chloroform-d) δ 209.3, 141.8, 141.2, 138.7, 128.9, 128.8, 127.0(9), 127.0(7), 127.0(5), 43.7, 35.4, 31.3, 30.0, 28.9, 23.7.

HRMS (ESI) m/z calcd. for $C_{19}H_{23}O$ ([M+H]$^+$): 267.1743, found: 267.1741.

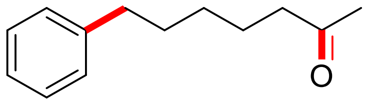

7-Phenylheptan-2-one (38)[20]**:** Colorless oil was obtained with 60% isolated yield following the general procedure C (0.3 mmol scale, 34.3 mg, average yield of three times).

^{1}H NMR (400 MHz, Chloroform-d) δ 7.28 – 7.22 (m, 2H), 7.17 – 7.13 (m, 3H), 2.59 (t, J = 7.6 Hz, 2H), 2.38 (t, J = 7.4 Hz, 2H), 2.09 (s, 3H), 1.65 – 1.54 (m, 4H), 1.35 – 1.27 (m, 2H).

^{13}C NMR (101 MHz, Chloroform-d) δ 209.1, 142.5, 128.3, 128.2, 125.6, 43.6, 35.7, 31.2, 29.8, 28.7, 23.6.

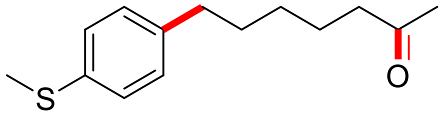

7-(4-(Methylthio)phenyl)heptan-2-one (39): Colorless oil was obtained with 64% isolated yield following the general procedure C (0.3 mmol scale, 45.4 mg, average yield of two times).

^{1}H NMR (400 MHz, Chloroform-d) δ 7.20 – 7.17 (m, 2H), 7.10 – 7.07 (m, 2H), 2.57 – 2.54 (m, 2H), 2.45 (s, 3H), 2.42 – 2.39 (m, 2H), 2.11 (s, 3H), 1.63 – 1.55 (m, 4H), 1.35 – 1.29 (m, 2H).

^{13}C NMR (101 MHz, Chloroform-d) δ 209.2, 139.8, 135.2, 129.0, 127.2, 43.7, 35.2, 31.3, 30.0, 28.8, 23.2, 16.4.

HRMS (ESI) m/z calcd. for $C_{14}H_{21}OS$ ([M+H]$^+$): 237.1308, found: 237.1310.

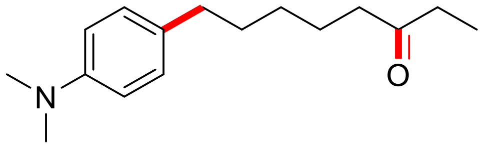

8-(4-(Dimethylamino)phenyl)octan-3-one (40): Colorless oil was obtained with 54% isolated yield (Procedure C, 5.0 equiv. alcohol, 4 mA, 16 h. 0.3 mmol scale, 40.0 mg, average yield of two times).

^{1}H NMR (600 MHz, Chloroform-d) δ 7.06 (d, J = 8.8 Hz, 2H), 6.68 (d, J = 6.6 Hz, 2H), 3.50 (t, J = 7.8 Hz, 1H), 2.93 (s, 6H), 2.46 – 2.39 (m, 1H), 2.35 – 2.30 (m, 1H), 2.01 – 1.94 (m, 1H), 1.68 – 1.61 (m, 2H), 1.34 – 1.28 (m, 2H), 1.18 – 1.11 (m, 2H), 0.95 (t, J = 6.2 Hz, 3H), 0.86 – 0.83 (m, 2H).

^{13}C NMR (151 MHz, Chloroform-d) δ 212.2, 149.8, 129.0, 127.3, 112.9, 57.9, 40.7, 34.9, 31.9, 29.9, 22.8, 14.1, 8.1.

HRMS (ESI) m/z calcd. for $C_{16}H_{26}NO$ ([M+H]$^+$): 248.2009, found: 248.2001.

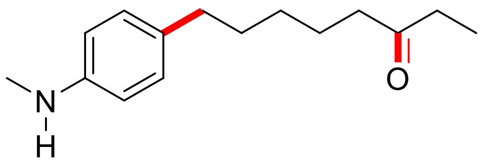

8-(4-(Methylamino)phenyl)octan-3-one (41): Colorless oil was obtained with 41% isolated yield (Procedure C, 5.0 equiv. alcohol, 4 mA, 3 h. 0.3 mmol scale, 28.7 mg, average yield of two times).

^{1}H NMR (400 MHz, Chloroform-d) δ 7.00 (d, J = 8.4 Hz, 2H), 6.56 (d, J = 8.4 Hz, 2H), 2.82 (s, 3H), 2.50 (t, J = 7.8 Hz, 2H), 2.43 – 2.37 (m, 4H), 1.61 – 1.56 (m, 4H), 1.35 – 1.29 (m, 2H), 1.05 (t, J = 7.2 Hz, 3H).

^{13}C NMR (151 MHz, Chloroform-d) δ 212.1, 147.5, 131.5, 129.2, 112.6, 42.5, 36.0, 34.9, 31.7, 31.1, 29.0, 23.9, 8.0.

HRMS (ESI) m/z calcd. for $C_{15}H_{24}ON$ ([M+H]$^+$): 234.1852, found: 234.1844.

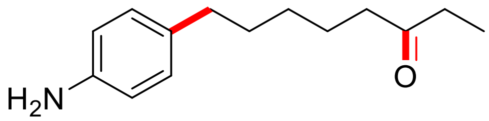

8-(4-Aminophenyl)octan-3-one (42): Yellow oil was obtained with 33% isolated yield (Procedure C, 5.0 equiv. alcohol, 4 mA, 3 h. 0.3 mmol scale, 21.7 mg, average yield of two times).

^{1}H NMR (400 MHz, Chloroform-d) δ 6.95 (d, J = 8.2 Hz, 2H), 6.62 (d, J = 8.2 Hz, 2H), 3.55 (br, 2H), 2.49 (t, J = 7.8 Hz, 2H), 2.41 – 2.36 (m, 4H), 1.61 – 1.54 (m, 4H), 1.34 – 1.28 (m, 2H), 1.04 (t, J = 7.2 Hz, 3H).

^{13}C NMR (151 MHz, Chloroform-d) δ 212.1, 144.2, 132.8, 129.2, 115.3, 42.5, 36.0, 34.9, 31.7, 28.9, 23.9, 8.0.

HRMS (ESI) m/z calcd. for $C_{14}H_{22}ON$ ([M+H]$^+$): 220.1696, found: 220.1697.

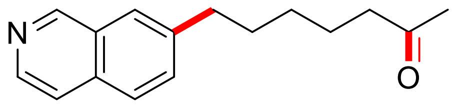

7-(Isoquinolin-7-yl)heptan-2-one (43): Colorless oil was obtained with 64% isolated

yield following the general procedure C (0.3 mmol scale, 46.3 mg, average yield of two times).

^{1}H NMR (600 MHz, Chloroform-d) δ 9.17 (s, 1H), 8.46 (d, *J* = 5.8 Hz, 1H), 7.86 (d, *J* = 8.2 Hz, 1H), 7.56 – 7.55 (m, 2H), 7.42 (dd, *J* = 8.2, 1.6 Hz, 1H), 2.78 (t, *J* = 7.6 Hz, 2H), 2.41 (t, *J* = 7.4 Hz, 2H), 2.10 (s, 3H), 1.73 – 1.68 (m, 2H), 1.63 – 1.58 (m, 2H), 1.37 – 1.32 (m, 2H).

^{13}C NMR (151 MHz, Chloroform-d) δ 209.1, 152.2, 145.3, 143.1, 136.2, 128.9, 127.6, 127.5, 124.9, 120.2, 43.6, 36.2, 30.9, 30.0, 28.8, 23.6.

HRMS (ESI) m/z calcd. for $C_{16}H_{20}NO$ ($[M+H]^+$): 242.1539, found: 242.1535.

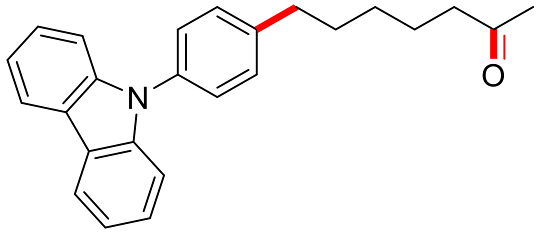

7-(4-(9H-carbazol-9-yl)phenyl)heptan-2-one (44): White solid was obtained with 49% isolated yield following the general procedure C (0.3 mmol scale, 52.3 mg, average yield of two times).

^{1}H NMR (600 MHz, Chloroform-d) δ 8.19 – 8.17 (m, 2H), 7.50 – 7.47 (m, 2H), 7.45 – 7.43 (m, 4H), 7.42 – 7.40 (m, 2H), 7.33 – 7.30 (m, 2H), 2.75 (t, *J* = 7.8 Hz, 2H), 2.49 (t, *J* = 7.4 Hz, 2H), 2.17 (s, 3H), 1.78-1.73 (m, 2H), 1.72 – 1.67 (m, 2H), 1.46 – 1.41 (m, 2H).

^{13}C NMR (151 MHz, Chloroform-d) δ 209.2, 142.0, 141.1, 135.3, 129.8, 127.0, 125.9, 123.3, 120.3, 119.8, 109.9, 43.3, 35.5, 31.3, 30.0, 28.9, 23.7.

HRMS (ESI) m/z calcd. for $C_{25}H_{26}NO$ ($[M+H]^+$): 356.2009, found: 356.2010.

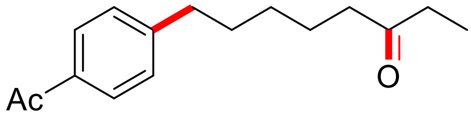

8-(4-Acetylphenyl)octan-3-one (45): Colorless oil was obtained with 80% isolated yield following the general procedure C (0.3 mmol scale, 59.1 mg, average yield of two

times).

^{1}H NMR (600 MHz, Chloroform-d) δ 7.86 (d, *J* = 8.2 Hz, 2H), 7.24 (d, *J* = 8.0 Hz, 2H), 2.65 (t, *J* = 7.6 Hz, 2H), 2.57 (s, 3H), 2.41 – 2.37 (m, 4H), 1.65-1.57 (m, 4H), 1.33 – 1.28 (m, 2H), 1.03 (t, *J* = 7.2 Hz, 3H).

^{13}C NMR (101 MHz, Chloroform-d) δ 211.8, 198.0, 148.5, 135.1, 128.7, 128.6, 43.0, 36.0, 35.8, 31.0, 28.9, 26.7, 23.7, 7.9.

HRMS (ESI) m/z calcd. for $C_{16}H_{23}O_2$ ($[M+H]^+$): 247.1693, found: 247.1684.

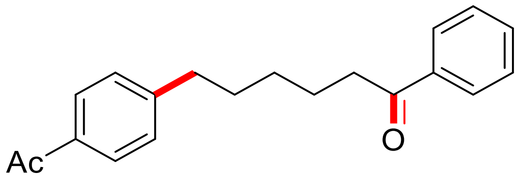

6-(4-Acetylphenyl)-1-phenylhexan-1-one (46): Colorless oil was obtained with 75% isolated yield following the general procedure C (0.3 mmol scale, 66.2 mg, average yield of two times).

^{1}H NMR (400 MHz, Chloroform-d) δ 7.95 (d, *J* = 8.4 Hz, 2H), 7.88 (d, *J* = 8.0 Hz, 2H), 7.58 – 7.54 (m, 1H), 7.48 – 7.44 (m, 2H), 7.27 – 7.25 (m, 2H), 2.97 (t, *J* = 7.2 Hz, 2H), 2.69 (t, *J* = 7.6 Hz, 2H), 2.58 (s, 3H), 1.82 – 1.74 (m, 2H), 1.71 – 1.66 (m, 2H), 1.47 – 1.39 (m, 2H).

^{13}C NMR (101 MHz, Chloroform-d) δ 200.4, 198.1, 148.6, 137.1, 135.1, 133.1, 128.8, 128.7, 128.6, 128.2, 38.5, 36.4, 30.7, 29.0, 26.2, 24.1.

HRMS (ESI) m/z calcd. for $C_{20}H_{23}O_2$ ($[M+H]^+$): 295.1693, found: 295.1688.

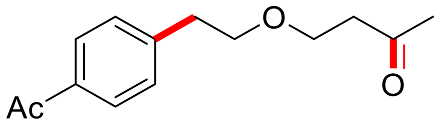

4-(4-Acetylphenethoxy)butan-2-one (47): Colorless oil was obtained with 72% isolated yield following the general procedure C (0.3 mmol scale, 50.6 mg, average yield of two times).

^{1}H NMR (400 MHz, Chloroform-d) δ 7.79 (d, *J* = 8.2 Hz, 2H), 7.22 (d, *J* = 8.0 Hz, 2H), 3.62 – 3.56 (m, 4H), 2.83 (t, *J* = 6.8 Hz, 2H), 2.57 (t, *J* = 6.2 Hz, 2H), 2.49 (s, 3H), 2.04 (s, 3H).

^{13}C NMR (101 MHz, Chloroform-d) δ 207.2, 197.7, 144.8, 135.2, 129.0, 128.3, 71.1, 65.7, 43.5, 36.0, 30.3, 26.4.

HRMS (ESI) m/z calcd. for $C_{14}H_{19}O_3$ ($[M+H]^+$): 235.1329, found: 235.1329.

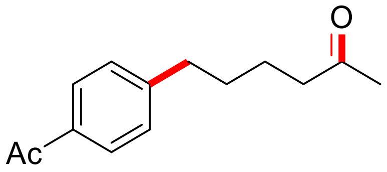

6-(4-Acetylphenyl)hexan-2-one (48)[21]: Yellow oil was obtained with 68% isolated yield following the general procedure C (0.3 mmol scale, 44.5 mg, average yield of two times).

^{1}H NMR (600 MHz, Chloroform-d) δ 7.87 (d, *J* = 8.2 Hz, 2H), 7.25 (d, *J* = 8.2 Hz, 2H), 2.67 (t, *J* = 7.0 Hz, 2H), 2.56 (s, 3H), 2.45 (t, *J* = 6.8 Hz, 2H), 2.11 (s, 3H), 1.65 – 1.59 (m, 4H).

^{13}C NMR (151 MHz, Chloroform-d) δ 208.5, 197.5, 147.9, 134.8, 128.4, 128.3, 43.2, 35.5, 30.3, 29.7, 26.4, 23.1.

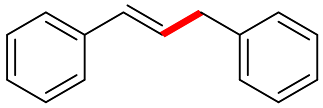

(*E*)-Prop-1-ene-1,3-diyldibenzene (49)[22]: Colorless oil was obtained with 76% isolated yield following the general procedure B (0.3 mmol scale, 44.3 mg, average yield of two times).

^{1}H NMR (600 MHz, Chloroform-d) δ 7.36 (d, *J* = 7.4 Hz, 2H), 7.32 – 7.27 (m, 4H), 7.25 – 7.19 (m, 4H), 6.46 (d, *J* = 15.6 Hz, 1H), 6.38 – 6.34 (m, 1H), 3.55 (d, *J* = 5.6 Hz, 2H).

^{13}C NMR (151 MHz, Chloroform-d) δ 140.3, 137.6, 131.2, 129.4, 128.8, 128.6(4), 128.6(3) 127.2, 126.3(2), 126.2(6), 39.5.

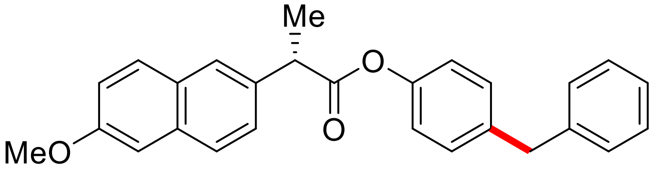

4-Benzylphenyl (R)-2-(6-methoxynaphthalen-2-yl)propanoate (50): White solid was obtained with 80% isolated yield following the general procedure B (0.3 mmol scale, 95.2 mg).

^{1}H NMR (600 MHz, Chloroform-d) δ 7.75 – 7.71 (m, 3H), 7.48 (dd, *J* = 8.4, 3.0 Hz, 1H), 7.26 – 7.23 (m, 2H), 7.19 – 7.10 (m, 7H), 6.90 – 6.88 (m, 2H), 4.07 (q, *J* = 7.0 Hz, 1H), 3.93 (s, 2H), 3.90 (s, 3H), 1.67 (d, *J* = 7.0 Hz, 3H).

^{13}C NMR (151 MHz, Chloroform-d) δ 173.4, 157.9, 149.2, 140.9, 138.7, 135.3, 133.9, 129.9, 129.5, 129.1, 129.0, 128.6, 127.5, 126.2(8), 126.2(6), 121.4, 119.2, 105.7, 54.7, 45.7, 41.4, 18.7 *one peak is missing due to overlap*.

HRMS (ESI) m/z calcd. for $C_{27}H_{25}O_3$ ([M+H]$^+$): 397.1798, found: 397.1791.

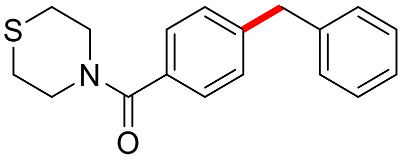

(4-Benzylphenyl)(thiomorpholino)methanone (51): Colorless oil was obtained with 46% isolated yield following the general procedure B (0.3 mmol scale, 41.0 mg, average yield of two times).

^{1}H NMR (400 MHz, Chloroform-d) δ 7.30 – 7.26 (m, 4H), 7.24 – 7.16 (m, 5H), 4.01 – 3.67 (m, 6H), 2.59 – 2.54 (m, 4H).

^{13}C NMR (151 MHz, Chloroform-d) δ 170.6, 143.0, 140.2, 133.4, 129.0, 128.9, 128.5, 127.0, 126.2, 50.1, 44.5, 41.7, 27.9, 27.4.

HRMS (ESI) m/z calcd. for $C_{18}H_{20}ONS$ ([M+H]$^+$): 298.1260, found: 298.1253.

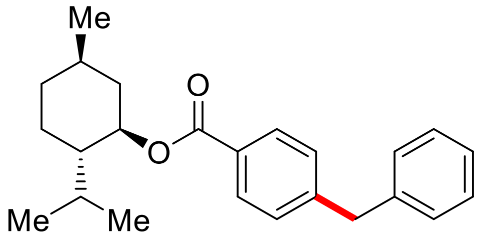

(1R,2S,5R)-2-Isopropyl-5-methylcyclohexyl 4-benzylbenzoate (52)[6]: Colorless oil was obtained with 71% isolated yield following the general procedure B (0.3 mmol scale, 74.7 mg).

^{1}H NMR (600 MHz, Chloroform-d) δ 8.01 – 7.99 (m, 2H), 7.33 – 7.30 (m, 2H), 7.28 (d, J = 8.2 Hz, 2H), 7.25 – 7.22 (m, 1H), 7.21 – 7.19 (m, 2H), 4.95 (td, J = 10.8, 4.4 Hz, 1H), 4.05 (s, 2H), 2.17 – 2.13 (m, 1H), 2.01 – 1.96 (m, 1H), 1.77 – 1.73 (m, 2H), 1.61 – 1.54 (m, 2H), 1.17 – 1.09 (m, 2H), 0.96 – 0.93 (m, 7H), 0.82 (d, J = 7.0 Hz, 3H).

^{13}C NMR (151 MHz, Chloroform-d) δ 166.1, 146.4, 140.3, 129.9, 129.0(2), 128.9(8), 128.9, 128.7, 126.5, 74.8, 47.4, 42.0, 41.1, 34.4, 31.5, 26.6, 23.7, 22.2, 20.9, 16.6.

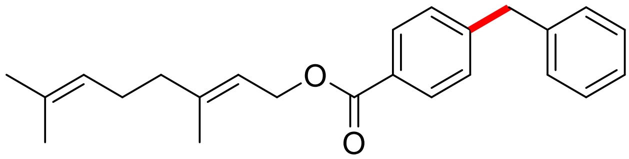

(*E*)-3,7-dimethylocta-2,6-dien-1-yl 4-benzylbenzoate (53)[6]: Colorless oil was obtained with 70% isolated yield following the general procedure B (0.3 mmol scale, 73.2 mg).

^{1}H NMR (600 MHz, Chloroform-d) δ 7.96 (d, J = 8.2 Hz, 2H), 7.30 – 7.27 (m, 2H), 7.24 (d, J = 8.2 Hz, 2H), 7.22 – 7.19 (m, 1H), 7.16 (d, J = 7.0 Hz, 2H), 5.45 (t, J = 7.6 Hz, 1H), 5.10 – 5.08 (m, 1H), 4.82 (d, J = 7.0 Hz, 2H), 4.02 (s, 2H), 2.14 – 2.10 (m, 2H), 2.07 – 2.05 (m, 2H), 1.75 (s, 3H), 1.67 (s, 3H), 1.60 (s, 3H).

^{13}C NMR (151 MHz, Chloroform-d) δ 166.7, 146.5, 142.4, 140.3, 132.0, 130.0, 129.1, 129.0, 128.7, 128.6, 126.5, 123.9, 118.6, 61.9, 42.0, 39.7, 26.4, 25.8, 17.8, 16.7.

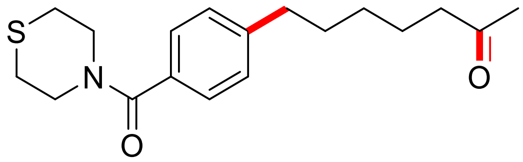

7-(4-(Thiomorpholine-4-carbonyl)phenyl)heptan-2-one (54): Colorless oil was obtained with 58% isolated yield following the general procedure C (0.3 mmol scale, 55.6 mg, average yield of two times).

^{1}H NMR (600 MHz, Chloroform-d) δ 7.29 (d, *J* = 8.2 Hz, 2H), 7.20 (d, *J* = 8.0 Hz, 2H), 4.00 – 3.70 (m, 4H), 2.71 – 2.59 (m, 6H), 2.42 (t, *J* = 7.4 Hz, 2H), 2.13 (s, 3H), 1.64 – 1.58 (m, 4H), 1.35 – 1.30 (m, 2H).

^{13}C NMR (151 MHz, Chloroform-d) δ 209.1, 170.9, 144.5, 133.1, 128.6, 126.9, 50.2, 44.6, 43.6, 35.5, 31.0, 29.9, 28.6, 27.8, 27.5, 23.5.

HRMS (ESI) m/z calcd. for $C_{18}H_{26}NO_2S$ ($[M+H]^+$): 320.1679, found: 320.1670.

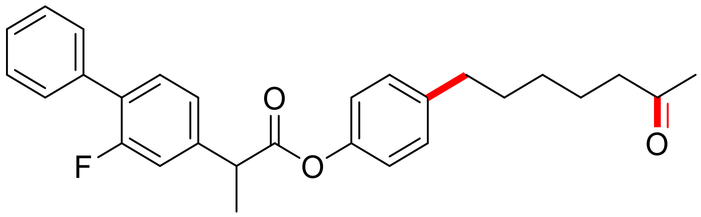

4-(6-Oxoheptyl)phenyl 2-(2-fluoro-[1,1'-biphenyl]-4-yl)propanoate (55): Colorless oil was obtained with 71% isolated yield following the general procedure C (0.3 mmol scale, 92.1 mg).

^{1}H NMR (600 MHz, Chloroform-d) δ 7.55 (d, *J* = 8.4 Hz, 2H), 7.45 – 7.42 (m, 3H), 7.38 – 7.35 (m, 1H), 7.25 – 7.21 (m, 2H), 7.13 (d, *J* = 8.4 Hz, 2H), 6.93 (d, *J* = 8.4 Hz, 2H), 3.98 (q, *J* = 7.0 Hz, 1H), 2.58 (t, *J* = 7.6 Hz, 2H), 2.40 (t, *J* = 7.4 Hz, 2H), 2.11 (s, 3H), 1.64 (d, *J* = 7.2 Hz, 3H), 1.62 – 1.55 (m, 4H), 1.32 – 1.29 (m, 2H).

^{13}C NMR (151 MHz, Chloroform-d) δ 209.2, 172.8, 159.9 (d, *J* = 248.6 Hz), 148.8, 141.5 (d, *J* = 7.6 Hz), 140.3, 135.5, 131.1 (d, *J* = 3.8 Hz), 129.4, 129.1 (d, *J* = 3.0 Hz), 128.6, 128.1 (d, *J* = 13.6 Hz), 127.8, 123.7 (d, *J* = 3.2 Hz), 120.7, 115.4 (d, *J* = 23.6 Hz), 45.2, 43.7, 35.9, 31.3, 30.0, 28.8, 23.7, 18.5.

^{19}F NMR (565 MHz, Chloroform-d) δ -117.36 (m).

HRMS (ESI) m/z calcd. for $C_{28}H_{30}O_3F$ ($[M+H]^+$): 433.2173, found: 433.2173.

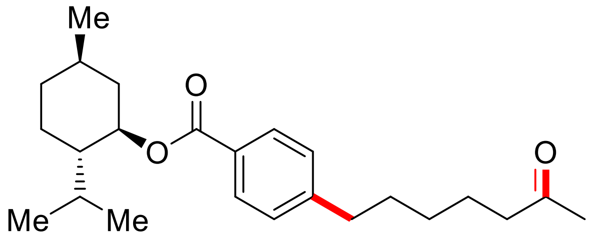

(1R,2S,5R)-2-Isopropyl-5-methylcyclohexyl 4-(6-oxoheptyl)benzoate (56): Colorless oil was obtained with 58% isolated yield following the general procedure C (0.3 mmol scale, 64.8 mg, average yield of two times).

^{1}H NMR (600 MHz, Chloroform-d) δ 7.93 (d, J = 8.2 Hz, 2H), 7.21 (d, J = 8.2 Hz, 2H), 4.90 (td, J = 10.8, 4.4 Hz, 1H), 2.63 (t, J = 7.6 Hz, 2H), 2.39 (t, J = 7.4 Hz, 2H), 2.11 – 2.07 (m, 4H), 1.97 – 1.92 (m, 1H), 1.72 – 1.68 (m, 2H), 1.64 – 1.50 (m, 6H), 1.32 – 1.27 (m, 2H), 1.14 – 1.04 (m, 2H), 0.90 – 0.88 (m, 7H), 0.77 (d, J = 7.0 Hz, 3H).

^{13}C NMR (151 MHz, Chloroform-d) δ 208.9, 166.1, 147.9, 129.7, 128.5, 128.4, 74.6, 47.3, 43.6, 41.0, 35.8, 34.4, 31.5, 31.0, 29.9, 28.7, 26.5, 23.7, 23.6, 22.1, 20.8, 16.6.

HRMS (ESI) m/z calcd. for $C_{24}H_{37}O_3$ ([M+H]$^+$): 373.2737, found: 373.2733.

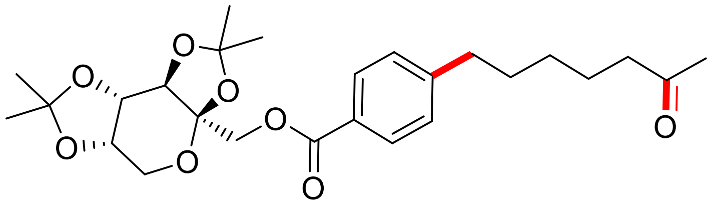

((3aR)-2,2,7,7-Tetramethyltetrahydro-3aH-bis([1,3]dioxolo)[4,5-b:4',5'-d]pyran-3a-yl)methyl 4-(6-oxoheptyl)benzoate (57): Colorless oil was obtained with 79% isolated yield following the general procedure C (0.3 mmol scale, 112.9 mg).

^{1}H NMR (600 MHz, Chloroform-d) δ 7.88 (d, J = 8.2 Hz, 2H), 7.19 (d, J = 8.2 Hz, 2H), 5.89 (d, J = 3.6 Hz, 1H), 5.42 (d, J = 2.8 Hz, 1H), 4.56 (d, J = 3.6 Hz, 1H), 4.32 – 4.27 (m, 2H), 4.06 – 4.02 (m, 2H), 2.61 (t, J = 7.6 Hz, 2H), 2.36 (t, J = 7.4 Hz, 2H), 2.06 (s, 3H), 1.59 – 1.53 (m, 4H), 1.49 (s, 3H), 1.35 (s, 3H), 1.29 – 1.25 (m, 5H), 1.21 (s, 3H).

^{13}C NMR (151 MHz, Chloroform-d) δ 208.8, 165.1, 148.8, 129.8, 128.6, 127.0, 112.2, 109.3, 105.1, 83.4, 79.9, 76.4, 72.6, 67.1, 43.4, 35.7, 30.8, 29.8, 28.6, 26.8, 26.7, 26.2, 25.2, 23.4.

HRMS (ESI) m/z calcd. for $C_{26}H_{37}O_8$ ($[M+H]^+$): 477.2483, found: 477.2467.

3 NMR Spectra

Compound 1

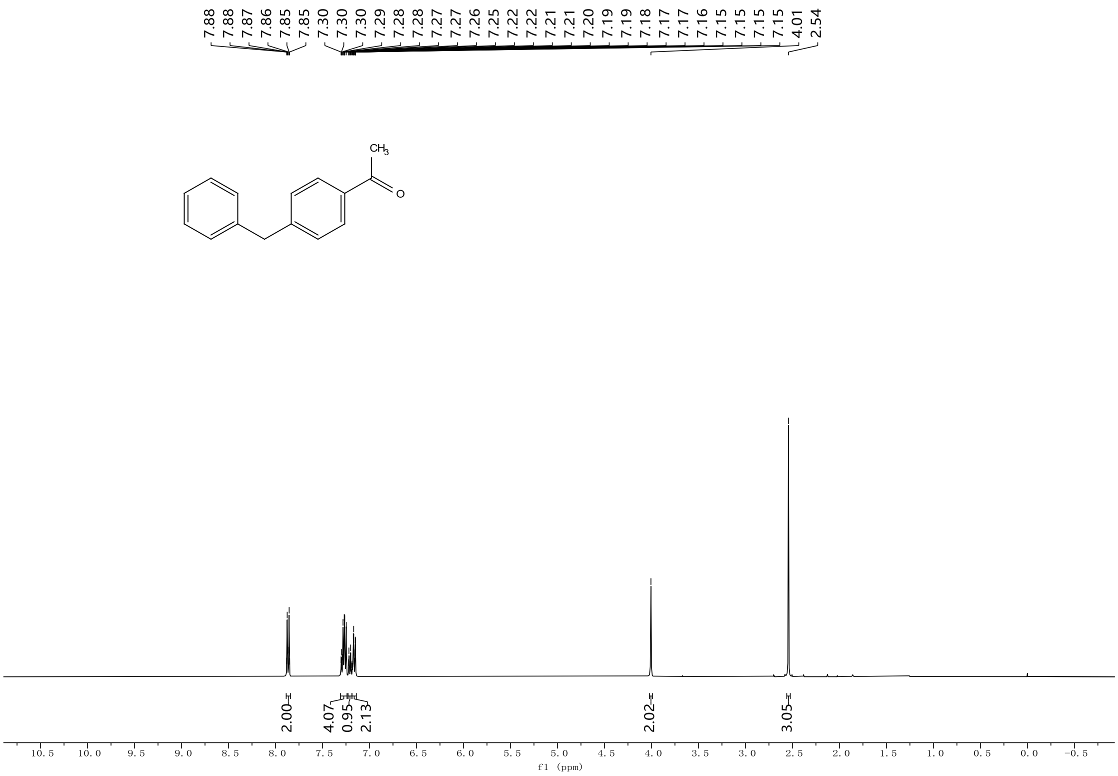

Supplementary Figure 31. ^{1}H NMR spectra of compound **1** (400 MHz, r.t., $CDCl_3$).

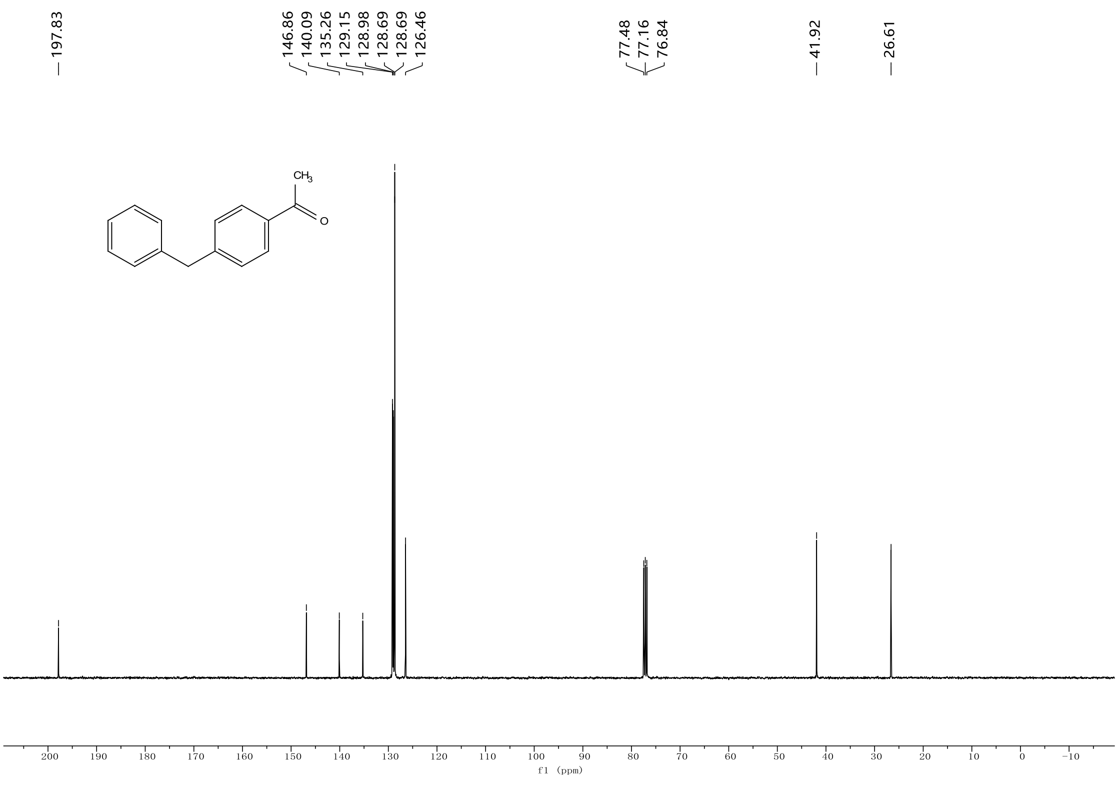

Supplementary Figure 32. ^{13}C NMR spectra of compound **1** (101 MHz, r.t., $CDCl_3$).

Compound 2

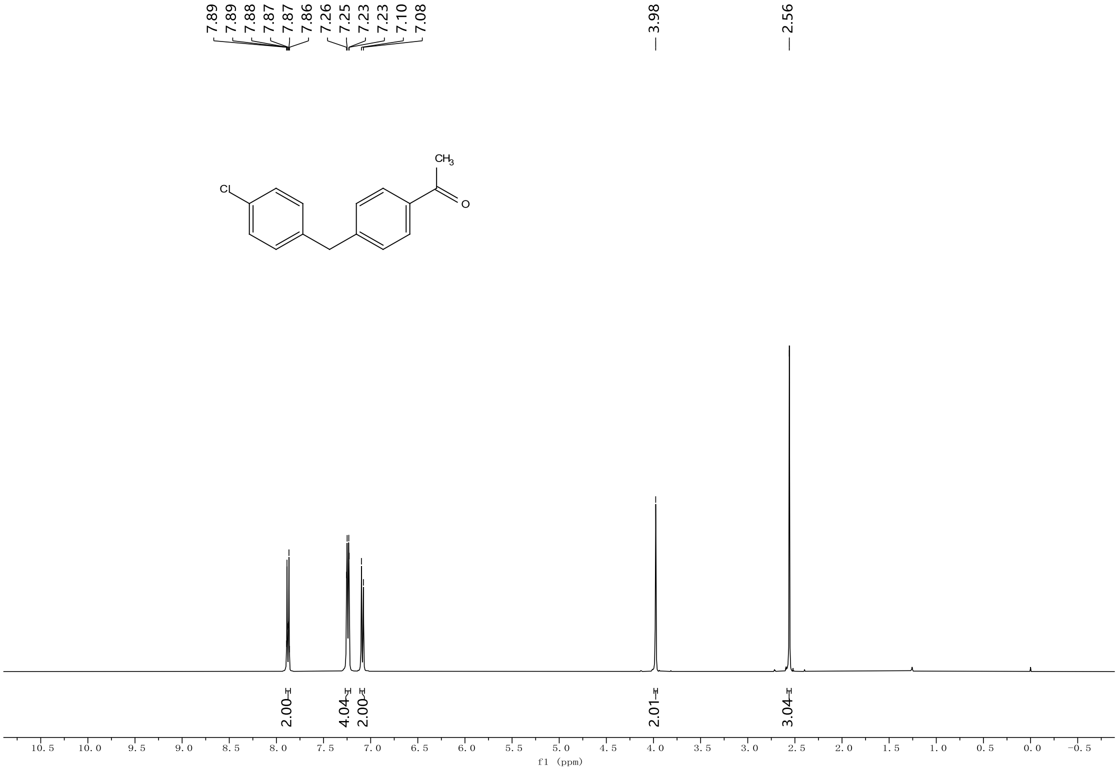

Supplementary Figure 33. ^{1}H NMR spectra of compound **2** (400 MHz, r.t., $CDCl_3$).

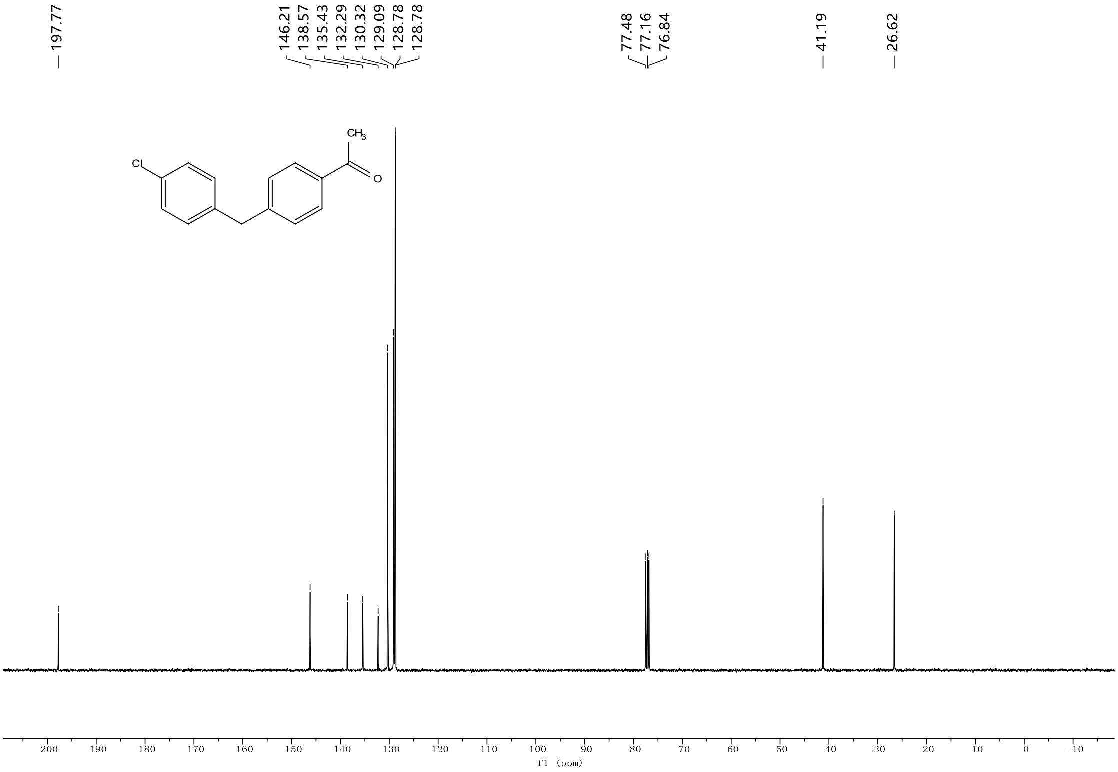

Supplementary Figure 34. ^{13}C NMR spectra of compound **2** (101 MHz, r.t., $CDCl_3$).

Compound 3

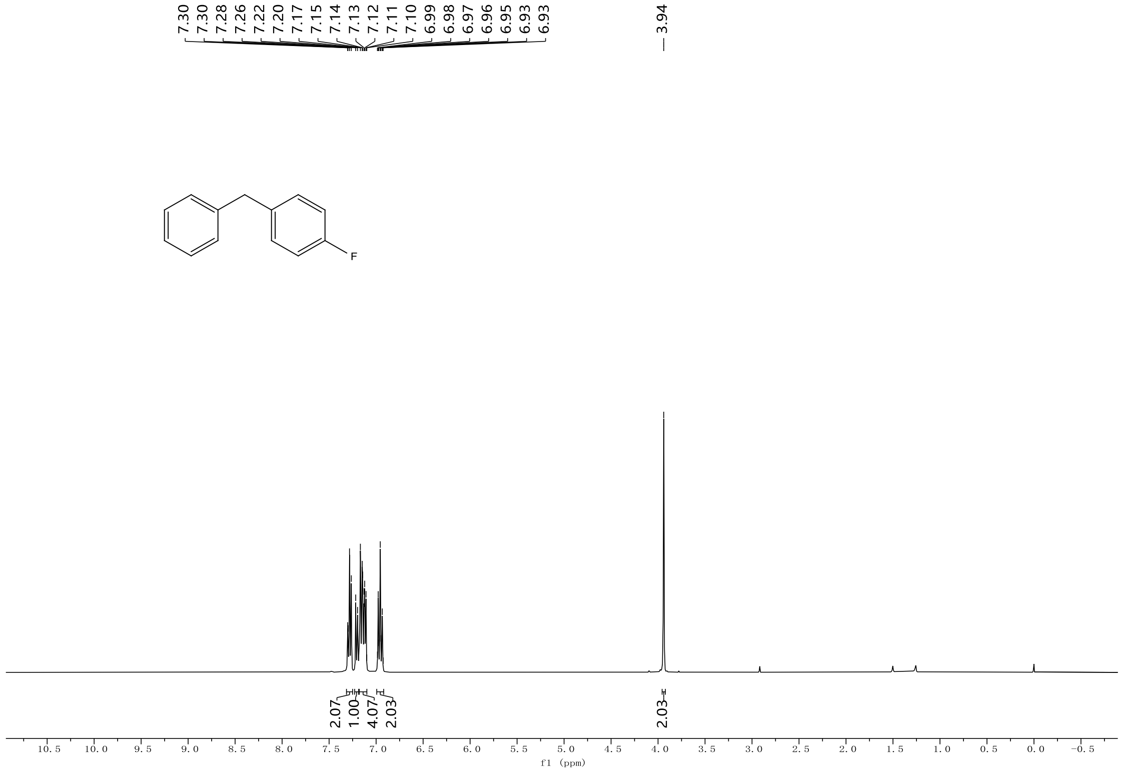

Supplementary Figure 35. ^{1}H NMR spectra of compound **3** (400 MHz, r.t., $CDCl_3$).

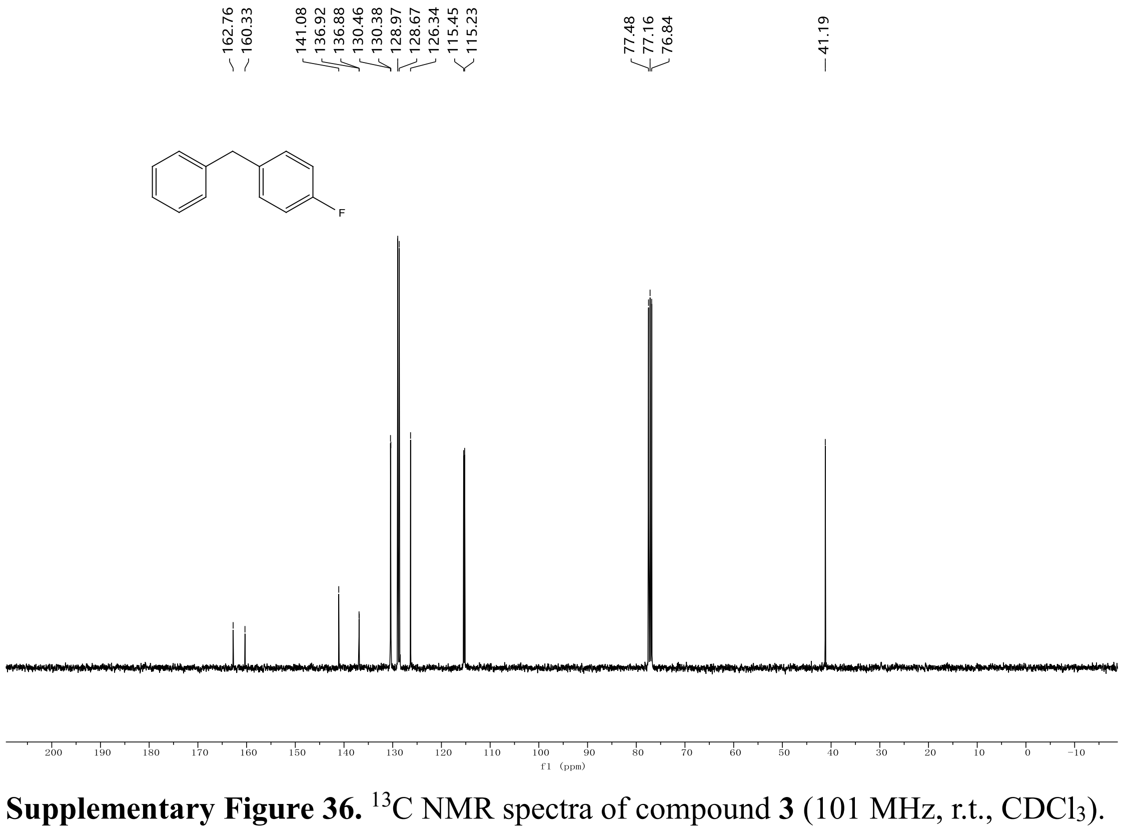

Supplementary Figure 36. ^{13}C NMR spectra of compound **3** (101 MHz, r.t., $CDCl_3$).

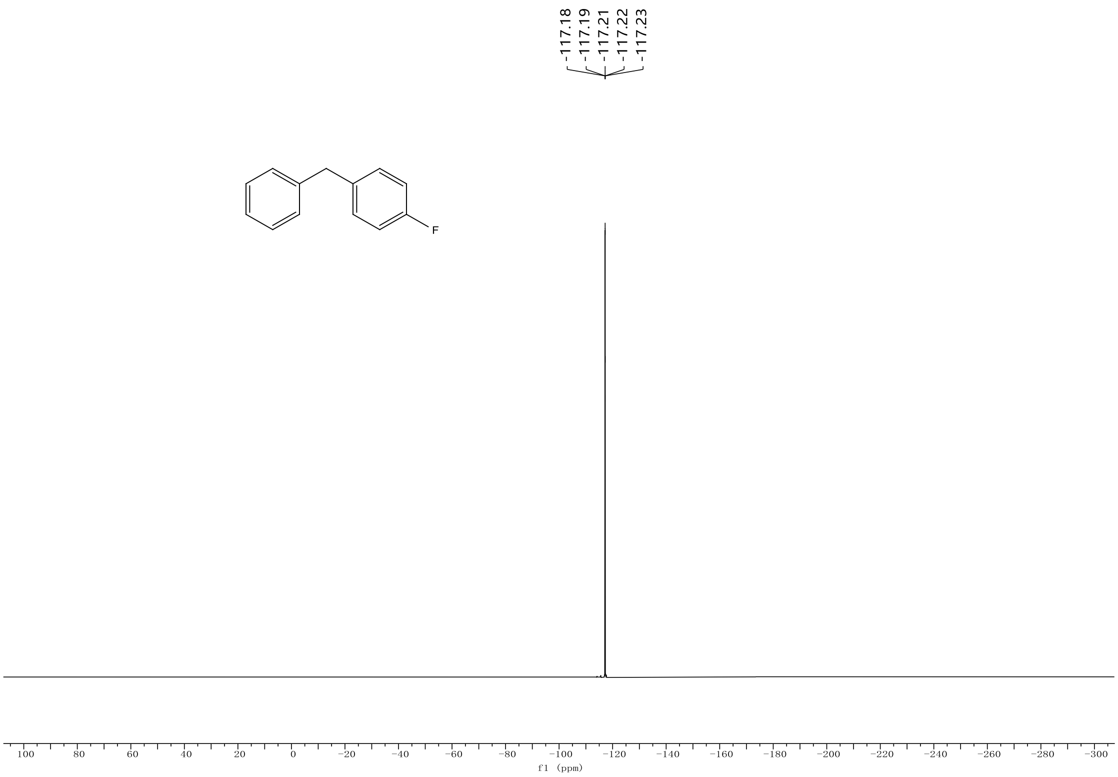

Supplementary Figure 37. ^{19}F NMR spectra of compound **3** (376 MHz, r.t., $CDCl_3$).

Compound 4

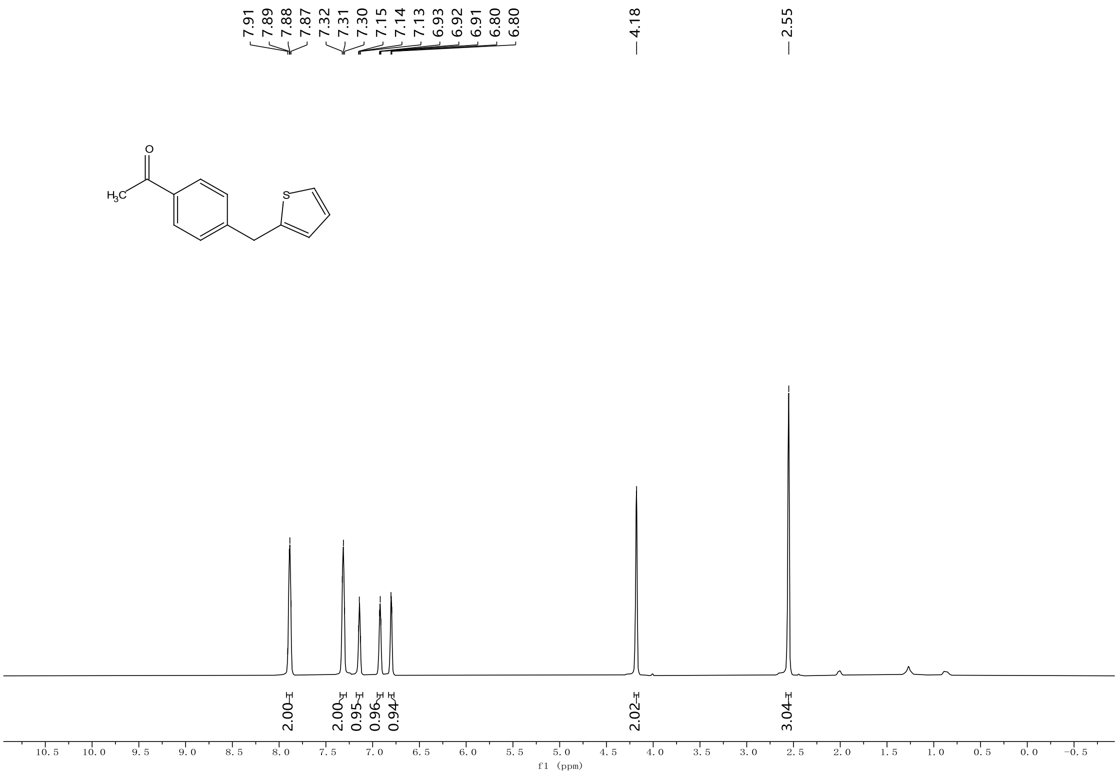

Supplementary Figure 38. ^{1}H NMR spectra of compound **4** (400 MHz, r.t., $CDCl_3$).

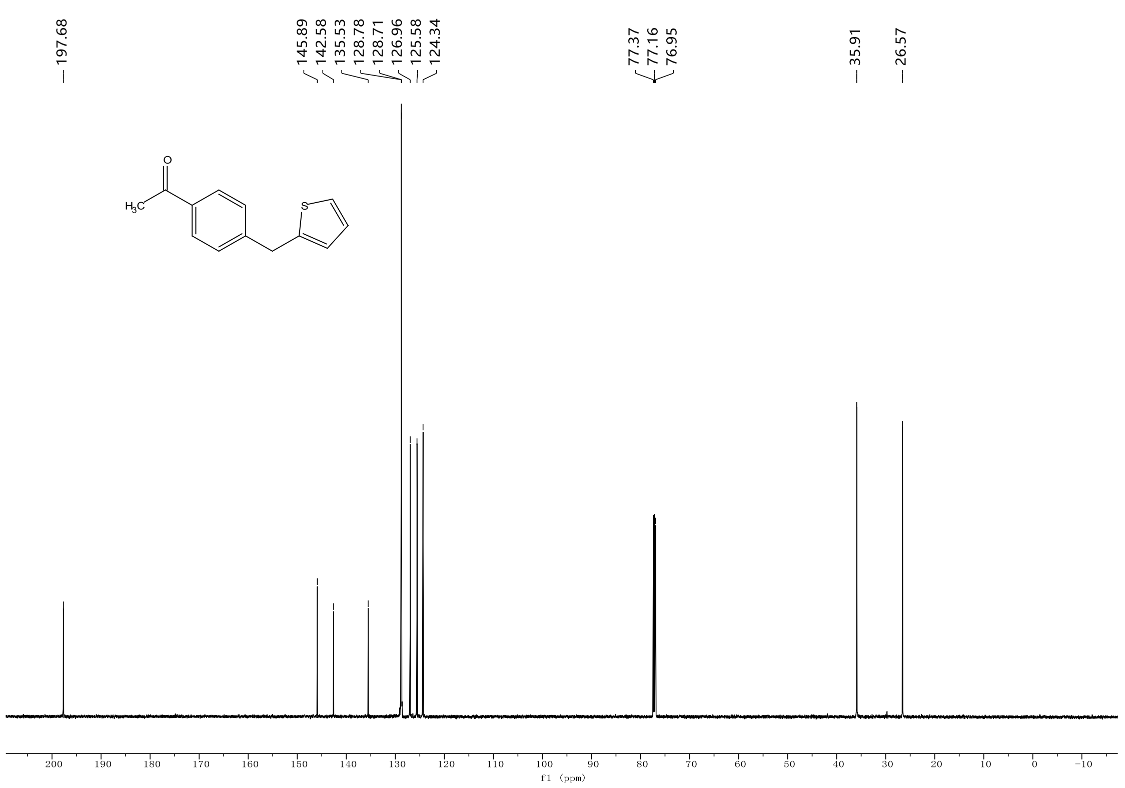

Supplementary Figure 39. ^{13}C NMR spectra of compound **4** (101 MHz, r.t., $CDCl_3$).

Compound 5

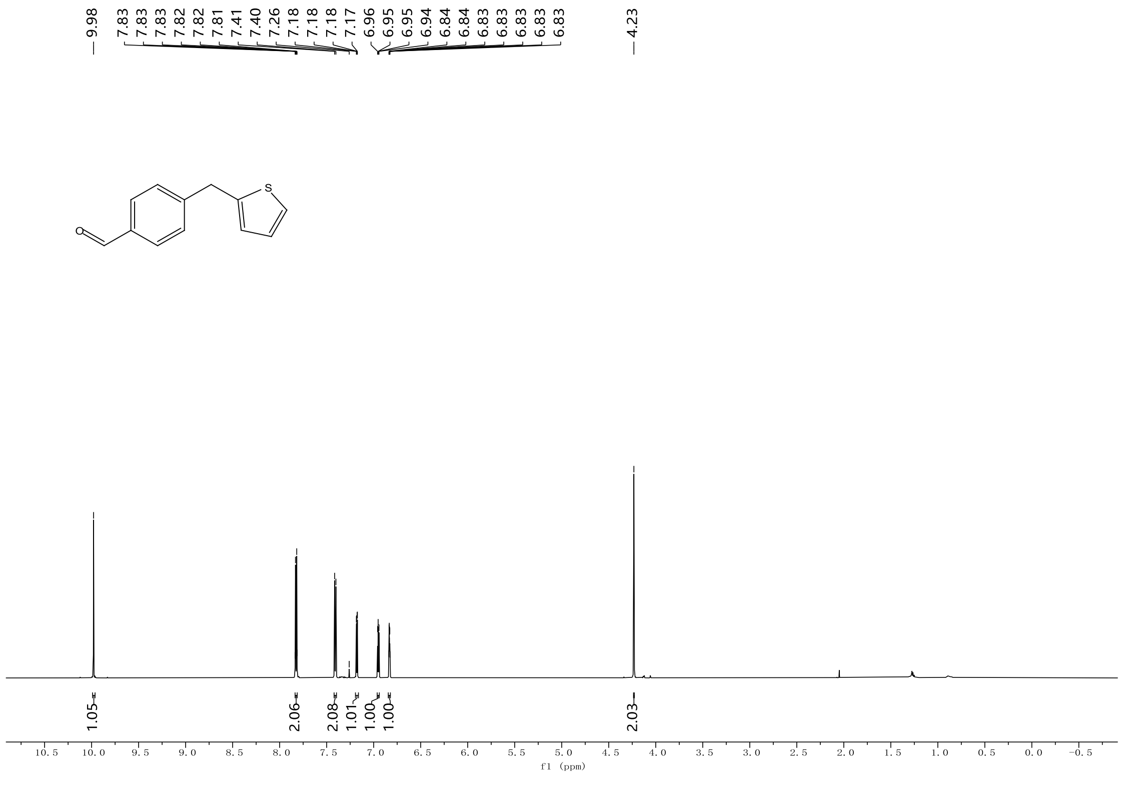

Supplementary Figure 40. ^{1}H NMR spectra of compound **5** (600 MHz, r.t., $CDCl_3$).

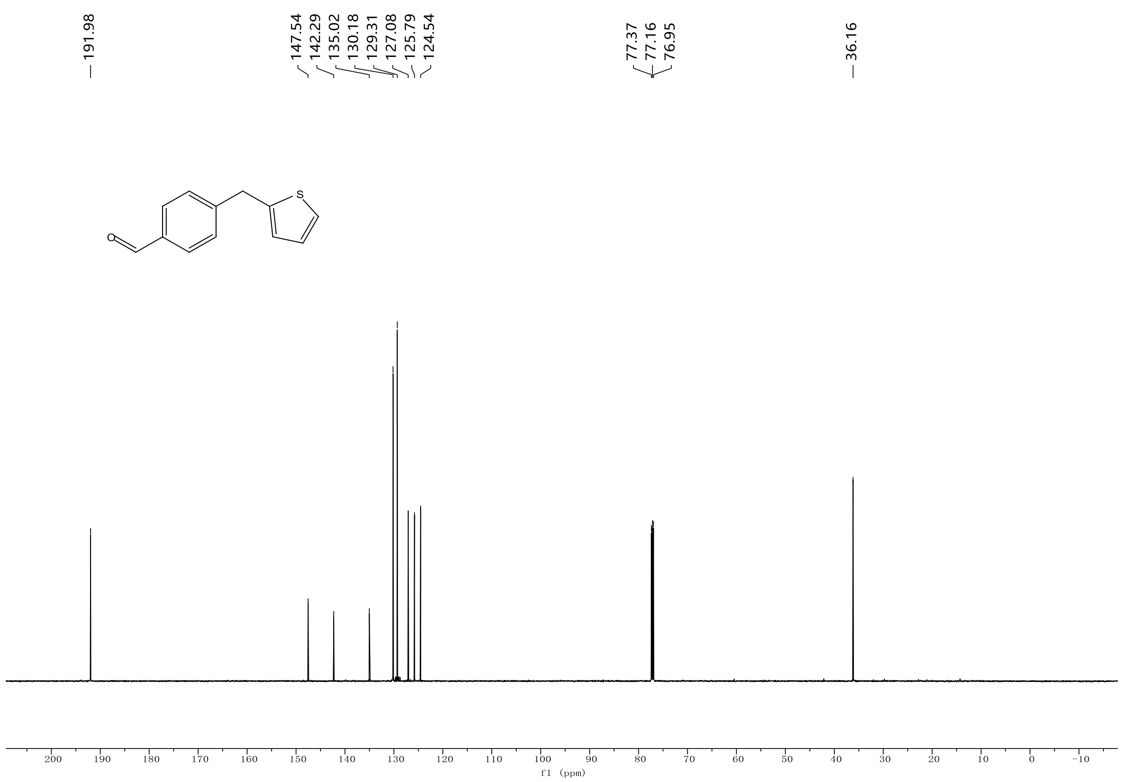

Supplementary Figure 41. ^{13}C NMR spectra of compound **5** (151 MHz, r.t., $CDCl_3$).

Compound 6

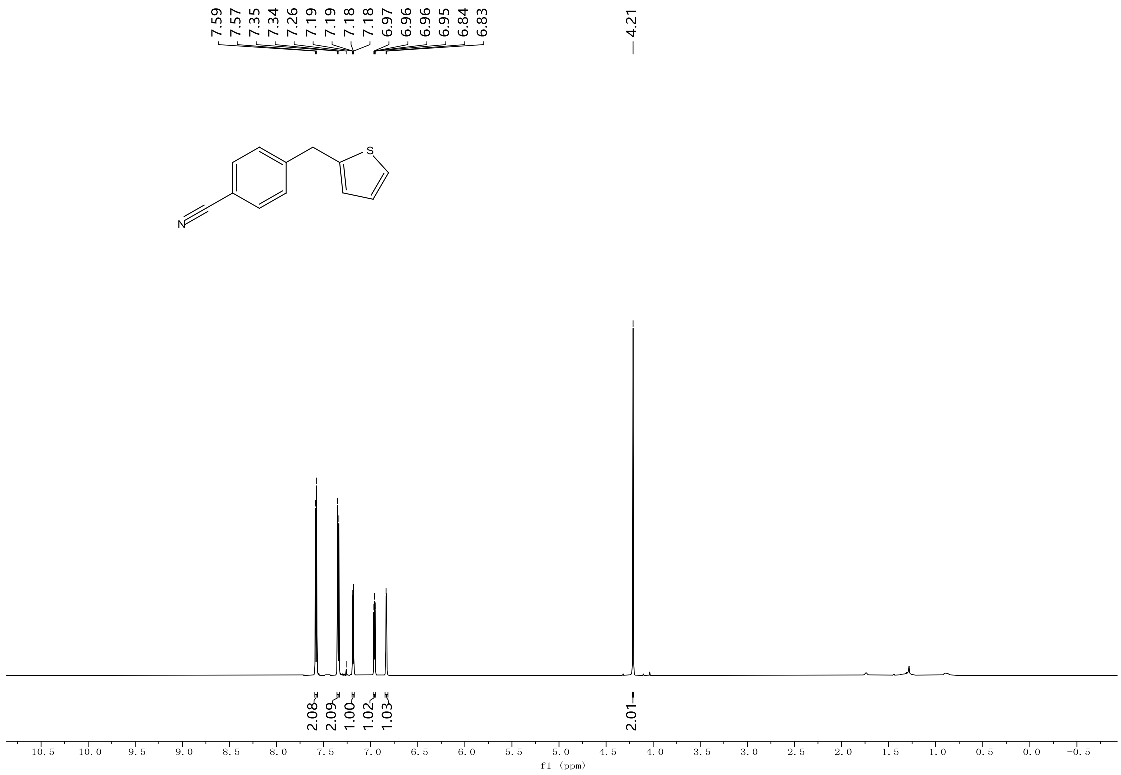

Supplementary Figure 42. ^{1}H NMR spectra of compound **6** (600 MHz, r.t., $CDCl_3$).

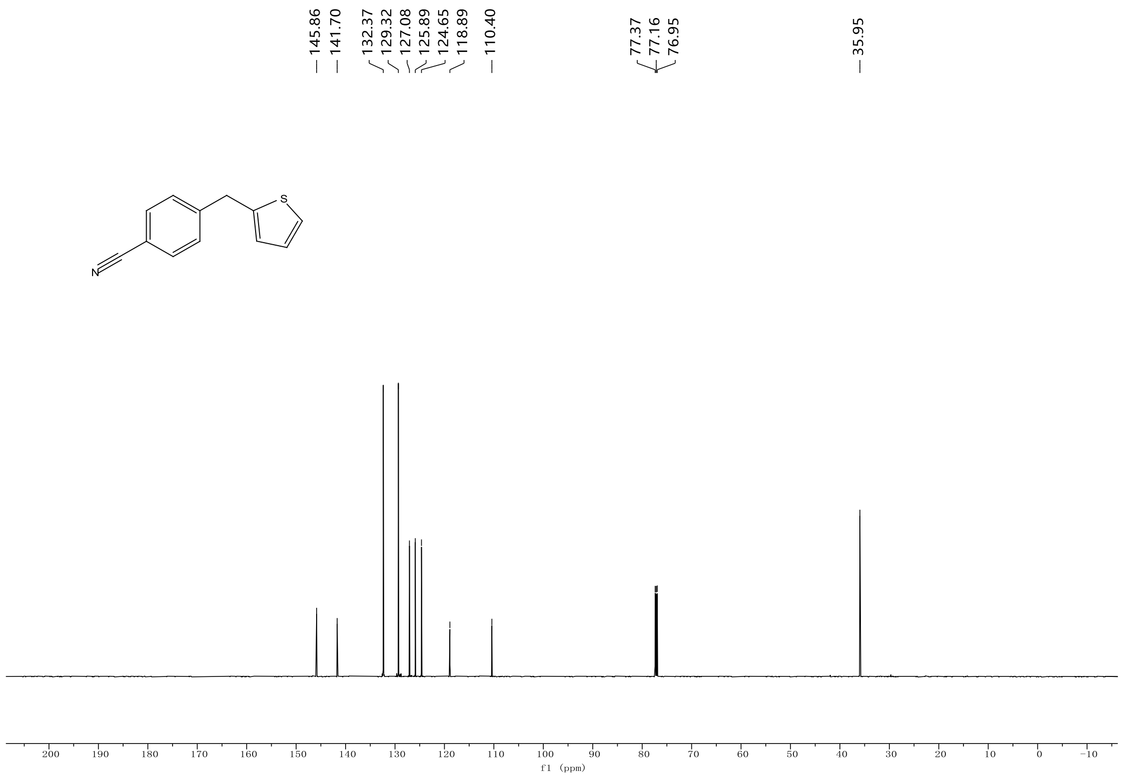

Supplementary Figure 43. ^{13}C NMR spectra of compound **6** (151 MHz, r.t., $CDCl_3$).

Compound 7

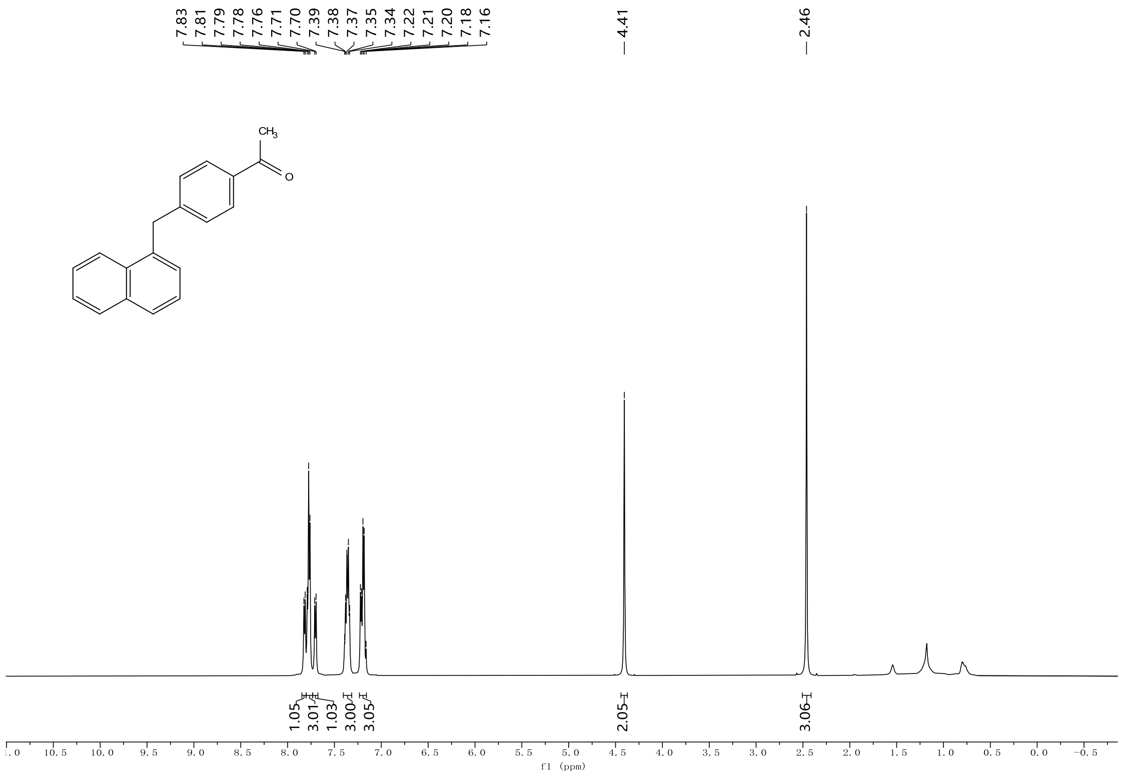

Supplementary Figure 44. ^{1}H NMR spectra of compound **7** (600 MHz, r.t., $CDCl_3$).

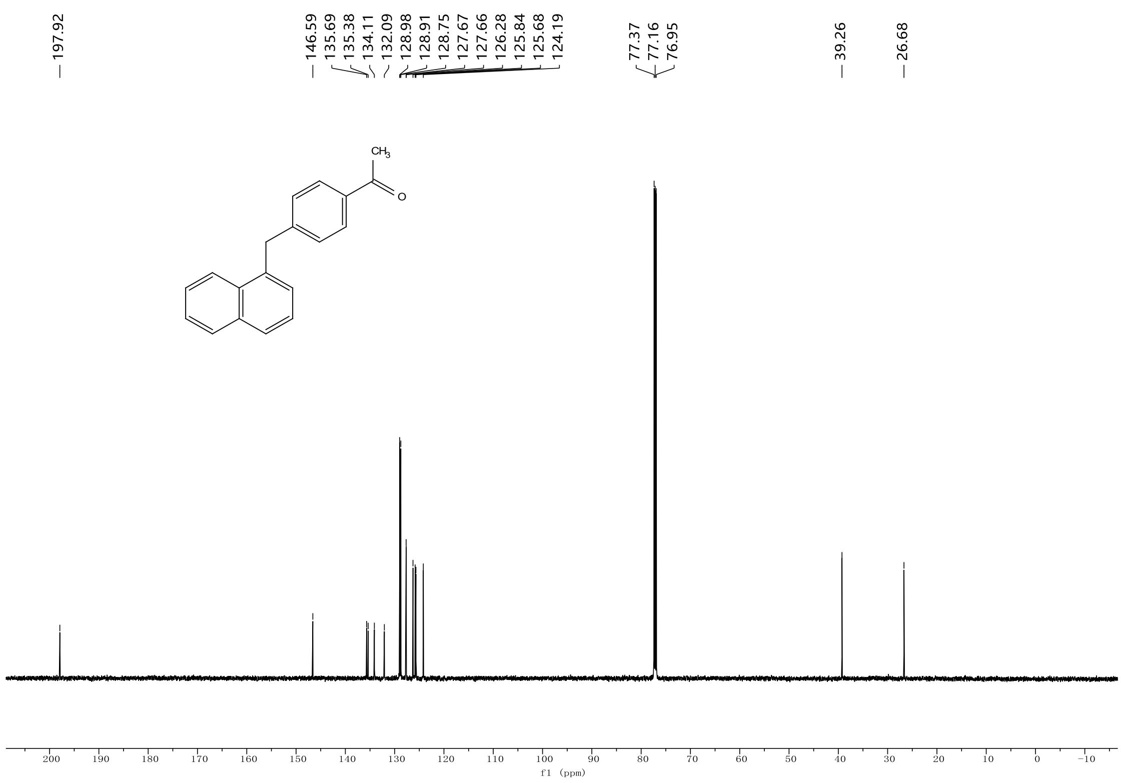

Supplementary Figure 45. ^{13}C NMR spectra of compound **7** (151 MHz, r.t., $CDCl_3$).

Compound 8

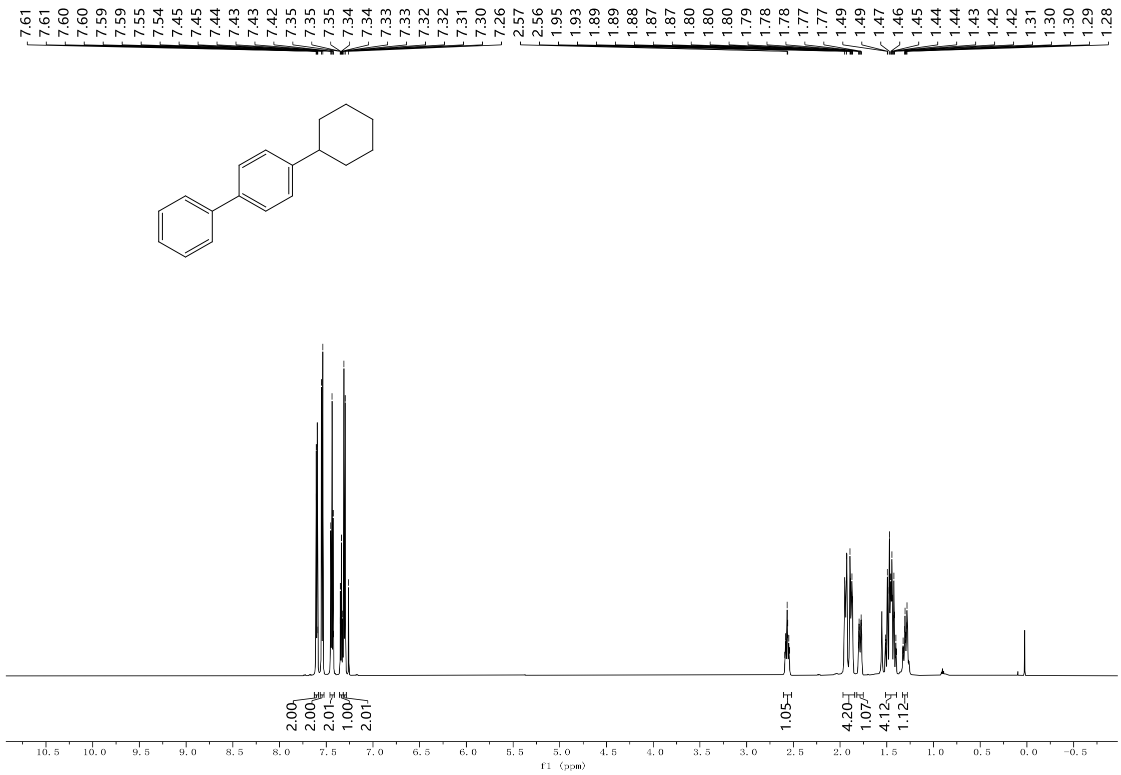

Supplementary Figure 46. ^{1}H NMR spectra of compound **8** (600 MHz, r.t., $CDCl_3$).

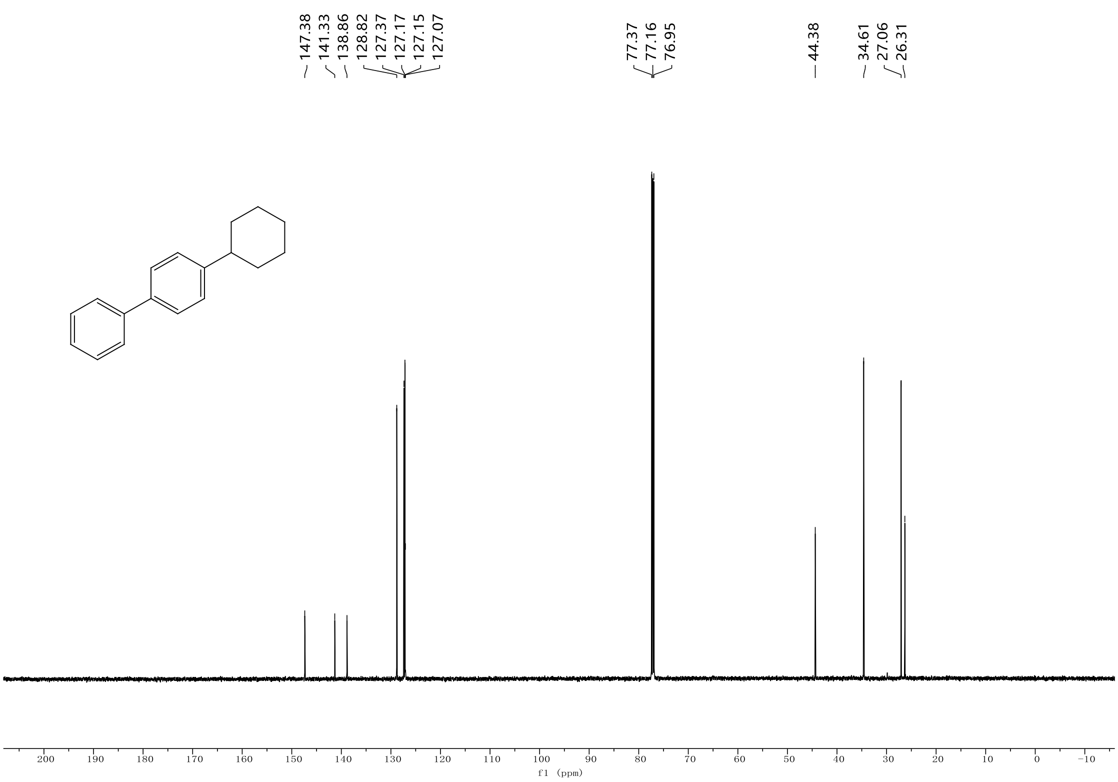

Supplementary Figure 47. ^{13}C NMR spectra of compound **8** (151 MHz, r.t., $CDCl_3$).

Compound 9

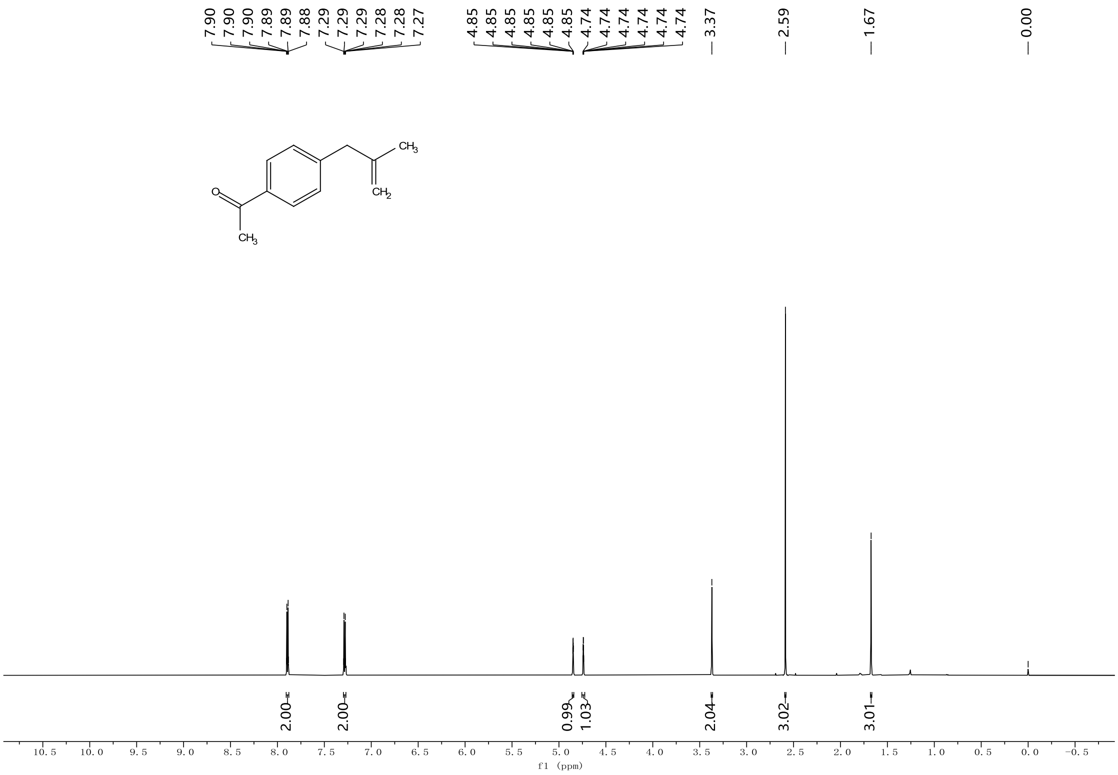

Supplementary Figure 48. ^{1}H NMR spectra of compound **9** (600 MHz, r.t., $CDCl_3$).

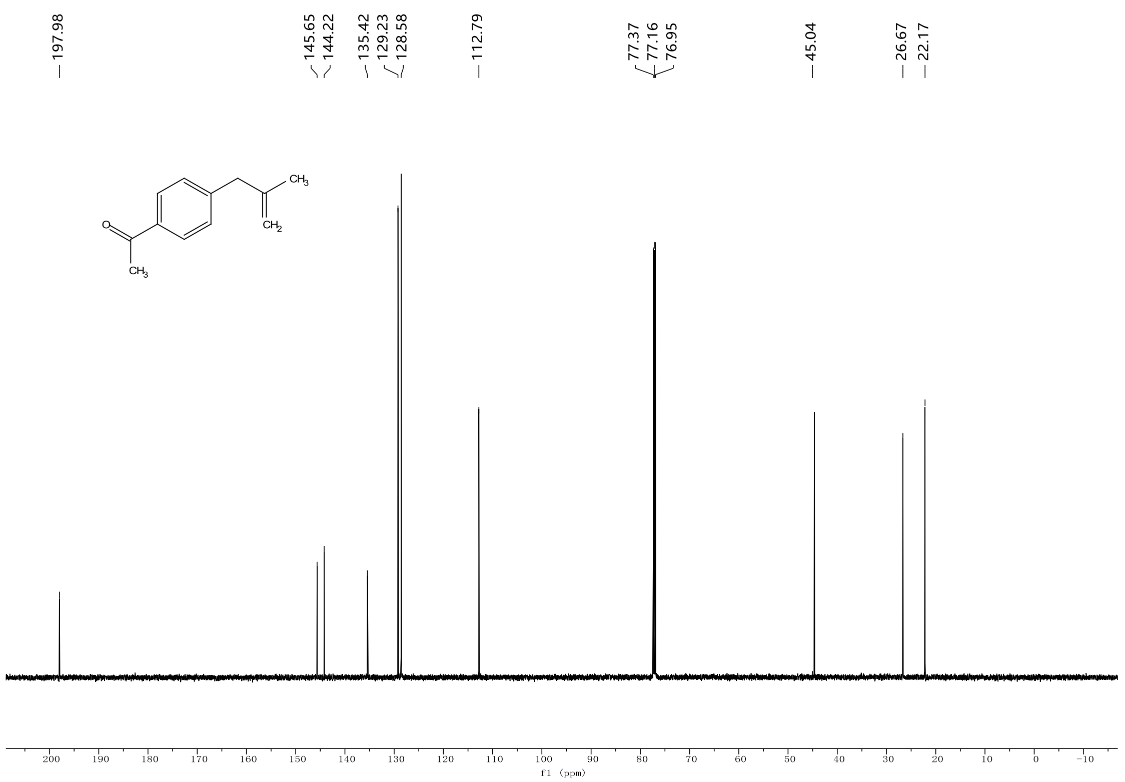

Supplementary Figure 49. ^{13}C NMR spectra of compound **9** (151 MHz, r.t., $CDCl_3$).

Compound 10

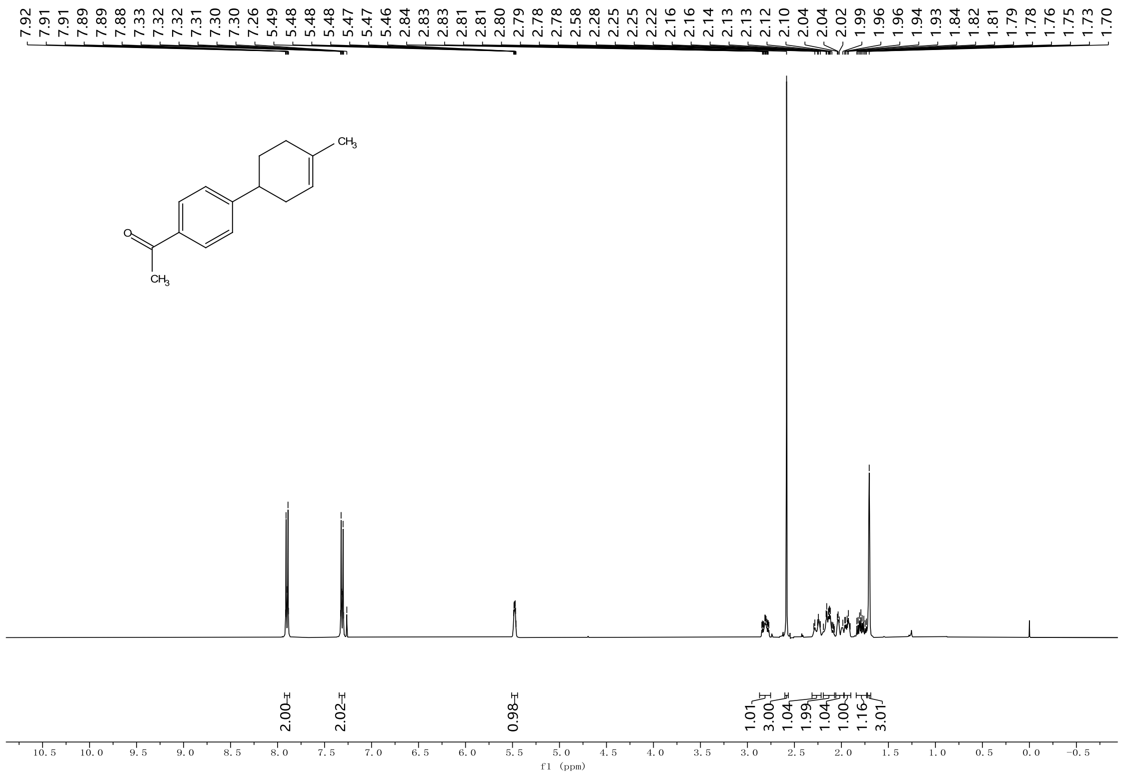

Supplementary Figure 50. ^{1}H NMR spectra of compound **10** (400 MHz, r.t., $CDCl_3$).

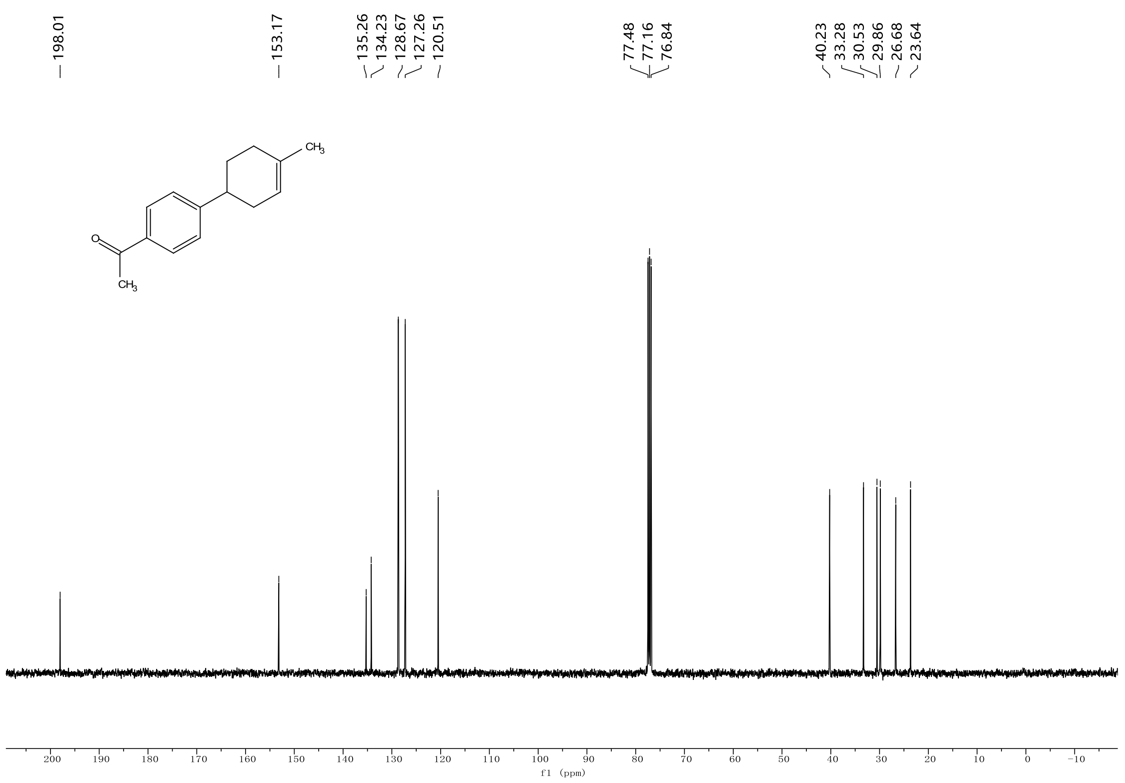

Supplementary Figure 51. ^{13}C NMR spectra of compound **10** (101 MHz, r.t., $CDCl_3$).

Compound 11

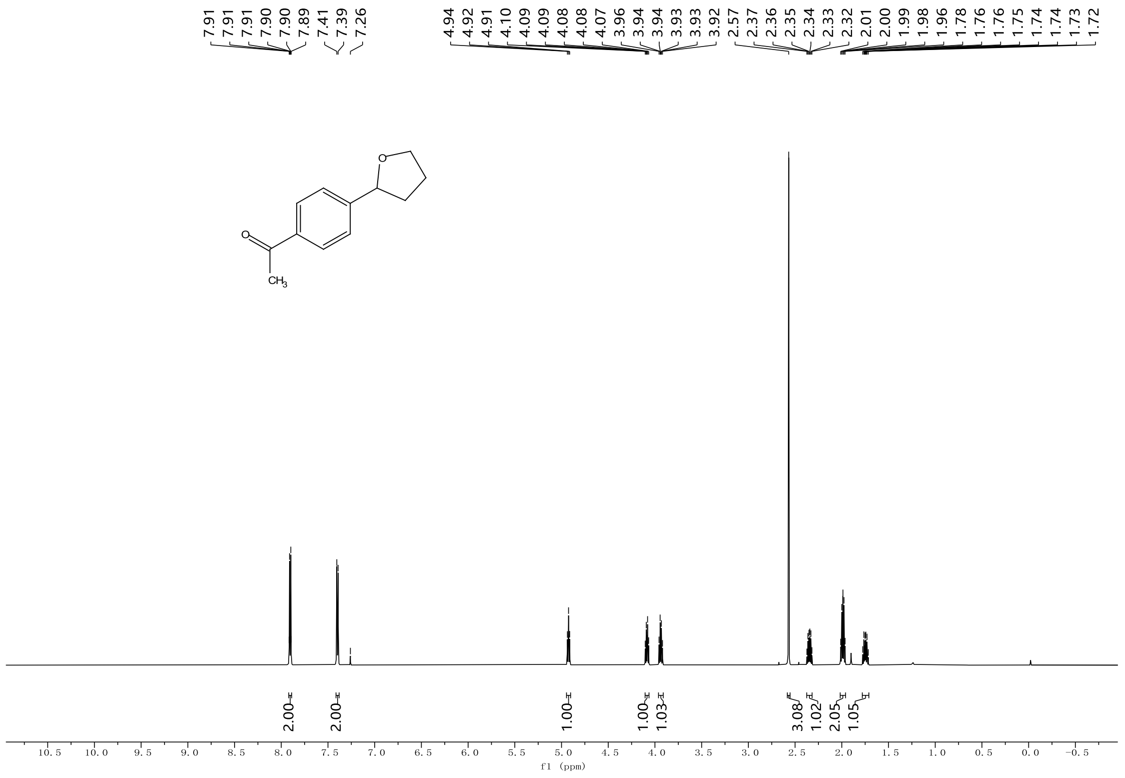

Supplementary Figure 52. ^{1}H NMR spectra of compound **11** (600 MHz, r.t., $CDCl_3$).

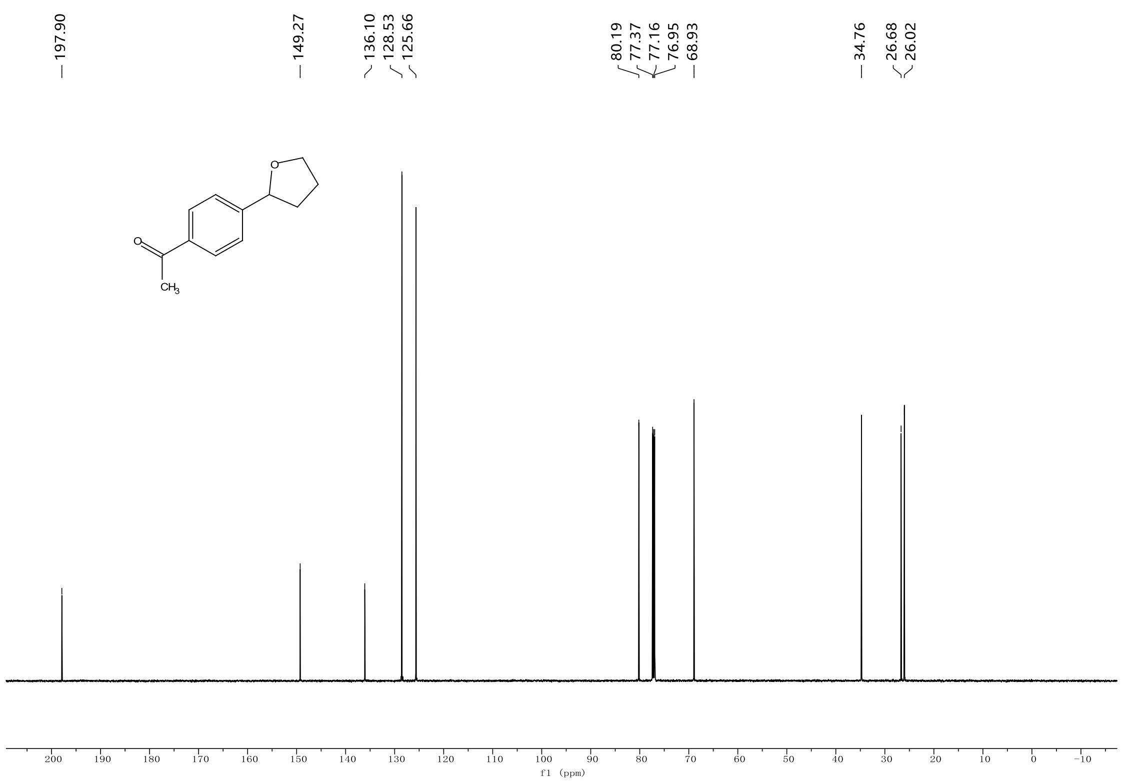

Supplementary Figure 53. ^{13}C NMR spectra of compound **11** (151 MHz, r.t., $CDCl_3$).

Compound 12

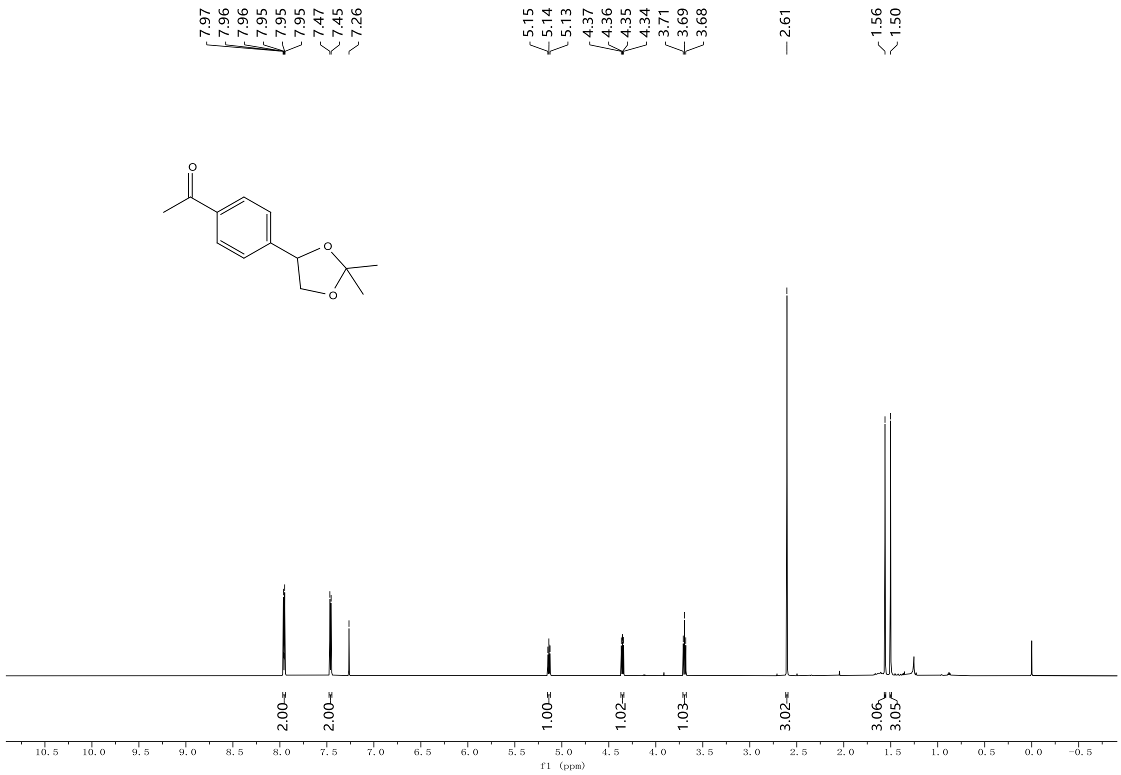

Supplementary Figure 54. ^{1}H NMR spectra of compound **12** (600 MHz, r.t., $CDCl_3$).

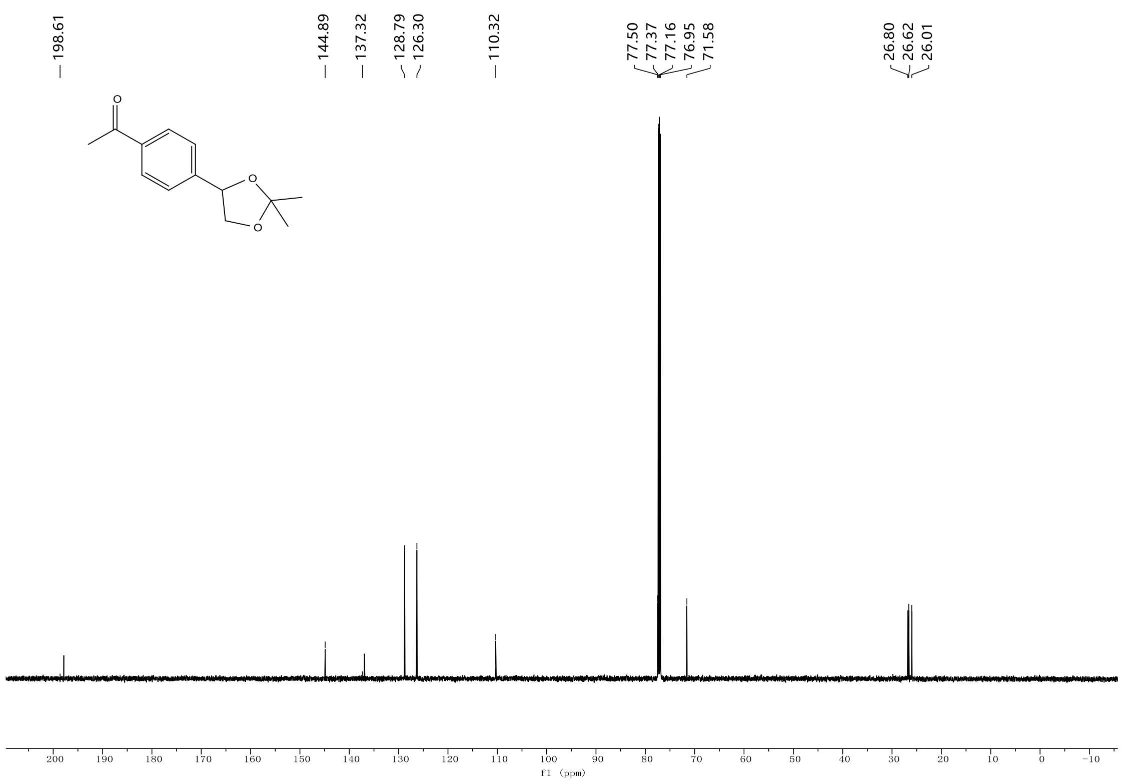

Supplementary Figure 55. ^{13}C NMR spectra of compound **12** (151 MHz, r.t., $CDCl_3$).

Compound 13

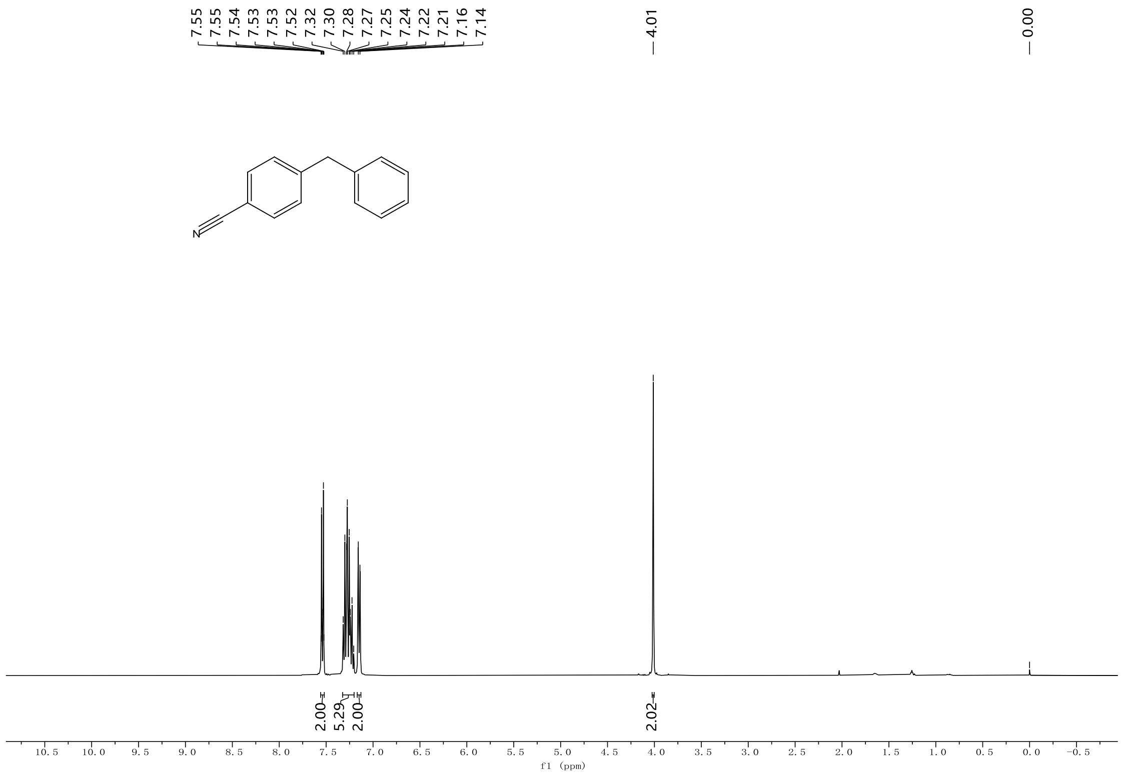

Supplementary Figure 56. ^{1}H NMR spectra of compound **13** (400 MHz, r.t., $CDCl_3$).

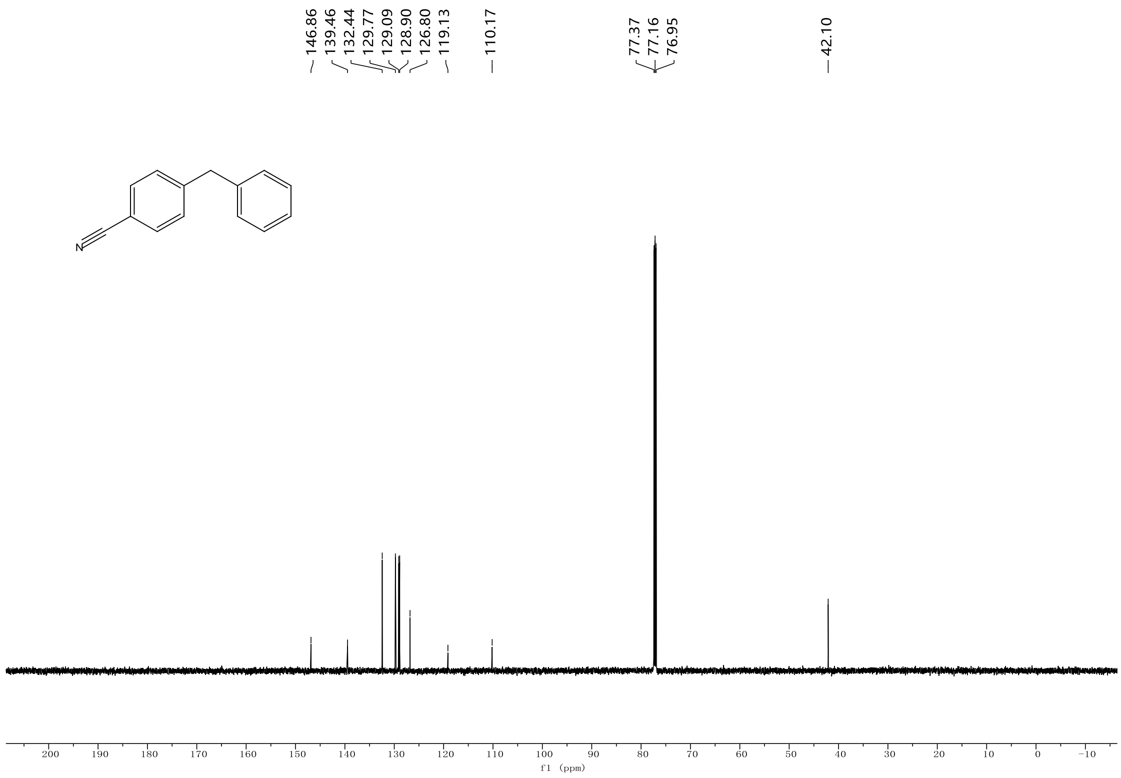

Supplementary Figure 57. ^{13}C NMR spectra of compound **13** (151 MHz, r.t., $CDCl_3$).

Compound 14

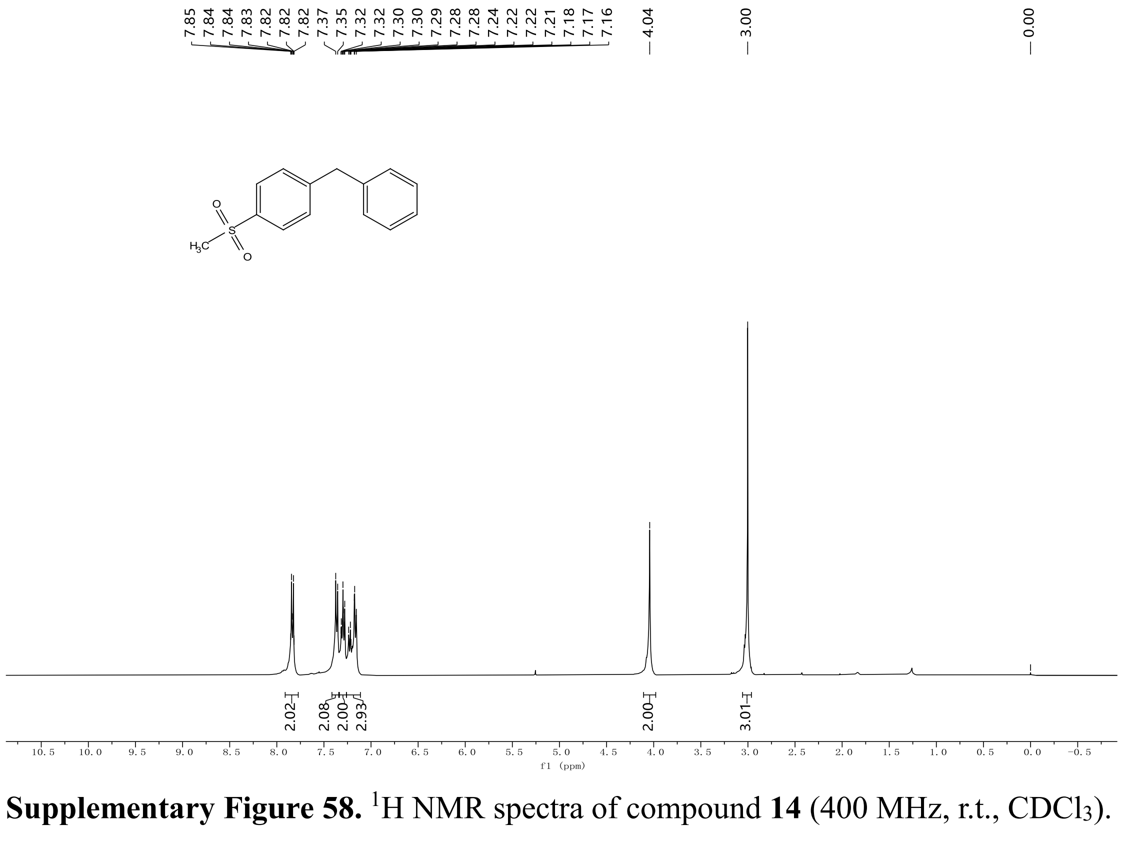

Supplementary Figure 58. ^{1}H NMR spectra of compound **14** (400 MHz, r.t., $CDCl_3$).

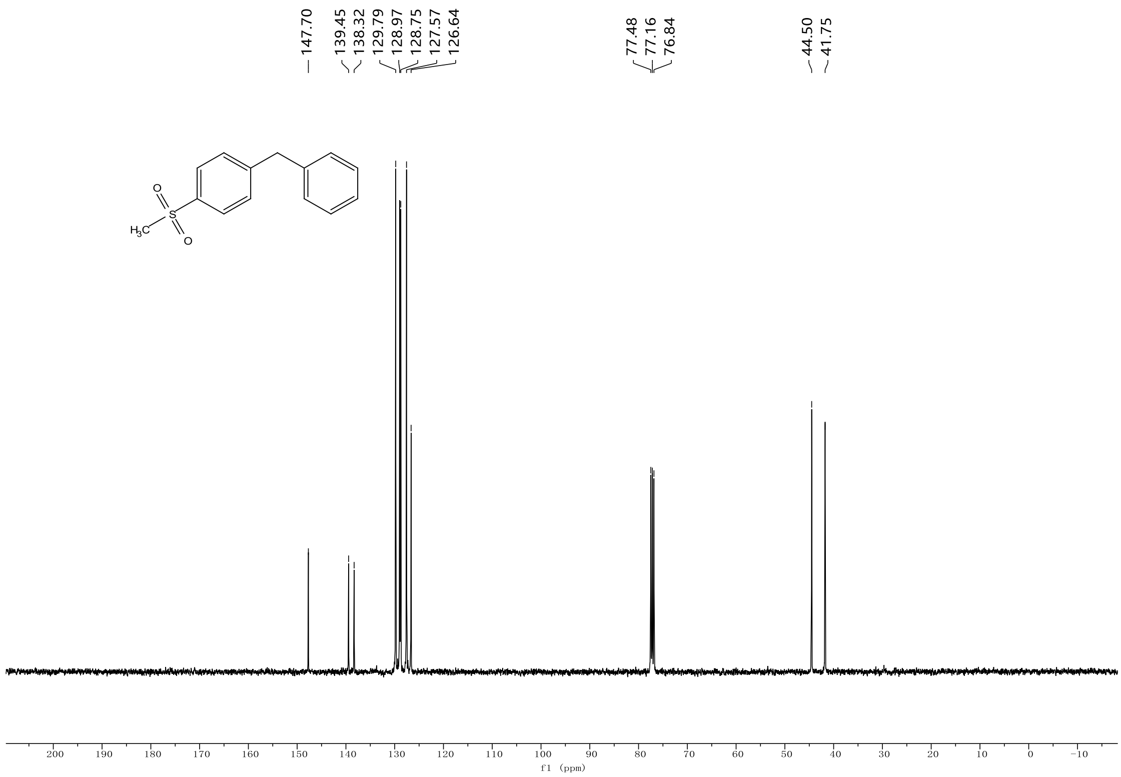

Supplementary Figure 59. ^{13}C NMR spectra of compound **14** (101 MHz, r.t., $CDCl_3$).

Compound 15

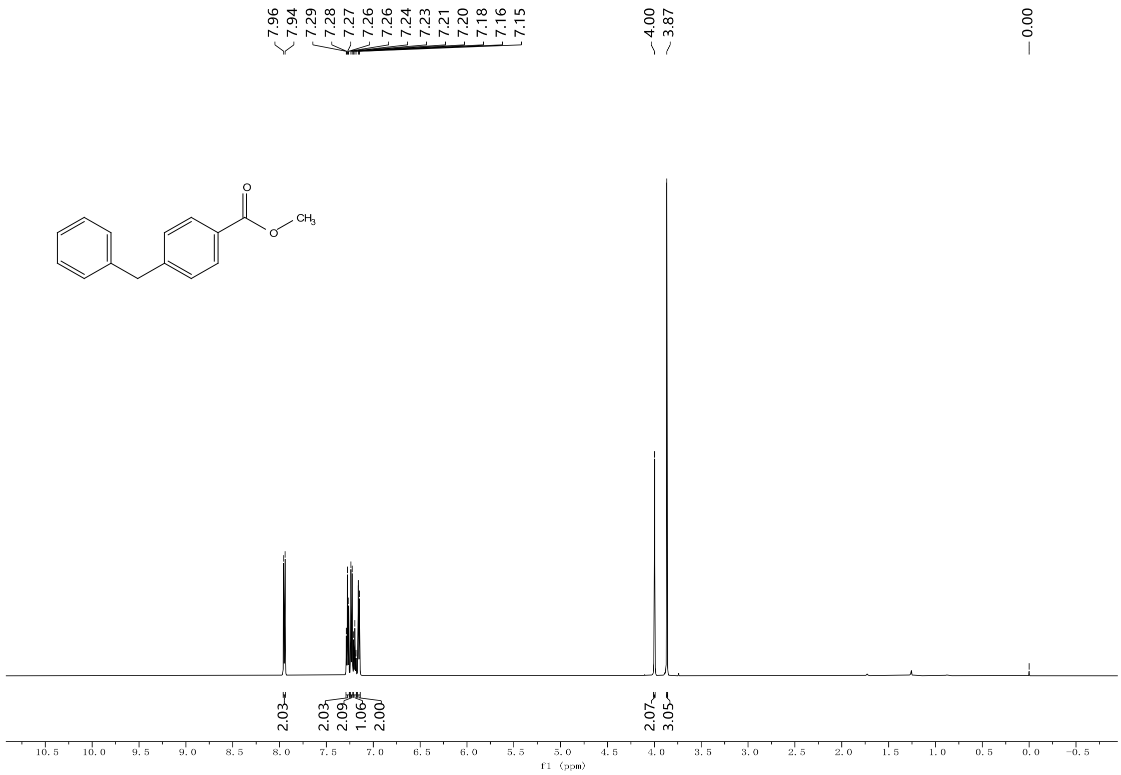

Supplementary Figure 60. ^{1}H NMR spectra of compound **15** (600 MHz, r.t., $CDCl_3$).

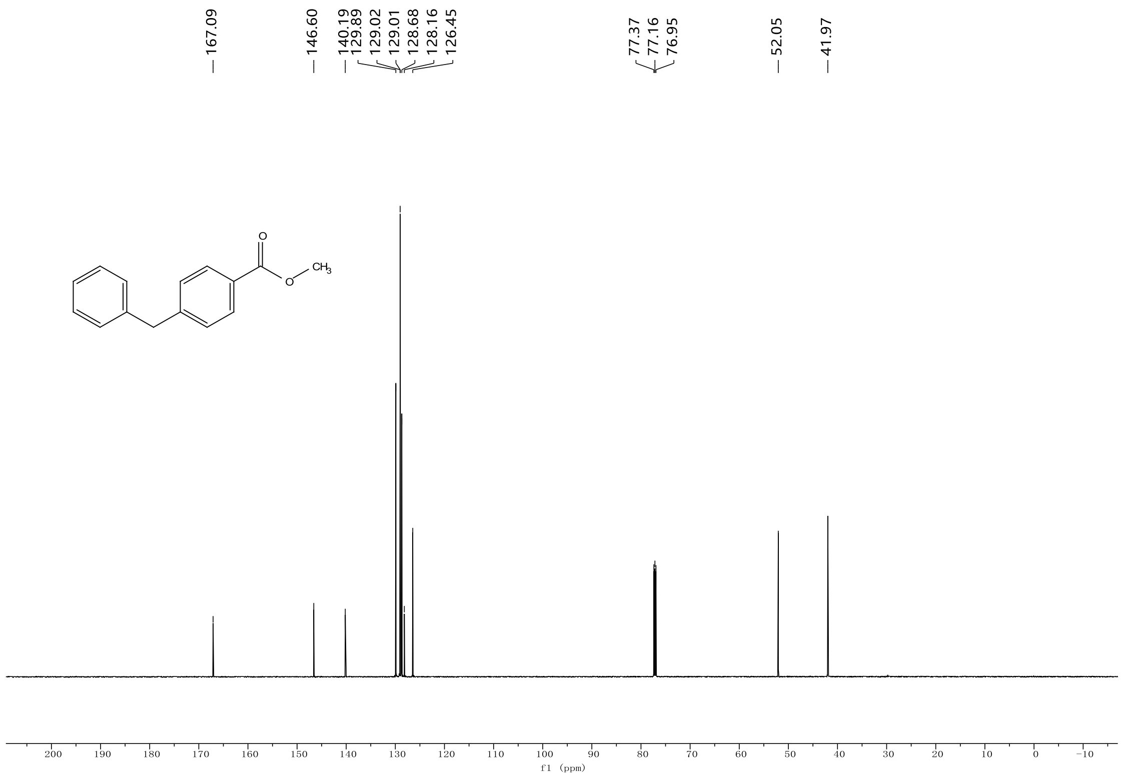

Supplementary Figure 61. ^{13}C NMR spectra of compound **15** (151 MHz, r.t., $CDCl_3$).

Compound 16

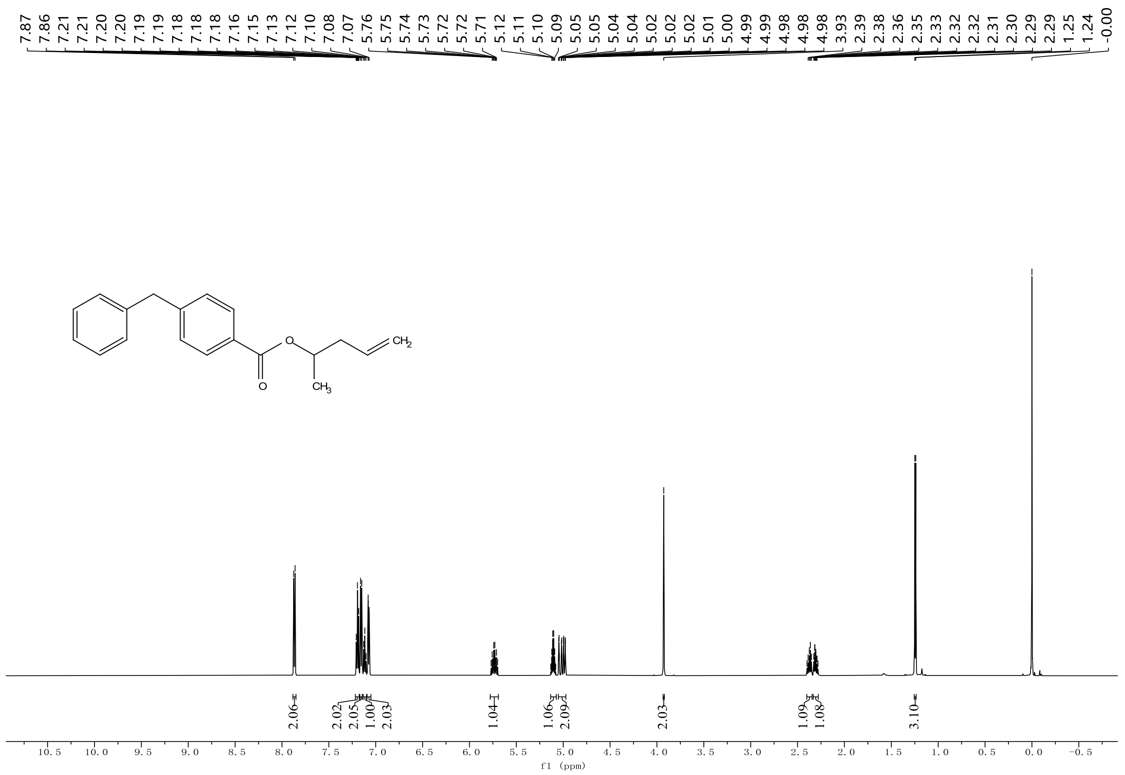

Supplementary Figure 62. ^{1}H NMR spectra of compound **16** (600 MHz, r.t., $CDCl_3$).

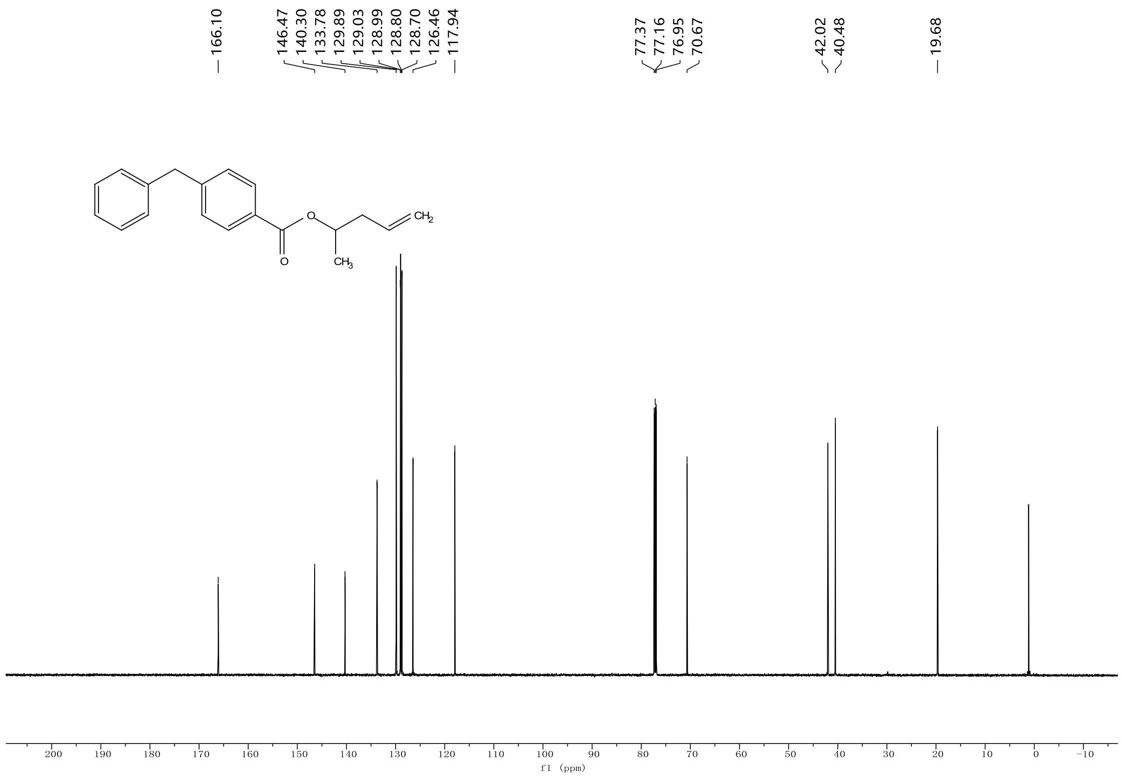

Supplementary Figure 63. ^{13}C NMR spectra of compound **16** (151 MHz, r.t., $CDCl_3$).

Compound 17

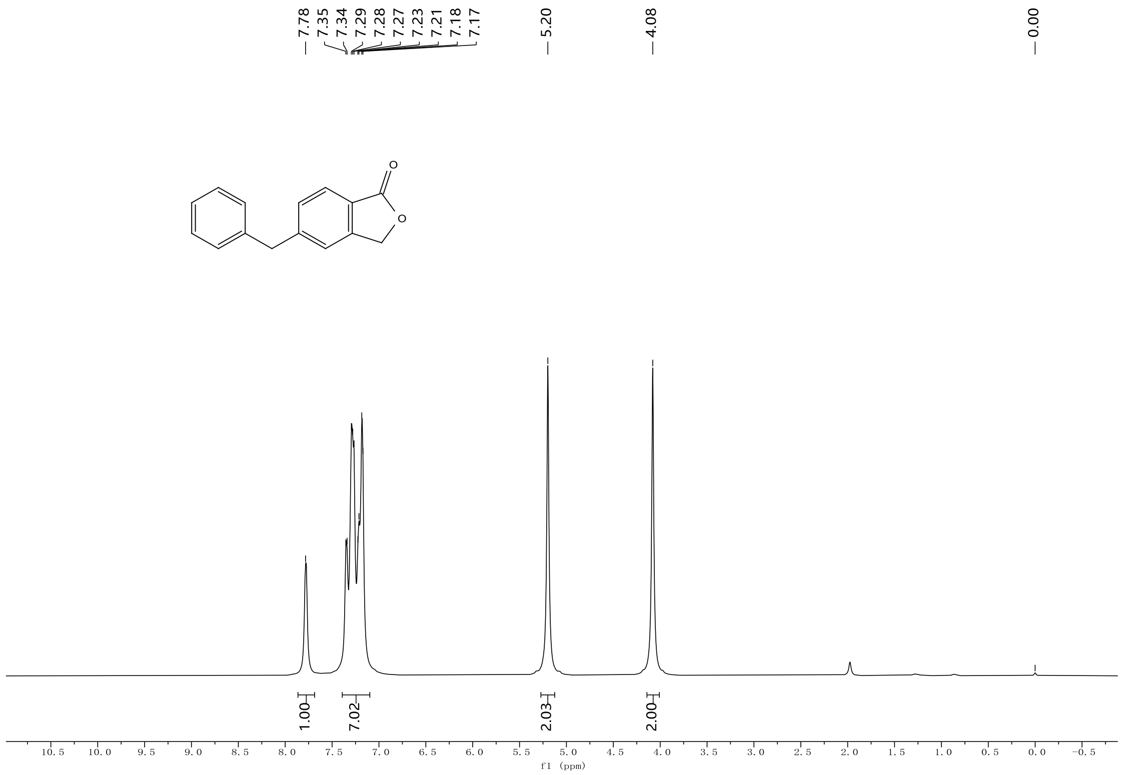

Supplementary Figure 64. ^{1}H NMR spectra of compound **17** (600 MHz, r.t., $CDCl_3$).

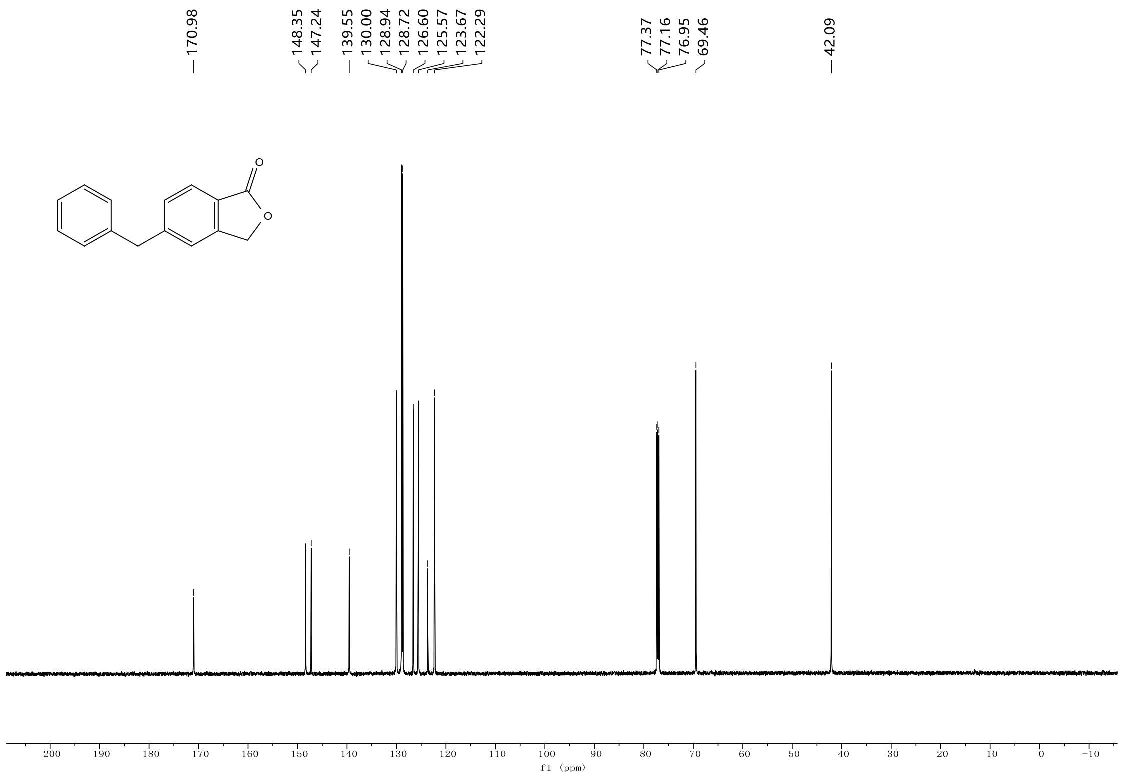

Supplementary Figure 65. ^{13}C NMR spectra of compound **17** (151 MHz, r.t., $CDCl_3$).

Compound 18

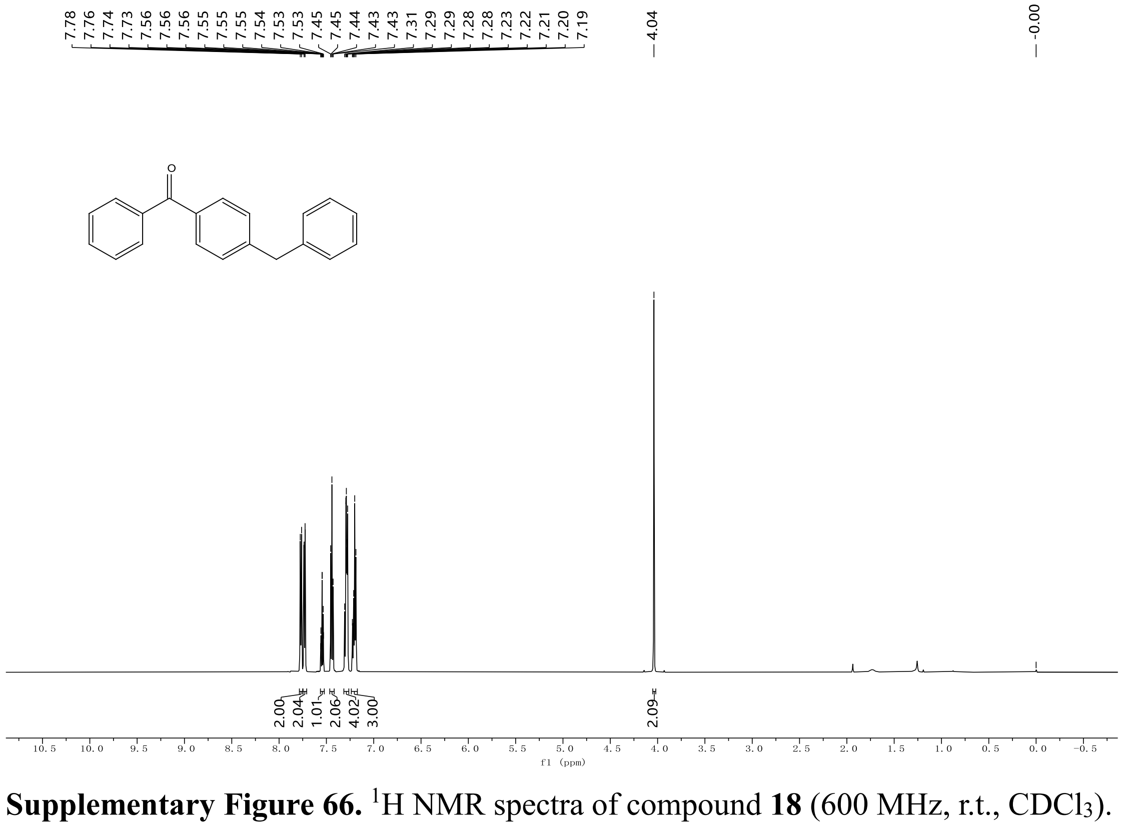

Supplementary Figure 66. ^{1}H NMR spectra of compound **18** (600 MHz, r.t., $CDCl_3$).

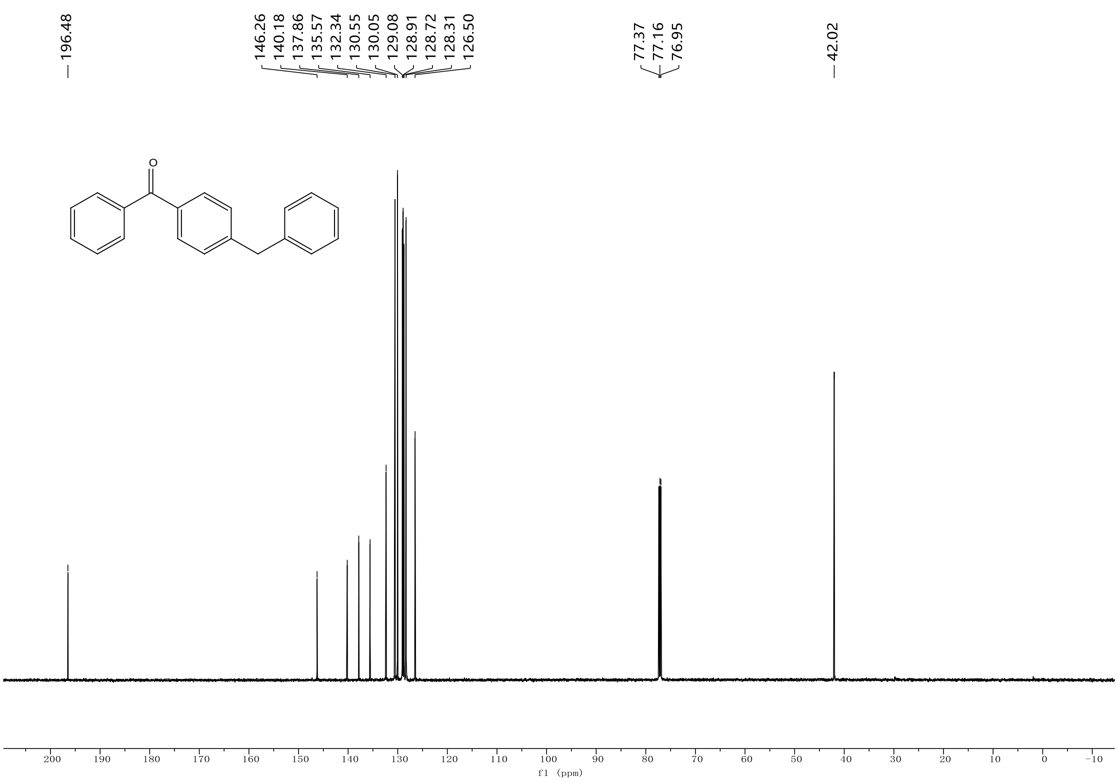

Supplementary Figure 67. ^{13}C NMR spectra of compound **18** (151 MHz, r.t., $CDCl_3$).

Compound 19

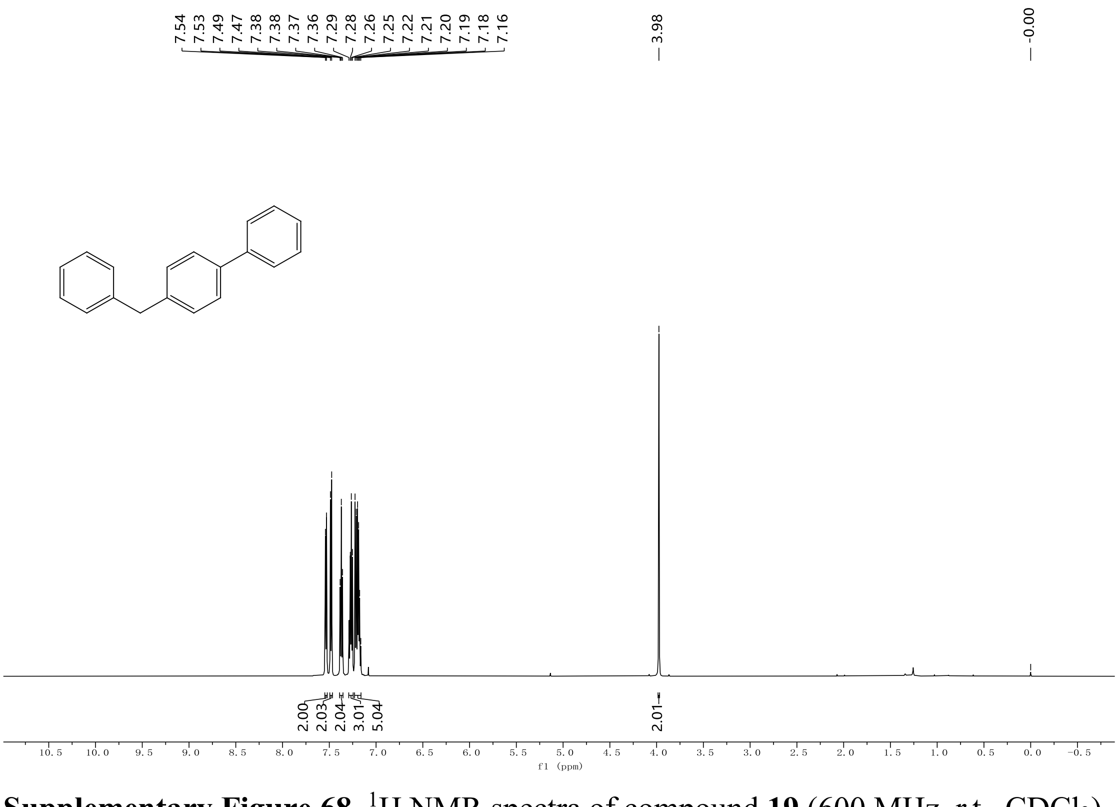

Supplementary Figure 68. ^{1}H NMR spectra of compound **19** (600 MHz, r.t., $CDCl_3$).

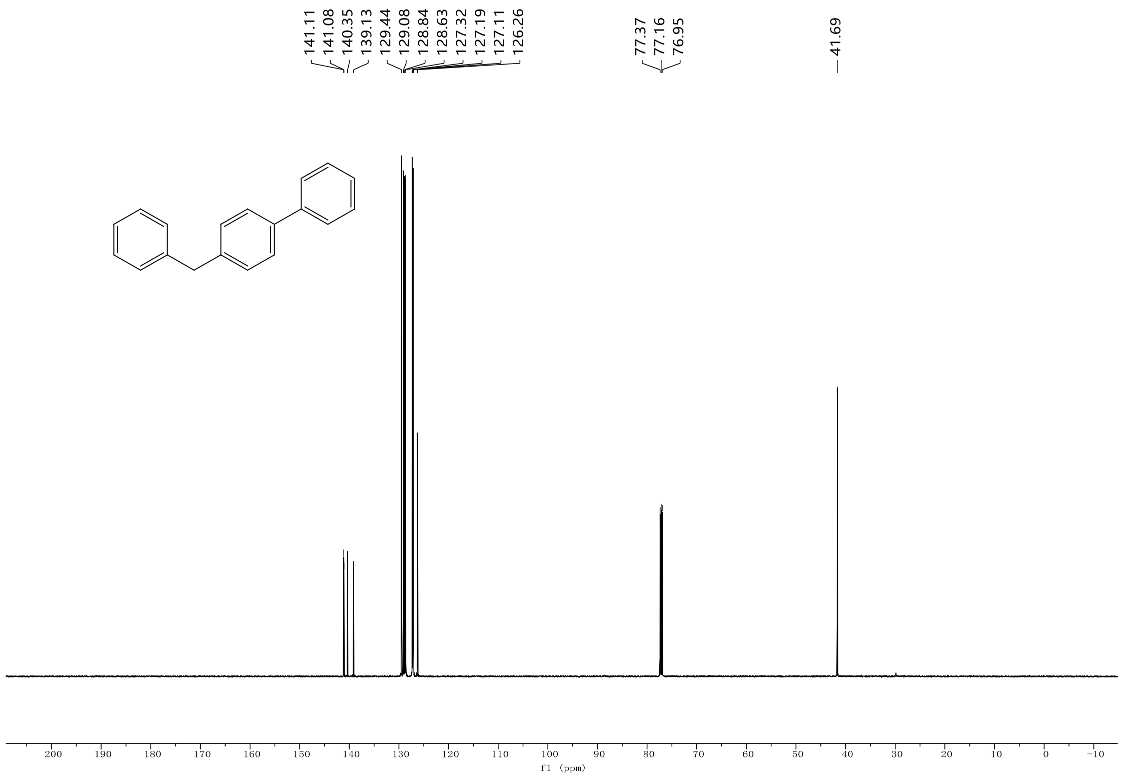

Supplementary Figure 69. ^{13}C NMR spectra of compound **19** (151 MHz, r.t., $CDCl_3$).

Compound 20

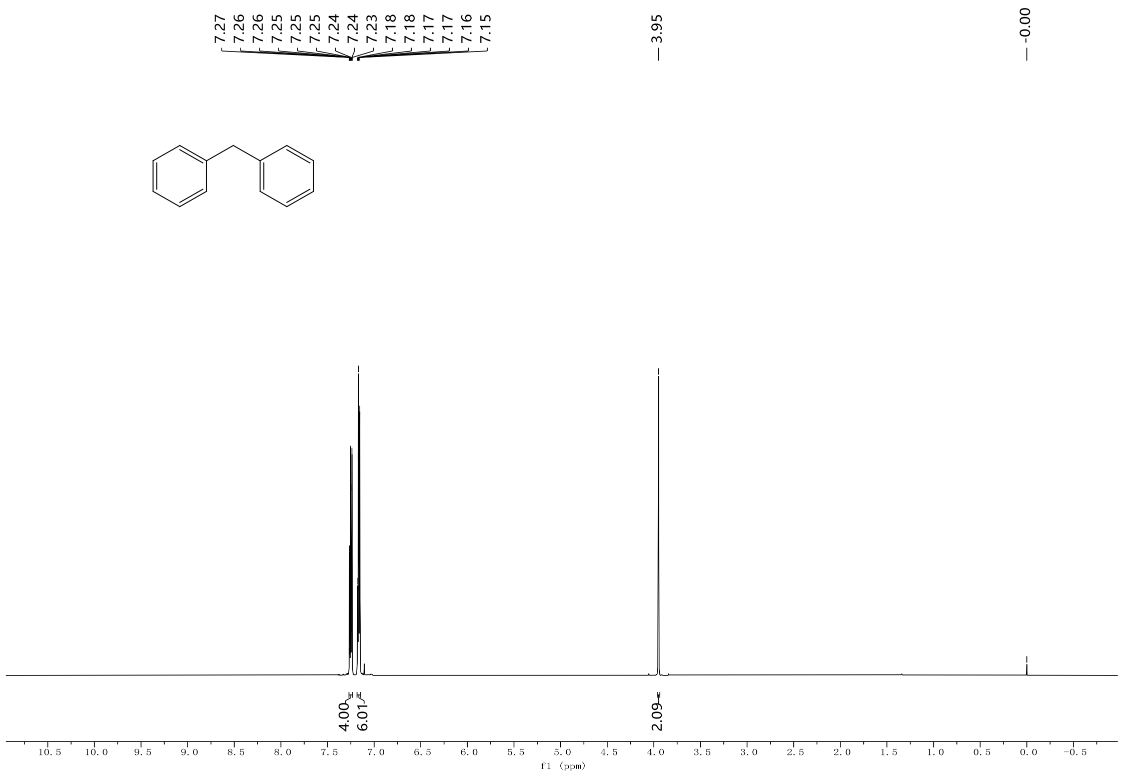

Supplementary Figure 70. ^{1}H NMR spectra of compound **20** (600 MHz, r.t., $CDCl_3$).

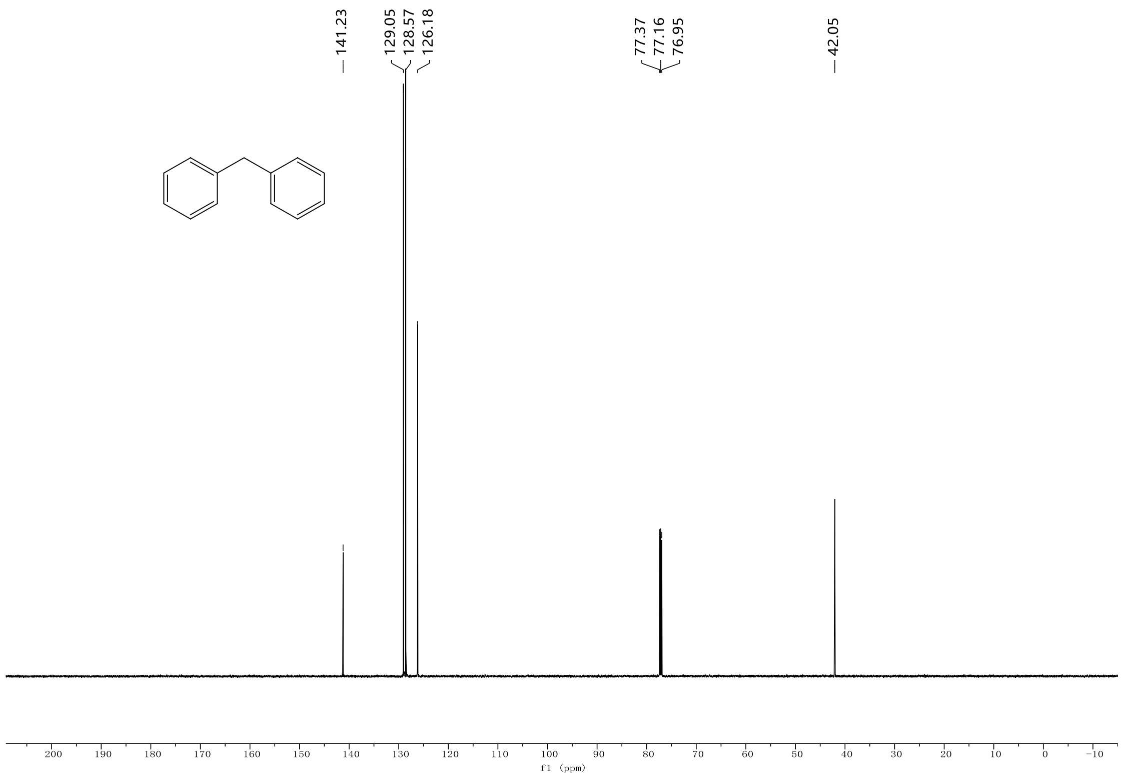

Supplementary Figure 71. ^{13}C NMR spectra of compound **20** (151 MHz, r.t., $CDCl_3$).

Compound 21

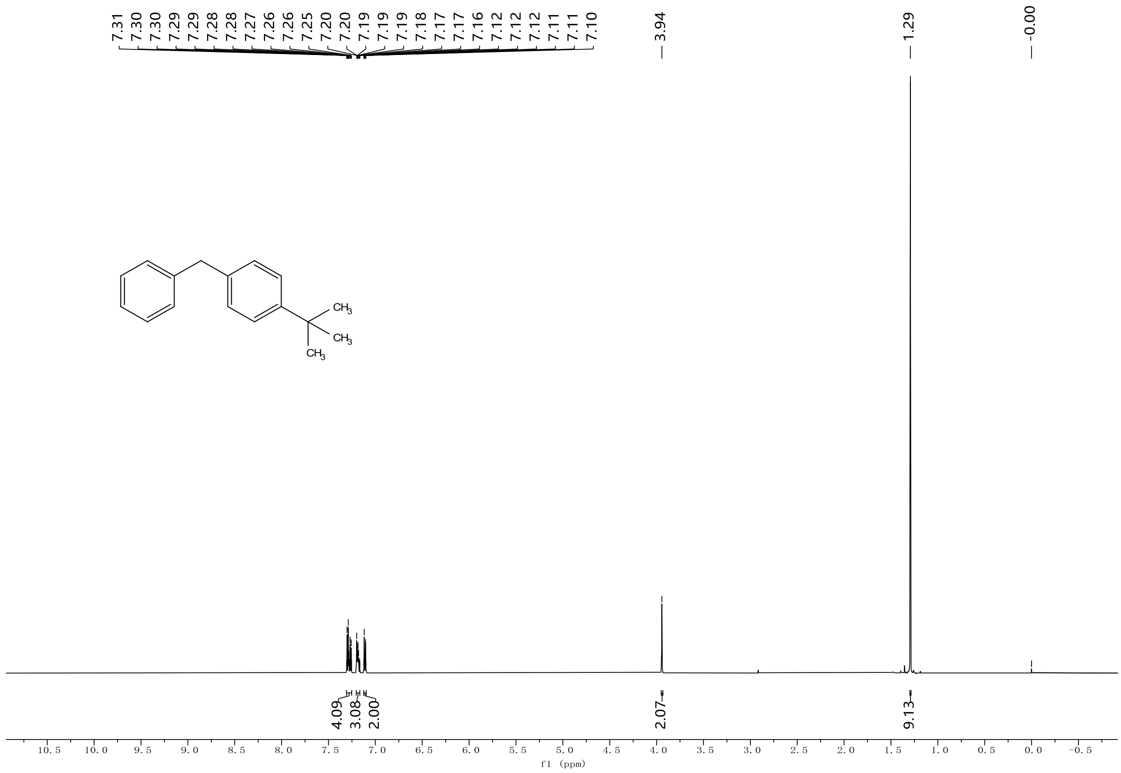

Supplementary Figure 72. ^{1}H NMR spectra of compound **21** (600 MHz, r.t., $CDCl_3$).

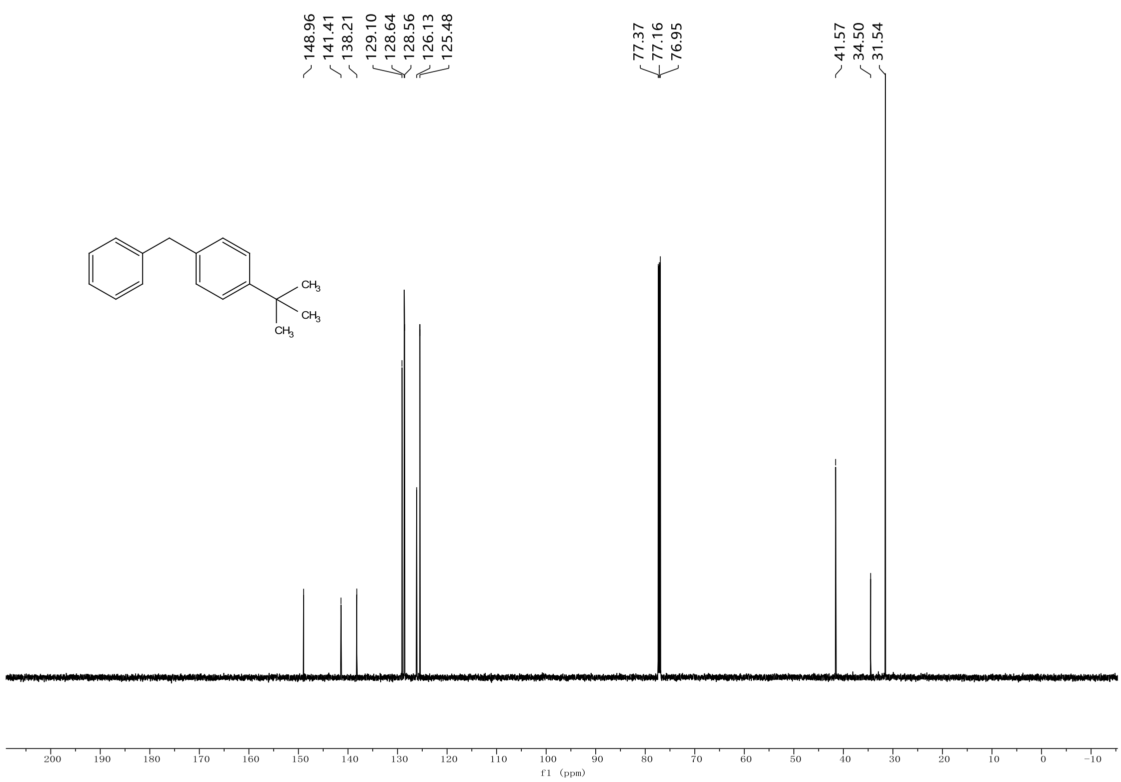

Supplementary Figure 73. ^{13}C NMR spectra of compound **21** (151 MHz, r.t., $CDCl_3$).

Compound 22

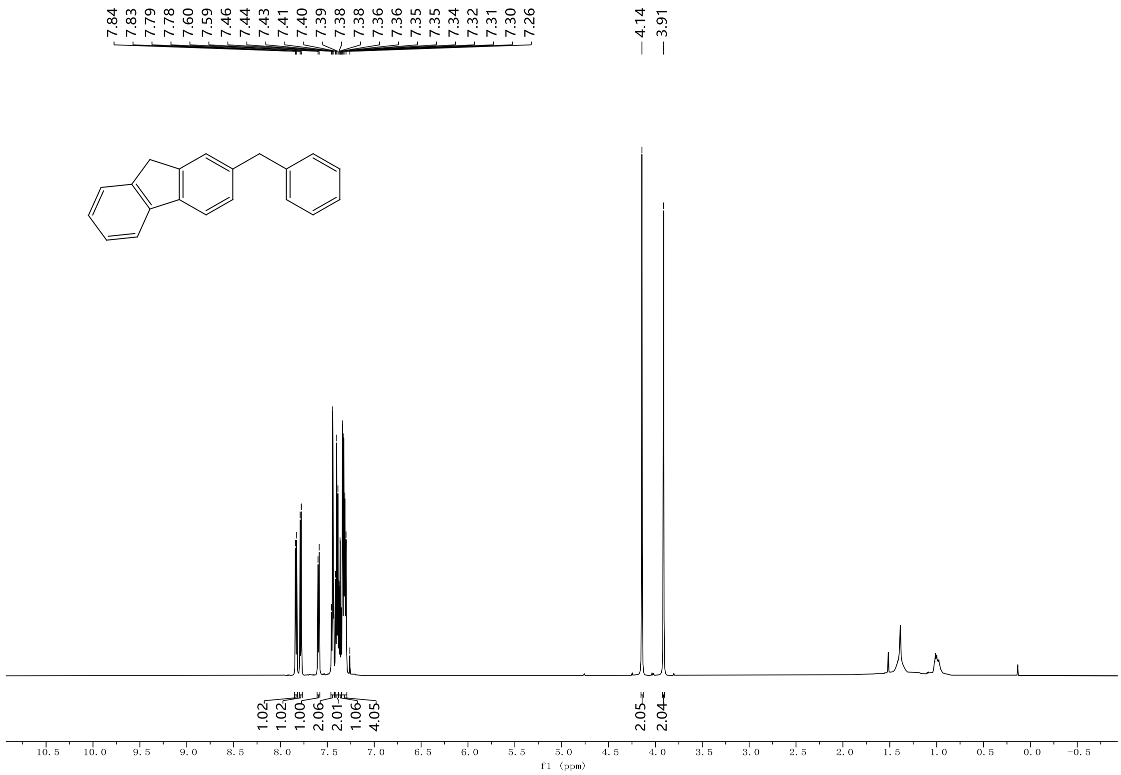

Supplementary Figure 74. ^{1}H NMR spectra of compound **22** (600 MHz, r.t., $CDCl_3$).

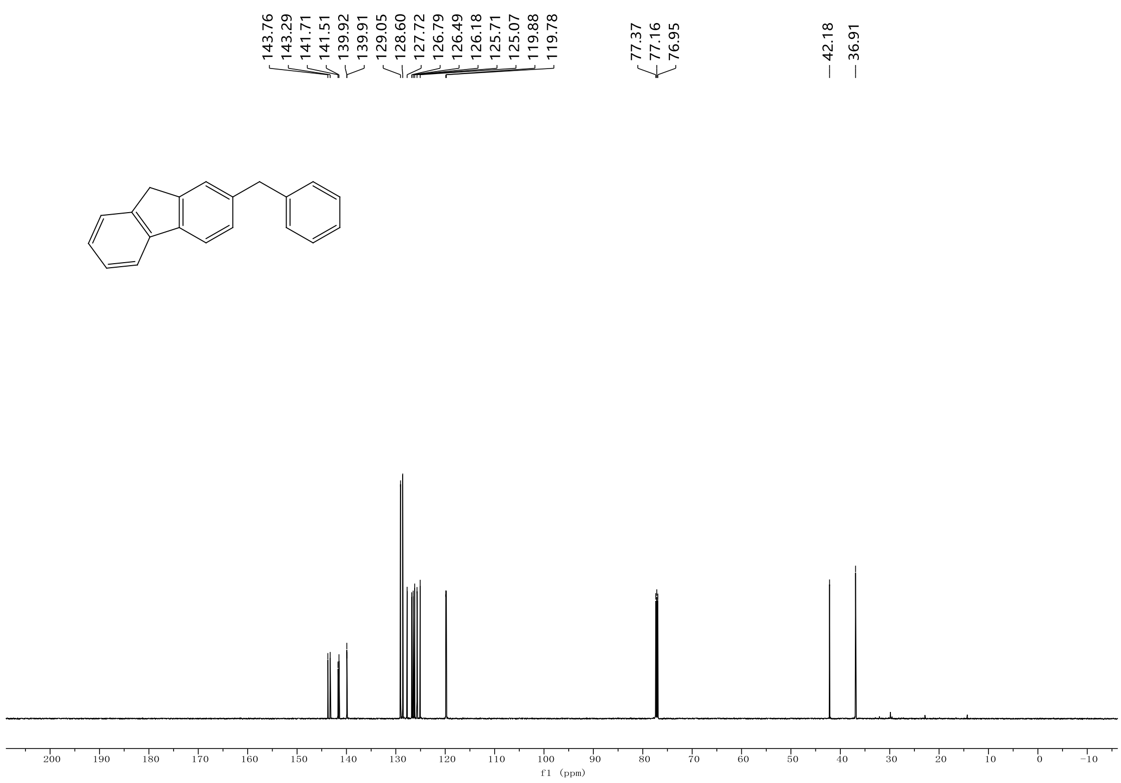

Supplementary Figure 75. ^{13}C NMR spectra of compound **22** (151 MHz, r.t., $CDCl_3$).

Compound 23

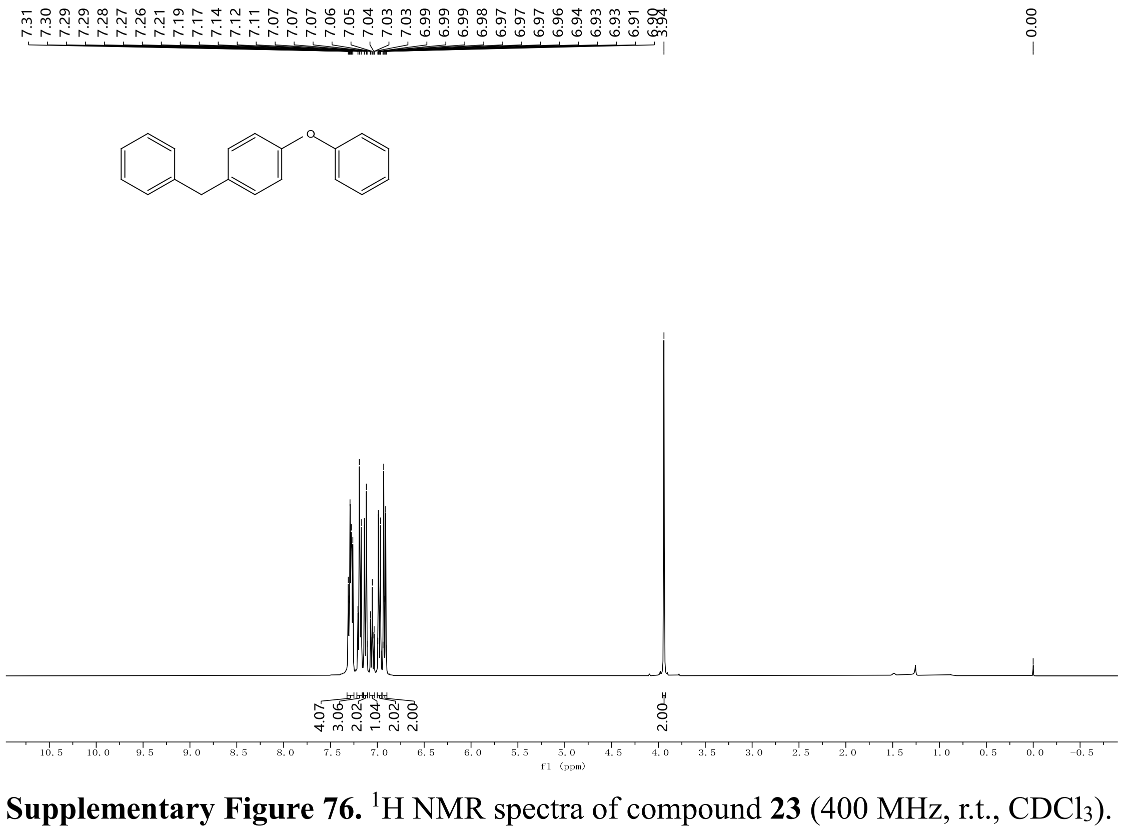

Supplementary Figure 76. ^{1}H NMR spectra of compound **23** (400 MHz, r.t., $CDCl_3$).

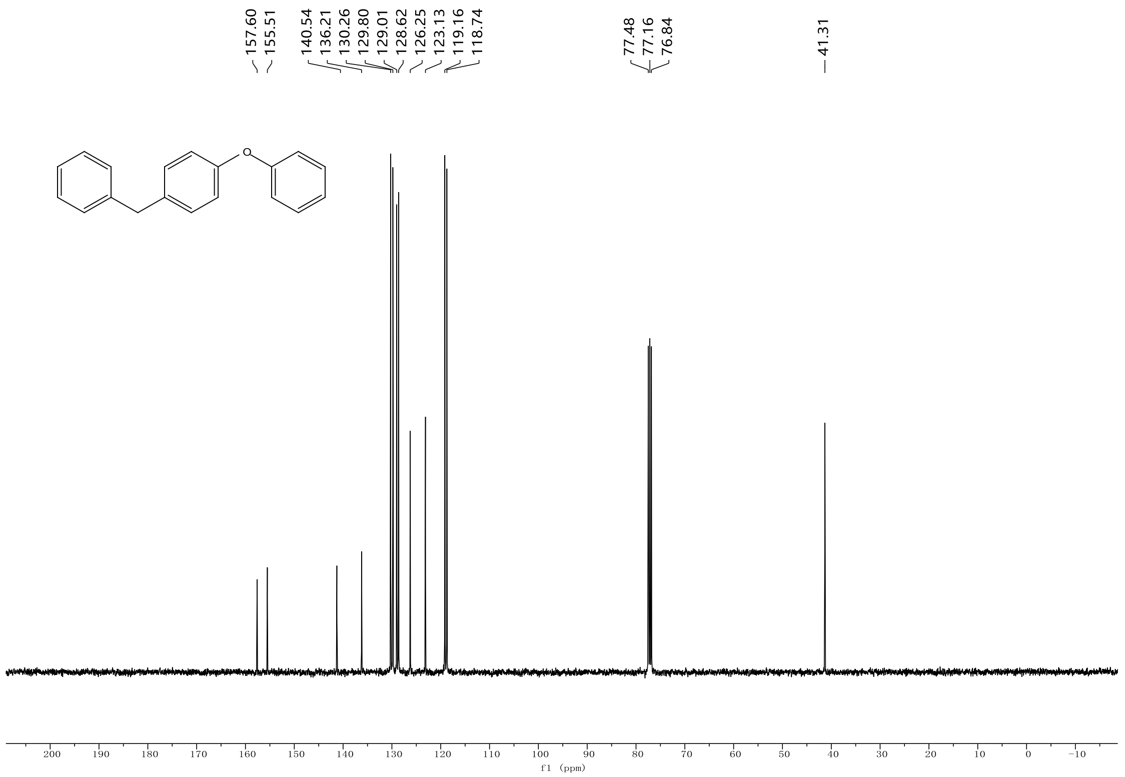

Supplementary Figure 77. ^{13}C NMR spectra of compound **23** (101 MHz, r.t., $CDCl_3$).

Compound 24

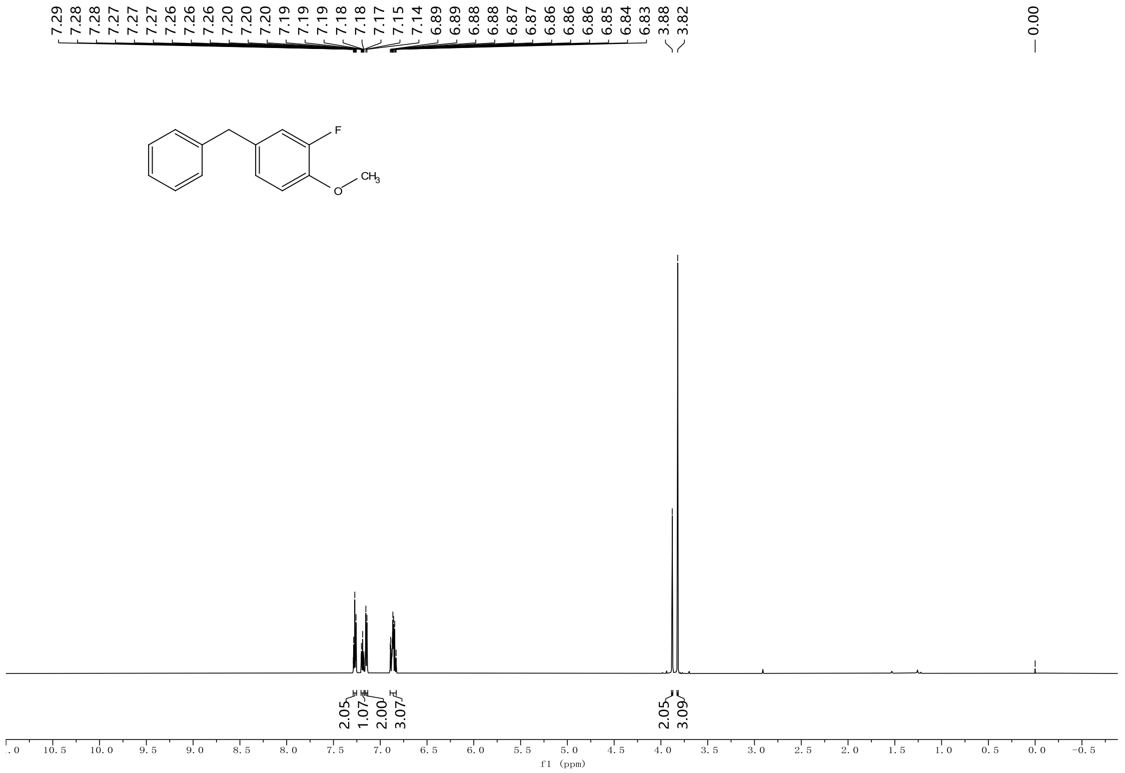

Supplementary Figure 78. ^1H NMR spectra of compound **24** (600 MHz, r.t., $CDCl_3$).

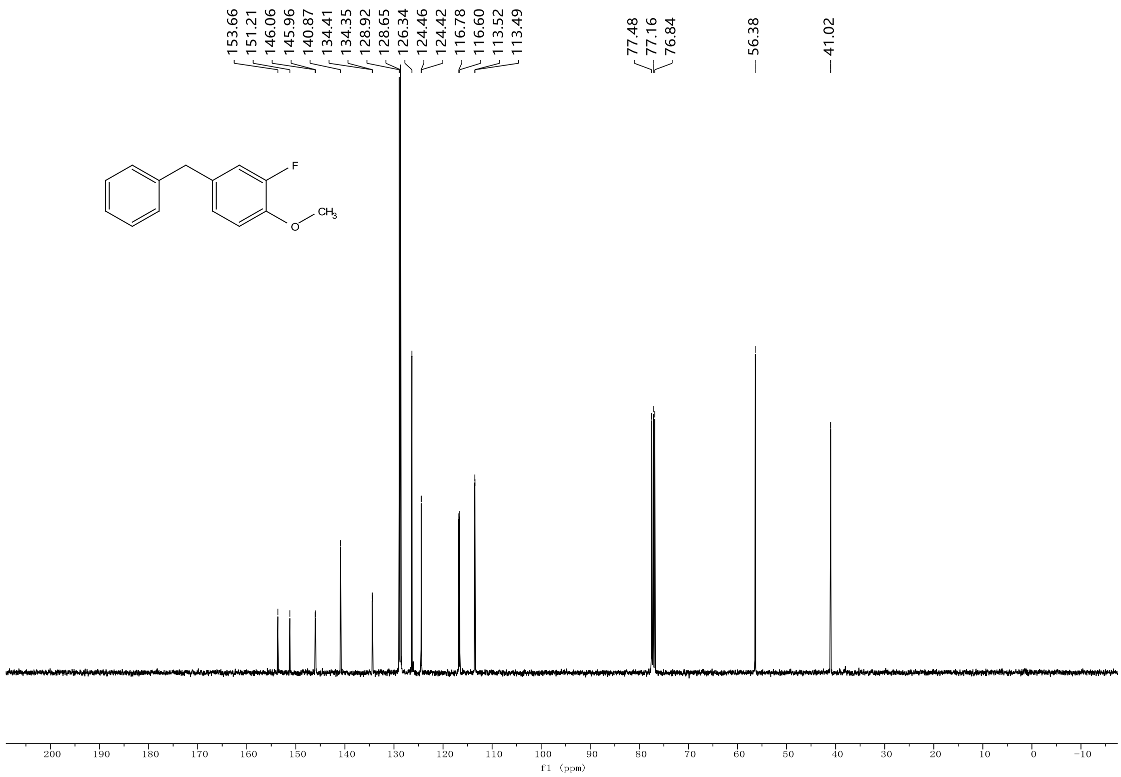

Supplementary Figure 79. ^{13}C NMR spectra of compound **24** (101 MHz, r.t., $CDCl_3$).

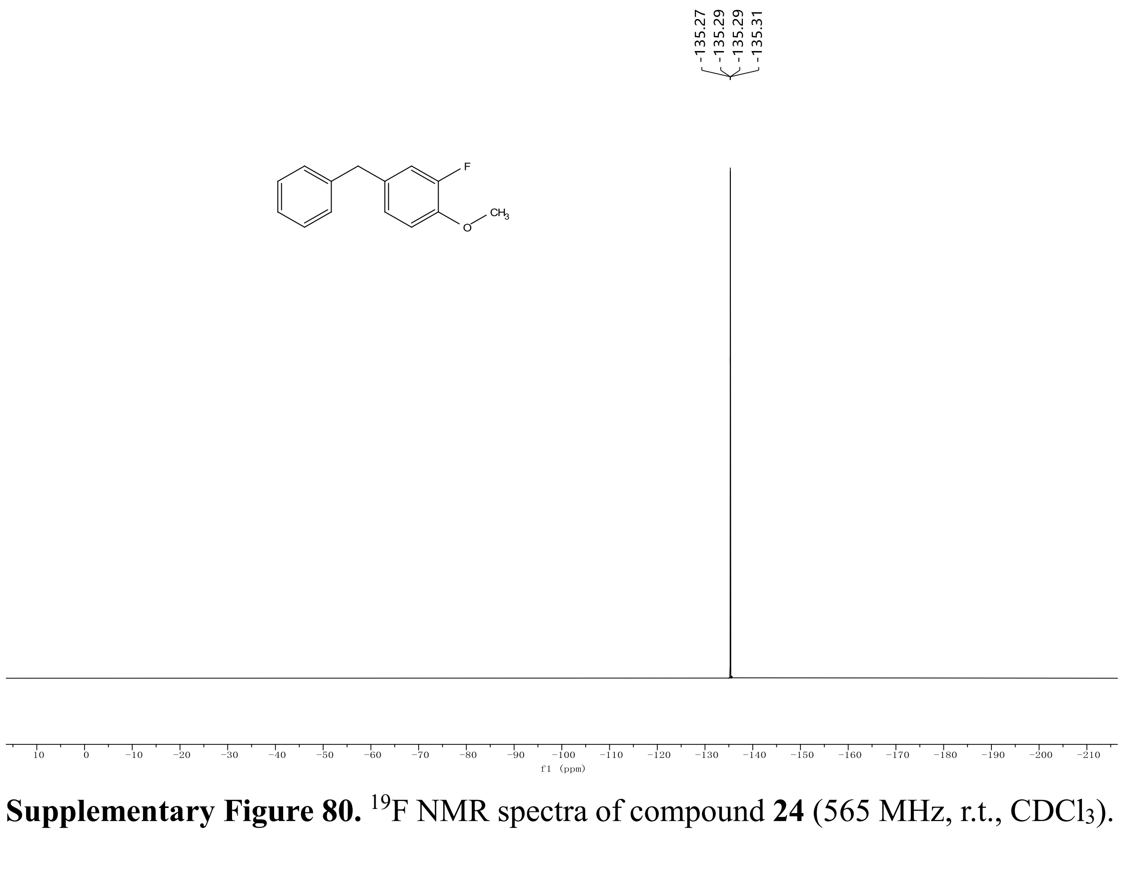

Supplementary Figure 80. ^{19}F NMR spectra of compound **24** (565 MHz, r.t., $CDCl_3$).

Compound 25

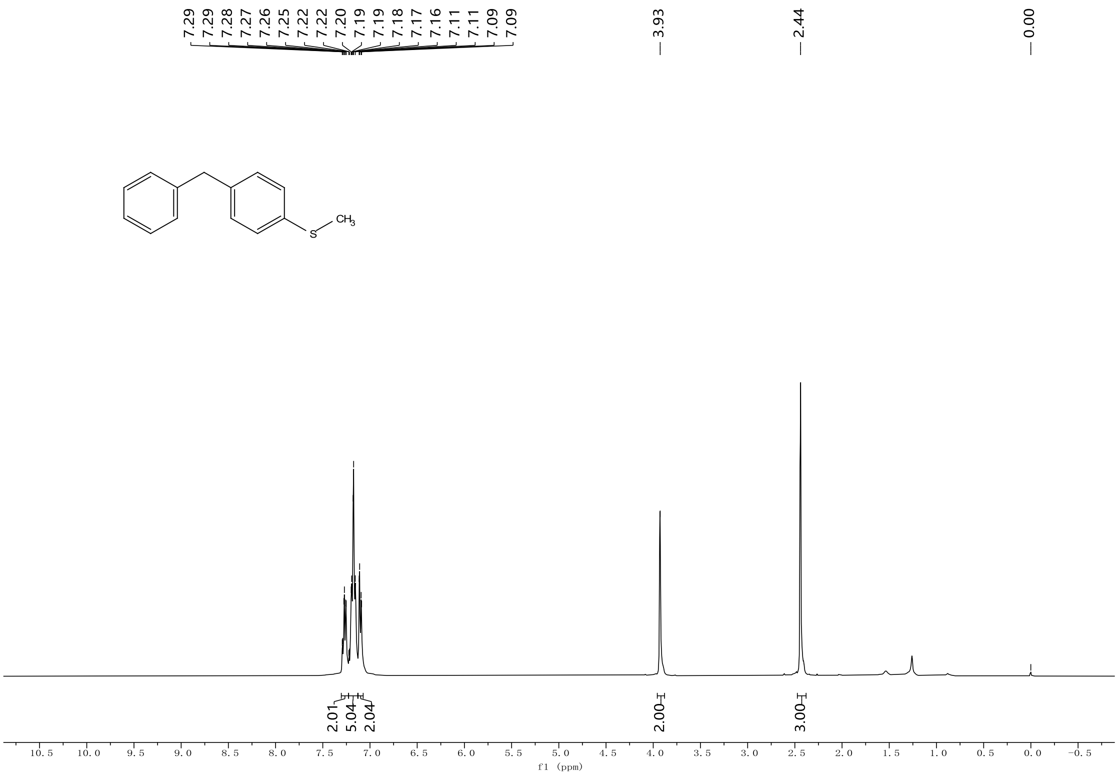

Supplementary Figure 81. ^{1}H NMR spectra of compound **25** (400 MHz, r.t., $CDCl_3$).

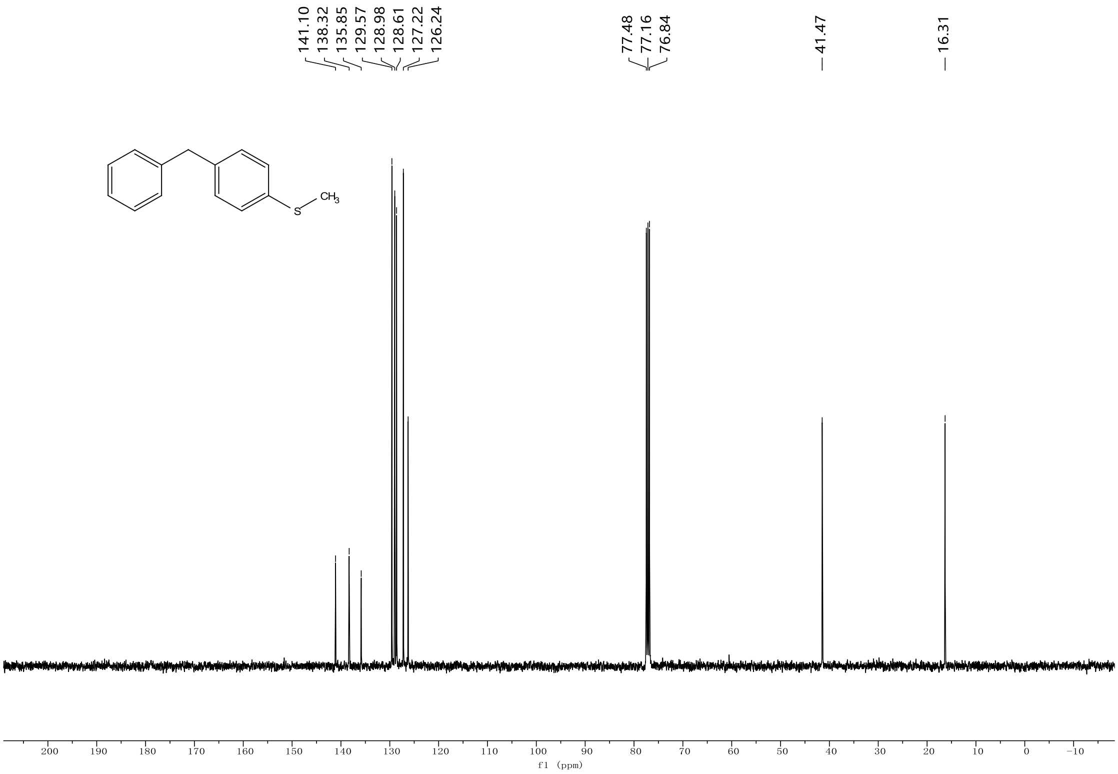

Supplementary Figure 82. ^{13}C NMR spectra of compound **25** (101 MHz, r.t., $CDCl_3$).

Compound 26

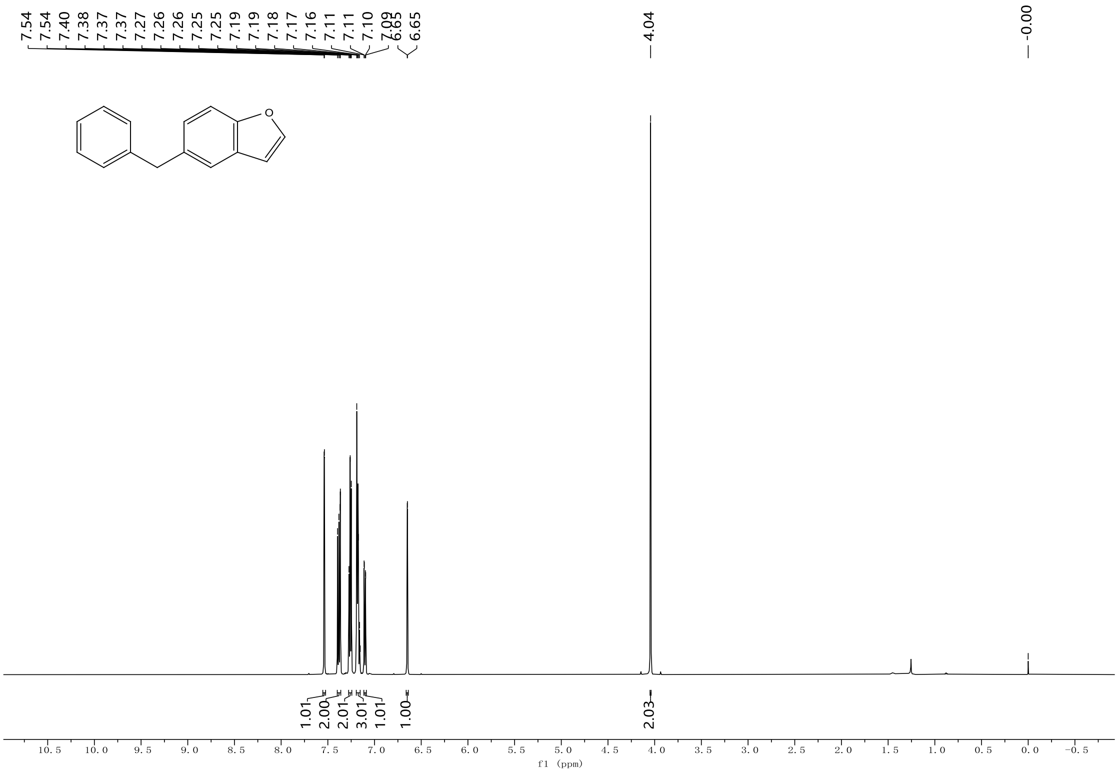

Supplementary Figure 83. ^{1}H NMR spectra of compound **26** (600 MHz, r.t., $CDCl_3$).

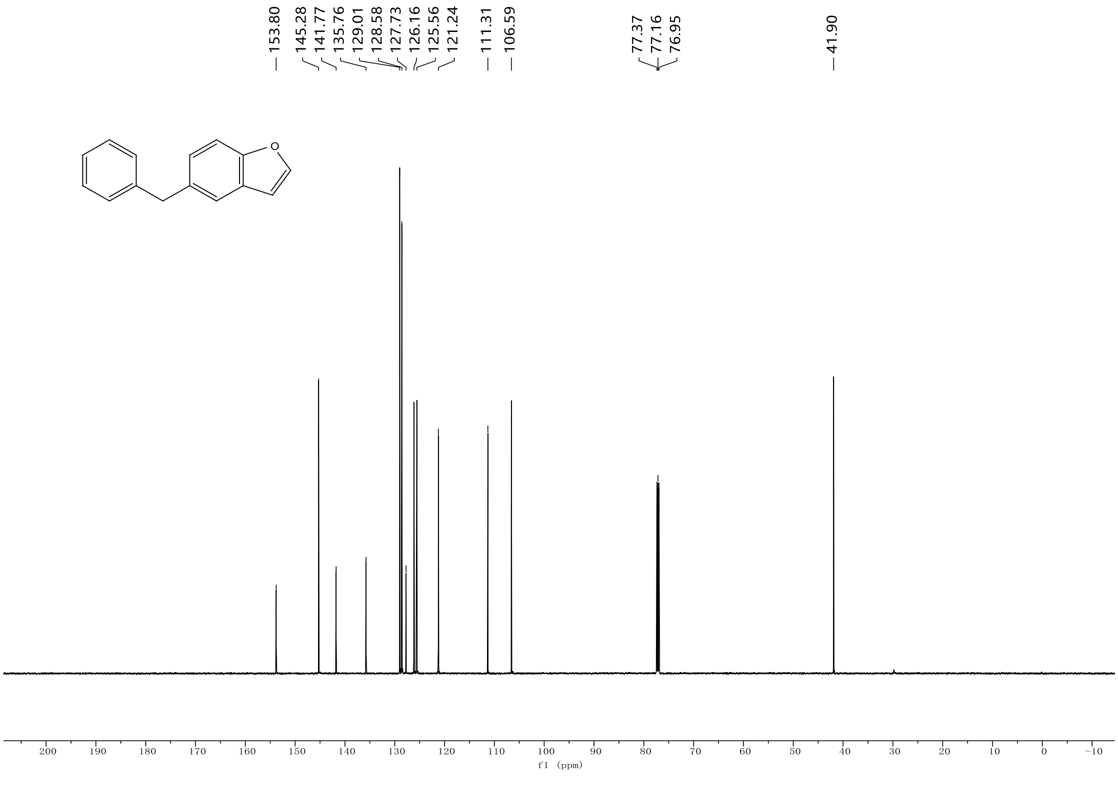

Supplementary Figure 84. ^{13}C NMR spectra of compound **26** (151 MHz, r.t., $CDCl_3$).

Compound 27

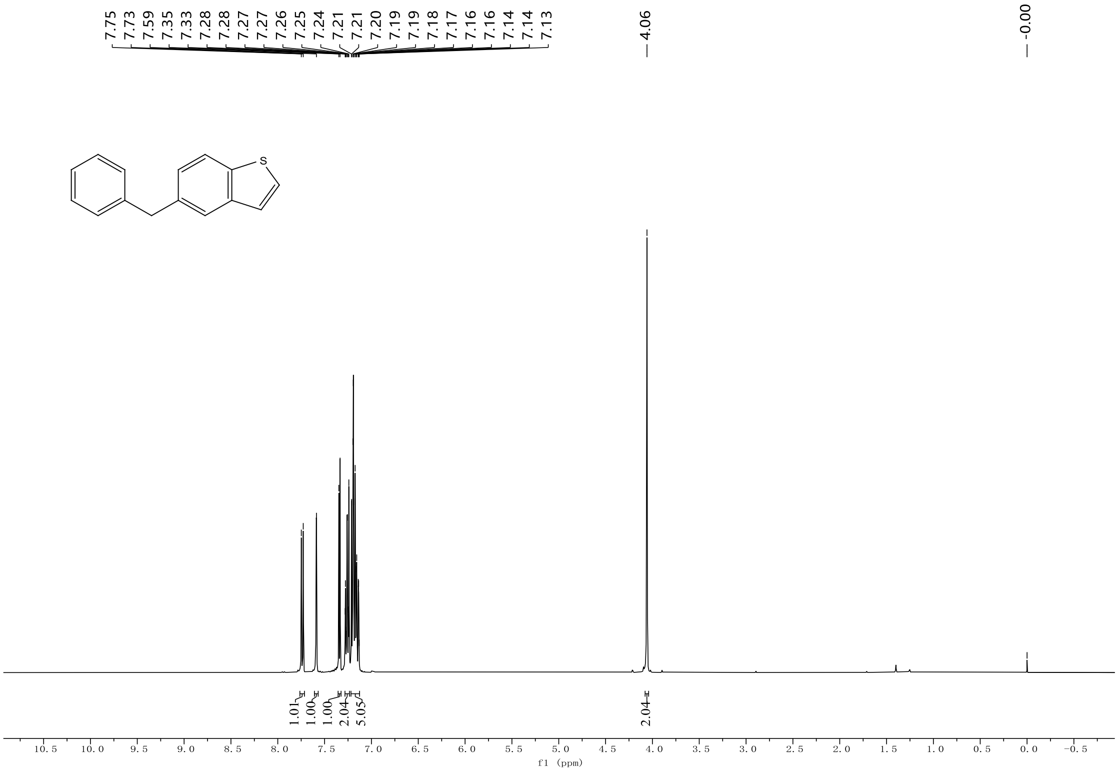

Supplementary Figure 85. ^{1}H NMR spectra of compound **27** (400 MHz, r.t., $CDCl_3$).

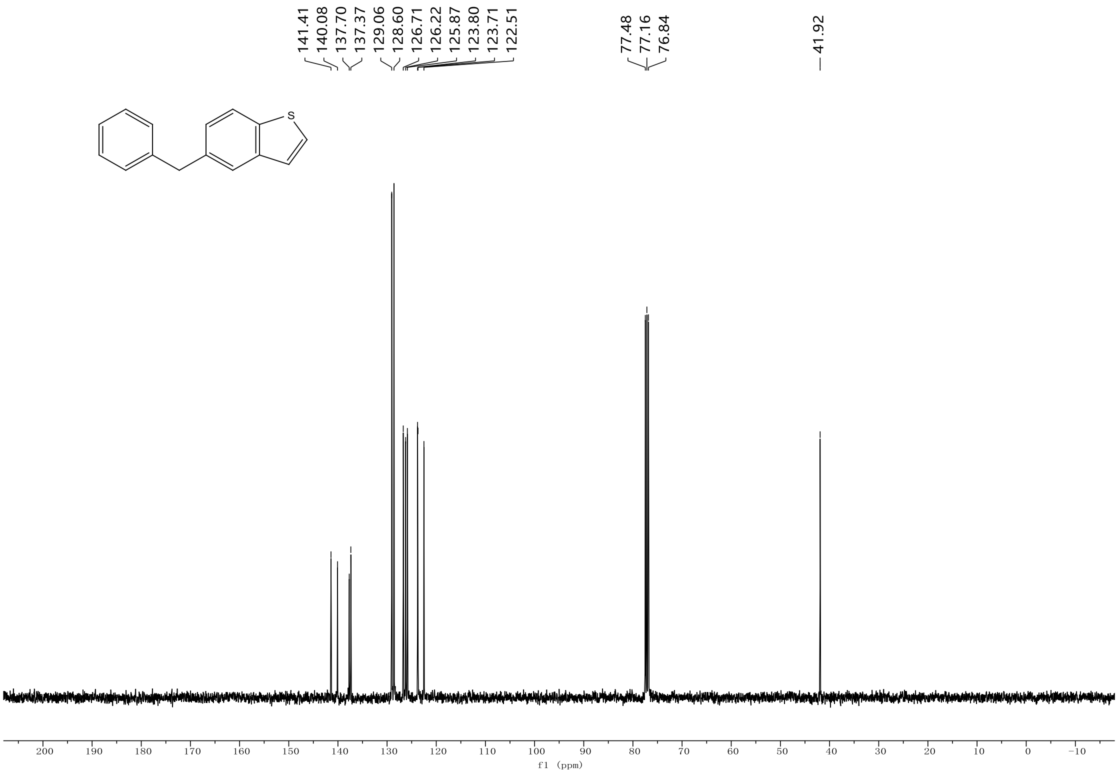

Supplementary Figure 86. ^{13}C NMR spectra of compound **27** (101 MHz, r.t., $CDCl_3$).

Compound 28

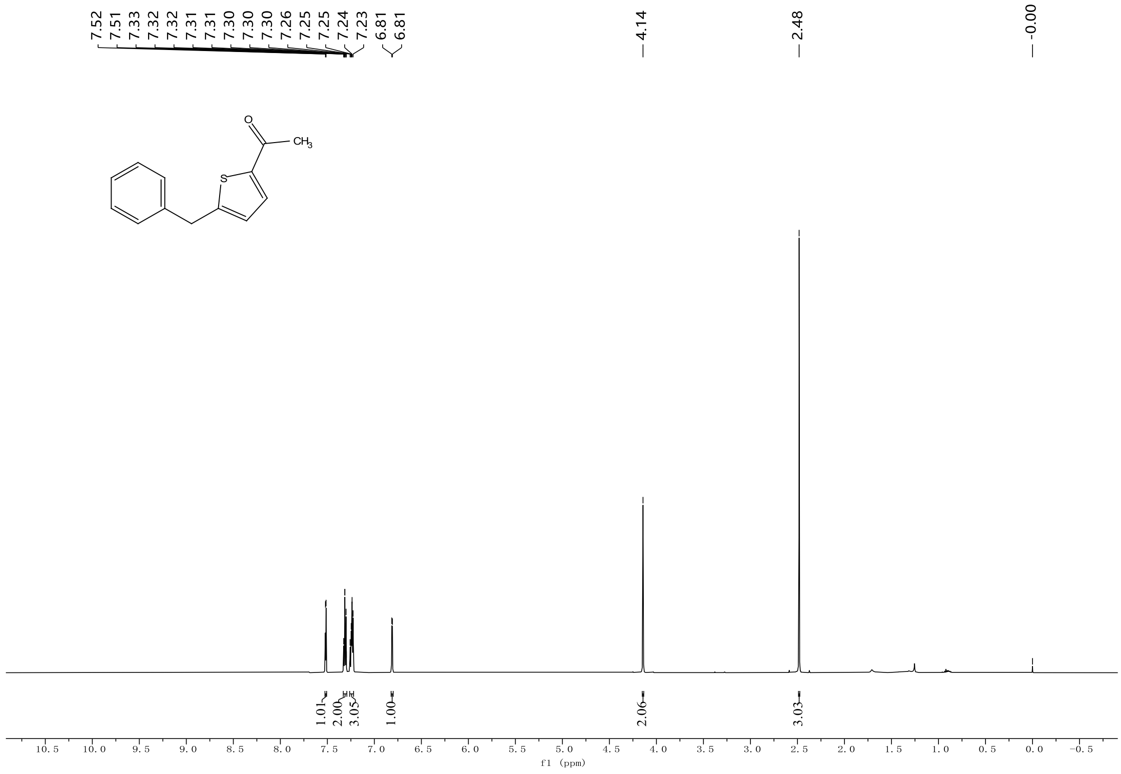

Supplementary Figure 87. ^{1}H NMR spectra of compound **28** (600 MHz, r.t., $CDCl_3$).

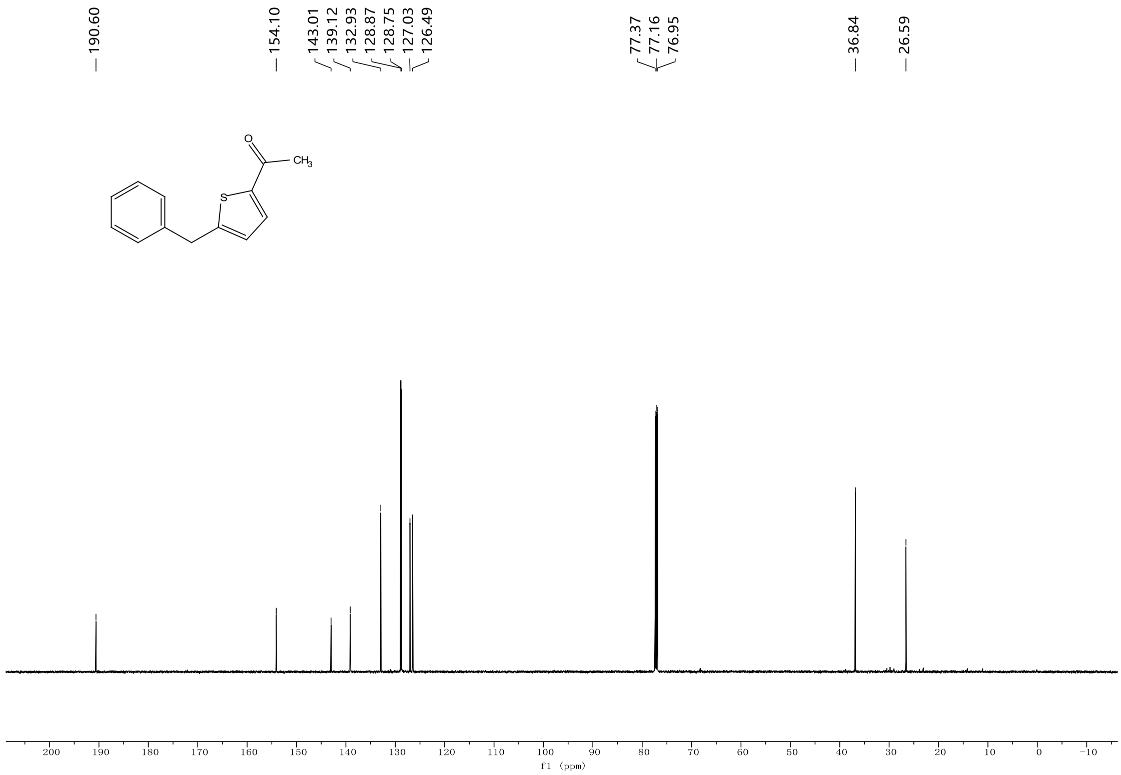

Supplementary Figure 88. ^{13}C NMR spectra of compound **28** (151 MHz, r.t., $CDCl_3$).

Compound 29

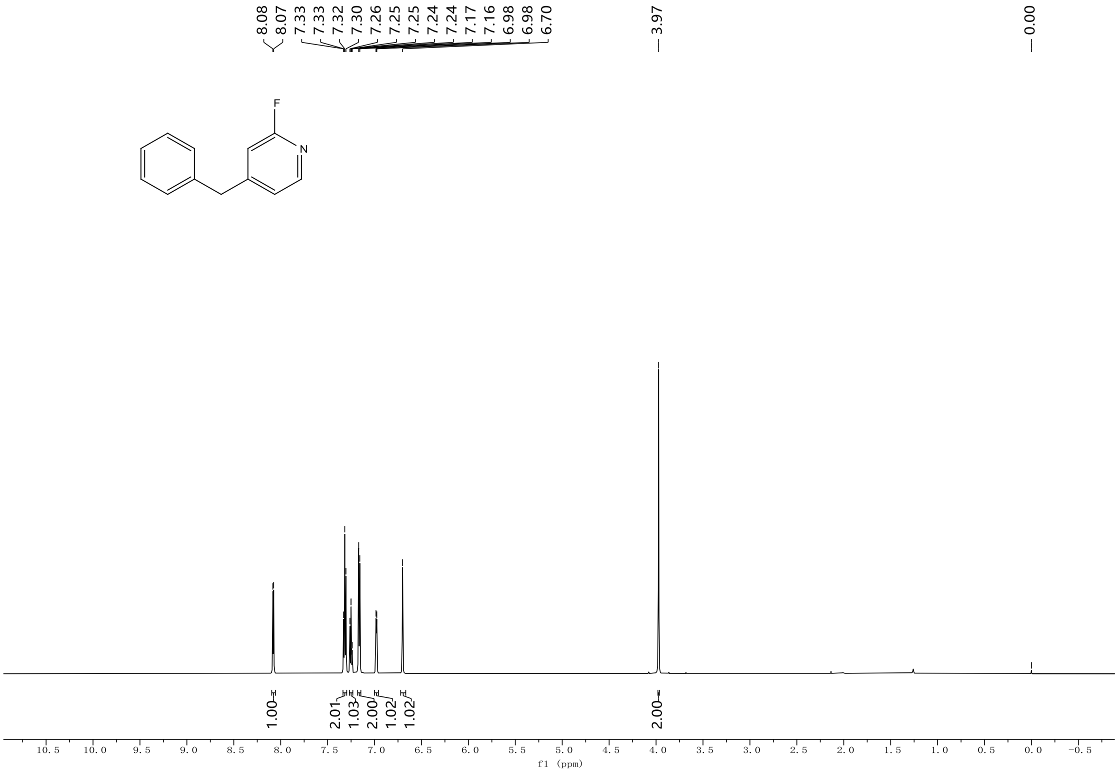

Supplementary Figure 89. ^{1}H NMR spectra of compound **29** (600 MHz, r.t., $CDCl_3$).

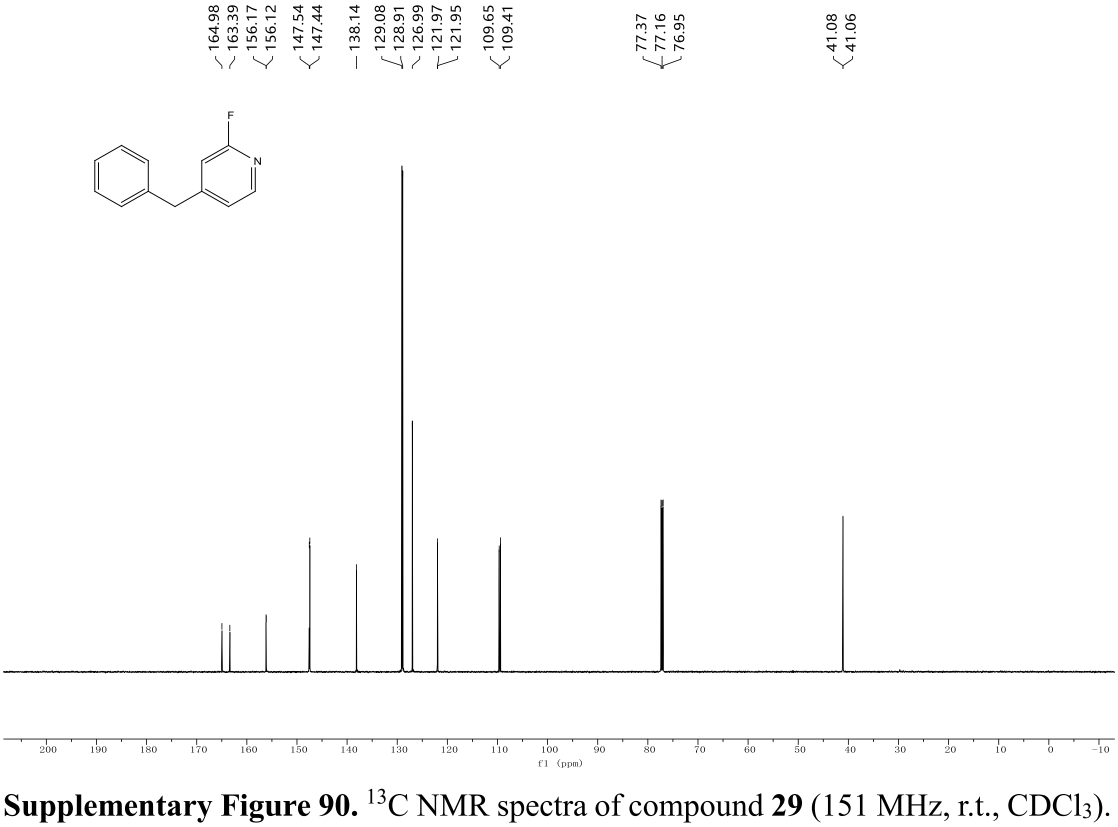

Supplementary Figure 90. ^{13}C NMR spectra of compound **29** (151 MHz, r.t., $CDCl_3$).

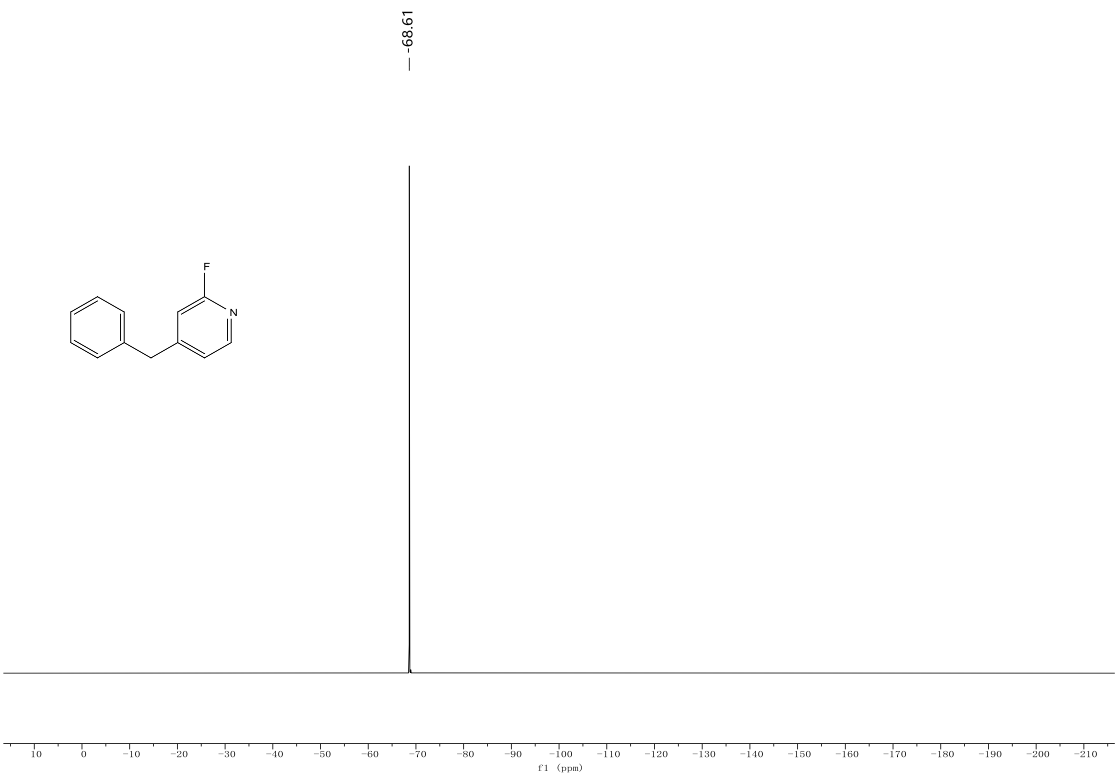

Supplementary Figure 91. ^{19}F NMR spectra of compound **29** (565 MHz, r.t., $CDCl_3$).

Compound 30

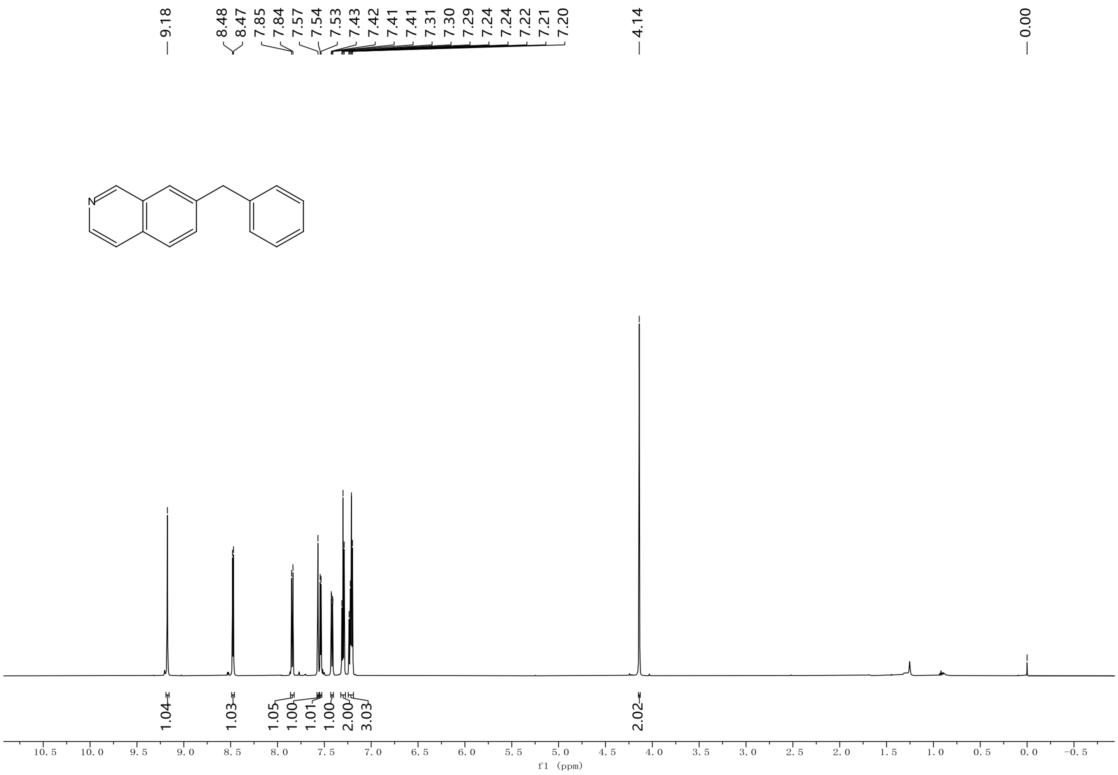

Supplementary Figure 92. ^{1}H NMR spectra of compound **30** (600 MHz, r.t., $CDCl_3$).

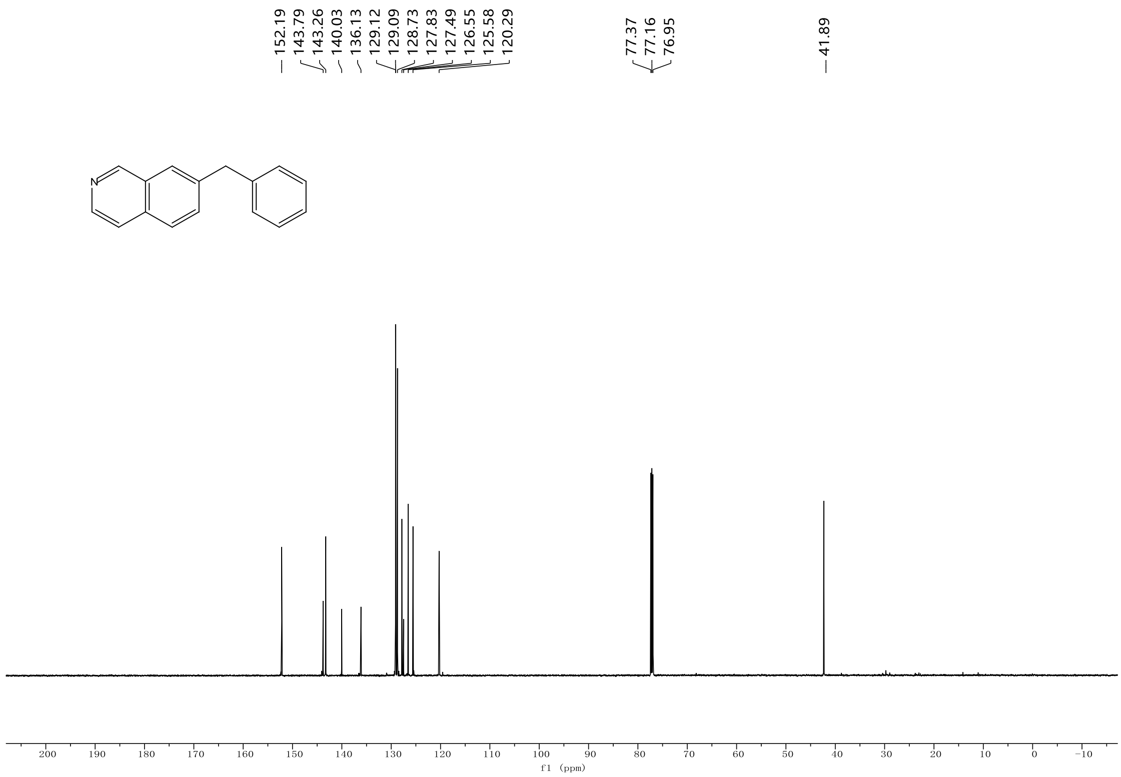

Supplementary Figure 93. ^{13}C NMR spectra of compound **30** (151 MHz, r.t., $CDCl_3$).

Compound 31

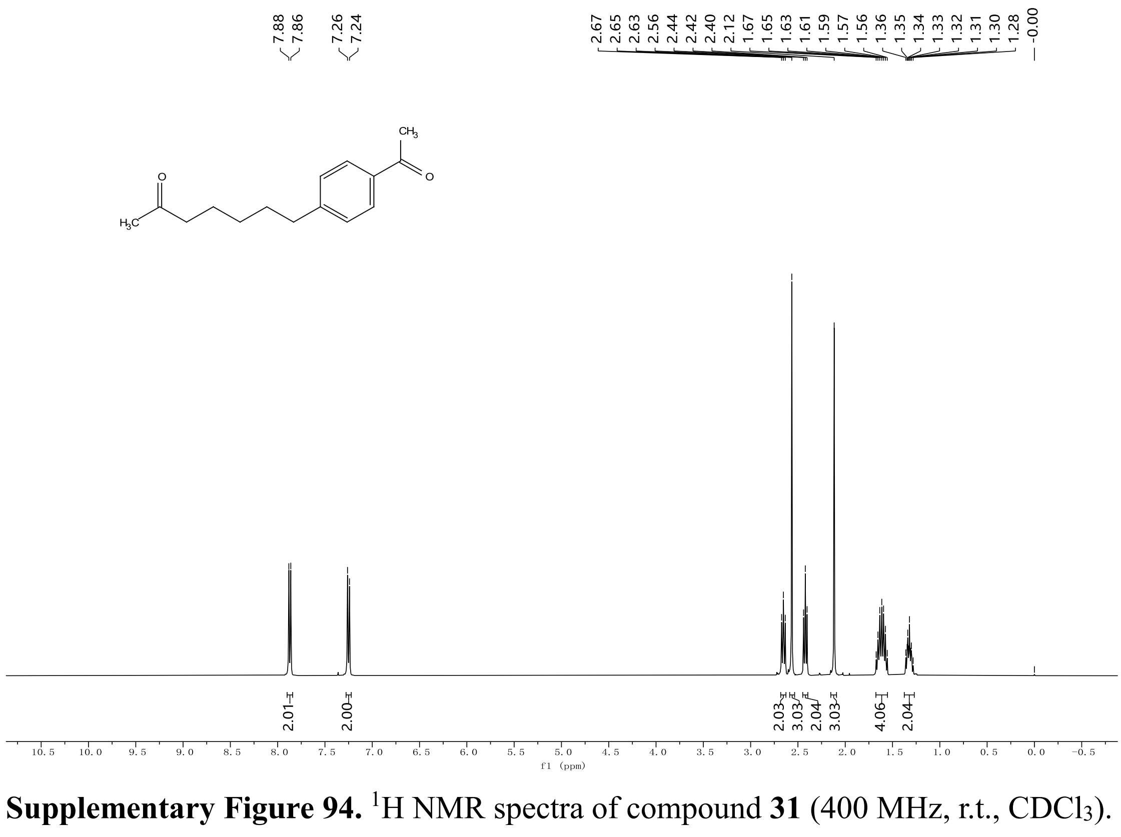

Supplementary Figure 94. ^{1}H NMR spectra of compound **31** (400 MHz, r.t., $CDCl_3$).

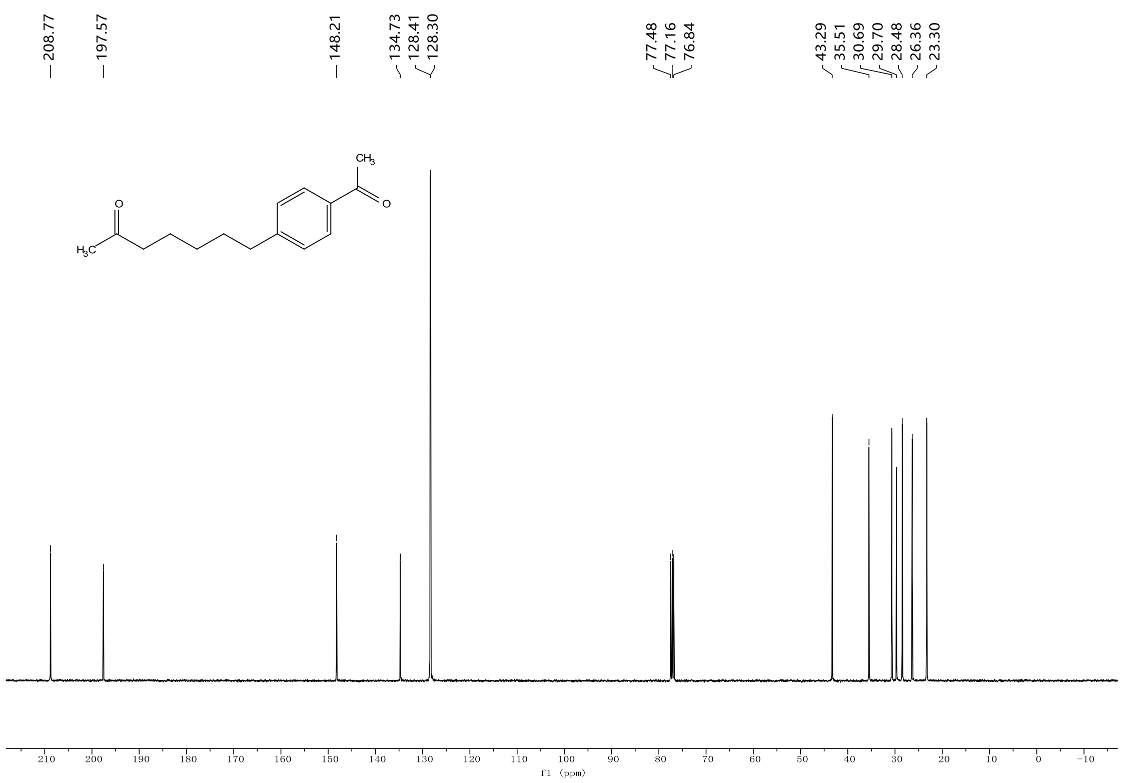

Supplementary Figure 95. ^{13}C NMR spectra of compound **31** (101 MHz, r.t., $CDCl_3$).

Compound 32

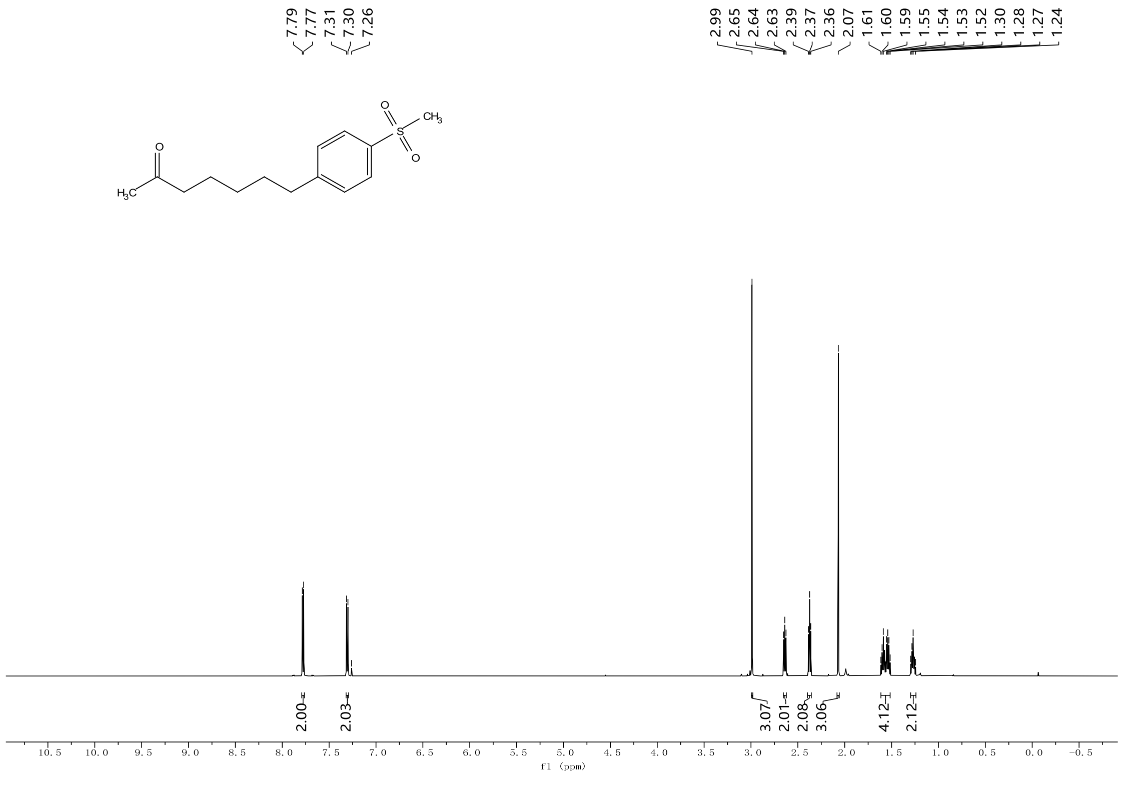

Supplementary Figure 96. ^{1}H NMR spectra of compound **32** (600 MHz, r.t., $CDCl_3$).

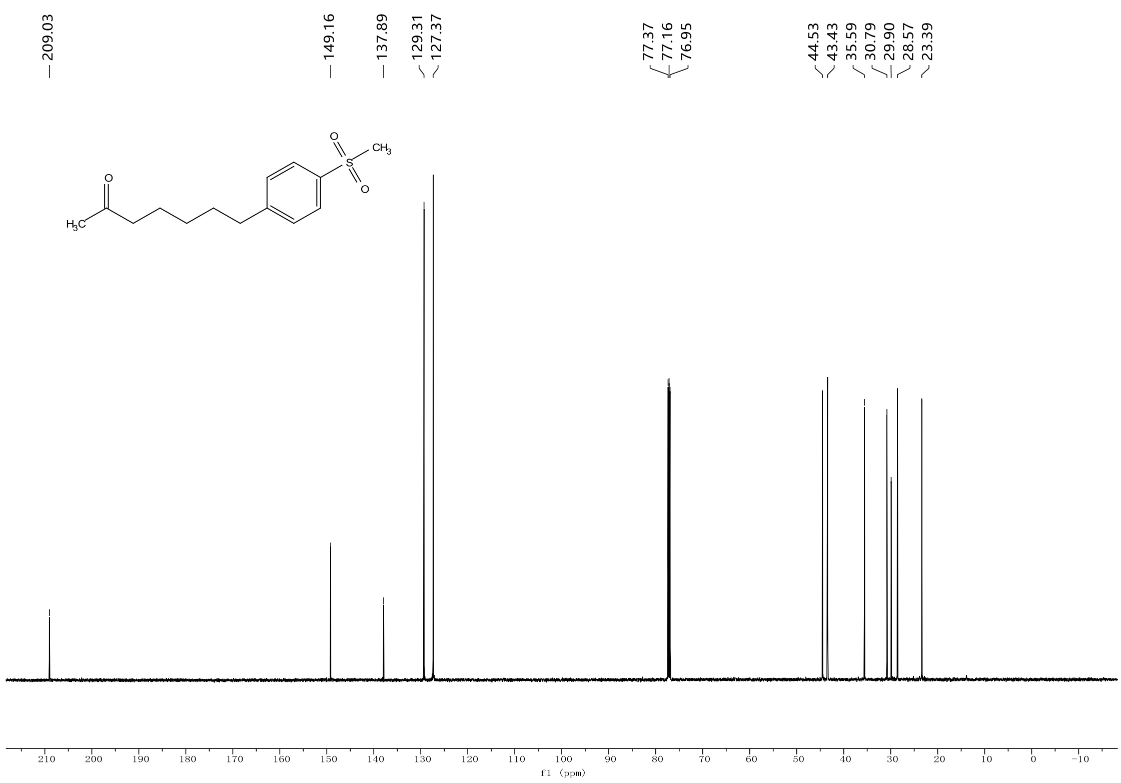

Supplementary Figure 97. ^{13}C NMR spectra of compound **32** (151 MHz, r.t., $CDCl_3$).

Compound 33

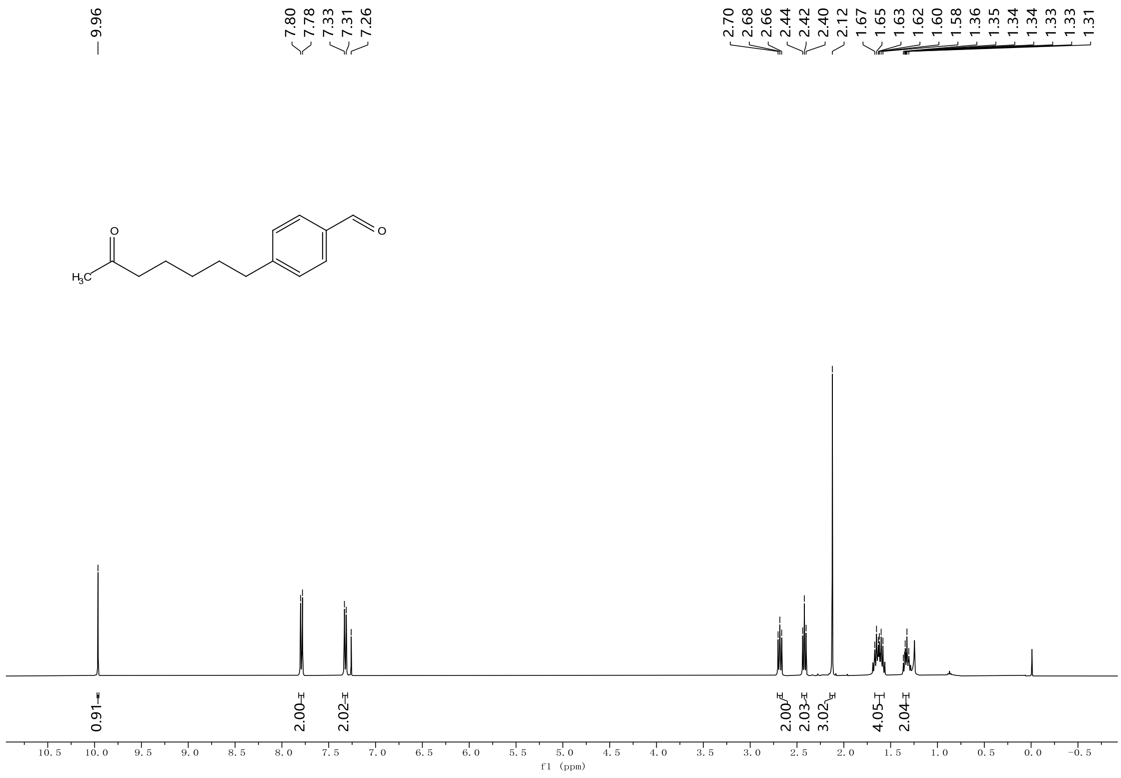

Supplementary Figure 98. ^{1}H NMR spectra of compound **33** (400 MHz, r.t., $CDCl_3$).

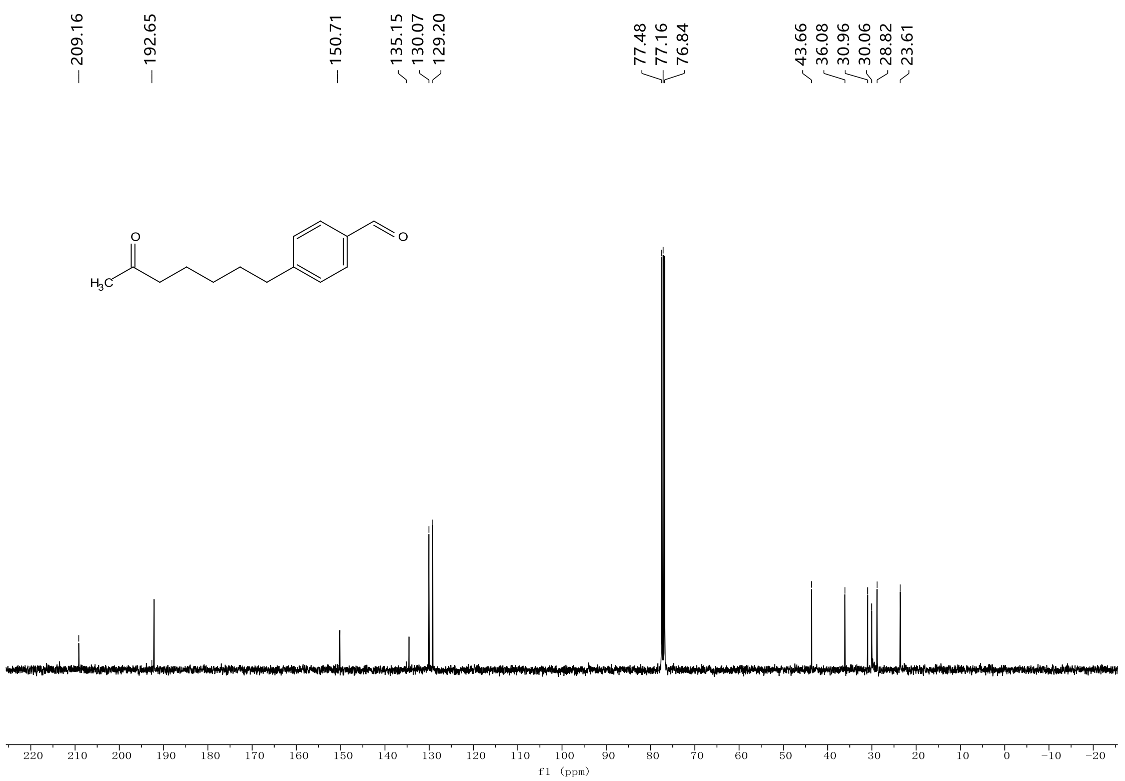

Supplementary Figure 99. ^{13}C NMR spectra of compound **33** (101 MHz, r.t., $CDCl_3$).

Compound 34

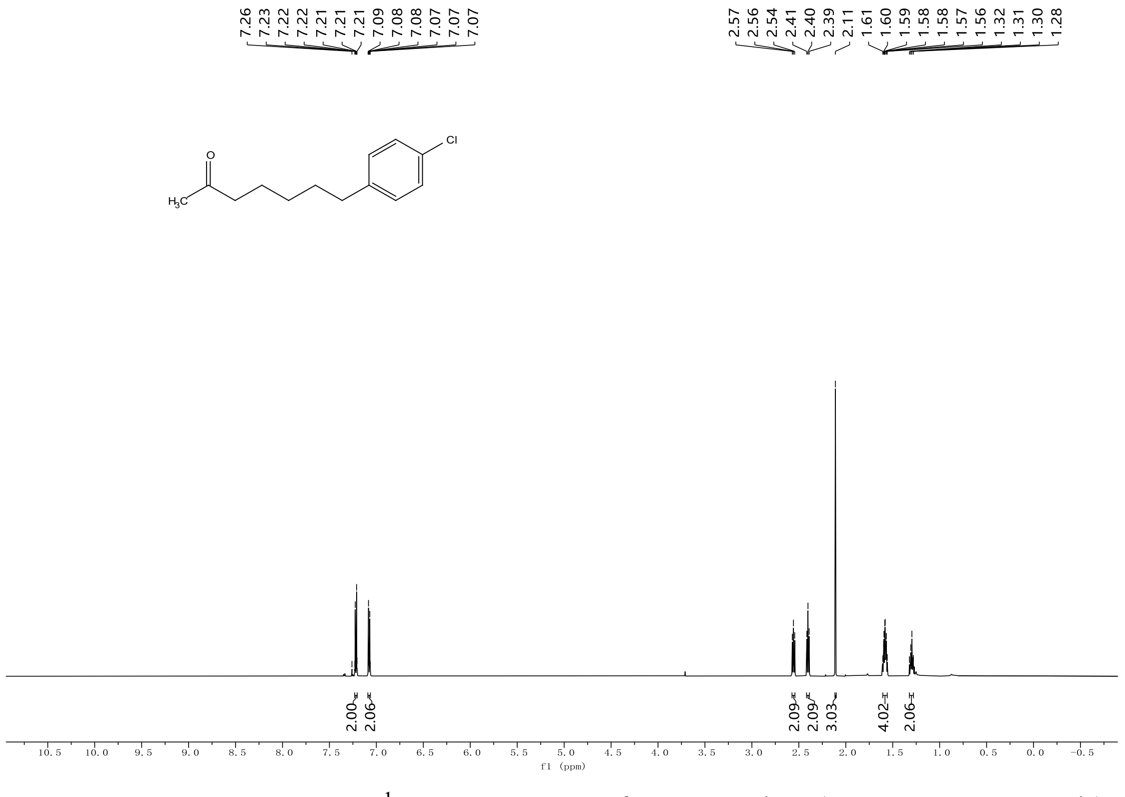

Supplementary Figure 100. ^{1}H NMR spectra of compound **34** (600 MHz, r.t., $CDCl_3$).

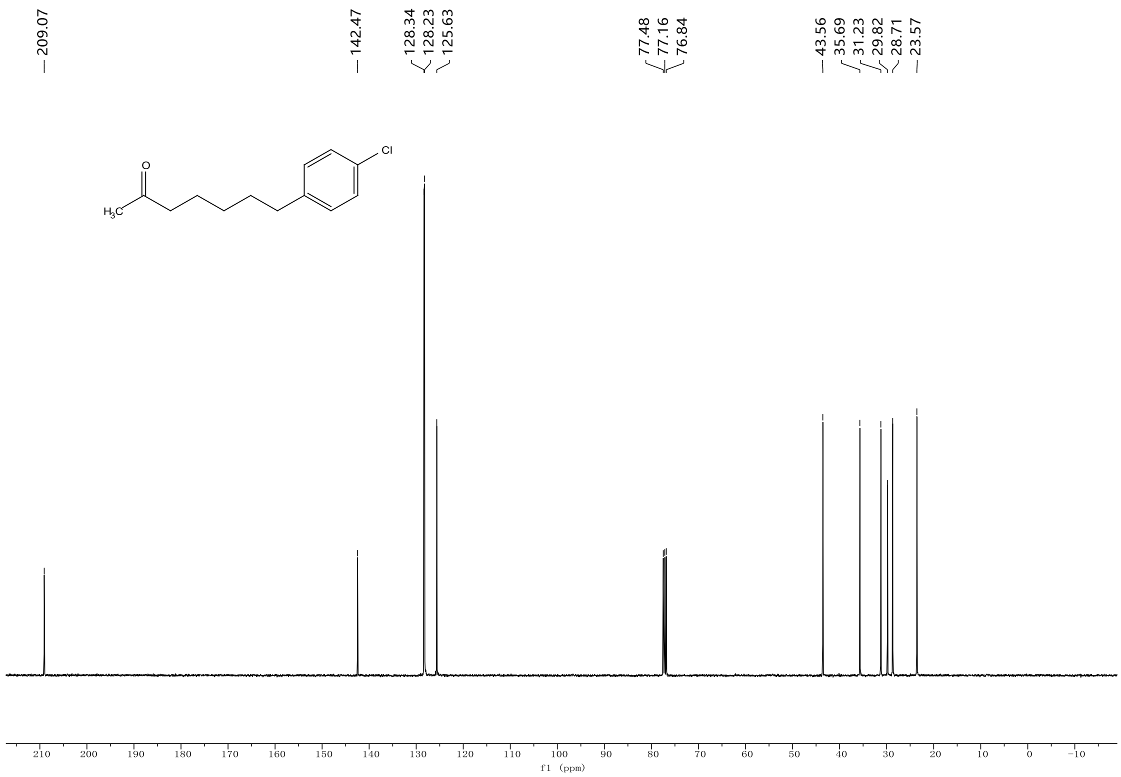

Supplementary Figure 101. ^{13}C NMR spectra of compound **34** (101 MHz, r.t., $CDCl_3$).

Compound 35

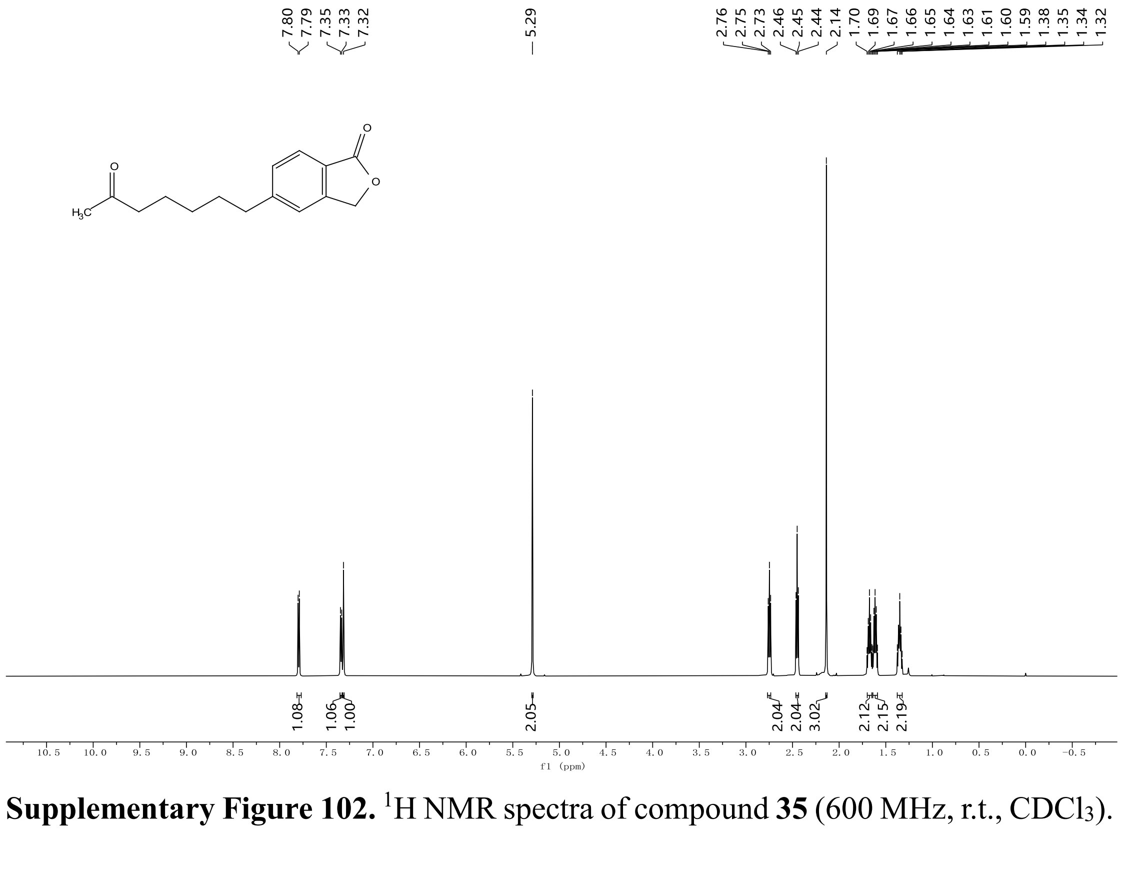

Supplementary Figure 102. ^{1}H NMR spectra of compound **35** (600 MHz, r.t., $CDCl_3$).

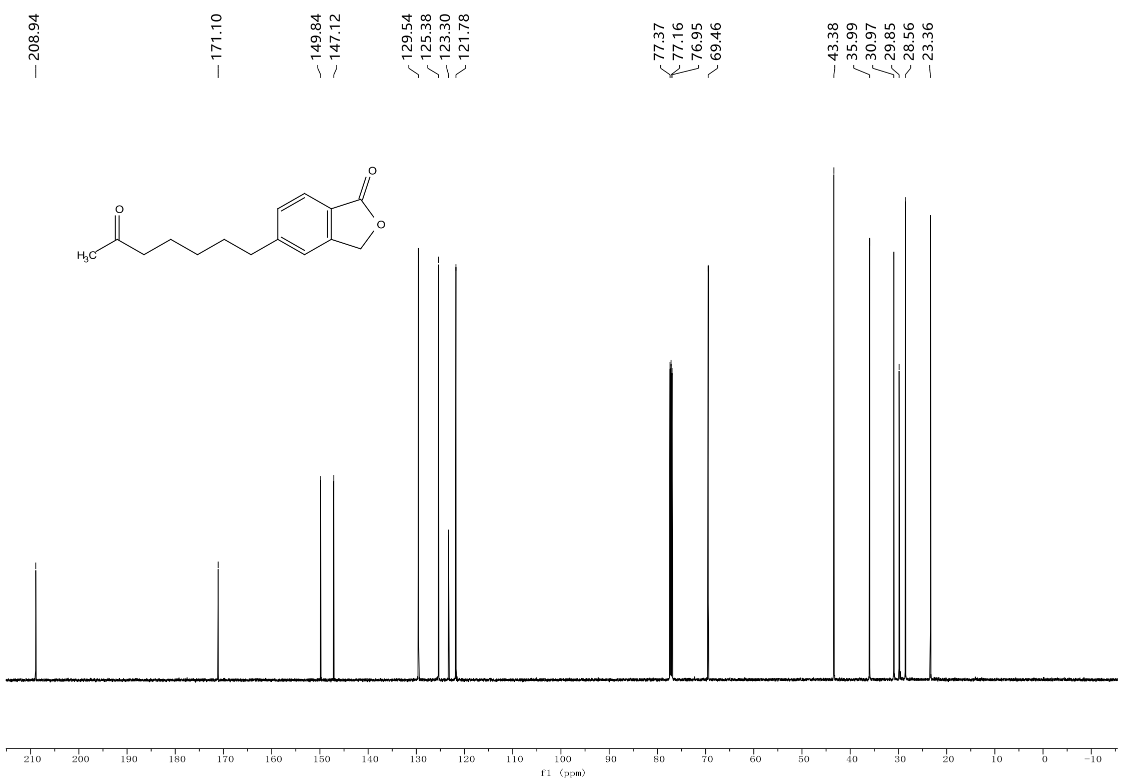

Supplementary Figure 103. ^{13}C NMR spectra of compound **35** (151 MHz, r.t., $CDCl_3$).

Compound 36

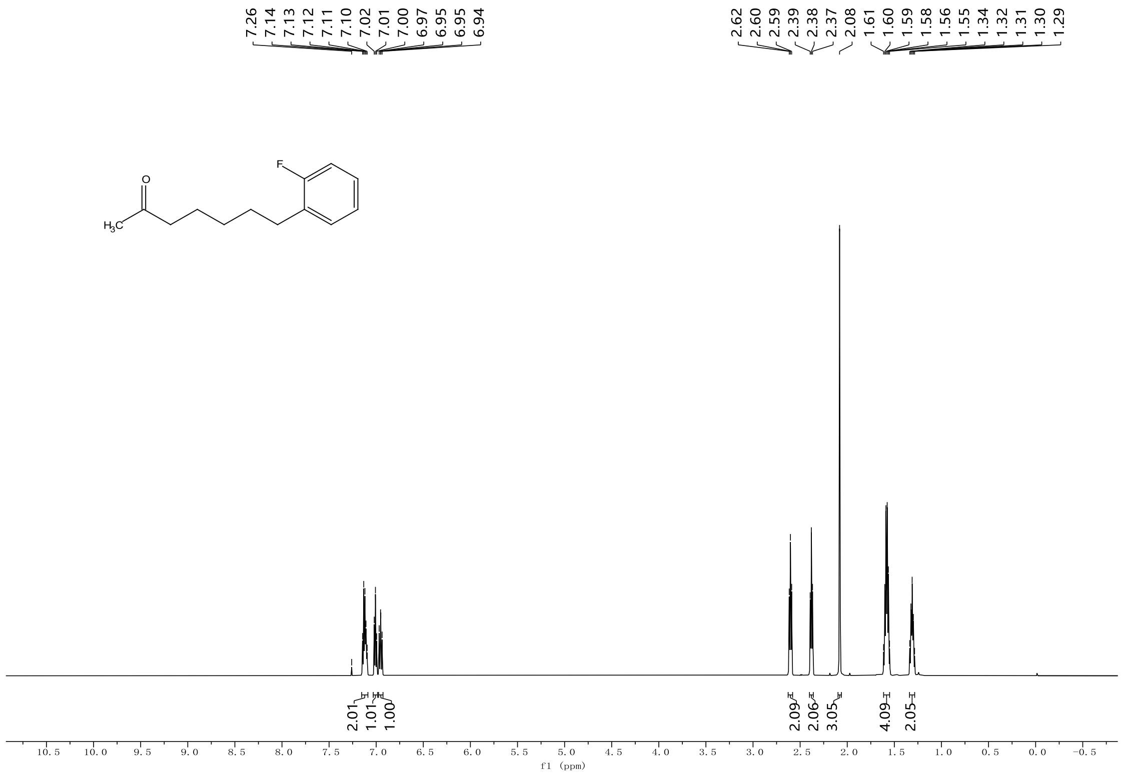

Supplementary Figure 104. ^{1}H NMR spectra of compound **36** (600 MHz, r.t., $CDCl_3$).

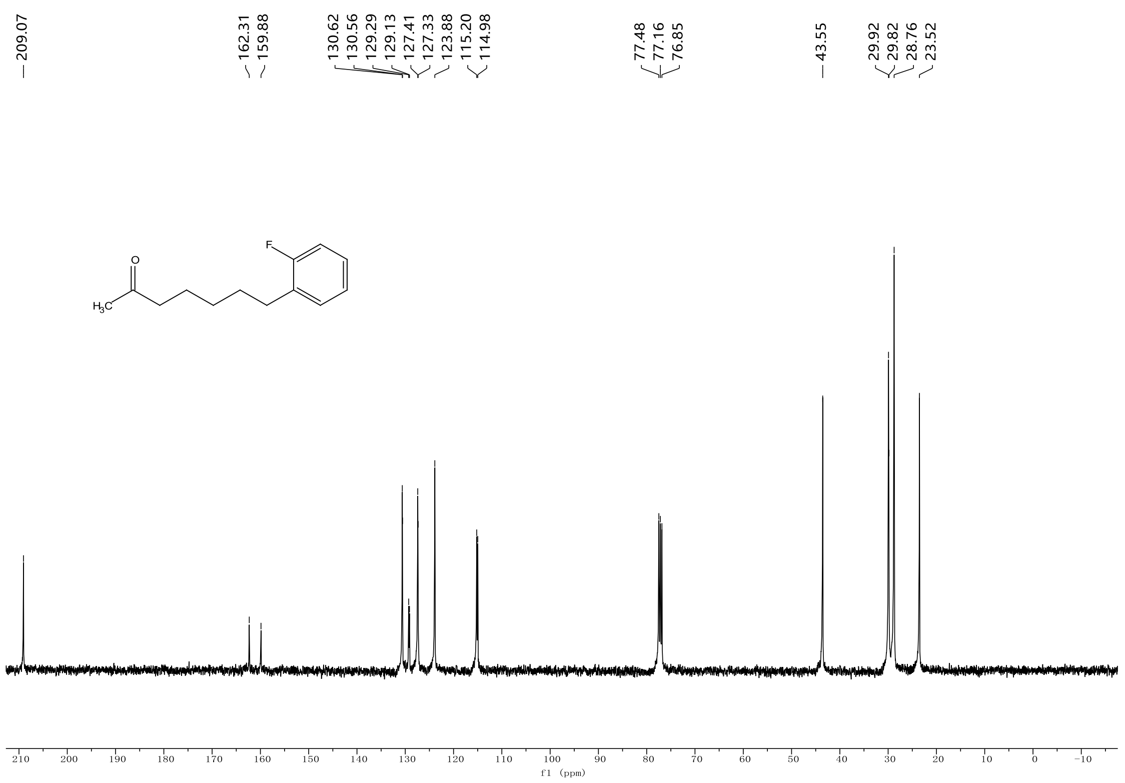

Supplementary Figure 105. ^{13}C NMR spectra of compound **36** (101 MHz, r.t., $CDCl_3$).

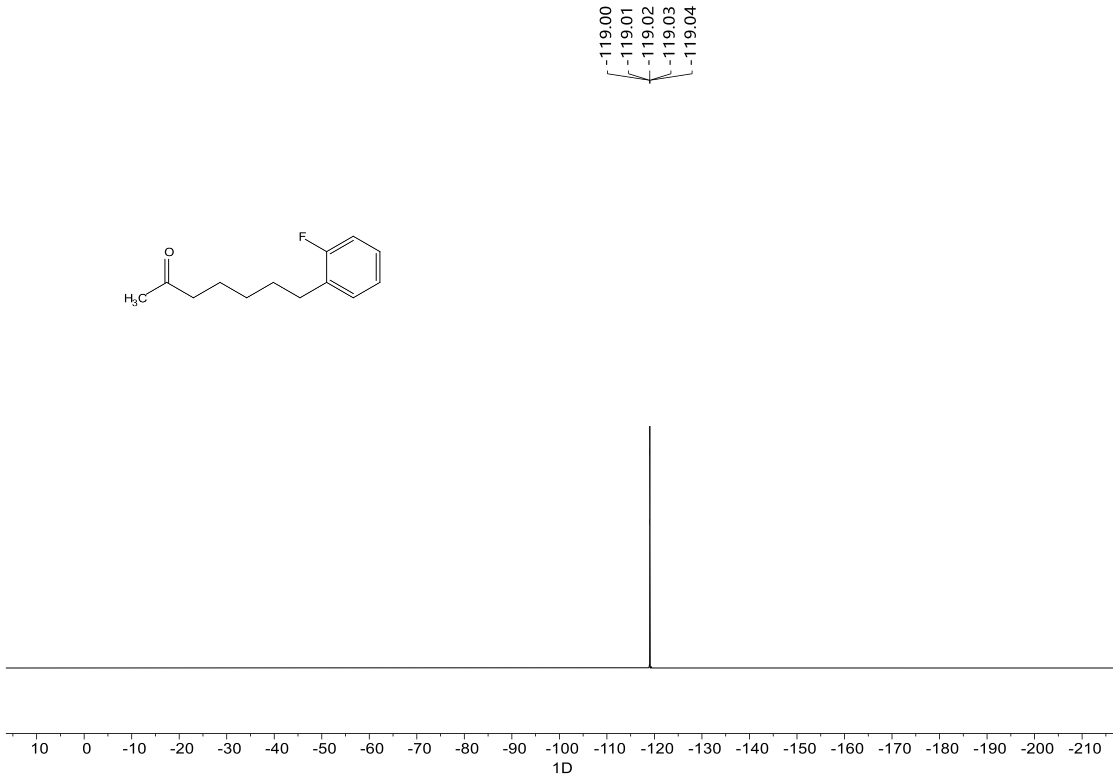

Supplementary Figure 106. ^{19}F NMR spectra of compound **36** (376 MHz, r.t., $CDCl_3$).

Compound 37

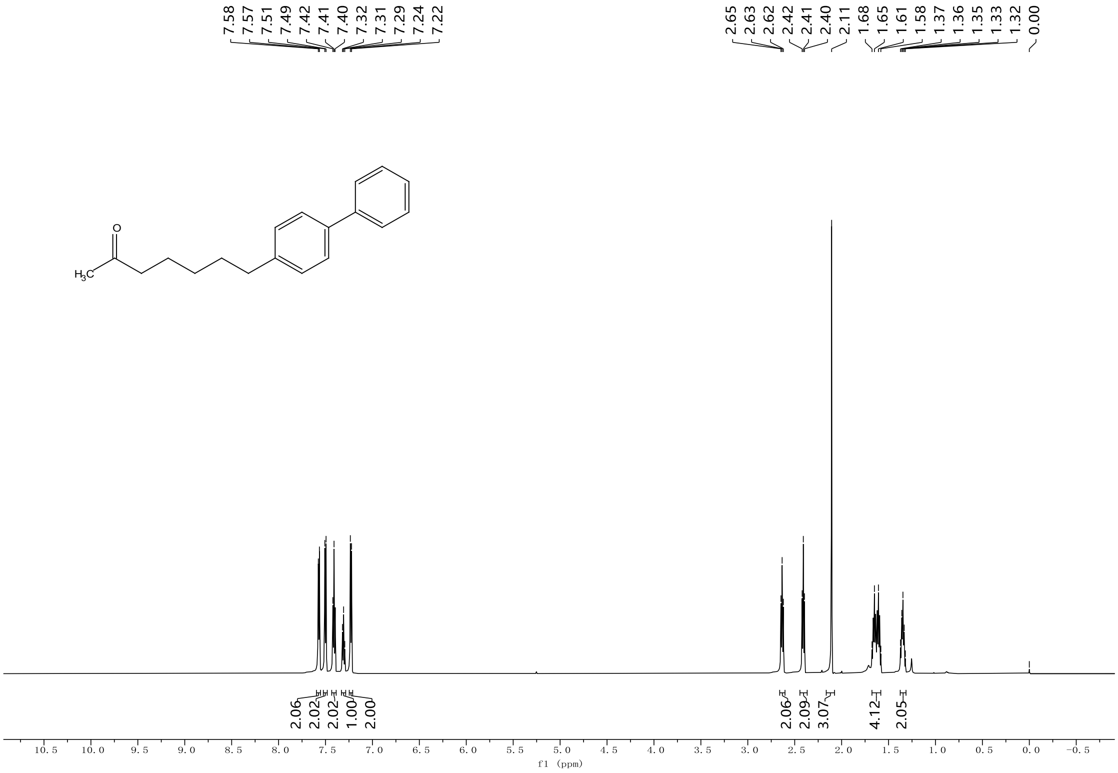

Supplementary Figure 107. ^{1}H NMR spectra of compound **37** (600 MHz, r.t., $CDCl_3$).

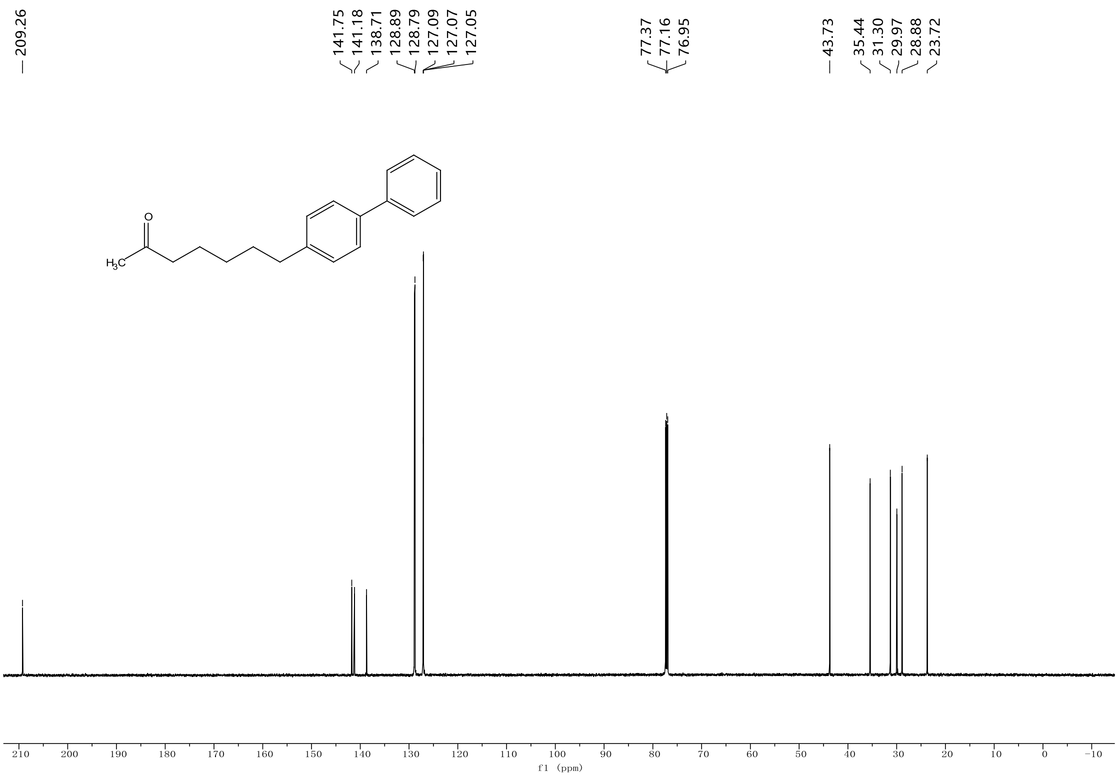

Supplementary Figure 108. ^{13}C NMR spectra of compound **37** (151 MHz, r.t., $CDCl_3$).

Compound 38

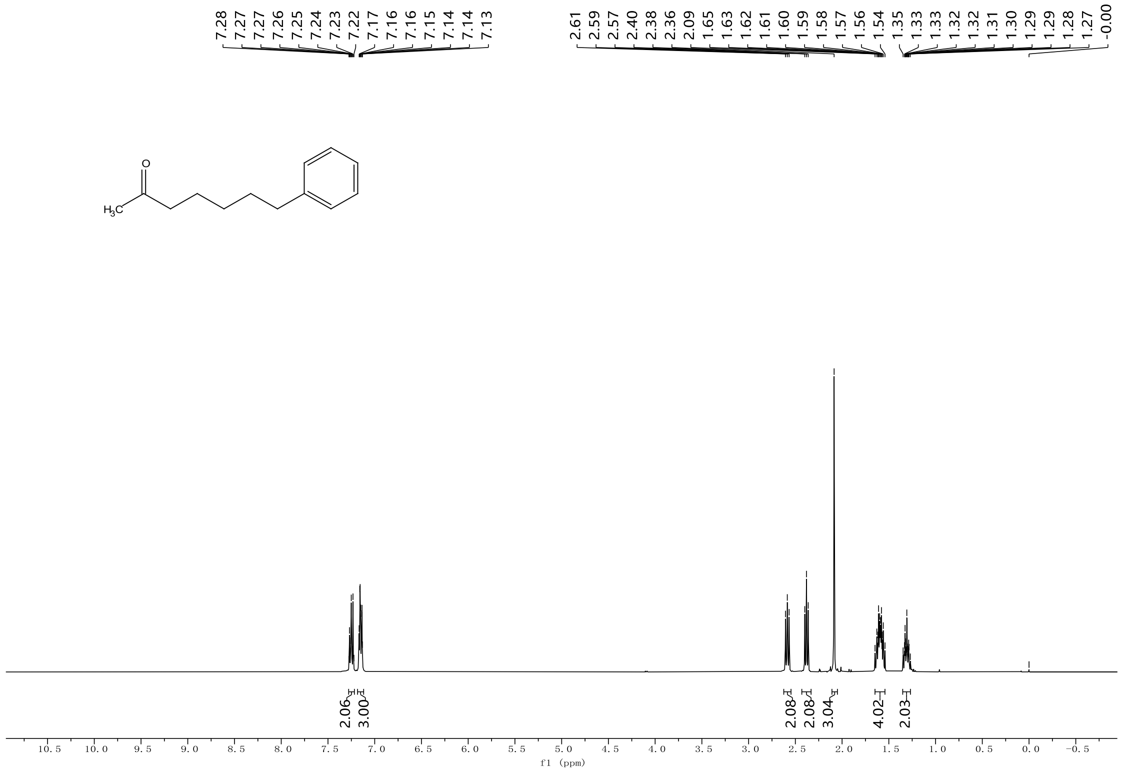

Supplementary Figure 109. ^{1}H NMR spectra of compound **38** (400 MHz, r.t., $CDCl_3$).

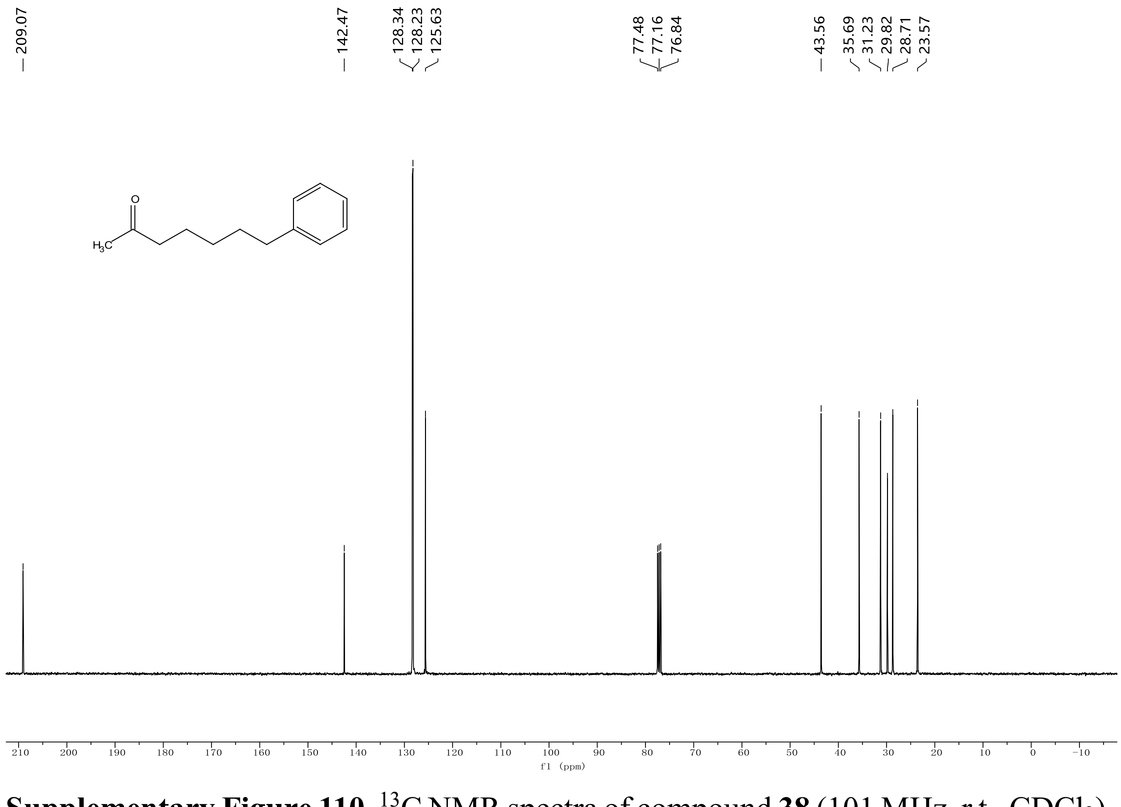

Supplementary Figure 110. ^{13}C NMR spectra of compound **38** (101 MHz, r.t., $CDCl_3$).

Compound 39

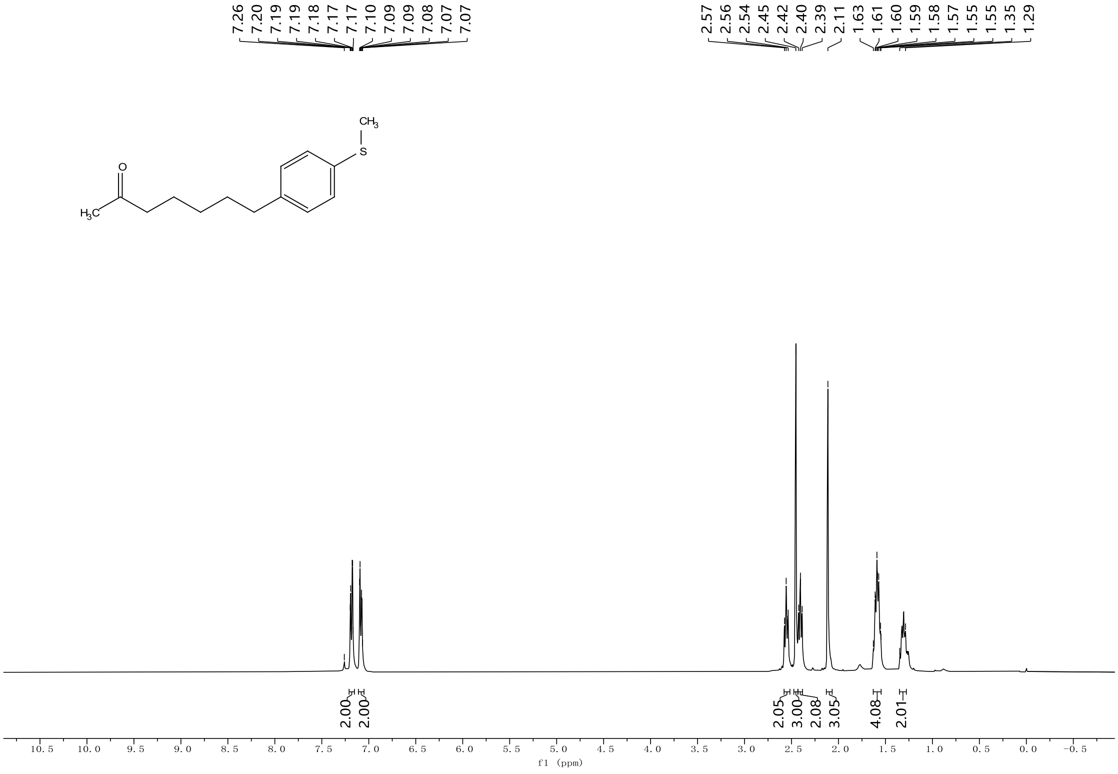

Supplementary Figure 111. ^{1}H NMR spectra of compound **39** (400 MHz, r.t., $CDCl_3$).

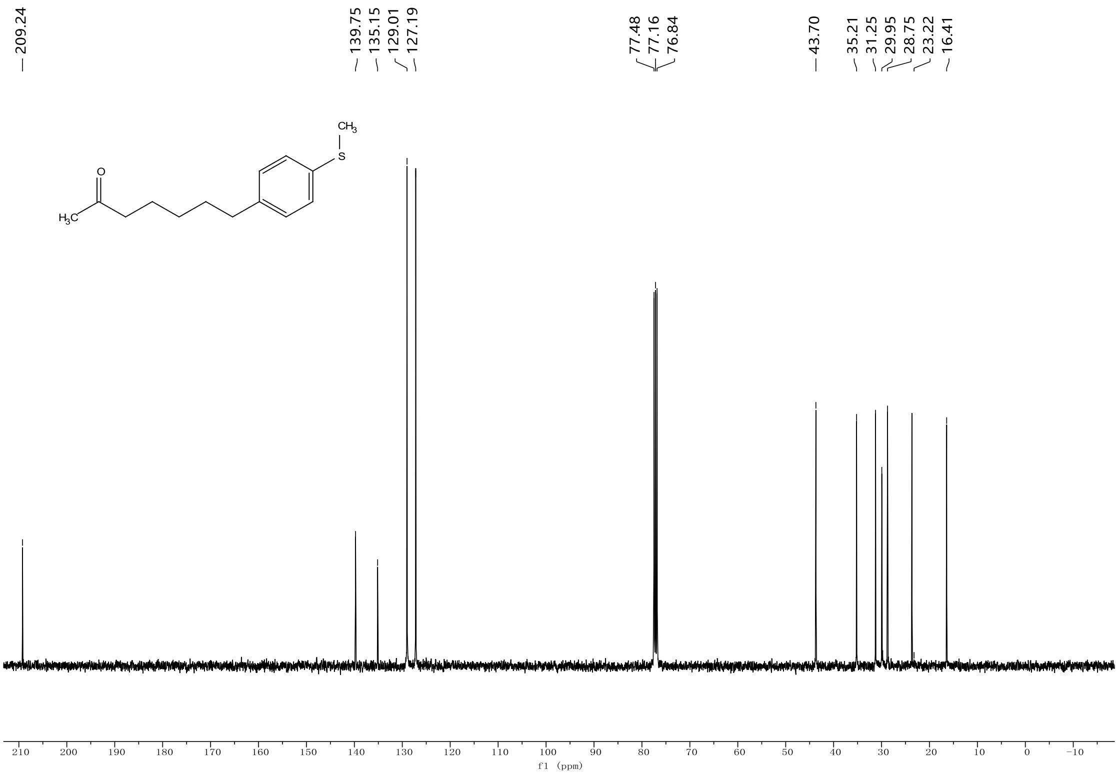

Supplementary Figure 112. ^{13}C NMR spectra of compound **39** (101 MHz, r.t., $CDCl_3$).

Compound 40

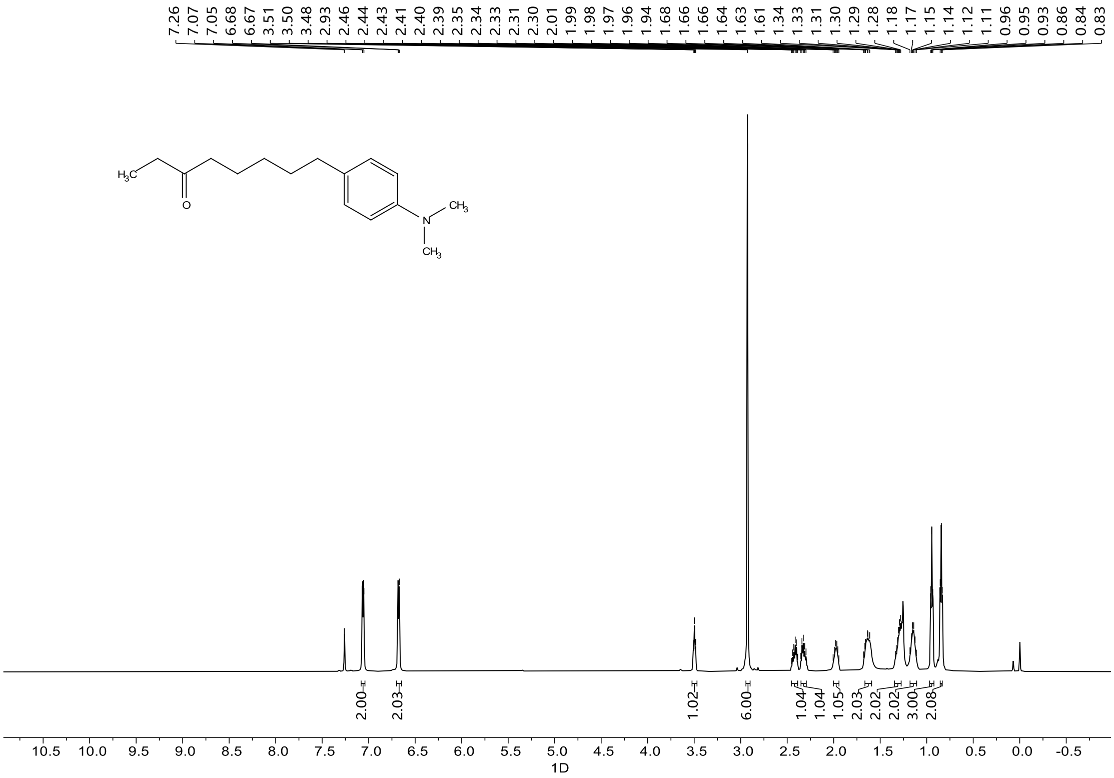

Supplementary Figure 113. ^{1}H NMR spectra of compound **40** (600 MHz, r.t., $CDCl_3$)

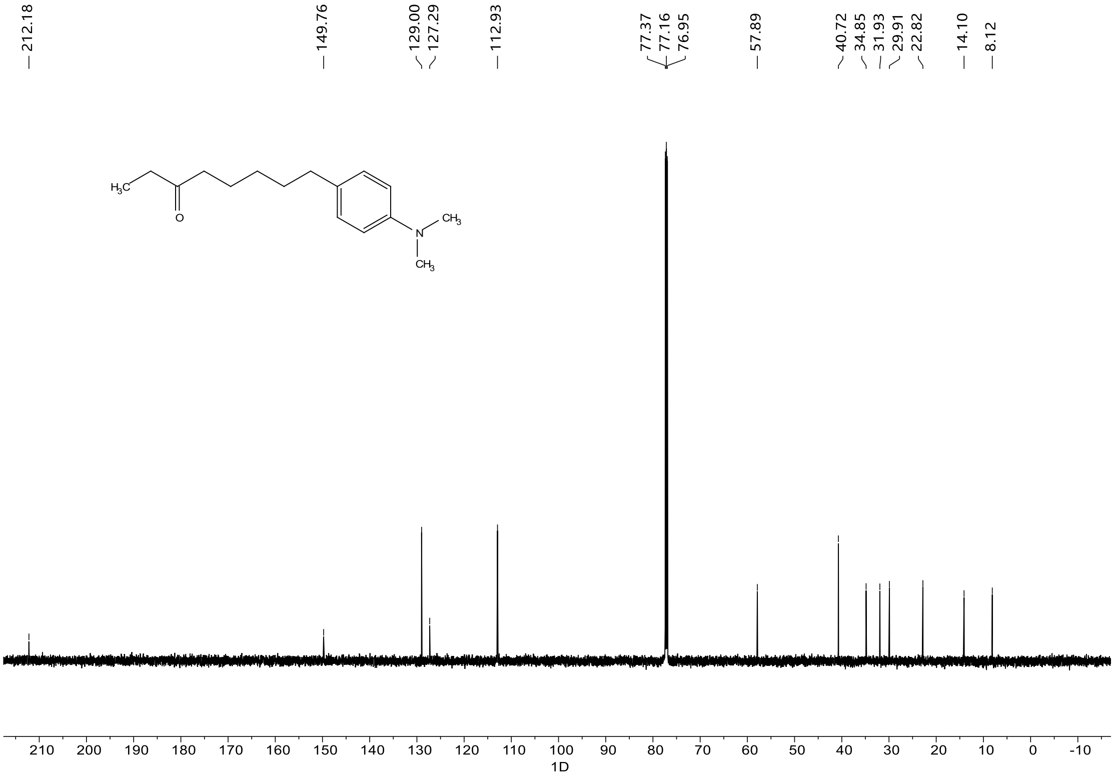

Supplementary Figure 114. ^{13}C NMR spectra of compound **40** (151 MHz, r.t., $CDCl_3$).

Compound 41

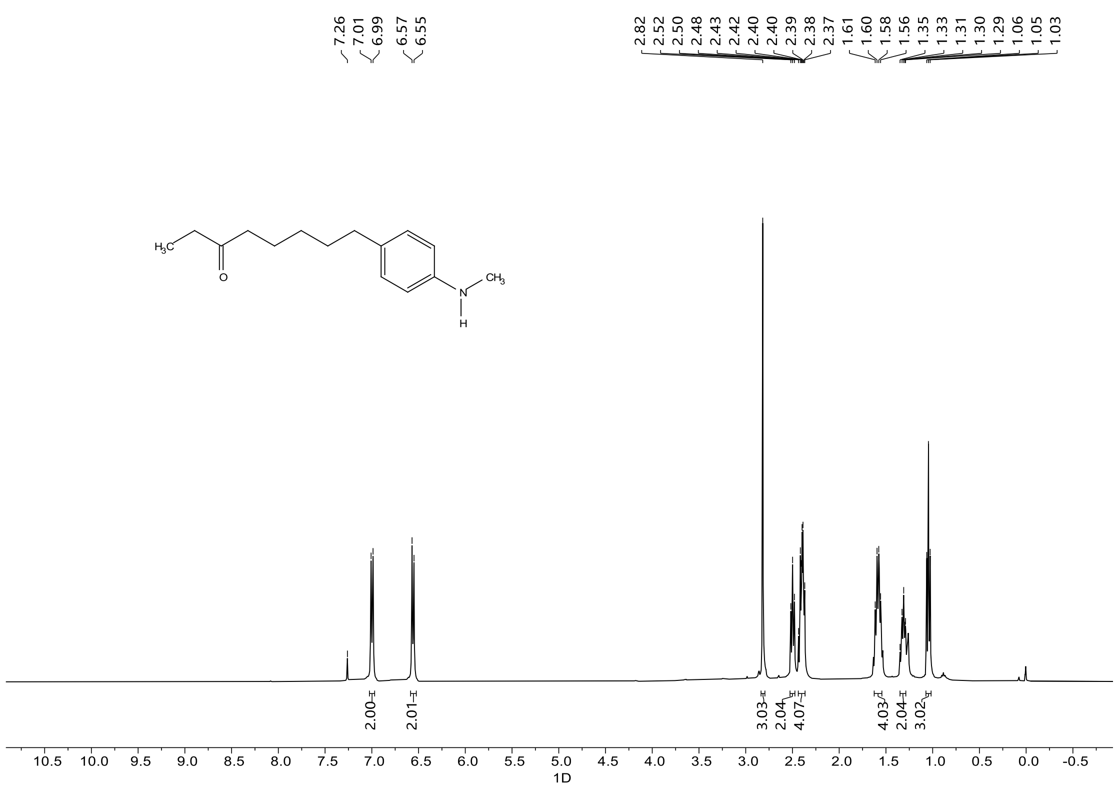

Supplementary Figure 115. ^{1}H NMR spectra of compound **41** (400 MHz, r.t., $CDCl_3$).

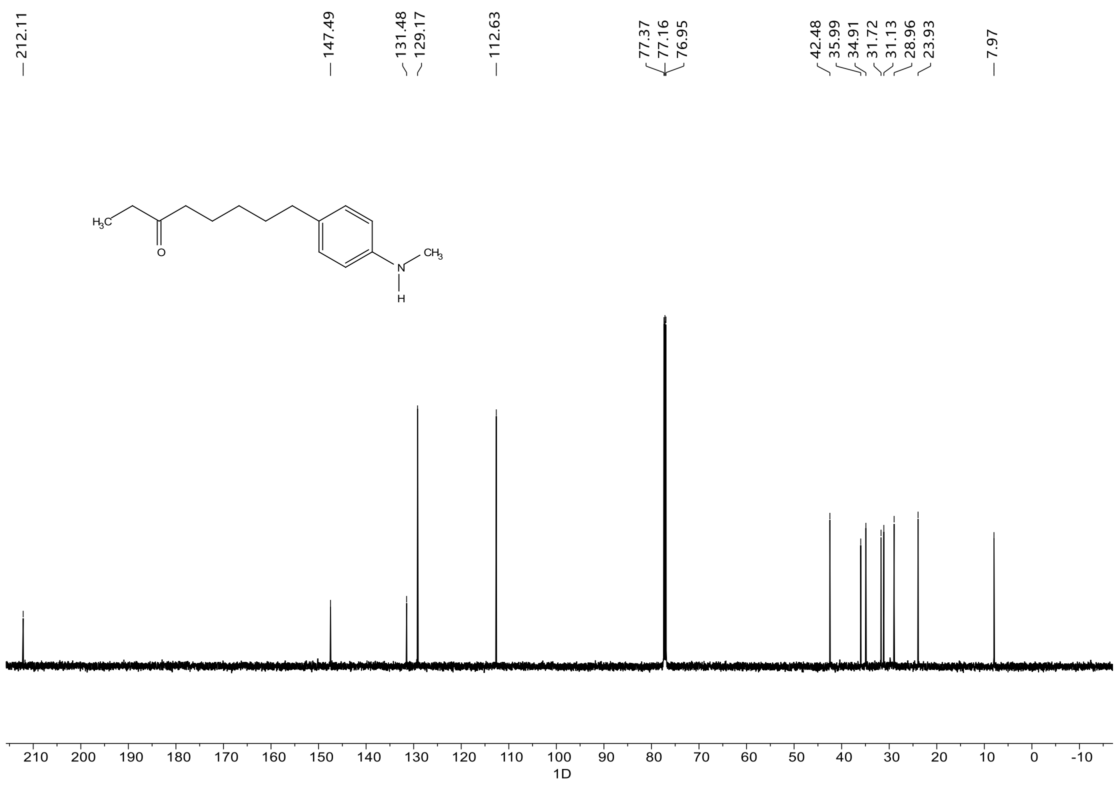

Supplementary Figure 116. ^{13}C NMR spectra of compound **41** (101 MHz, r.t., $CDCl_3$).

Compound 42

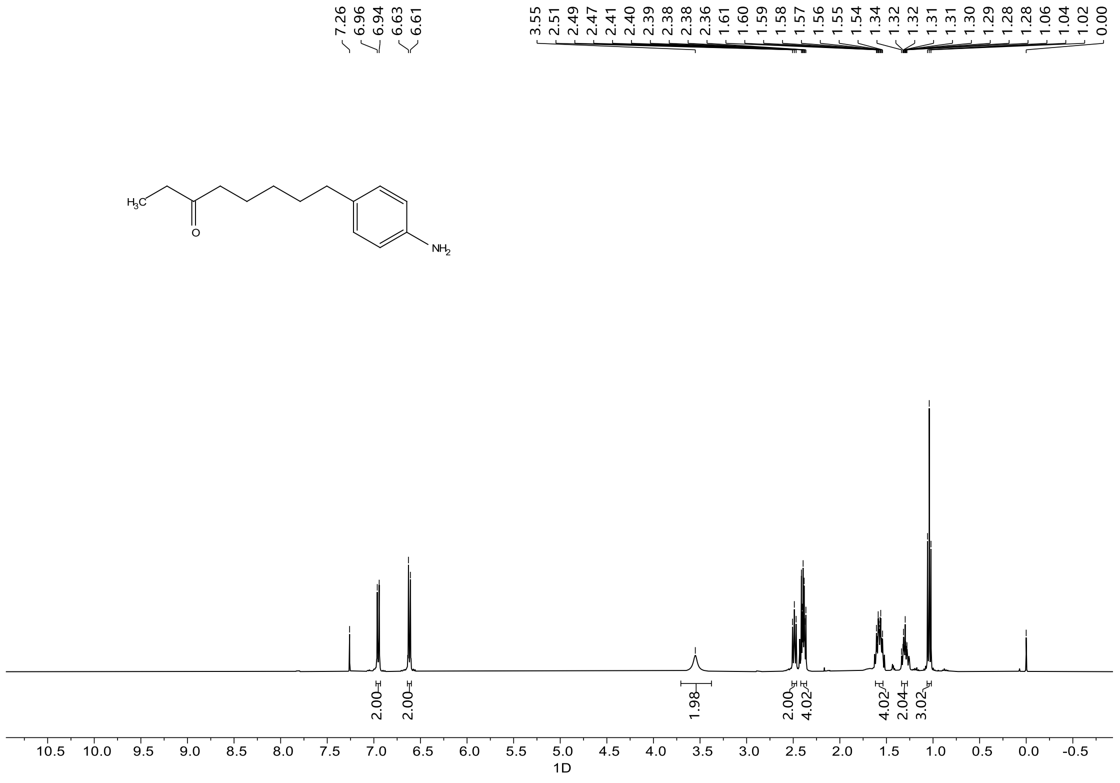

Supplementary Figure 117. ^{1}H NMR spectra of compound **42** (400 MHz, r.t., $CDCl_3$).

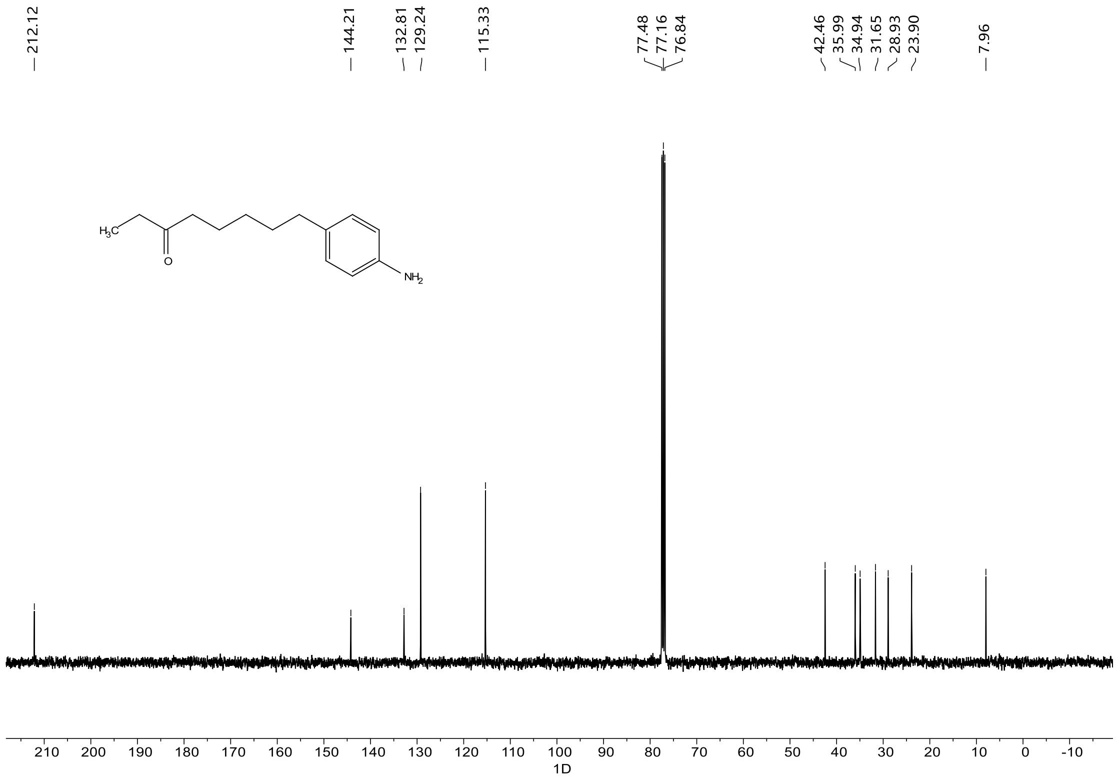

Supplementary Figure 118. ^{13}C NMR spectra of compound **42** (151 MHz, r.t., $CDCl_3$).

Compound 43

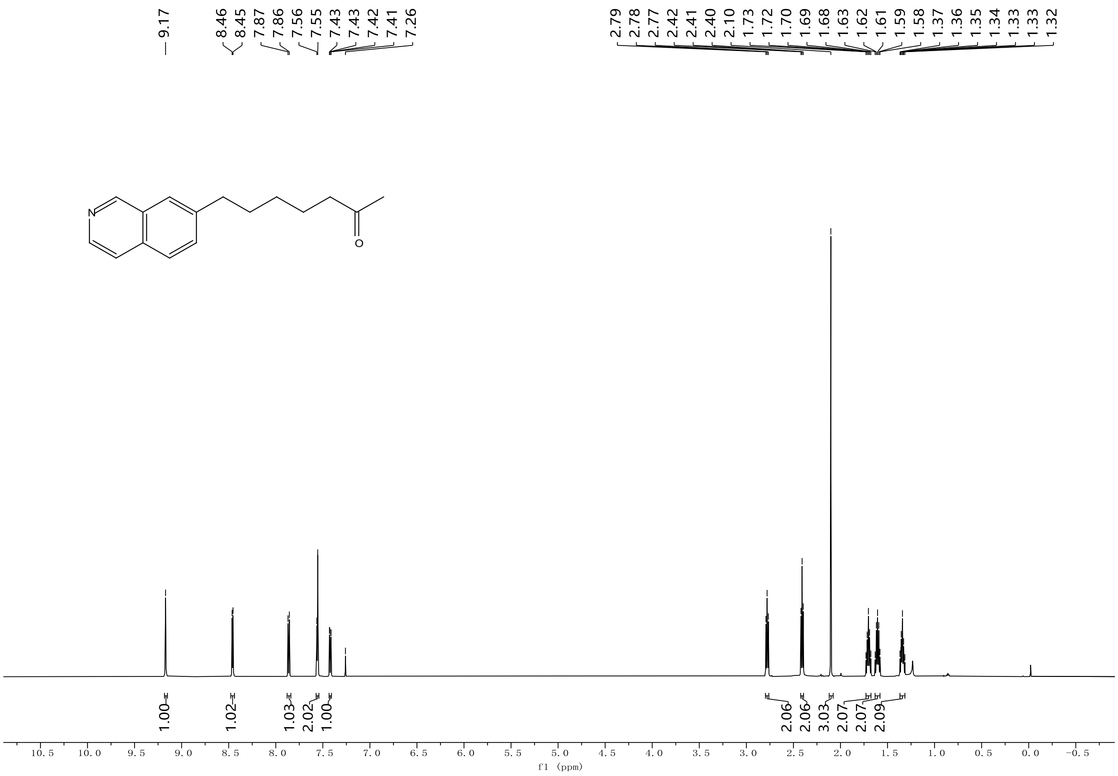

Supplementary Figure 119. ^{1}H NMR spectra of compound **43** (600 MHz, r.t., $CDCl_3$).

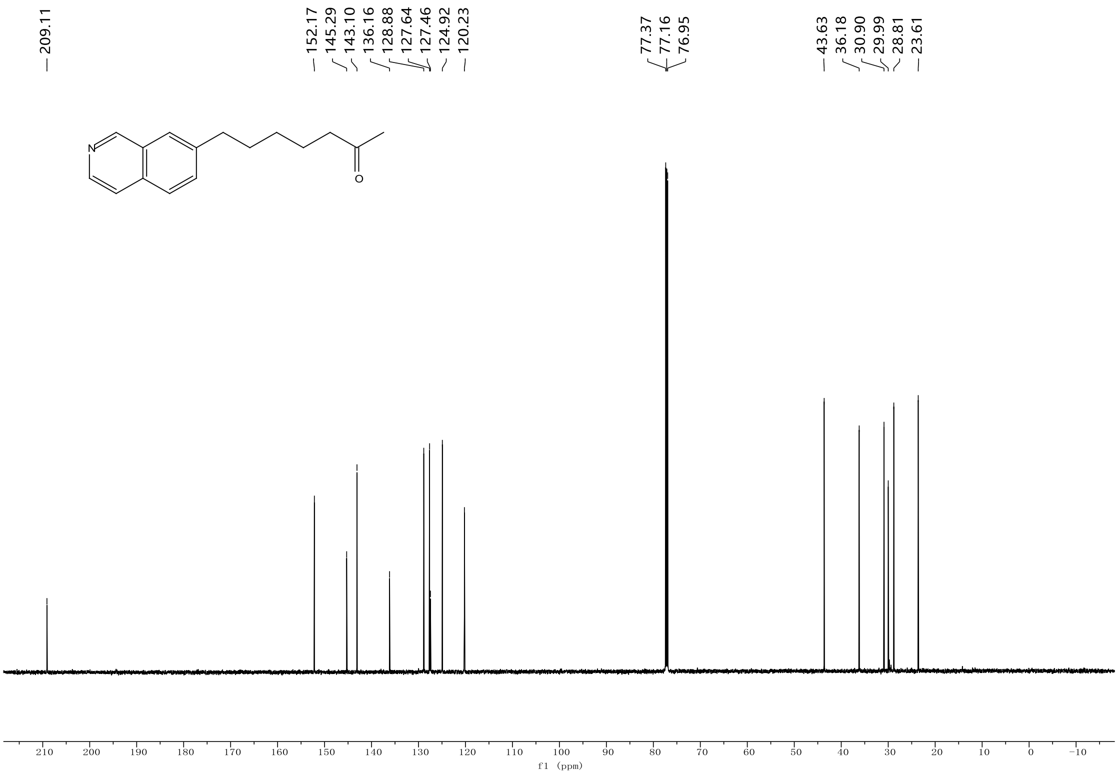

Supplementary Figure 120. ^{13}C NMR spectra of compound **43** (151 MHz, r.t., $CDCl_3$).

Compound 44

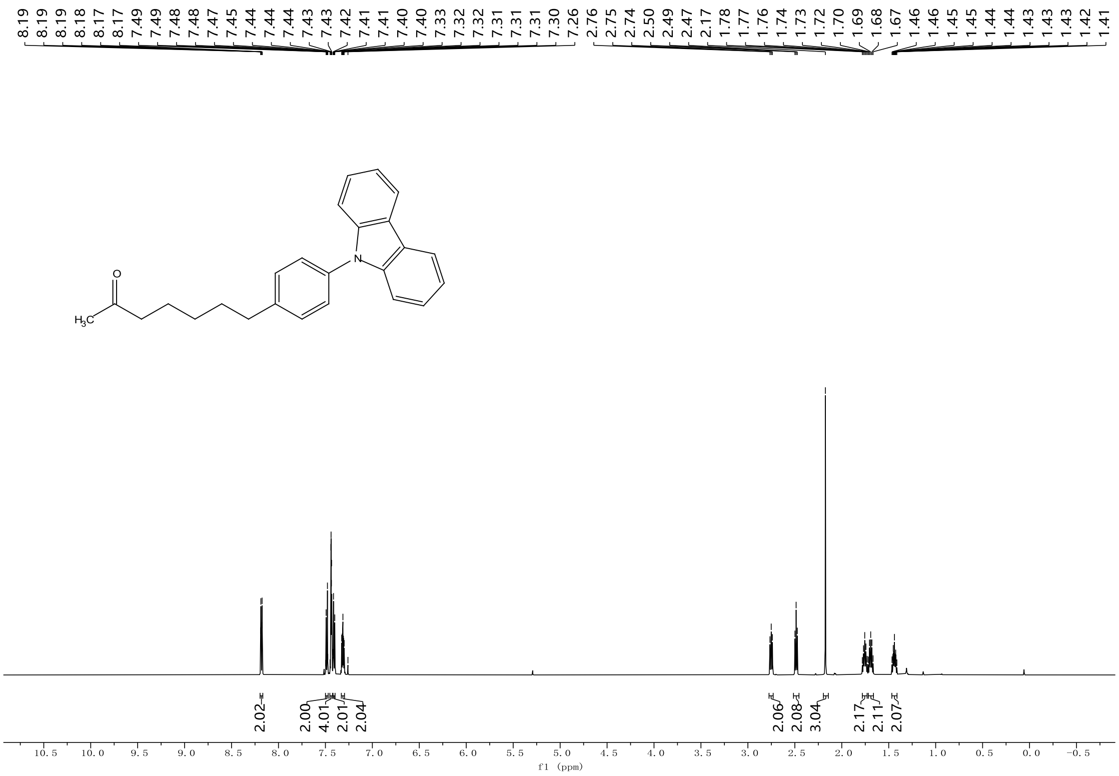

Supplementary Figure 121. ^{1}H NMR spectra of compound **44** (600 MHz, r.t., $CDCl_3$).

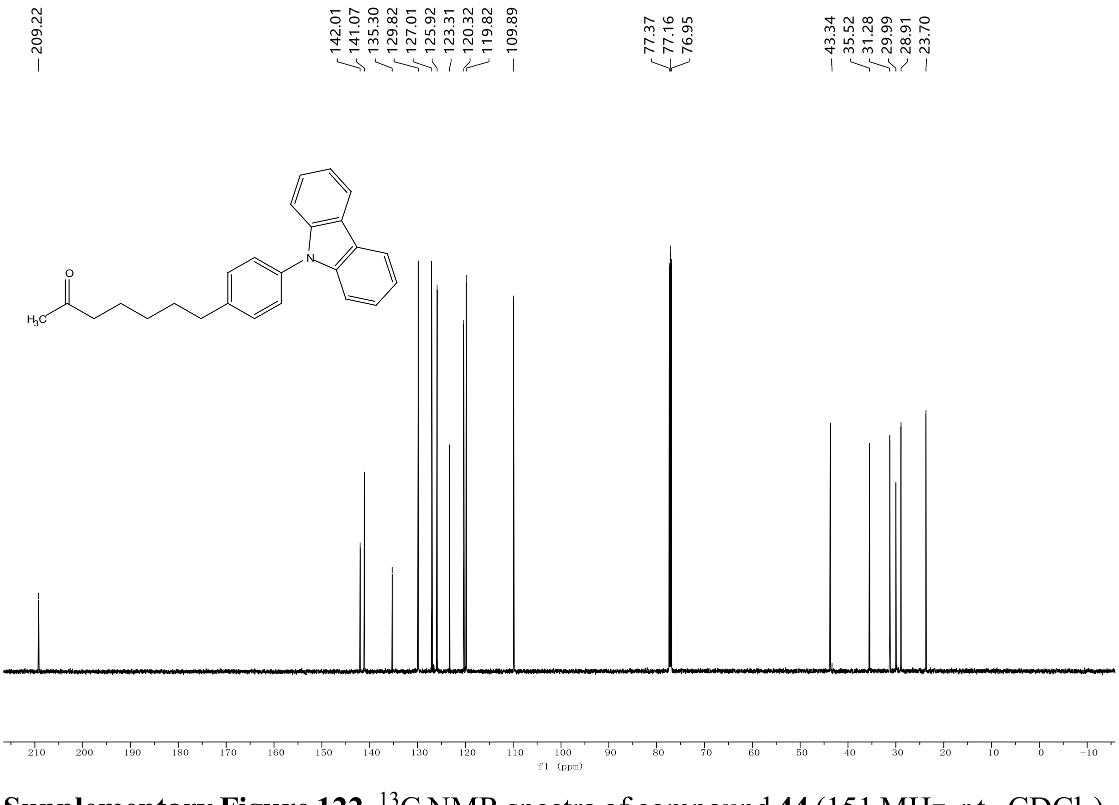

Supplementary Figure 122. ^{13}C NMR spectra of compound **44** (151 MHz, r.t., $CDCl_3$).

Compound 45

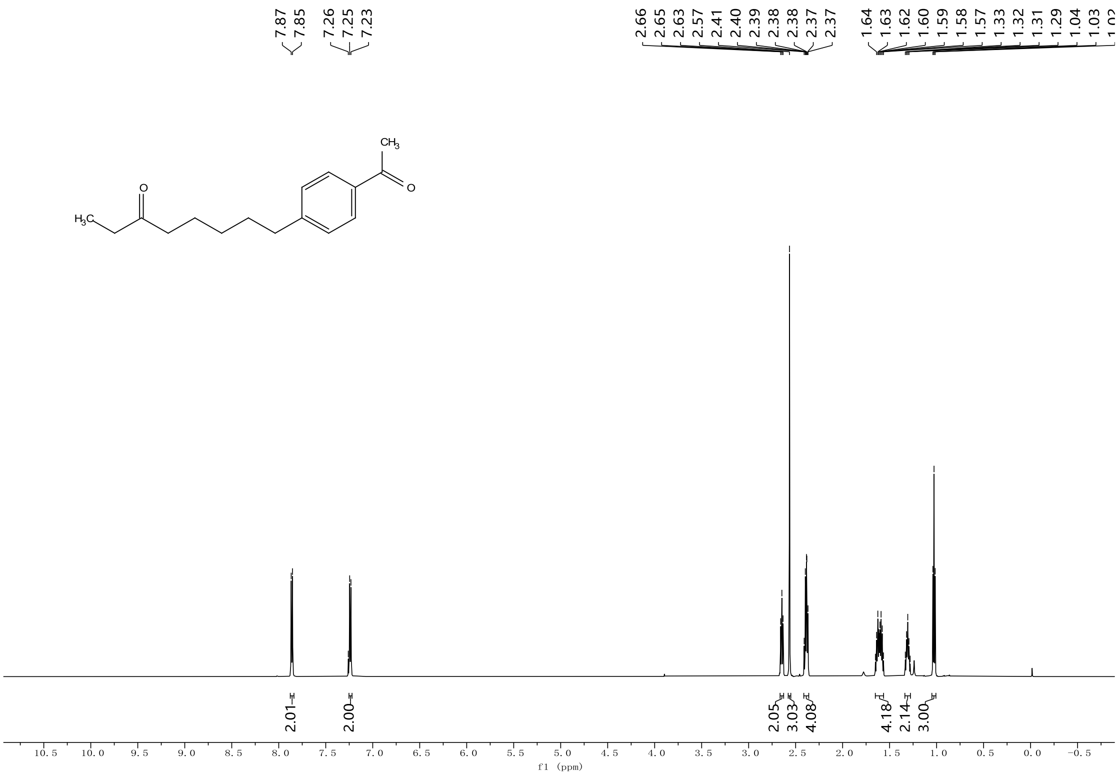

Supplementary Figure 123. ^{1}H NMR spectra of compound **45** (600 MHz, r.t., $CDCl_3$).

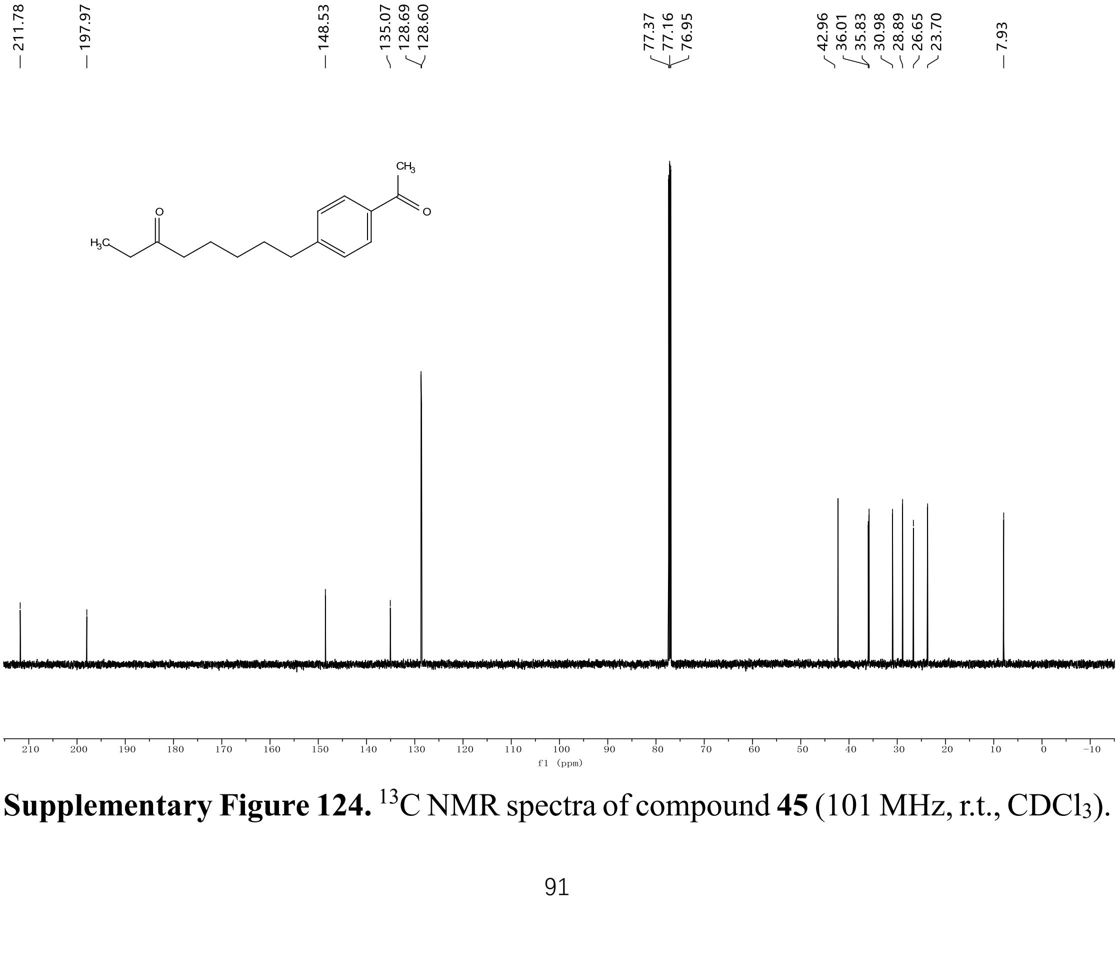

Supplementary Figure 124. ^{13}C NMR spectra of compound **45** (101 MHz, r.t., $CDCl_3$).

Compound 46

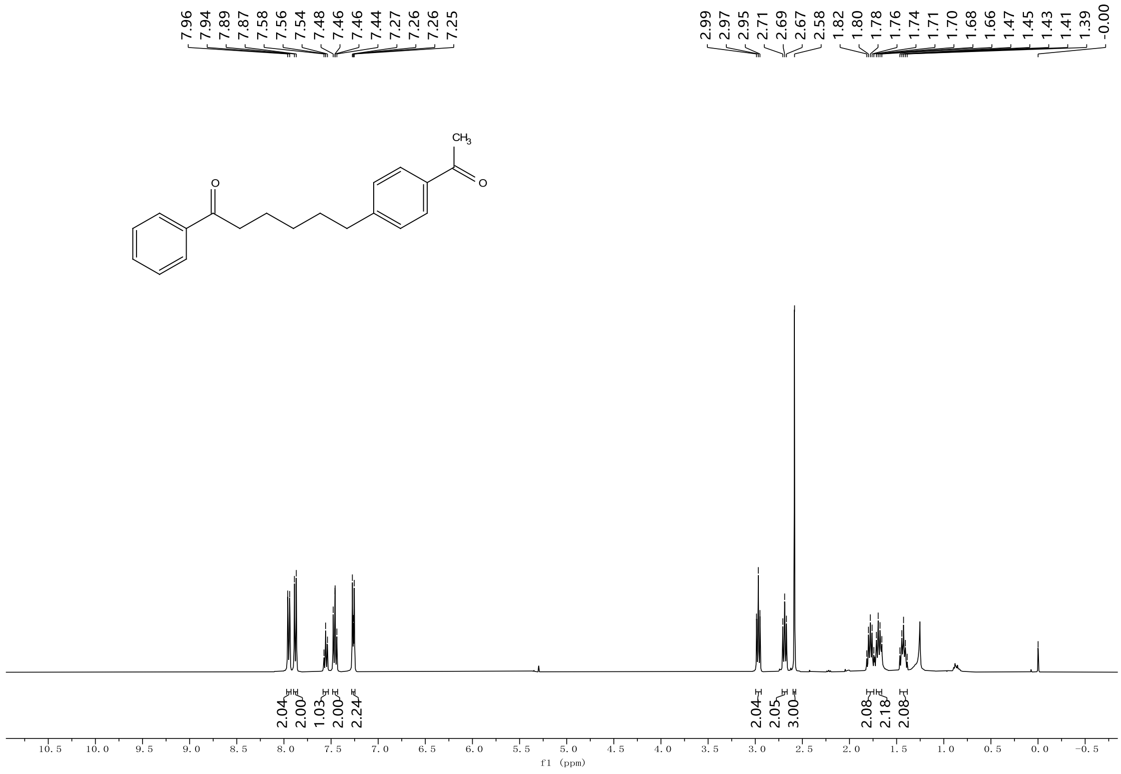

Supplementary Figure 125. ^{1}H NMR spectra of compound **46** (400 MHz, r.t., $CDCl_3$).

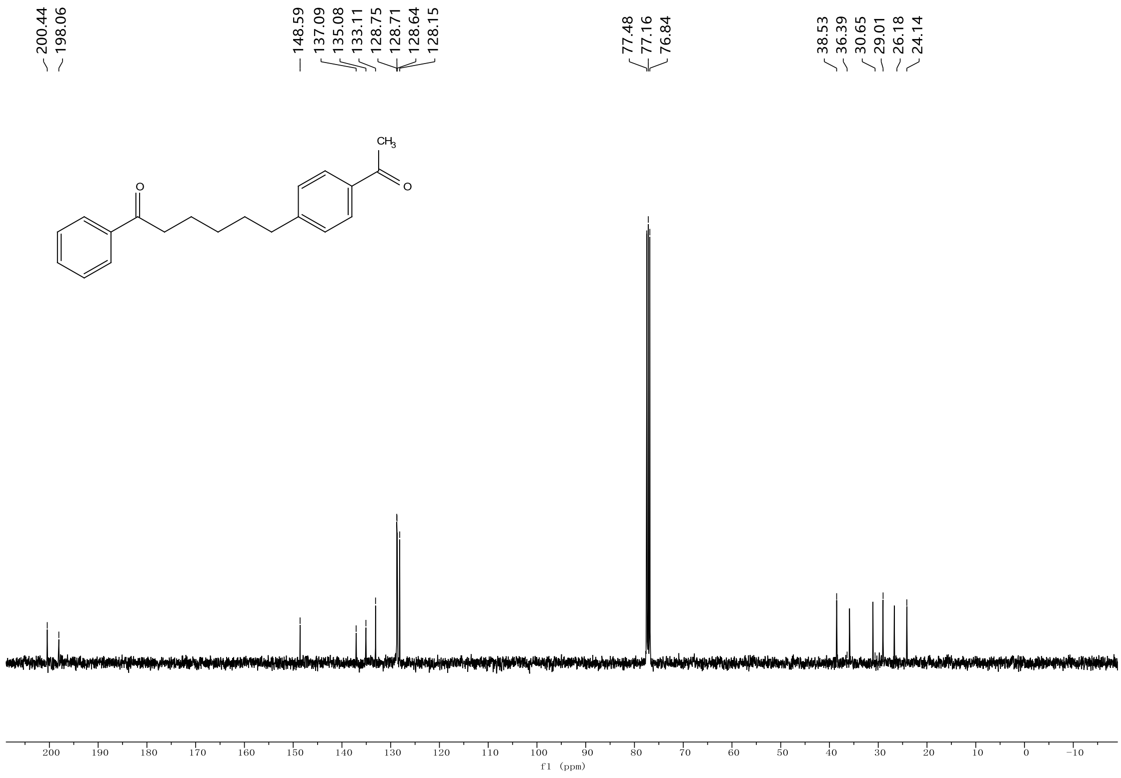

Supplementary Figure 126. ^{13}C NMR spectra of compound **46** (101 MHz, r.t., $CDCl_3$).

Compound 47

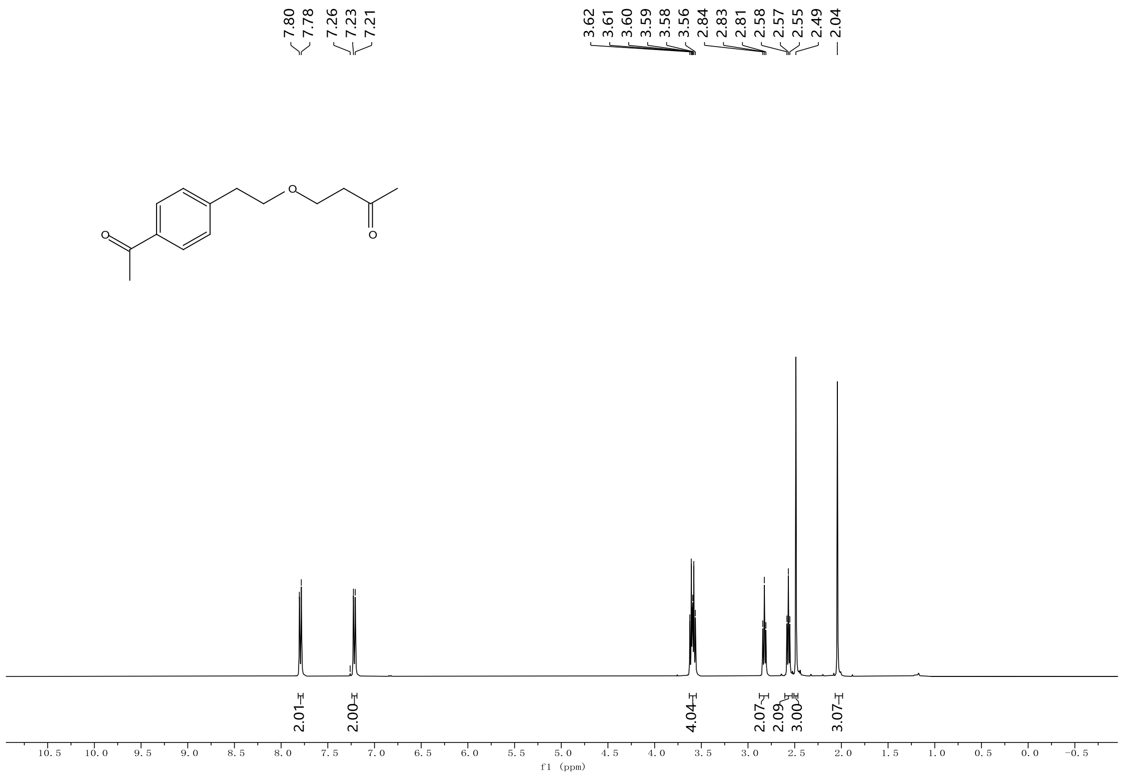

Supplementary Figure 127. ^{1}H NMR spectra of compound **47** (400 MHz, r.t., $CDCl_3$).

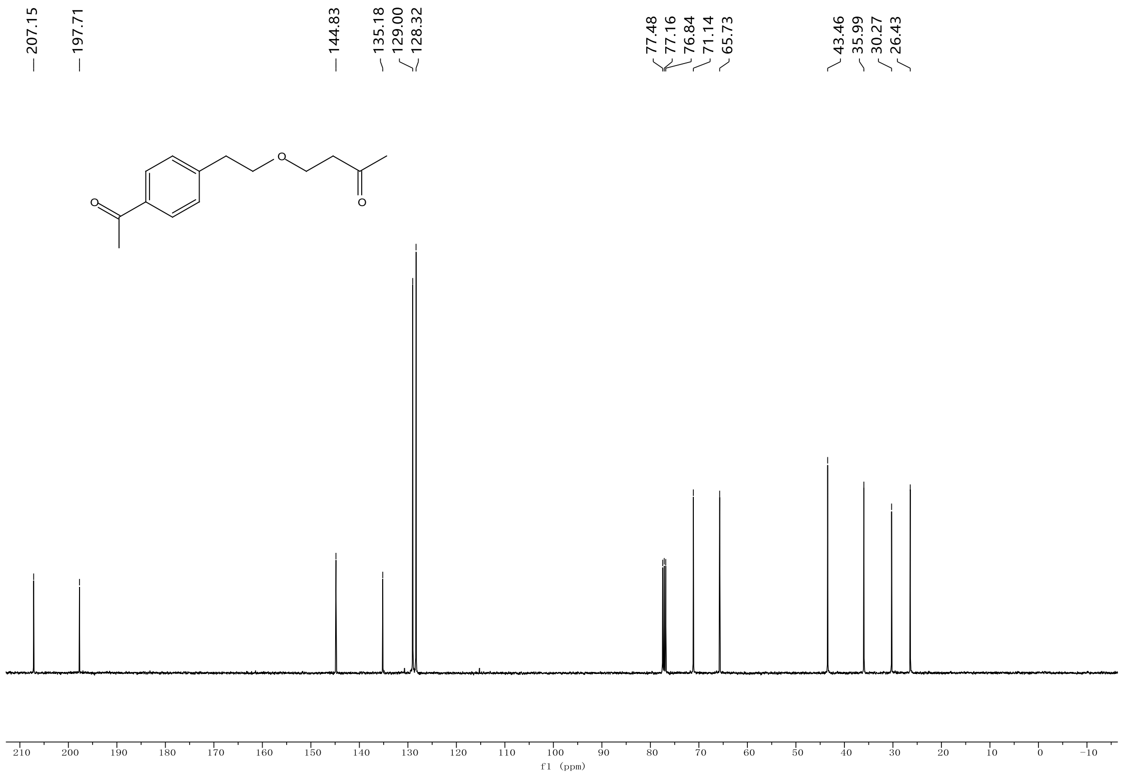

Supplementary Figure 128. ^{13}C NMR spectra of compound **47** (101 MHz, r.t., $CDCl_3$).

Compound 48

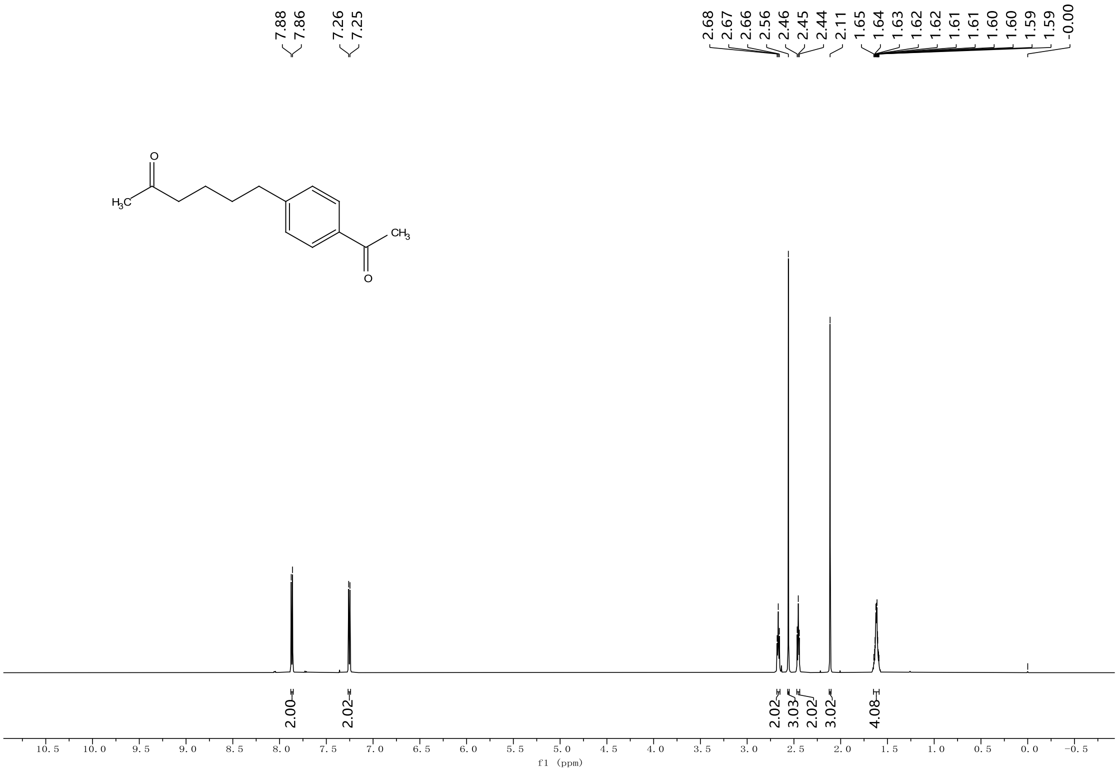

Supplementary Figure 129. ^{1}H NMR spectra of compound **48** (600 MHz, r.t., $CDCl_3$).

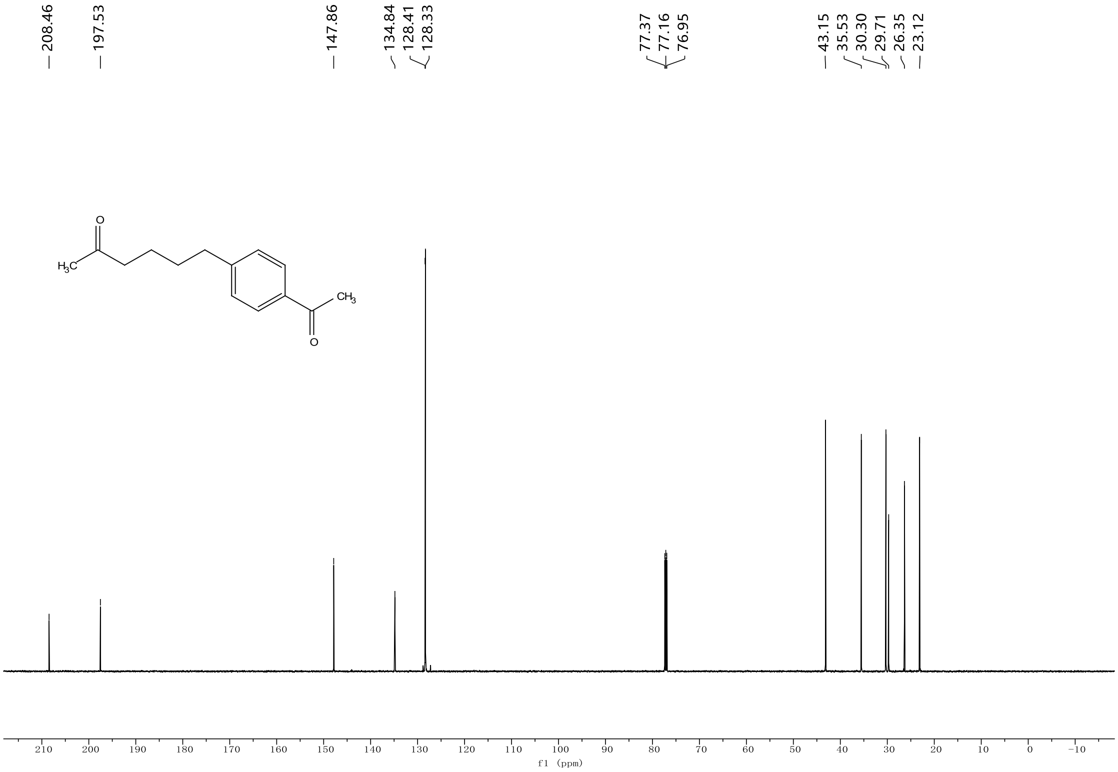

Supplementary Figure 130. ^{13}C NMR spectra of compound **48** (151 MHz, r.t., $CDCl_3$).

Compound 49

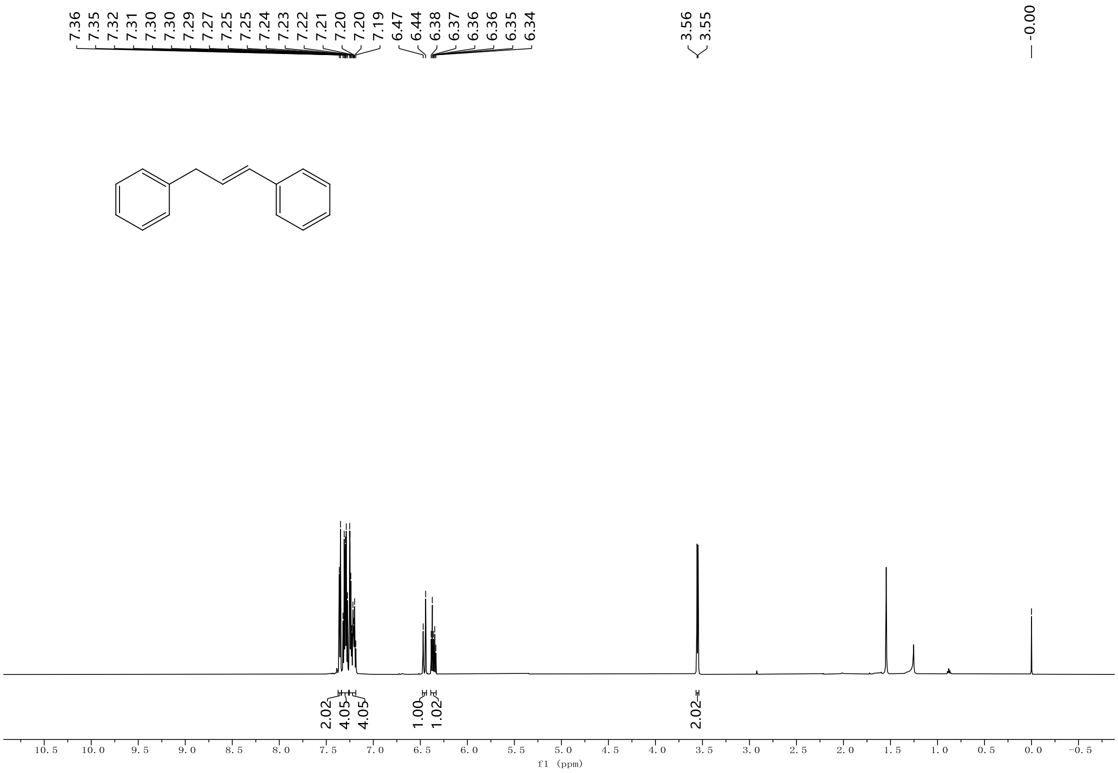

Supplementary Figure 131. ^{1}H NMR spectra of compound **49** (600 MHz, r.t., $CDCl_3$).

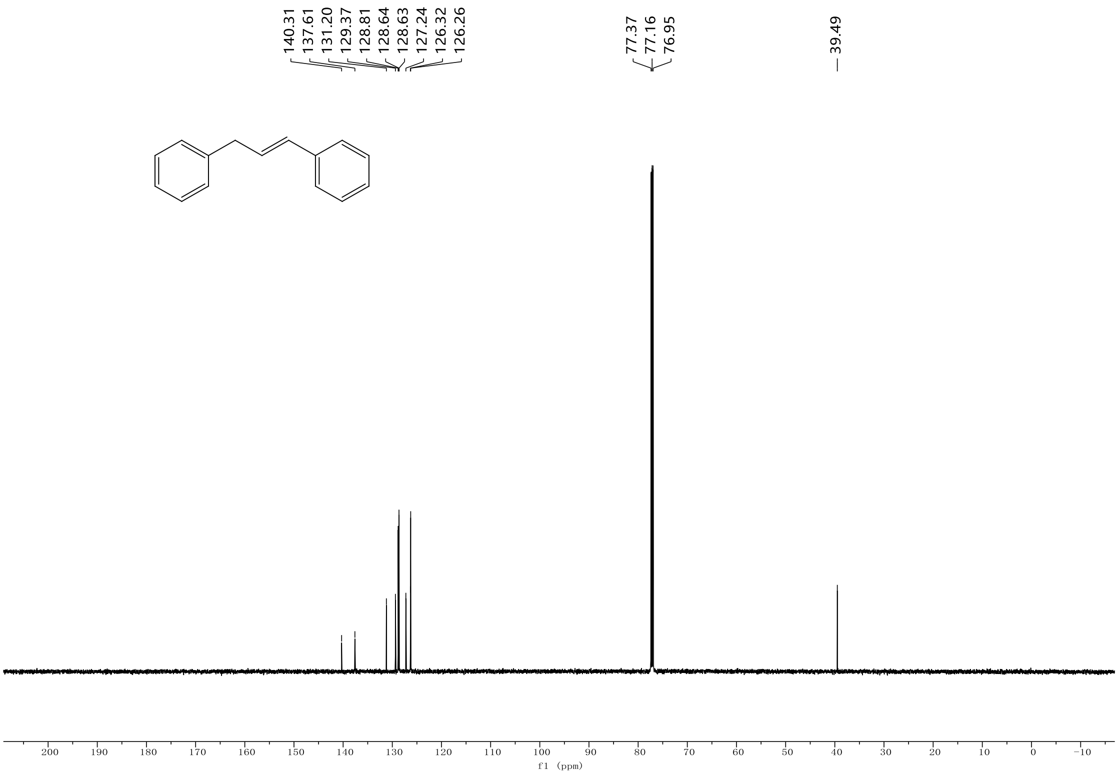

Supplementary Figure 132. ^{13}C NMR spectra of compound **49** (151 MHz, r.t., $CDCl_3$).

Compound 50

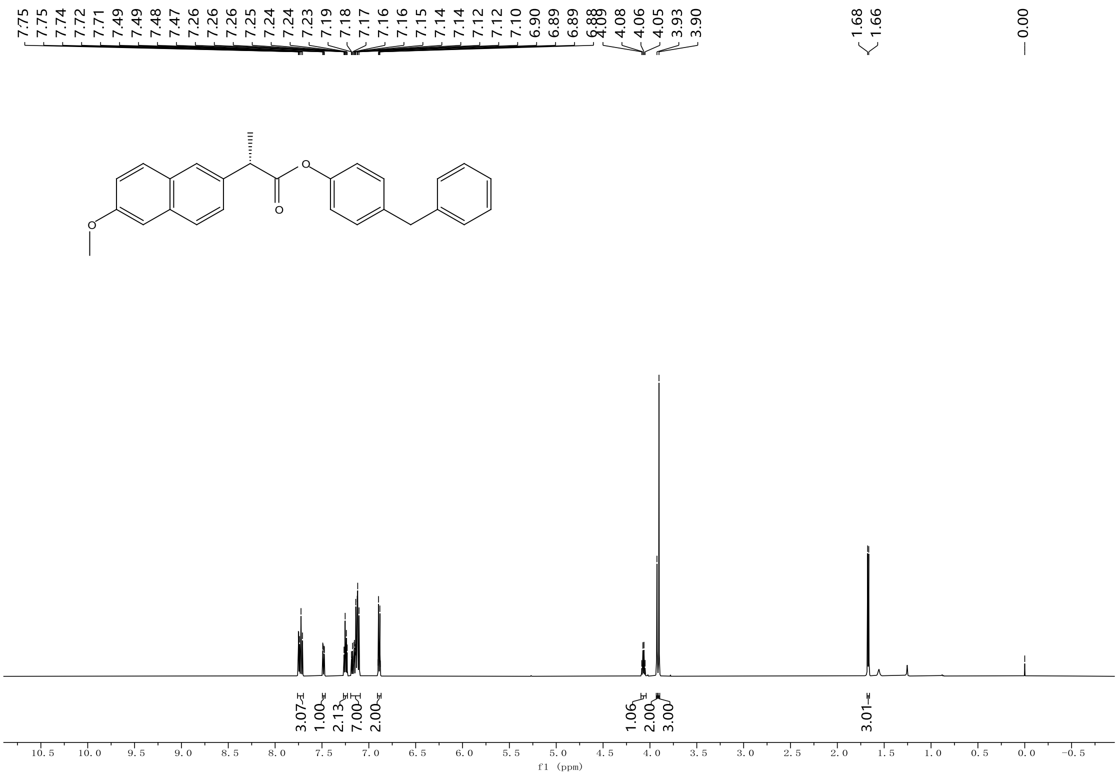

Supplementary Figure 133. ^{1}H NMR spectra of compound **50** (600 MHz, r.t., $CDCl_3$).

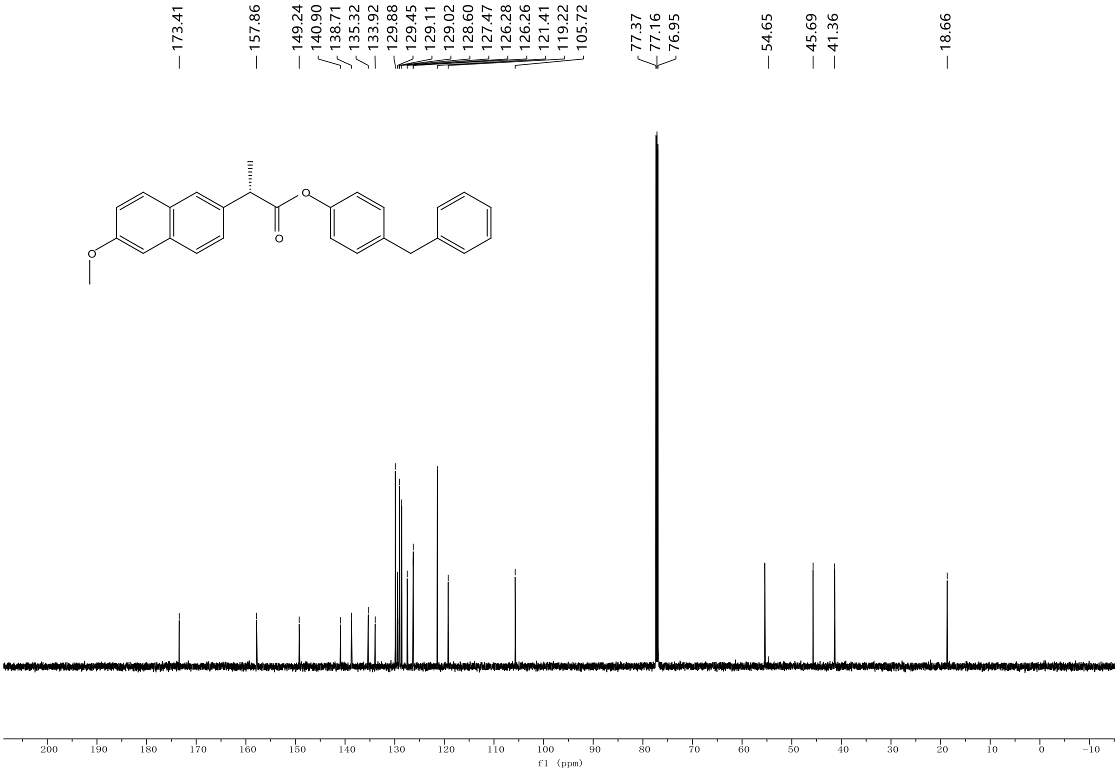

Supplementary Figure 134. ^{13}C NMR spectra of compound **50** (151 MHz, r.t., $CDCl_3$).

Compound 51

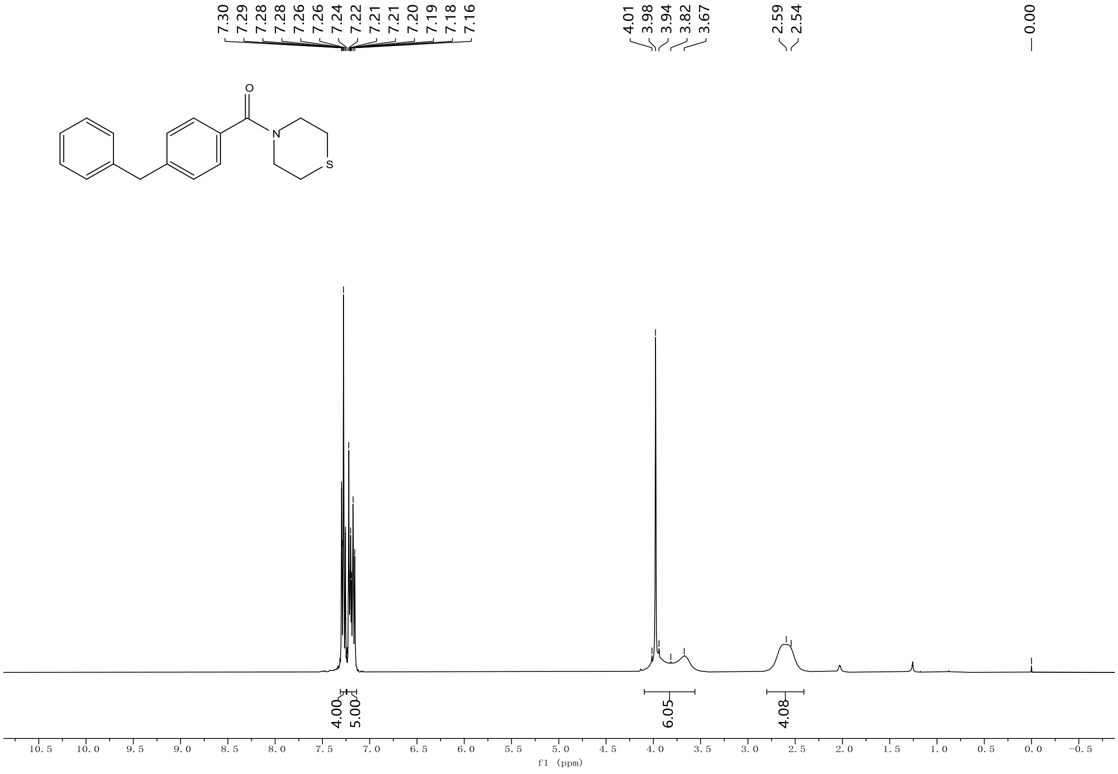

Supplementary Figure 135. ^{1}H NMR spectra of compound **51** (400 MHz, r.t., $CDCl_3$).

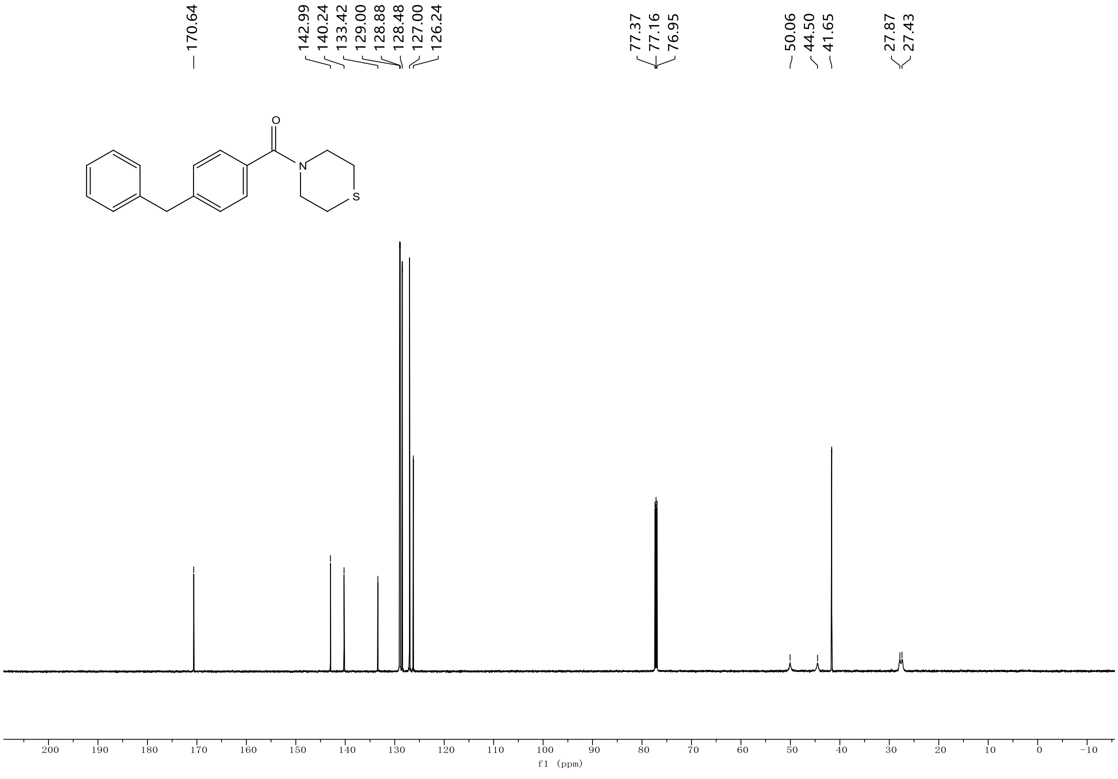

Supplementary Figure 136. ^{13}C NMR spectra of compound **51** (151 MHz, r.t., $CDCl_3$).

Compound 52

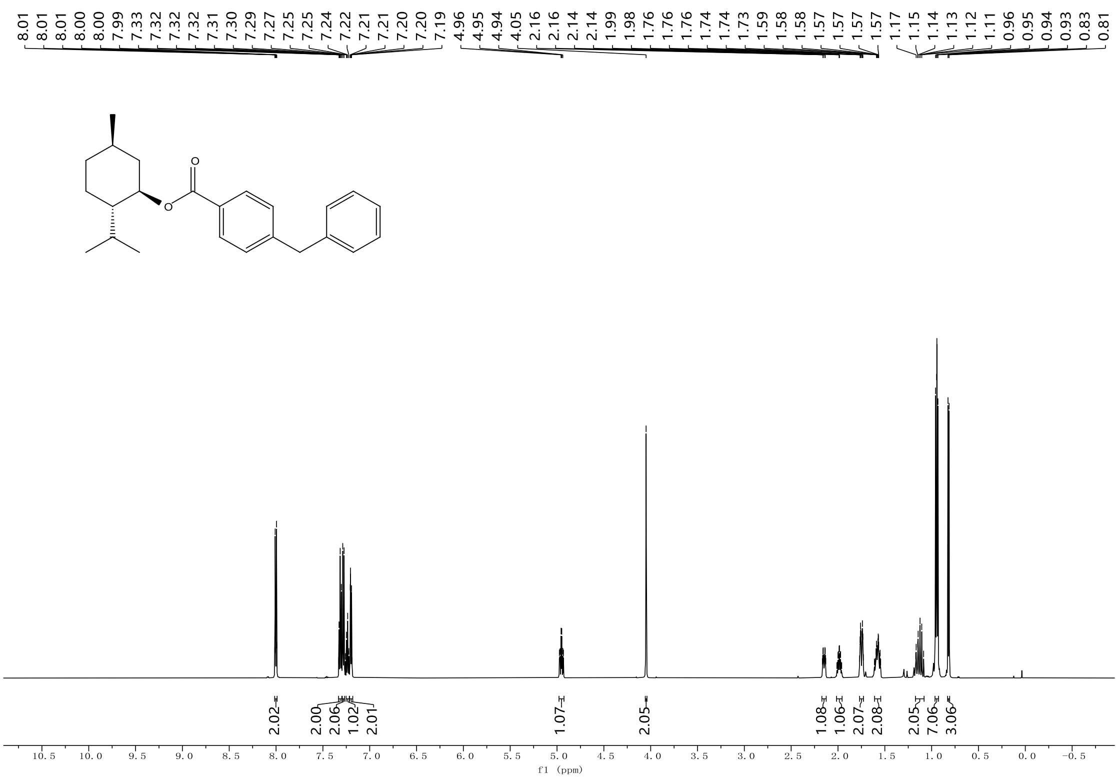

Supplementary Figure 137. ^{1}H NMR spectra of compound **52** (600 MHz, r.t., $CDCl_3$).

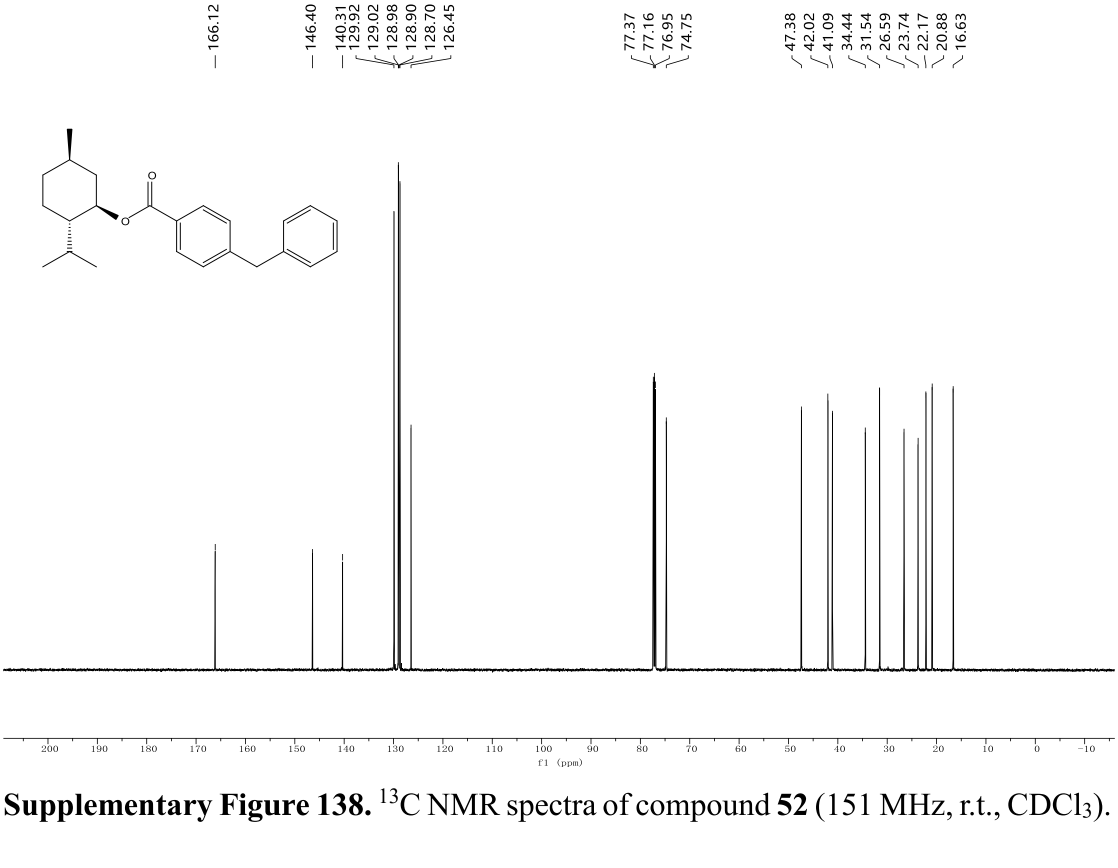

Supplementary Figure 138. ^{13}C NMR spectra of compound **52** (151 MHz, r.t., $CDCl_3$).

Compound 53

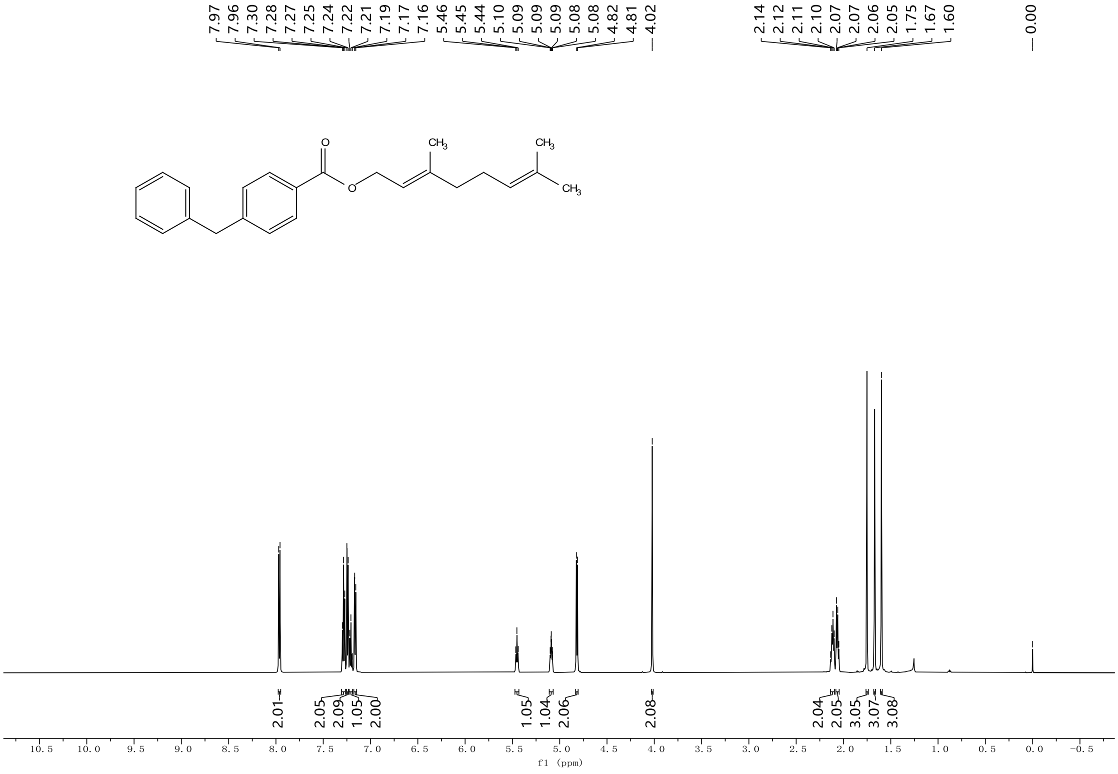

Supplementary Figure 139. ^{1}H NMR spectra of compound **53** (600 MHz, r.t., $CDCl_3$).

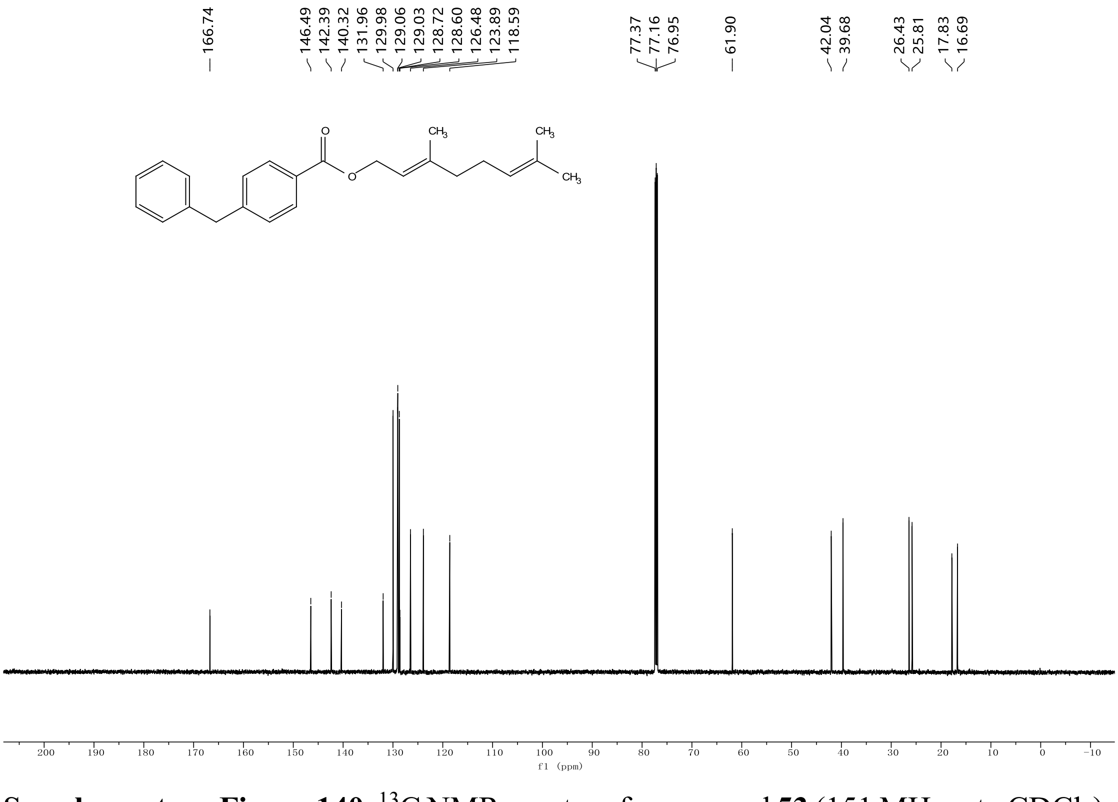

Supplementary Figure 140. ^{13}C NMR spectra of compound **53** (151 MHz, r.t., $CDCl_3$).

Compound 54

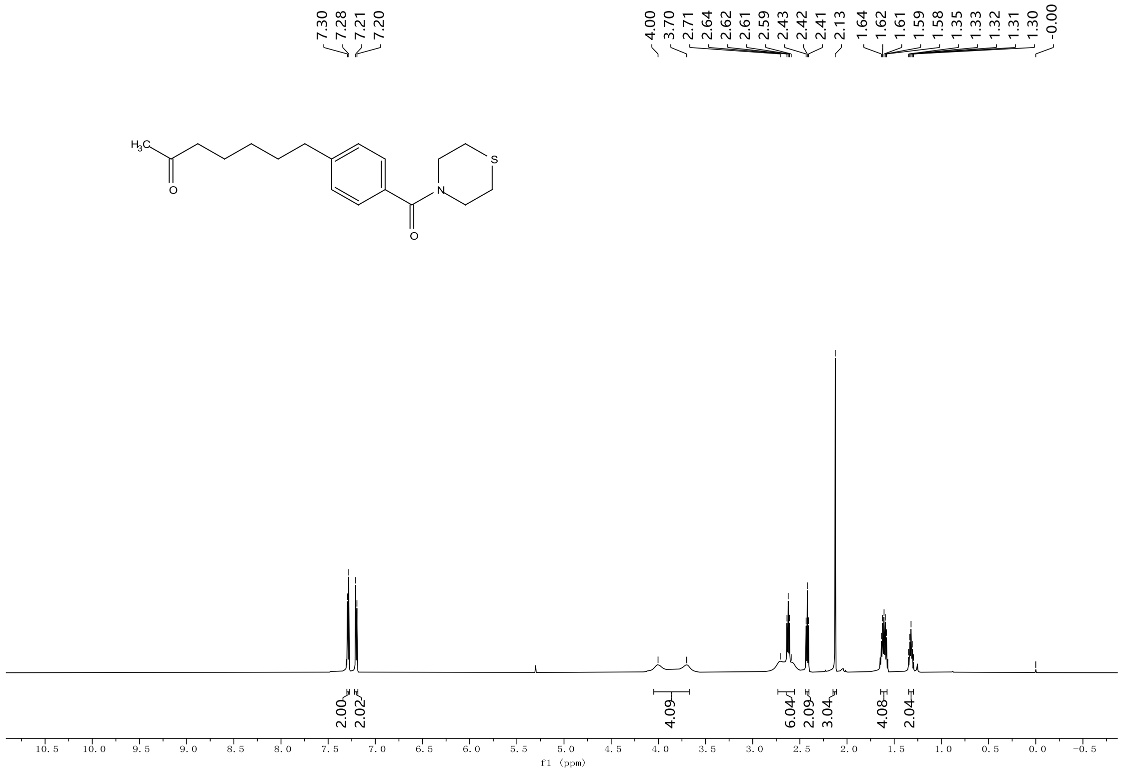

Supplementary Figure 141. ^{1}H NMR spectra of compound **54** (600 MHz, r.t., $CDCl_3$).

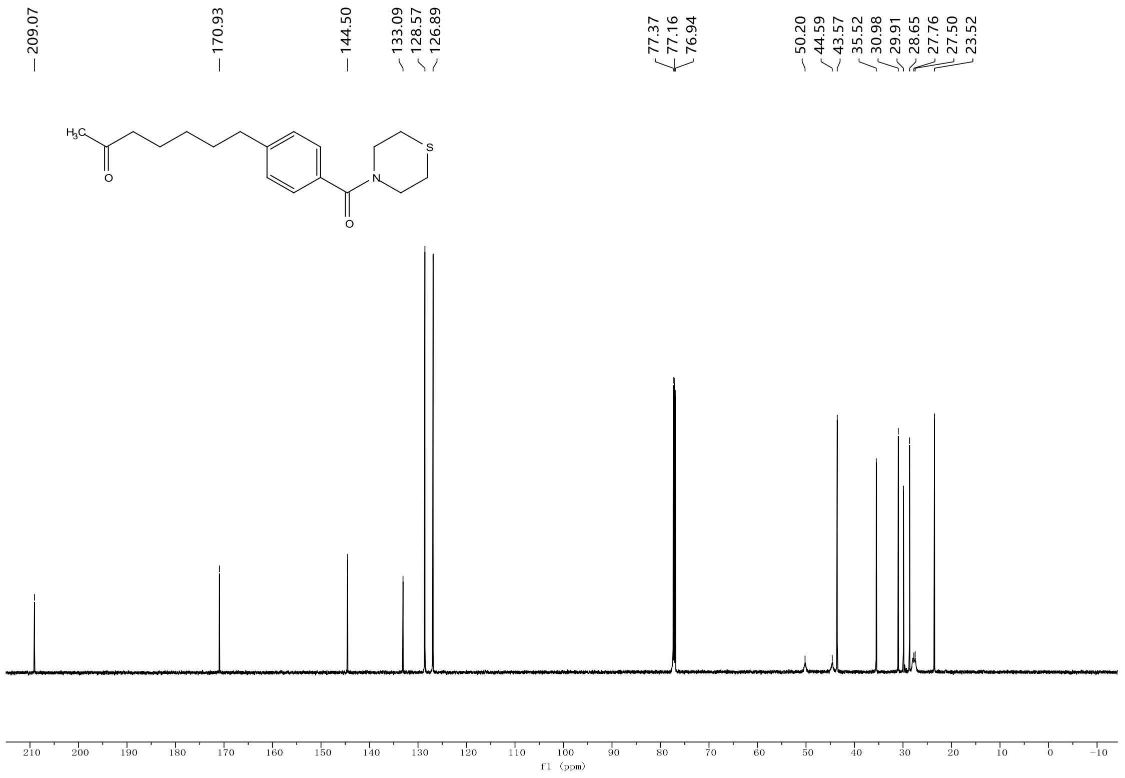

Supplementary Figure 142. ^{13}C NMR spectra of compound **54** (151 MHz, r.t., $CDCl_3$).

Compound 55

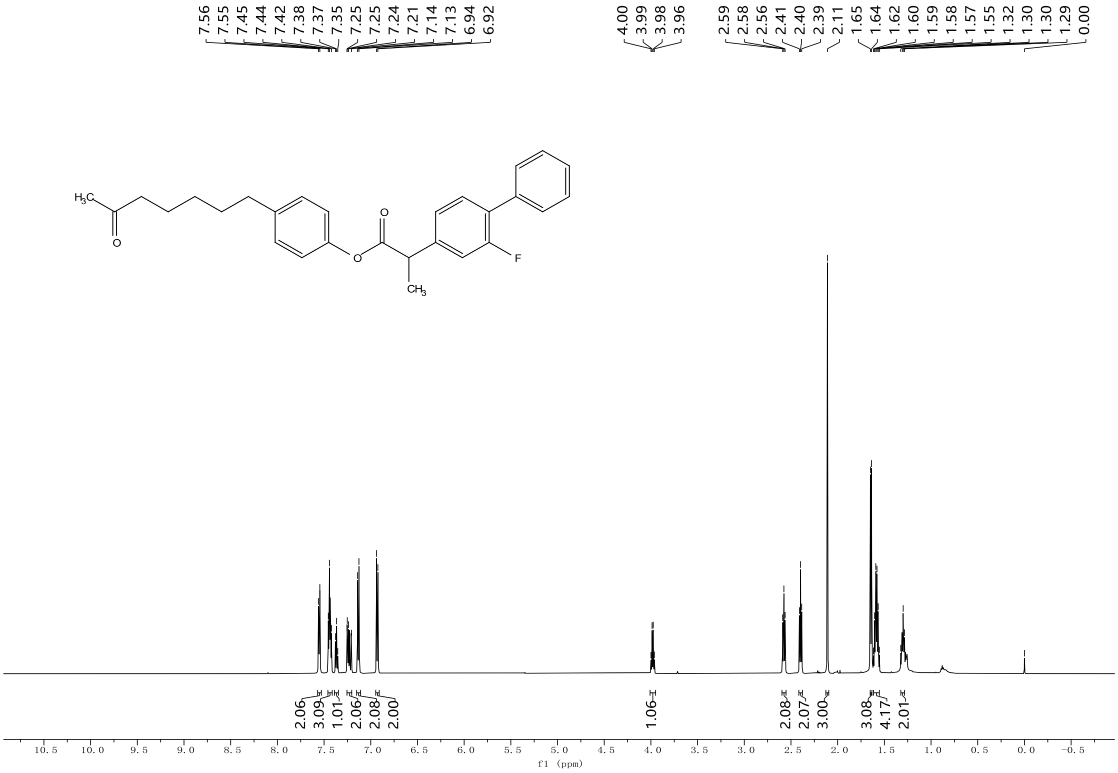

Supplementary Figure 143. ^{1}H NMR spectra of compound **55** (600 MHz, r.t., $CDCl_3$).

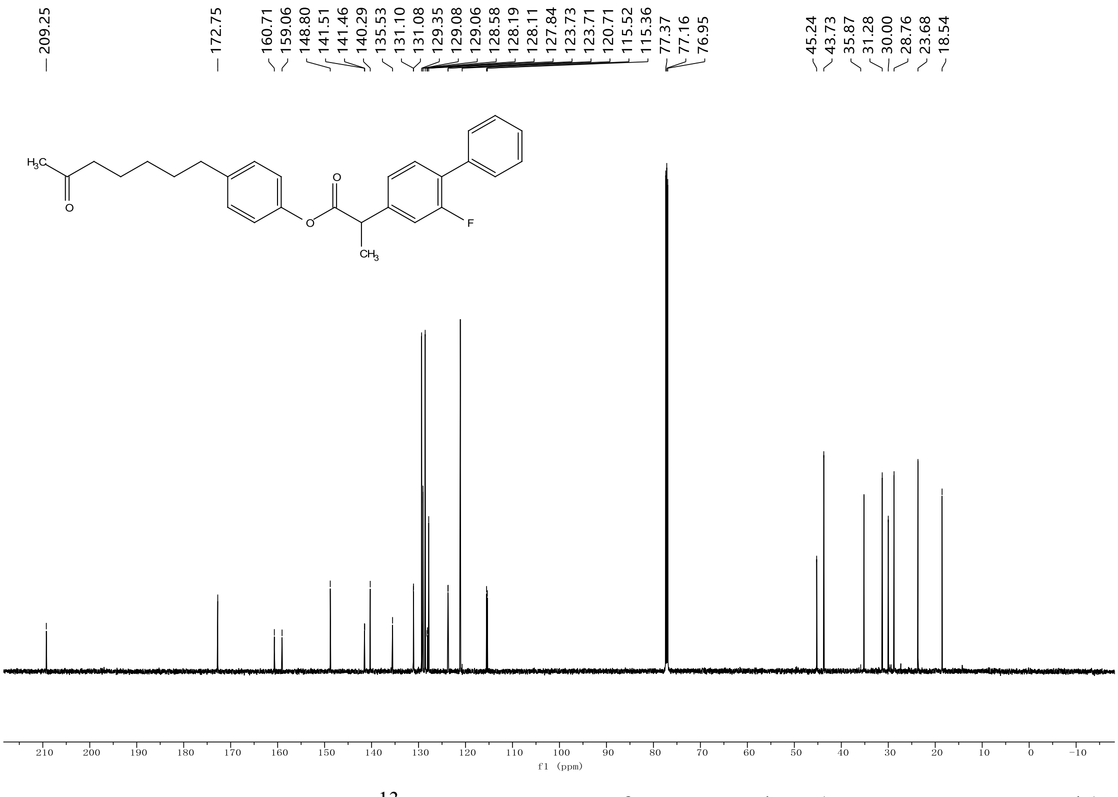

Supplementary Figure 144. ^{13}C NMR spectra of compound **55** (151 MHz, r.t., $CDCl_3$).

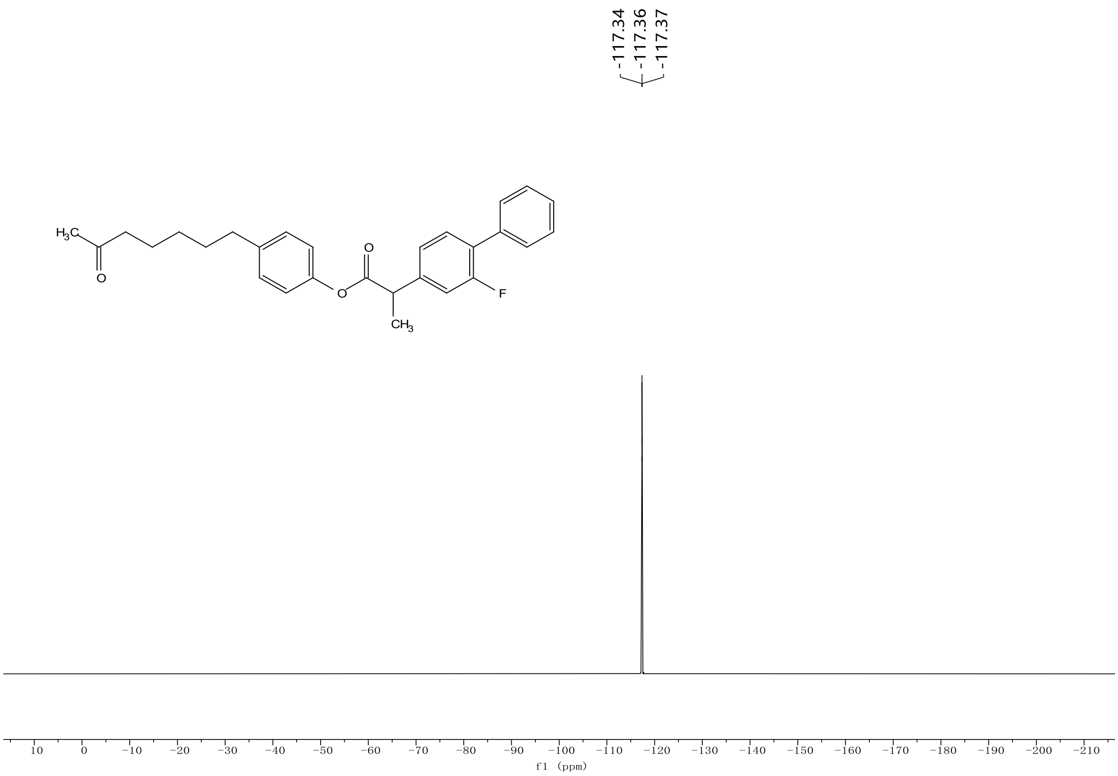

Supplementary Figure 145. ^{19}F NMR spectra of compound **55** (565 MHz, r.t., $CDCl_3$).

Compound 56

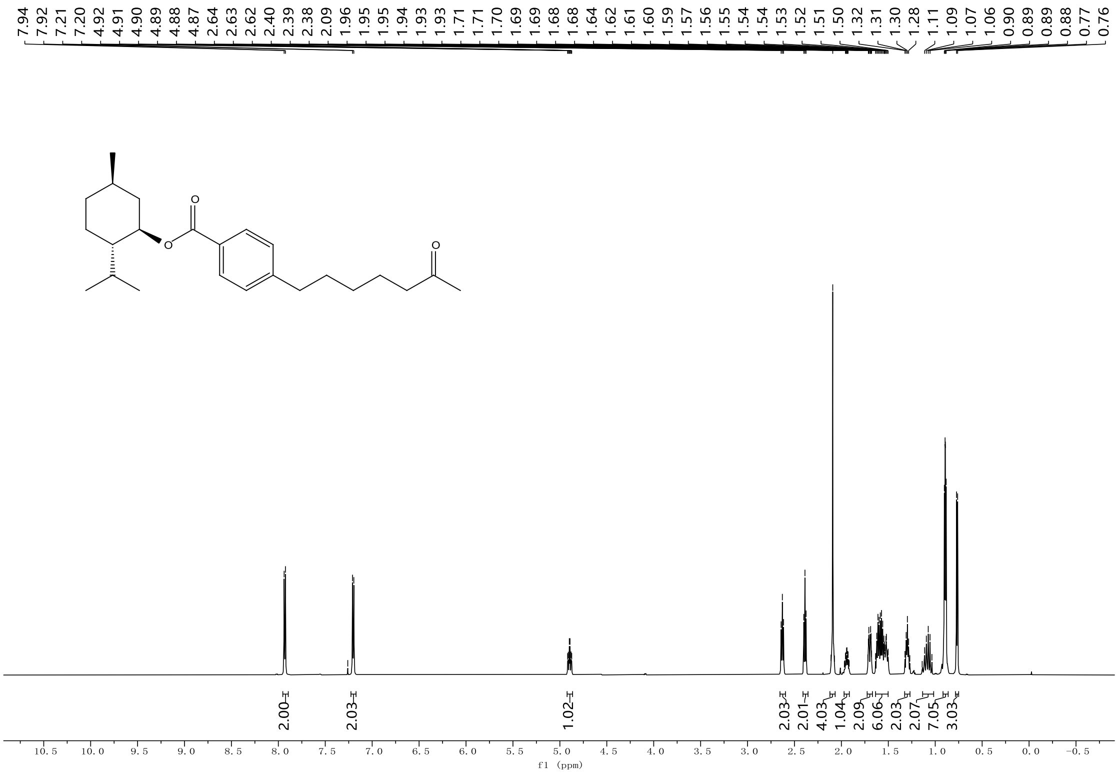

Supplementary Figure 146. ^{1}H NMR spectra of compound **56** (600 MHz, r.t., $CDCl_3$).

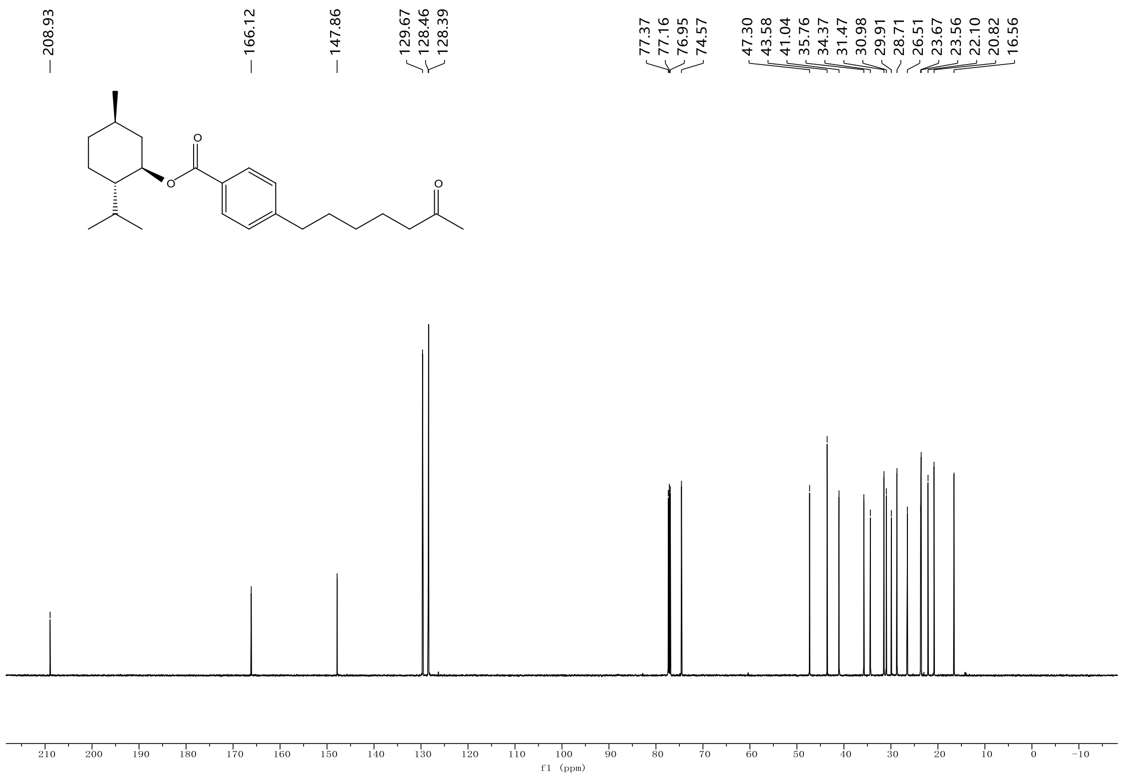

Supplementary Figure 147. ^{13}C NMR spectra of compound **56** (151 MHz, r.t., $CDCl_3$).

Compound 57

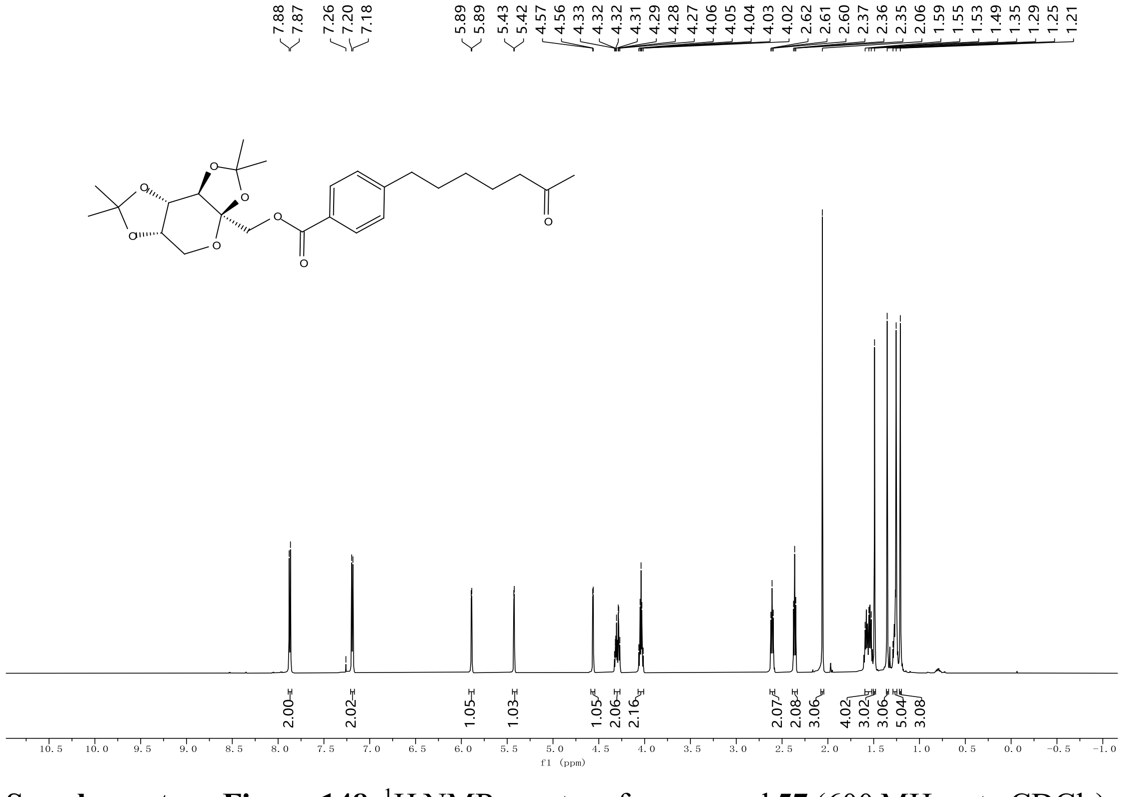

Supplementary Figure 148. ^{1}H NMR spectra of compound **57** (600 MHz, r.t., $CDCl_3$).

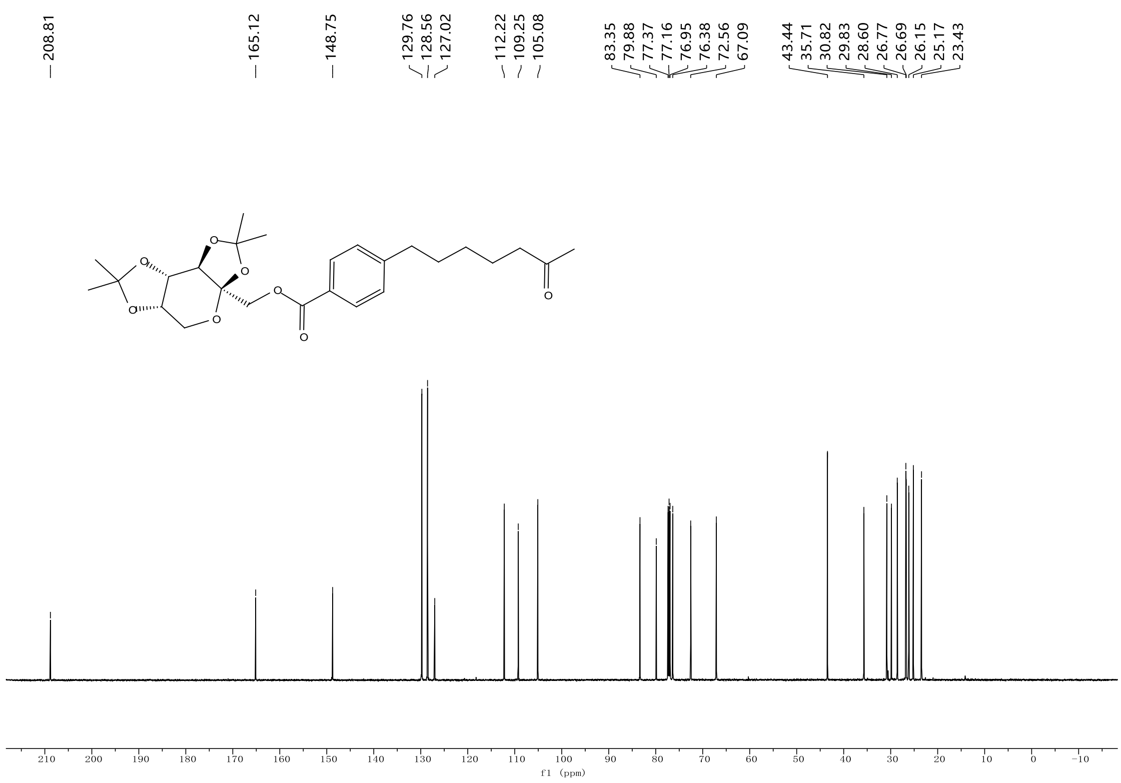

Supplementary Figure 149. ^{13}C NMR spectra of compound **57** (151 MHz, r.t., $CDCl_3$).

4 Supplementary References

[1] Zou, L.; Xiang, S.-Q.; Sun, R.; Lu, Q.-Q. Selective c(sp^3)−H arylation/alkylation of alkanes enabled by paired electrocatalysis, *Nat. Commun.,* **2023**, *14,* 7992.

[2] He, X.-C.; Li, K.-R.; Gao, J.; Guan, J.-P.; Chen, H.-B.; Xiang, H.-Y.; Chen, K.; Yang, H. Photoexcited Ni^{II}–aryl complex-mediated giese reaction of aryl bromides, *Org. Lett.* **2023**, *25,* 4056.

[3] Sun, Y.-F.; Qiao, H.-L.; Ling, Y.; Yang, S.-X.; Rui, S.-X.; Pelosi, P.; Yang, X.-L. New Analogues of (*E*)-*β*-farnesene with insecticidal activity and binding affinity to aphid odorant-binding proteins, *J. Agric. Food Chem.,* **2011**, *59,* 2456.

[4] Yuma, K.; Yasushi, I.; Yohei, O.; Chiba, K. Electrochemical amide bond formation from benzaldehydes and amines: oxidation by cathodic-generated hydrogen peroxide, *Eur. J. Org. Chem.,***2020**, *25,* 3844.

[5] Xu, Q.-H.; Wei, L.-P.; Xiao, B. Alkyl-$GeMe_3$: neutral metalloid radical precursors upon visible-light photocatalysis, *Angew. Chem. In. Ed.*, **2022**, *61*, e202115592.

[6] Li, J.-B.; Huang, C.-Y.; Li, C.-J. Two-in-one metallaphotoredox cross-couplings

enabled by a photoactive ligand, *Chem*., **2022**, *8*, 2419.
[7] Hu, X.-L.; Zhang, L.; Nickel catalysis enables convergent paired electrolysis for direct arylation of benzylic C–H bonds, *Chem. Sci.*, **2020**,*11*, 10786
[8] Prantik M.; Danielle M.; Shacklady-McAtee; Glenn P. A.; Yap, Eric, R. Sirianni; Mary P. Watson. Nickel-catalyzed cross couplings of benzylic ammonium salts and boronic acids: stereospecific formation of diarylethanes via C–N bond activation, *J. Am. Chem. Soc.*, **2013**, *135*, 280.
[9] Li, M.; Tian, Y.; Sun, K.-H.; Xu, Z.-M.; Tian, L.-F.; Wang, Y.-H. Two-in-one metallaphotoredox cross-couplings enabled by a photoactive ligand, *Chem. Commun.*, **2023**, *59*, 5587.
[10] Hayashi, Sayuri; Hirano, Koji; Yorimitsu, Hideki; Oshima, Koichiro. Palladium-catalyzed stereo- and regiospecific allylation of aryl halides with homoallyl alcohols via retro-allylation: selective generation and use of σ-allylpalladium, *J. Am. Chem. Soc.*, **2006**, *128*, 2210.
[11] Zuo, Z.-W.; Ahneman, Derek T.; Chu, L.-L.; Terrett, Jack A.; Doyle, Abigail G.; MacMillan, David W. C., Merging photoredox with nickel catalysis: coupling of α-carboxyl sp^3-carbons with aryl halides, *Science*, **2014**, *345*, 437.
[12] Martin, Ruben; Fuerstner, Alois. Nickel-catalyzed ross couplings of benzylic ammonium salts and boronic acids: stereospecific formation of diarylethanes via C–N bond activation, *Angew. Chem. In. Ed.*, **2004**, *43*, 3955.
[13] Xie, L.-G.; Wang, Z.-X.; Nickel-catalyzed cross-coupling of aryltrimethylammonium iodides with organozinc reagents, *Angew. Chem. In. Ed.*, **2011**, *50*, 4901.
[14] Lv, L.-Y.; Zhu, D.-H.; Tang, J.-T.; Qiu, Z.-H.; Li, C.-C.; Gao, J.; Li, C.-J.; Cross-coupling of phenol derivatives with umpolung aldehydes catalyzed by nickel, *ACS Catal.*, **2018**, *8*, 4622.
[15] Luo, J.; Hu, B.; Wu, W.-D.; Hu, M.-W.; Liu, T. Leo. Nickel-catalyzed electrochemical $C(sp^3)–C(sp^2)$ cross-coupling reactions of benzyl trifluoroborate and organic halides, *Angew. Chem. In. Ed.*, **2021**, *60*, 6107.

[16] McLaughlin, Suzuki−miyaura cross-coupling of benzylic phosphates with arylboronic acids, *Org. Lett.*, **2005**, *7*, 4875.

[17] Dan, X.; Yang, Q.; Xing, L.-Z.; Tang, Y.-R.; Wang, W.-T.; Cai, Y.-F. Heterogeneous metallaphotocatalytic C(sp^2)–C(sp^3) cross-coupling reactions with integrated bipyridyl-ni(II)-carbon nitride. *Org. Lett.*, **2023**, *25*, 4124.

[18] Xia, Y.-M.; Hu, F.-D.; Xia, Y.; Liu, Z.-X.; Ye, F.; Zhang, Y.; Wang, J.-B. Synthesis of di- and triarylmethanes through palladium-catalyzed reductive coupling of N-tosylhydrazones and aryl bromides, *Synthesis*, **2017**, *49*, 1073.

[19] Puy, Michael Van Der. Direct fluorination of substituted pyridines, *Tetrahedron Lett.*, **1987**, *28*, 255.

[20] Aragón, Jordi; Jaworski, Sebastian; Lloret-Fillol, Julio; Pascual, David; Sun, Suyun. Photoredox activation of inert alkyl chlorides for the reductive cross-coupling with aromatic alkenes, *Angew. Chem. In. Ed.*, **2022**, *61*, e202114365.

[21] Gary A. Molander; Takatoshi Ito. Cross-coupling reactions of potassium alkyltrifluoroborates with aryl and 1-alkenyl trifluoromethanesulfonates, *Org. Lett.*, **2001**, *3*, 393.

[22] Zhang, H.; Huang, X.-L. Ligand-free heck reactions of aryl iodides: significant acceleration of the rate through visible light irradiation at ambient temperature, *Adv. Synth. Catal.*, **2016**, *358*, 3736.

[23] Gao, X.; Zhou, J.-H.; Peng, X.-H. Efficient palladium(0) supported on reduced graphene oxide for selective oxidation of olefins using graphene oxide as a solid weak acid, *Catal. Commun.*, **2019**, *122*, 73.
